# Mechanistic Insights about the Ligand‐Enabled Oxy‐arylation/vinylation of Alkenes via Au(I)/Au(III) Catalysis

**DOI:** 10.1002/chem.202202110

**Published:** 2022-08-25

**Authors:** Mathilde Rigoulet, Karinne Miqueu, Didier Bourissou

**Affiliations:** ^1^ CNRS/Université Paul Sabatier UPS Laboratoire Hétérochimie Fondamentale et Appliquée (LHFA UMR 5069) 118 route de Narbonne 31062 Toulouse France; ^2^ CNRS/Université de Pau et des Pays de l'Adour E2S-UPPA Institut des Sciences Analytiques et de Physico-Chimie pour l'Environnement et les Matériaux (IPREM UMR 5254) Hélioparc, 2 Avenue du Président Angot 64053 Pau Cedex 09 France

**Keywords:** complex density, functional theory, gold, mechanism, regioselectivity

## Abstract

The mechanism of oxy‐arylation/vinylation of alkenes catalyzed by the (MeDalphos)AuCl complex was comprehensively investigated by DFT. (P,N)Au(Ph)^2+^ and (P,N)Au(vinyl)^2+^ are key intermediates accounting for the activation of the alkenols and for their cyclization by outer‐sphere nucleophilic attack of oxygen. The 5‐*exo* and 6‐*endo* paths have been computed and compared, reproducing the peculiar regioselectivity difference observed experimentally between 4‐penten‐1‐ol, (*E*) and (*Z*)‐4‐hexen‐1‐ols. Examining the way the alkenol coordinates to gold (more η^*2*^ or η^*1*^) can offer, in some cases, a simple way to predict the favored path of cyclization.

## Graphical abstract

**DFT calculations** provide valuable insights into the mechanism of the oxy‐arylation/vinylation of akenes catalyzed by gold. The way alkenols coordinate to (MeDalphos)Au(R’)^2+^ (R’=phenyl, vinyl) was found to significantly depend on their substitution pattern (terminal, *Z*/*E* internal) and to play a major role in the 5‐*exo*/6‐*endo* regioselectivity.

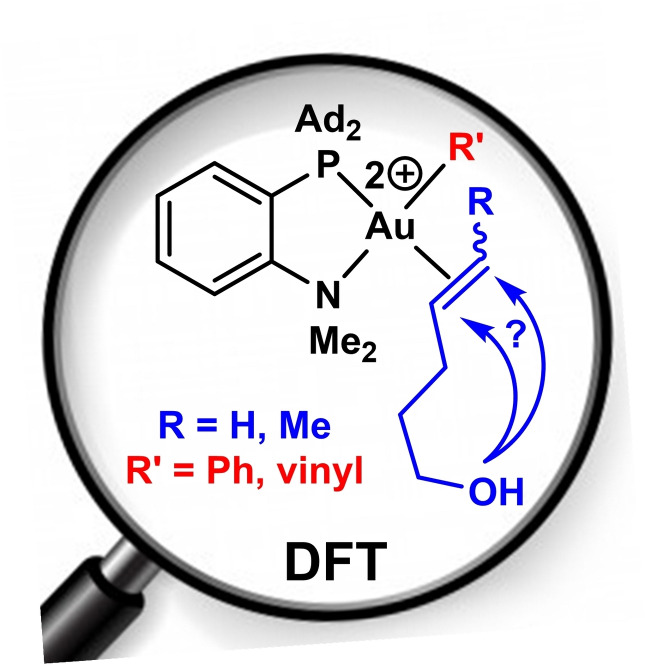

## Introduction

**Figure 1:**
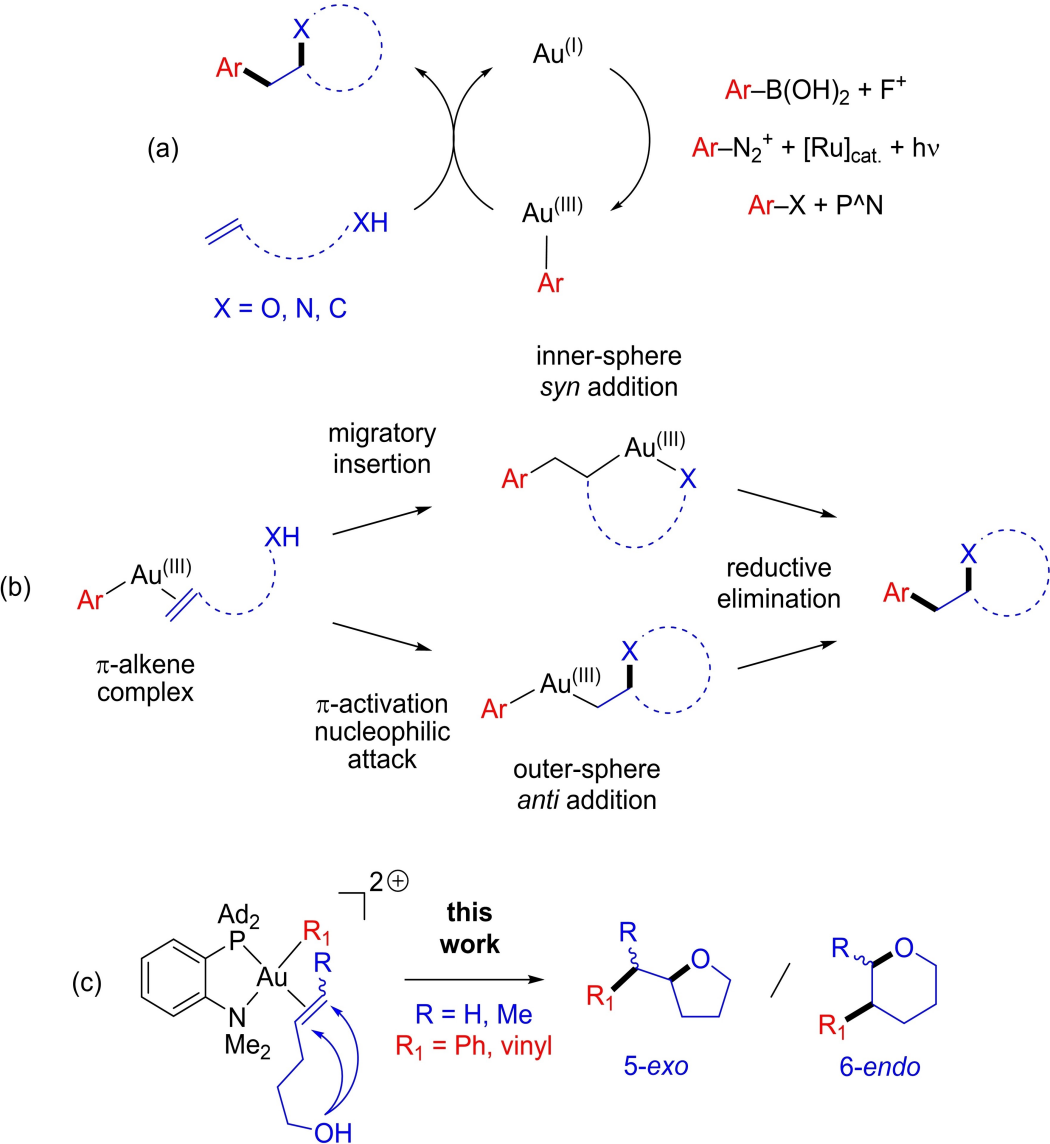
General representations of Au(I)/Au(III)‐catalyzed 1,2‐difunctionalizations of alkenes (a) and the associated inner/outer‐sphere paths (b); reactions considered in this work (c).

Over the past 10 years, the repertoire of gold catalysis has been extended to Au(I)/Au(III) catalysis, with three complementary approaches based on external oxidants, photoredox conditions and most recently ligand‐enabled oxidative addition.^[1]^ The associated synthetic developments include C−C and C−X (X=N, S, Se …) cross‐couplings, but also the combination of Au(I)/Au(III) cycles with π‐activation at gold.^[2]^ This enables to perform a variety of intra as well as intermolecular 1,2‐difunctionalization reactions of alkenes, with O, N and C‐based nucleophiles (Figure 1a).^[3]^

It is to note that gold is not a simple copy of the other transition metals in these transformations. It displays complementary behavior, in particular high functional group tolerance.^[4]^ Moreover, the aforementioned oxidative/photoredox/ligand‐enabled approaches result in different reaction profiles of gold with respect to the preference for electron‐enriched/deprived aryl substrates,[^2h^, ^5^] as well as *exo*/*endo* regioselectivity.[^2c^, ^2h^]

These synthetic developments have stimulated mechanistic studies to better understand the way the reactions proceed and what influence them. From an experimental viewpoint, the stereochemical outcome of the reaction (*syn*/*anti* addition across the C=C double bond as most often determined by D‐labeling experiments) was used as reporter to distinguish inner‐ and outer‐sphere paths (Figure 1b). All reactions reported to date apparently follow the outer‐sphere path, whatever the conditions (external oxidants, light‐mediated or ligand‐enabled).[^2a^, ^2c^, ^2h^]

Two computational studies have also been reported recently.[^6^, ^7^, ^8^] In 2016, Yu and co‐workers studied the oxy‐arylation of alkenes under dual gold photoredox conditions, showing that Au(III)‐aryl complexes are first generated by radical addition to gold and single electron transfer, followed by coordination of the alkene, cyclization and reductive elimination.^[7]^ Earlier this year, Zhang and co‐workers investigated the ligand‐enabled 1,2‐diarylation of alkenes, considering both the coupling of aryl alkenes with aryl iodides and that of iodoaryl alkenes with indoles.^[8]^ The reactions were found to involve π‐activation not migratory insertion, and the *exo*/Markovnikov regioselectivities observed experimentally were nicely reproduced theoretically.

Here we report a complementary Density Functional Theory (DFT) study we have carried out on the intramolecular oxy‐arylation/vinylation of alkenes (Figure 1c). We have shown these reactions to be efficiently catalyzed by the (MeDalphos)AuCl complex (thanks to ligand‐enabled oxidative addition). The outer‐sphere path was supported experimentally by the observed *trans* selectivity of the alkene difunctionalization. Moreover, an unprecedented switch of regioselectivity, 5‐*exo* vs. 6‐*endo* cyclization, was noticed between *Z*‐ and *E*‐substituted internal alkenols. Special attention was thus given to the influence of the substitution pattern of the alkene: terminal, *Z*/*E*‐internal. The geometry and electronic structures of the key π‐alkene Au(III) complex, prior to cyclization by outer‐sphere nucleophilic attack, turned to be decisive.

## Results and Discussion

**Scheme 1:**
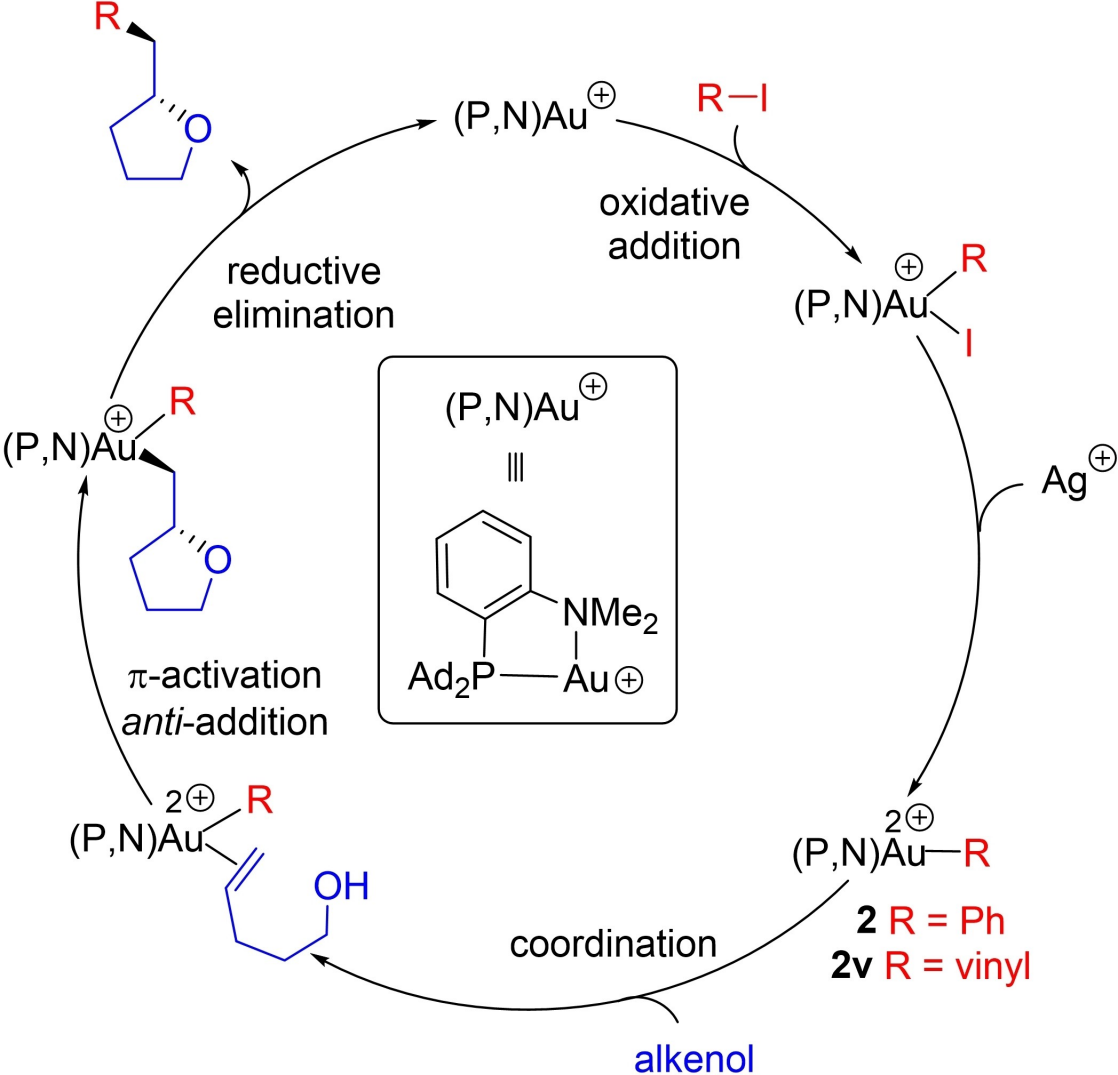
Simplified catalytic cycle proposed to account for the 1,2‐oxy‐arylation/vinylation of alkenols catalyzed by the (P,N)AuCl complex (with 4‐penten‐1‐ol as model substrate, only showing the 5‐*exo* cyclization product obtained experimentally).

The outer‐sphere catalytic cycle proposed to account for the oxy‐arylation/vinylation of alkenes catalyzed by (MeDalphos)AuCl is displayed in Scheme 1. It starts by oxidative addition of the aryl/vinyl iodide substrate triggered by the hemilabile (P,N) ligand. Following iodide abstraction by the silver salt, the alkenol coordinates to gold and the pendant hydroxyl group attacks the C=C double bond (*anti* to gold). Finally, reductive elimination induces C(sp^2^)−C(sp^3^) coupling and releases the product. The oxidative addition step has already been comprehensively studied experimentally and computationally.[^5a^, ^9^] The outer‐sphere mechanism was supported experimentally over the alternative inner‐sphere pathway (involving migratory insertion) by the selective formation of *anti* addition products from internal alkenols (as well as D‐labeled *N*‐tosyl pent‐4‐enyl amines),^[2h]^ and computationally by Zhang and co‐workers in their recent study of 1,2‐diarylation reactions.^[8]^ In this work, we focused on the activation, cyclization and arylation/vinylation of the alkenols at gold. To this end, we studied computationally the structure and reactivity of the key (P,N)Au(Ph)^2+^/(P,N)Au(vinyl)^2+^ complexes **2**/**2 v**, considering terminal as well as internal alkenols and giving special attention to the regioselectivity (5‐*exo* vs. 6‐*endo* cyclization).

The main objective of this computational study was to gain further mechanistic insight on such gold‐catalyzed transformations. In particular, it was our aims (*i*) to identify the key intermediates and analyze their structure/reactivity, and (*ii*) to compute and compare the different cyclization modes to shed light into the regioselectivity (5‐*exo* vs. 6‐*endo*) and better understand the influence of the alkenol substitution pattern (terminal, *Z*/*E* internal).

The calculations were carried out on the real systems, without simplification of the (P,N) ligand, at the SMD(CH_2_Cl_2_)‐B3PW91‐D3(BJ)/SDD+f(Au), 6‐31+G** (other atoms)//B3PW91/SDD+f(Au), 6‐31G** (other atoms) level of theory. Solvent and dispersion effects were taken into account, but not the counter‐anion.^[10]^

**Figure 2:**
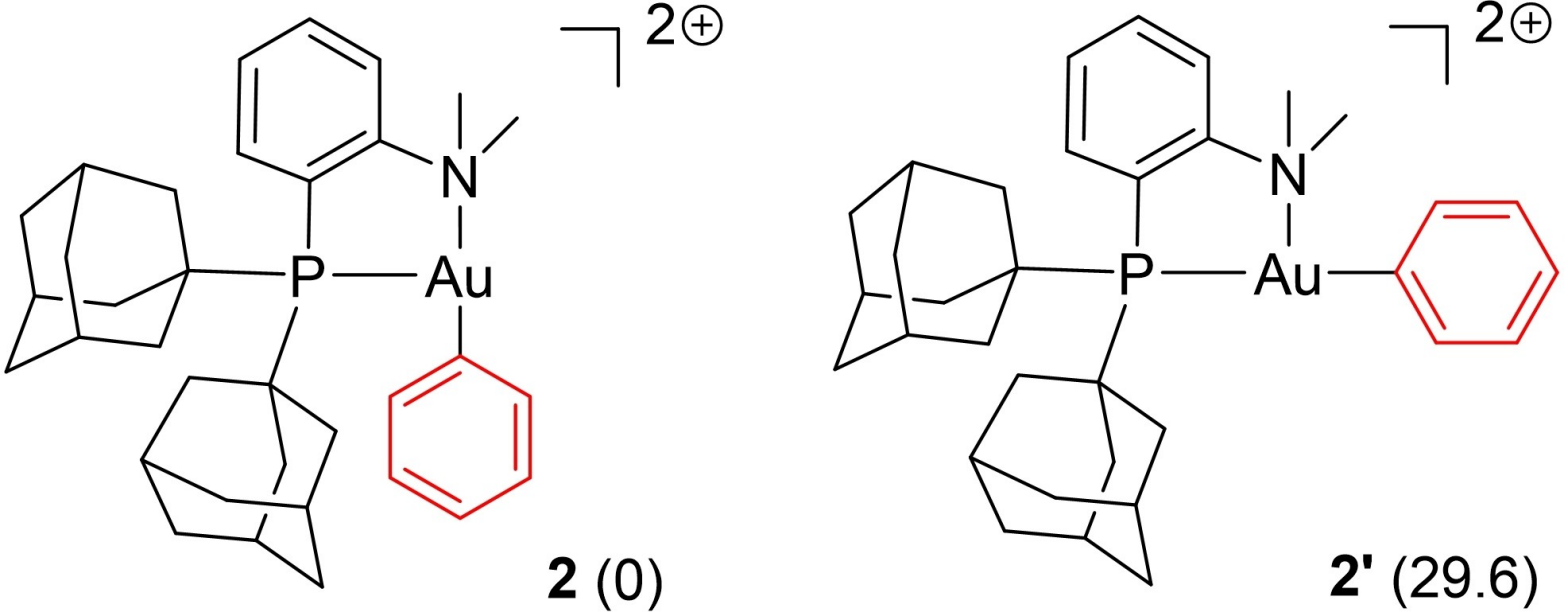
*Cis* and *trans* forms of the (P,N)Au(Ph)^2+^ complex. Relative Gibbs free energies in parentheses, in kcal/mol.

To begin with, we investigated the oxy‐phenylation reaction and studied the (P,N)Au(Ph)^2+^ complex, the key reactive species towards alkenols. Two *minima* were located on the potential energy surface (PES). In both cases, the 3‐coordinate gold center adopts T‐shape geometry with the phenyl group in *trans* position either to nitrogen (**2**) or phosphorus (**2’**) (Figure 2).^[11]^ The (P,N) ligand is highly dissymmetric electronically: the phosphine is a stronger σ‐donor ligand than the amine and exerts a stronger *trans* influence. Consistently, complex **2** with the phenyl group *trans* to nitrogen was found to be much more stable than **2’**. Considering the very large energy gap between the two isomers (29.6 kcal/mol), only **2** was then considered.

### Oxy‐arylation of 4‐penten‐1‐ol (terminal alkenol)

**Scheme 2:**
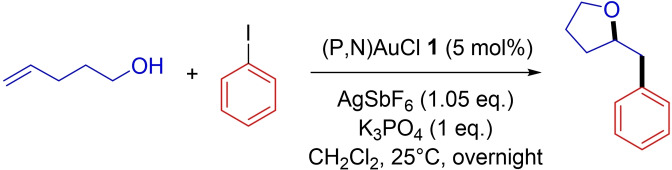
Catalytic oxy‐phenylation of 4‐penten‐1‐ol with the (P,N) gold(I) complex **1**.^[2h]^

The reaction of the (P,N)Au(Ph)^2+^ complex **2** with 4‐penten‐1‐ol was explored first, as benchmark oxy‐arylation reaction proceeding exclusively by 5‐*exo* cyclization to give a 2‐benzyltetrahydrofuran (Scheme 2).^[2h]^

**Scheme 3:**
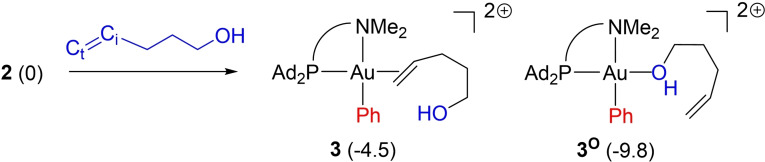
π and O‐adducts, respectively (**3**) and (**3^O^**), resulting from the coordination of 4‐penten‐1‐ol to the (P,N)Au(Ph)^2+^ complex **2**. Relative Gibbs free energies in parentheses, in kcal/mol. The terminal and internal C=C atoms of the alkenol are labeled C_t_ and C_i_, respectively.

The alkenol can bind to gold via either the C=C double bond or the oxygen atom. Energy *minima* for the two coordination modes were localized on the potential energy surface (Scheme 3, Figure S2).^[12]^ In the π‐complex **3**, the C=C double bond is oriented about perpendicular to the gold coordination plane of (C_i_C_t_AuN=−105.5°) in order to minimize steric repulsions. The Au⋅⋅⋅N distance is short, 2.267 Å (vs. 2.194 Å in **2**), indicating strong N→Au interaction (it is found as a donor‐acceptor interaction by NBO analysis and the respective delocalization energy ΔE(2)=51.6 kcal/mol). The formation of the π‐complex **3** is exergonic by 4.5 kcal/mol. The corresponding *O*‐adduct **3^O^** lies 5.3 kcal/mol lower in energy, in line with the hard, oxophilic character of Au^III^.^[13]^ However, the formation of **3^O^** is probably an unproductive path. Indeed, from **3^O^**, no transition state (TS) could be located for the insertion of the alkene into the Au−O bond. If the alkenol coordinates via the oxygen atom, it is likely **3^O^** then converts into the π‐complex (**3**) from which the oxy‐arylation reaction proceeds readily.

**Figure 3:**
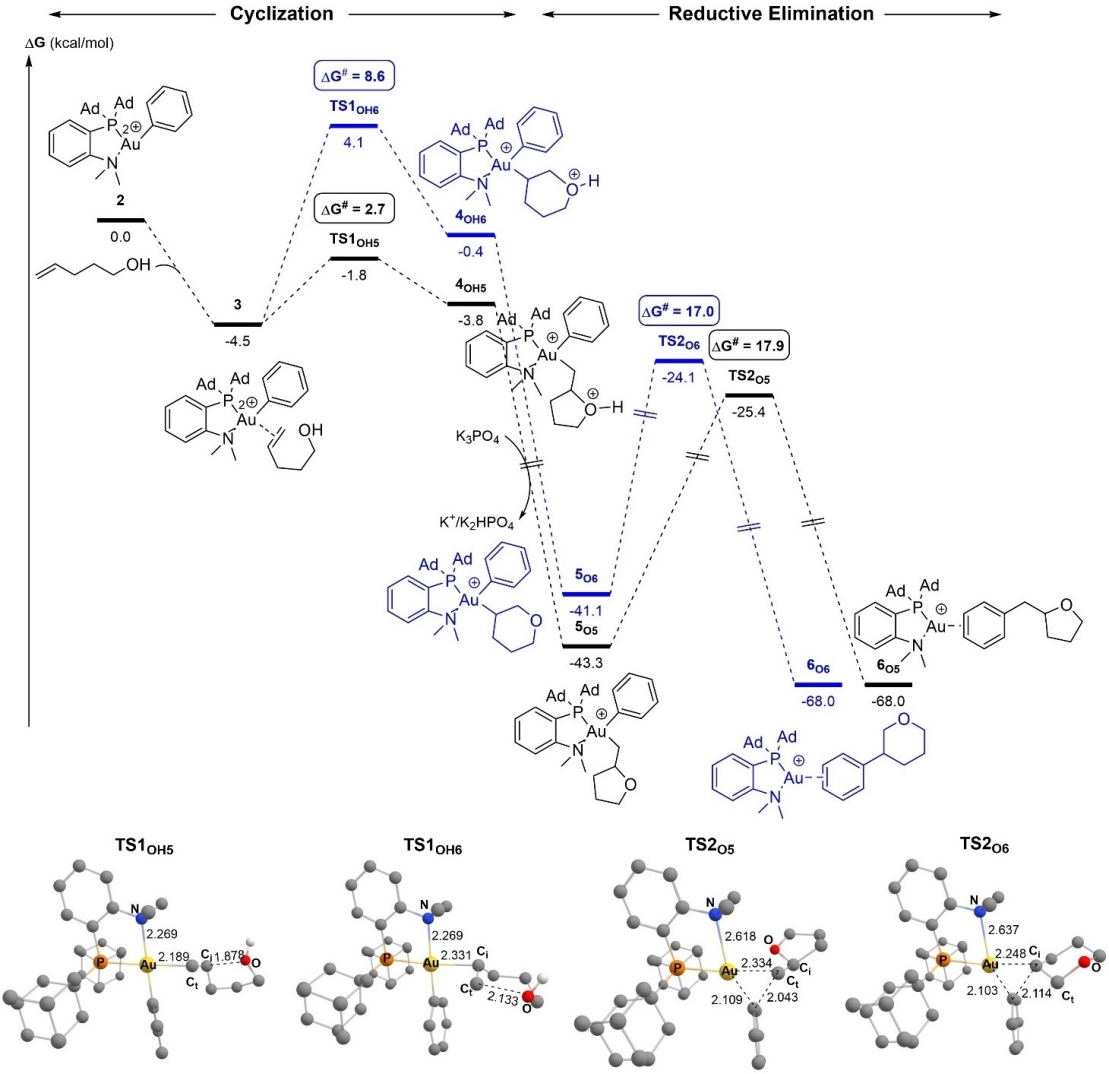
Energy profiles (ΔG in kcal/mol) for the 5‐*exo* (black)/6‐*endo* (blue) oxy‐phenylation of 4‐penten‐1‐ol by the (P,N)Au(Ph)^2+^ gold complex **2**. Calculations performed at the SMD(CH_2_Cl_2_)‐B3PW91‐D3(BJ)/SDD+f(Au), 6‐31+G**(other atoms)//B3PW91/SDD+f(Au), 6‐31G** (other atoms) level of theory in the presence of K_3_PO_4_ (K_3_PO_4_, K_2_HPO_4_ and K^+^ are included in all steps to ensure correct energy balance). Structures of the TSs with main distances in Å.

The energy profiles for the 5‐*exo* and 6‐*endo* oxy‐phenylation of 4‐penten‐1‐ol were both computed. They are depicted in Figure 3. From the π‐complex **3**, nucleophilic addition of the pendant alcohol can occur either on the C_i_ atom (internal carbon atom of the C=C double bond linked to the alkyl chain) or on the C_t_ atom (terminal CH_2_).^[14]^ The respective transition states (**TS1_OH5_** and **TS1_OH6_**) then evolve into the O‐protonated intermediates **4_OH5_** and **4_OH6_** (Figure 3). The activation barrier for the 5‐*exo* cyclization is very low (**TS1_OH5_**, ΔG^≠^=2.7 kcal/mol). It is significantly smaller than that of the 6‐*endo* cyclization (**TS1_OH6_**, ΔG^≠^=8.6 kcal/mol), in line with the full 5‐*exo* regioselectivity observed experimentally. Of note, the formation of the C_i_−O bond is slightly more advanced in **TS1_OH5_** (1.878 Å vs. 1.639 Å for **4_OH5_**) than that of the C_t_−O bond in **TS1_OH6_** (2.043 Å vs. 1.568 Å for **4_OH6_**).

The cyclized intermediates **4_OH_** then readily react with K_3_PO_4_ to give the monocationic complexes **5_O_** along with K^+^ and K_2_HPO_4_.^[15]^ This deprotonation step is highly exergonic (ΔG<−40 kcal/mol), making the formation of **5_O5/6_** irreversible. Reductive elimination finally induces C(sp^2^)−C(sp^3^) coupling and affords the oxy‐phenylation products as π‐adducts of the (P,N)Au^+^ fragment. This step is also highly exergonic, by 24.7 kcal/mol from **5_O5_** and 26.9 kcal/mol from **5_O6_**. The activation barriers for the formation of 2‐benzyltetrahydrofuran and 3‐phenyltetrahydropyran are very similar, 17.9 and 17.0 kcal/mol, respectively. These values fall in the same range than those computed for related C(sp^2^)−C(sp^3^) couplings at gold(III)^[16]^ and are consistent with a reaction proceeding within hours at room temperature (this step is likely rate‐determining). Of note, deprotonation of the cyclized intermediates **4_OH_** by K_3_PO_4_ facilitates the C(sp^2^)−C(sp^3^) coupling step, the activation barriers for the reductive elimination of **4_OH_** were found to be 6.9–7.9 kcal/mol larger than for the deprotonated intermediates **5_O_** (Figure S3).[^12^, ^17^]

The mechanism of C(sp^2^)−C(sp^3^) coupling at gold(III) deserves some comments. It involves concerted cleavage of the Au−Ph/Au−alkyl bonds and formation of the Ph−alkyl bond. The corresponding 3‐center transition state **TS2_O5_** is depicted in Figure 3. The Au⋅⋅⋅N distance noticeably increases (2.618 vs. 2.279 Å in **4_O5_**), which somewhat reduces the coordination number at gold and thereby facilitates the reductive elimination, in line with that previously reported with simple phosphine ligands.^[18]^

Thus, the energy profiles computed for the reaction of 4‐penten‐1‐ol with the (P,N)Au(Ph)^2+^ complex **2** are consistent with the facile and selective formation of 2‐benzyltetrahydrofuran. Cyclization of the π‐complex **3** by outer‐sphere nucleophilic attack of the oxygen atom is the regio‐discriminating step. Deprotonation by K_3_PO_4_ is assumed to occur after this cyclization, while the rate‐determining step is most likely the final reductive elimination leading to C(sp^2^)−C(sp^3^) coupling.

### Oxy‐arylation of (*Z*) and (*E*) 4‐hexen‐1‐ols (internal alkenols)

**Scheme 4:**
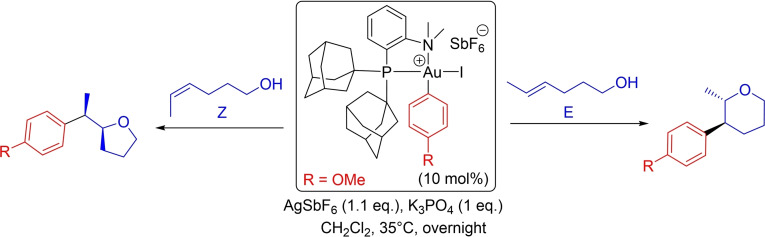
Switch of regioselectivity observed upon catalytic oxy‐arylation of (*Z*/*E*)‐4‐hexen‐1‐ols.^[2h]^

The reaction of the (P,N)Au(Ph)^2+^ complex **2** with internal alkenols was then investigated. The *E* and *Z* isomers of 4‐hexen‐1‐ol were considered with the aim to analyze the different outcomes we unexpectedly observed with these substrates experimentally. Indeed, if both (*E*) and (*Z*)‐4‐hexen‐1‐ol underwent the gold‐catalyzed oxy‐arylation reaction to give a single product resulting from *trans*‐addition across the C=C bond, a complete switch of regioselectivity was observed.[^2h^, ^19^] The *Z* substrate selectively underwent 5‐*exo* cyclization to produce a tetrahydrofuran derivative, whereas the *E* substrate reacted exclusively via a 6‐*endo* process to give a pyran ring (Scheme 4).

**Figure 4:**
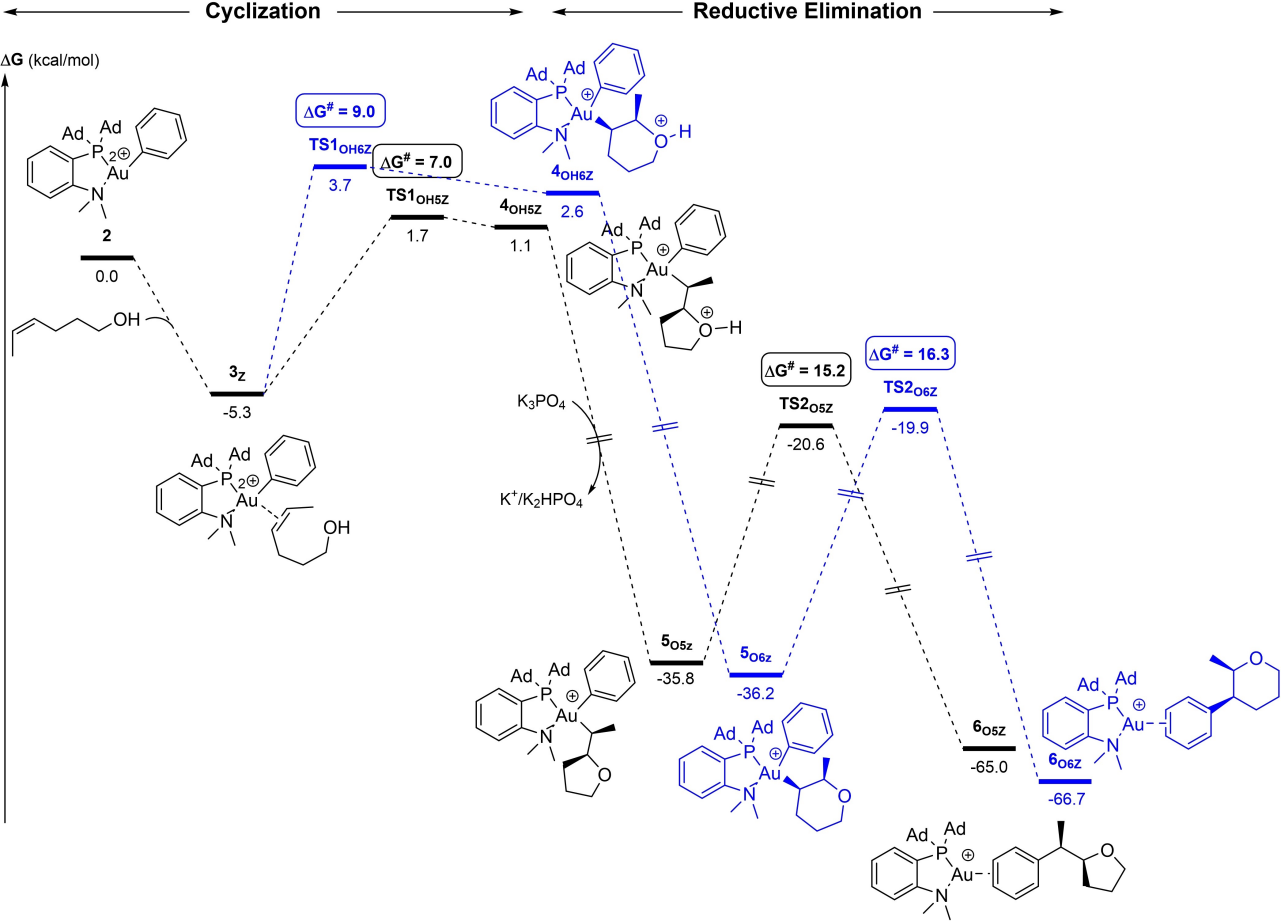
Energy profiles (ΔG in kcal/mol) for the 5‐*exo* (black)/6‐*endo* (blue) oxy‐phenylation of (*Z*)‐4‐hexen‐1‐ol by the (P,N)Au(Ph)^2+^ gold complex **2** computed at the SMD(CH_2_Cl_2_)‐B3PW91‐D3(BJ)/SDD+f(Au), 6‐31+G**(other atoms)//B3PW91/SDD+f(Au), 6‐31G** (other atoms) level of theory in the presence of K_3_PO_4_ (K_3_PO_4_, K_2_HPO_4_ and K^+^ are included in all steps to ensure correct energy balance).

First, we explored the oxy‐phenylation of (*Z*)‐4‐hexen‐1‐ol, where only the 5‐*exo* product was obtained, as for 4‐penten‐1‐ol. The energy profile is depicted in Figure 4 with the π‐complex **3**_***Z***_ as common gateway for the attack of the O atom to the two carbon atoms of the C=C double bond, C_i_ and C_Me_. As for the internal alkenol, π‐coordination of (*Z*)‐4‐hexen‐1‐ol to gold is slightly exergonic (by 5.3 kcal/mol in this case). The largest impact of the additional methyl group is found in the 5‐*exo* cyclization (**TS1_OH5Z_**). Its activation barrier increases by 4.3 kcal/mol at ΔG^≠^=7.0 kcal/mol (vs. 2.7 kcal/mol for 4‐penten‐1‐ol). This increase is tentatively attributed to steric factors as it is this carbon atom (C_Me_) that receives the bulky (P,N)Au(Ph) fragment. Comparatively, the activation barrier for the 6‐*endo* cyclization (**TS1_OH6Z_**) is only marginally affected at ΔG^≠^=9.0 kcal/mol (vs. 8.6 kcal/mol for 4‐penten‐1‐ol). In the end, the transition state **TS1_OH5Z_** remains lower in energy than **TS1_OH6Z_**, in agreement with the 5‐*exo* regioselectivity, but the difference between the 5‐*exo* and 6‐*endo* paths is only 2.0 kcal/mol (instead of 5.9 kcal/mol for 4‐penten‐1‐ol).

Once again, the two cyclized intermediates **4_OH5Z_** and **4_OH6Z_** are readily deprotonated by K_3_PO_4_, making the cyclization process overall strongly exergonic and thus irreversible (the transformation of the π‐complex **3**_***Z***_ into the monocationic complexes **5_O5Z_** and **5_O6Z_** is thermodynamically downhill in energy by >30  kcal/mol). The reductive elimination step is minimally affected by the methyl substituent. From **5_O5Z_** and **5_O6Z_**, C(sp^2^)−C(sp^3^) coupling involves activation barriers of 15.2–16.3 kcal/mol (see Figure S5 for the calculations from the protonated intermediates)^[12]^ and it is strongly exergonic, by 29.2–30.5 kcal/mol.

**Figure 5:**
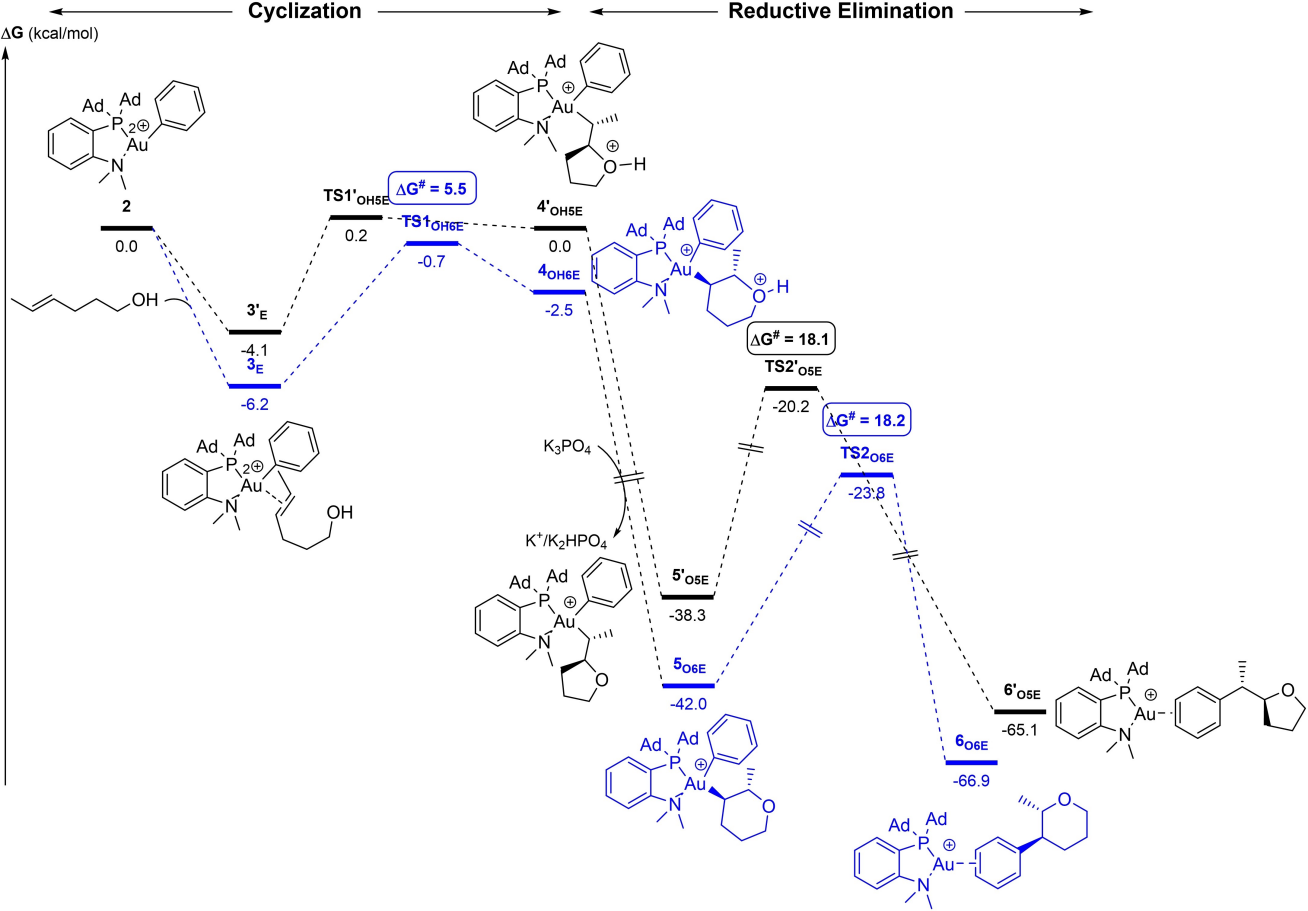
Energy profiles (ΔG in kcal/mol) for the 5‐*exo* (black)/6‐*endo* (blue) oxy‐phenylation of (*E*)‐4‐hexen‐1‐ol by the (P,N)Au(Ph)^2+^ gold complex **2** computed at the SMD(CH_2_Cl_2_)‐B3PW91‐D3(BJ)/SDD+f(Au), 6‐31+G**(other atoms)//B3PW91/SDD+f(Au), 6‐31G** (other atoms) level of theory in the presence of K_3_PO_4_ (K_3_PO_4_, K_2_HPO_4_ and K^+^ are included in all steps to ensure correct energy balance).

The reaction with the (*E*)‐4‐hexen‐1‐ol was also considered. Here, significant differences were noticed compared to (*Z*)‐4‐hexen‐1‐ol and 4‐penten‐1‐ol, in particular in the cyclization step (Figure 5). The most favorable paths for the nucleophilic attack of the oxygen atom to C_i_ and C_Me_ derive from distinct π complexes **3**_***E***_ and **3’**_***E***_, which differ in the way the C=C bond coordinates to gold. **3**_***E***_ is connected to the 6‐*endo* cyclization transition state **TS1_OH6E_** and **3’**_***E***_ to the 5‐*exo* one, **TS1’_OH5E_**.^[20]^ **3’**_***E***_ is slightly higher in energy than **3**_***E***_ (by 2.1 kcal/mol), and **TS1_OH6E_** lies 0.9 kcal/mol below **TS1’_OH5E_** in this case, in line with the 6‐*endo* selectivity observed experimentally. Of note, the activation barrier for the 6‐*endo* cyclization of (*E*)‐4‐hexen‐1‐ol (5.5 kcal/mol) is significantly lower than those found for 4‐penten‐1‐ol and (*Z*)‐4‐hexen‐1‐ol (8.6 and 9.0 kcal/mol, respectively), and consistently, the transition state **TS1_OH6E_** is earlier (the distance of the forming C⋅⋅⋅O bond is 2.335 Å, vs. 2.012–2.043 Å for the terminal and *Z*‐alkenols, respectively). The reductive elimination (C(sp^2^)−C(sp^3^) coupling) remains the rate‐determining step, with an activation barrier ΔG^≠^ of ∼18 kcal/mol for both the tetrahydropyran and tetrahydrofuran products, corroborating the weak impact of the substitution of the double bond of the alkenol by Me on this step.

Thus, the energy profiles computed for the reaction of the (P,N)Au(Ph)^2+^ complex **2** with 4‐penten‐1‐ol, (*Z*)‐4‐hexen‐1‐ol and (*E*)‐4‐hexen‐1‐ol nicely parallel the experimental results. The π‐complexes deriving from the coordination of the C=C bond to gold(III) are key intermediates. From there, outer‐sphere nucleophilic attack of the oxygen atom is governing the regioselectivity. Considering the different paths and locating the corresponding transition states, 5‐*exo* cyclization was found to be indeed favored for the terminal and *Z*‐alkenols, while the *E*‐alkenol undergoes preferentially 6‐*endo* cyclization.

**Figure 6:**
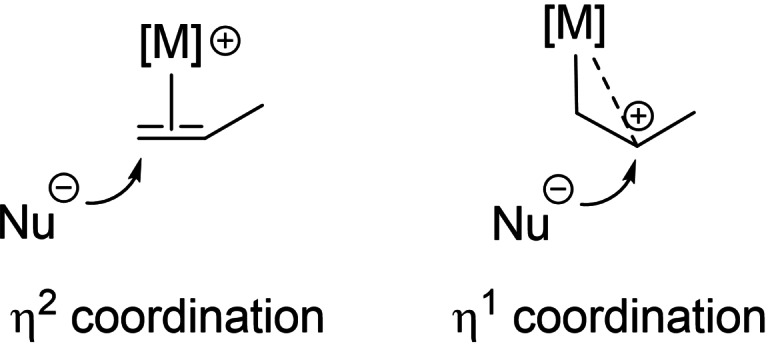
Schematic representation of nucleophilic additions to alkenes promoted by transition metals and the influence η^2^/η^1^ coordination may have on regioselectivity.

Previous computational studies have shown that π‐complexes play a key role in nucleophilic additions to π‐CC bonds promoted by transition metals. It was pointed out early on that the preferred site of nucleophilic attack may be related to the coordination mode of the π‐CC bonds (η^2^ to η^1^ slippage), potentially enabling to predict regioselectivity (Figure 6).^[21]^

For example, G. Ujaque, A Lledos and I. Fernández recently studied the origin of *anti*‐Markovnikov regioselectivity in the hydroamination of alkenes catalyzed by Rh(I) and Au(I) complexes.[^21c^, ^21d^] In both cases, the structure of the reactive π‐complex was found to control regioselectivity. The relative contribution of the terminal and internal C atoms in the LUMO was found to be critical, not their atomic charges. Zhang et al. also emphasized the role of π‐complexes in the 1,2‐diarylation of alkenes catalyzed by the (P N)Au complex, but here, regioselectivity was proposed to result rather from charge control and electrostatic interactions.^[8]^

### Structure of the gold(III) π‐complexes

**Figure 7:**
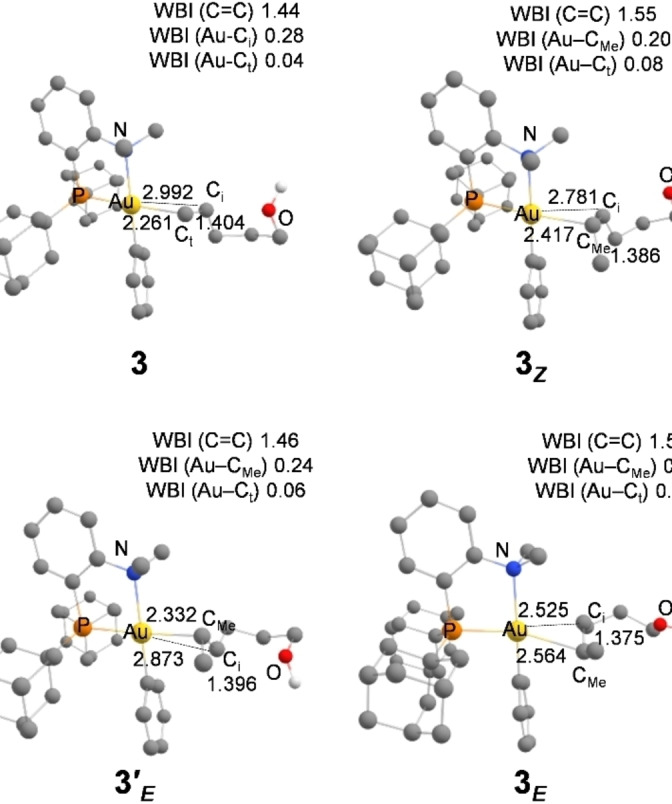
Main geometrical parameters (distances in Å) for the π‐complexes **3**, **3_Z_**, **3_E_** and **3’_E_** involved in the cyclization of alkenols promoted by the (P,N)Au(Ph)^2+^ gold complex **2**, computed at the B3PW91/SDD+f(Au), 6‐31G** (other atoms) level of theory. Wiberg bond indexes (WBI) for the C=C and Au−C bonds.

Given these precedents, the structure of the π‐complexes involved in the cyclization of 4‐penten‐1‐ol, (*Z*)‐4‐hexen‐1‐ol and (*E*)‐4‐hexen‐1‐ol promoted by the (P,N)Au(Ph)^2+^ gold(III) complex **2** were analyzed in‐depth and compared. Their optimized geometries are displayed in Figure 7.^[22]^ In the π‐complex **3**, the AuC_t_ distance (2.261 Å) is much shorter than the AuC_i_ distance (2.992 Å, Δ(AuC) 0.73 Å) indicating strong slippage of the C=C double bond towards η^1^‐type coordination. The (*Z*) internal alkenol also binds to complex **2** in a highly dissymmetric manner although the difference between the AuC distances is not as large in **3_Z_** (AuC_Me_ 2.417 Å, AuC_i_ 2.781 Å, Δ(AuC) 0.36 Å). Remarkably, the two π‐complexes deriving from (*E*)‐4‐hexen‐1‐ol are close in energy (ΔG=2.1 kcal/mol) but adopt quite different coordination modes. In **3’**_***E***_, the C=C double bond is dissymmetrically coordinated to gold. The AuC_Me_ distance (2.332 Å) is significantly shorter than the AuC_i_ distance (2.872 Å), the difference between the two (Δ(Au−C) 0.54 Å) being at halfway of those found in **3** and **3**_***Z***_. In stark contrast, the π‐complex **3**_***E***_ adopts a quasi symmetric η^2^‐type structure, with AuC_Me_ and AuC_i_ distances of 2.564 and 2.525 Å, respectively (Δ(AuC) 0.039 Å). The η^1^/ η^2^ slippage of the C=C bond at gold from complexes **3**, **3’**_***E***_ and **3**_***Z***_, to **3**_***E***_ is also apparent from the Wiberg Bond Indexes (WBI) of the C=C and Au−C bonds (Figure 7).

**Figure 8:**
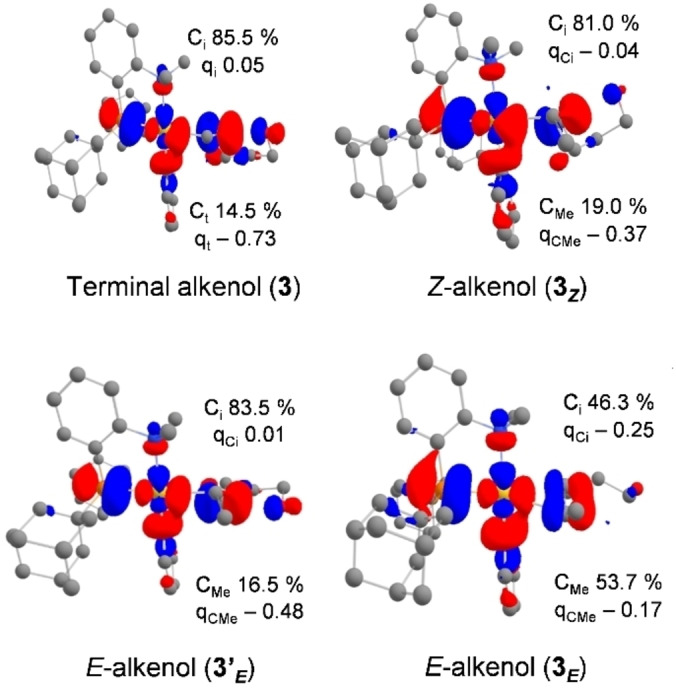
Plot of the LUMO (cutoff: 0.04) for the π‐complexes **3**, **3’_E_** and **3_Z/E_** involved in the cyclization of alkenols promoted by the (P,N)Au(Ph)^2+^ gold complex **2**. Relative participation of the C_t/Me_ and C_i_ atoms (in %) in the π_C=C_ orbital. NPA charges (q) of the C_t/Me_ and C_i_ atoms.

The electronic structure of the π‐complexes was also thoroughly analyzed. The relevant orbital for the outer‐sphere nucleophilic attack of the oxygen atom to the π‐activated C=C bond is the LUMO. It is mainly associated with the interaction of the π_C=C_ orbital of the alkenol with the σ*_AuP_ orbital of the gold fragment (Figures 8, S8 and S9).^[12]^ The contributions of the two carbon atoms of the C=C double bond (on which the cyclization may occur) were assessed by an orbital composition analysis (their relative weight is expressed in %). Accordingly, the LUMO was found to be strongly polarized towards the internal carbon atom C_i_ involved in 5‐*exo* cyclization for the π‐complexes **3** and **3**_***Z***_ deriving from 4‐penten‐1‐ol (85.5 % C_i_, 14.5 % C_t_) and (*Z*)‐4‐hexen‐1‐ol (81.0 % C_i_, 19.0 % C_Me_), respectively, as well as for the π‐complex **3’**_***E***_ deriving from (*E*)‐4‐hexen‐1‐ol (83.5 % C_i_, 16.5 % C_Me_). In contrast, the two carbon atoms contribute about equally to the LUMO of complex **3**_***E***_ involved in 6‐*endo* cyclization, and the contribution of C_Me_ actually becomes slightly predominant (46.3 % C_i_, 53.7 % C_Me_). As for electrostatic factors, we referred to the NPA charges of the carbon atoms of the C=C double bond (Figures 8 and S9).^[12]^ For the π‐complex **3** deriving from 4‐penten‐1‐ol, the internal carbon atom C_i_ bears a slightly positive charged (0.05) whereas the terminal carbon atom C_t_ is negatively charged (−0.73), in line with the observed 5‐*exo* cyclization upon nucleophilic attack of O to C_i_. For the π‐complexes **3_Z_** and **3’_E_**, the charge of C_i_ remains higher than that of C_Me_, but the difference between the two is reduced (Δq=0.41 and 0.47|e|, respectively vs. 0.78|e| for **3**). Once again, the π‐complex **3_E_** stands out from this picture. The atomic charges of C_i_ and C_Me_ are similar (the difference Δq is of only 0.08|e| in this case), in line with the symmetric η^2^‐type coordination of the C=C double bond. The carbon atom C_Me_ actually bears the highest charge in this case, in line with the observed 6‐*endo* cyclization.

**Figure 9:**
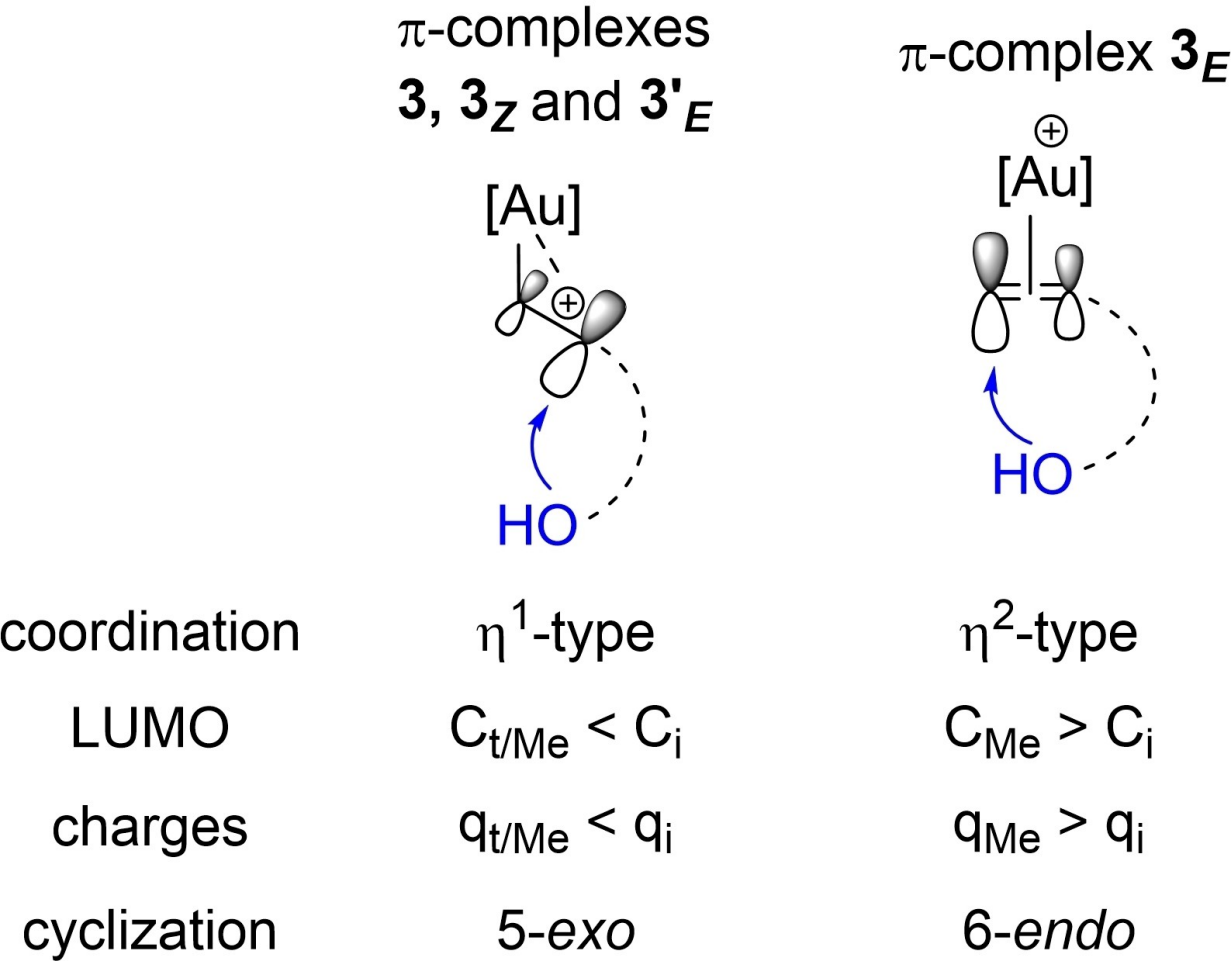
Schematic representation of the 5‐*exo*/6‐*endo* cyclization of alkenols at gold according to the coordination mode and electronic structure of the respective π‐complex (C_t_, C_i_ and C_Me_ refer to the terminal, internal and Me‐substituted carbon atoms of the C=C double bond, respectively).

Overall, these results nicely parallel our experimental observations and suggest that the regioselectivity outcome of the alkenol cyclization is to some extent encoded into the π‐complexes. It is noteworthy that in this case, orbital and charge effects apparently fall in line to drive the nucleophilic attack of O towards 5‐*exo* or 6‐*endo* cyclization (Figure 9). The origin of the switch of regioselectivity observed for the *E*‐internal alkenol is likely the symmetric η^2^‐type coordination of the C=C double bond at gold in **3**_***E***_, resulting in a larger coefficient on C_Me_ atom in the LUMO, and a higher atomic charge at C_Me_ than at C_i_.

If calculations are to be used to predict the preferred cyclization mode, it may be possible to simply refer to the π‐complex when it adopts a single well‐defined coordination mode (η^1^ or η^2^), as for 4‐penten‐1‐ol and (*Z*)‐4‐hexen‐1‐ol. However, when several *minima* close in energy are found for π‐complexes with η^1^ and η^2^‐like structures, as for (*E*)‐4‐hexen‐1‐ol, it is needed to locate the transition states for the 5‐*exo* and 6‐*endo* cyclizations to adequately compare the two paths and analyze the regioselectivity.

### Oxy‐vinylation of terminal and *Z*/*E*‐internal alkenols

**Scheme 5:**
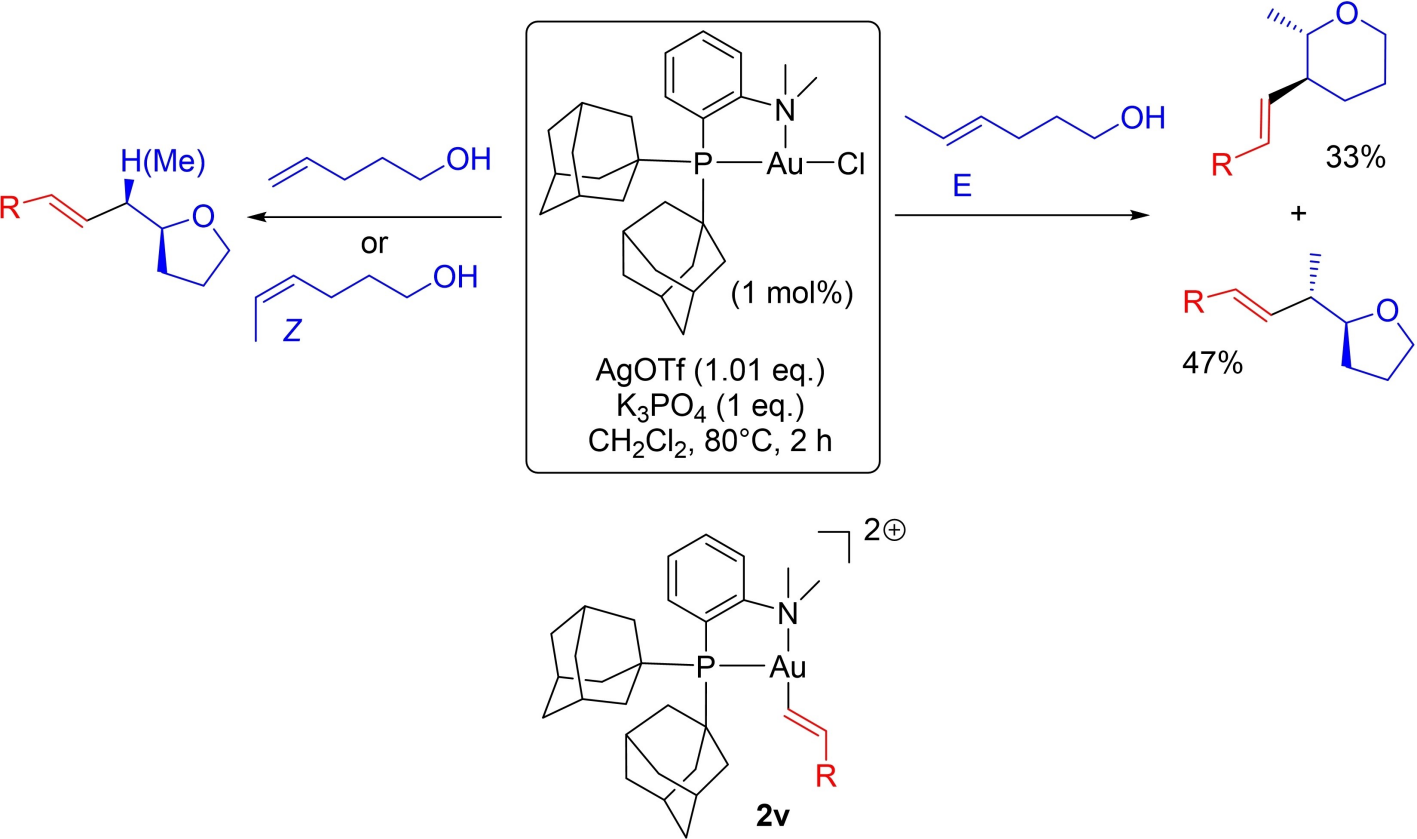
Oxy‐vinylation of alkenols (5‐*exo*/6‐*endo* cyclizations) involving the (P,N))Au(vinyl)^2+^ complex **2 v**.

Besides aryl iodides, the (MeDalphos)AuCl complex was shown to readily activate vinyl iodides and to efficiently catalyze the oxy‐vinylation of alkenols.^[2h]^ This transformation was also investigated by DFT with the aim to further assess the impact of the alkenol coordination on regioselectivity. Experimentally, 4‐penten‐1‐ol and (*Z*)‐4‐hexen‐1‐ol were found to exclusively undergo 5‐*exo* cyclization, while we obtained a mixture of tetrahydropyran/tetrahydrofuran products from (*E*)‐4‐hexen‐1‐ol due to competitive 5‐*exo* and 6‐*endo* cyclizations (Scheme 5).

Based on our previous study of the oxy‐phenylation reaction, we focused our computational investigations on the (P,N)Au(vinyl)^2+^ complex **2 v** as the active species (with the vinyl group *trans* to N) and special attention was given to the corresponding π‐complexes deriving from the coordination of the alkenols (terminal and *Z*/*E*‐internal) *trans* to phosphorus (Scheme 5).

Replacing the phenyl at gold for a vinyl group reduces symmetry. Upon coordination of the alkenol to gold, the (CH_2_)_3_OH chain may point in the same direction or opposite to the vinyl group at gold. Both orientations have been considered (Figure S10),^[12]^ but for sake of clarity, only the π‐complex **3 v** which is slightly more stable than the other π‐complex **3v^B^** (by 0.6 kcal/mol) will be discussed here. In **3 v**, the (CH_2_)_3_OH chain of the alkenol and the vinyl group at gold are oriented up and down with respect to the coordination plane of gold. Its formation is exergonic by 3.7 kcal/mol (vs. 4.5 kcal/mol for the corresponding Au(Ph) complex **3**).

**Figure 10:**
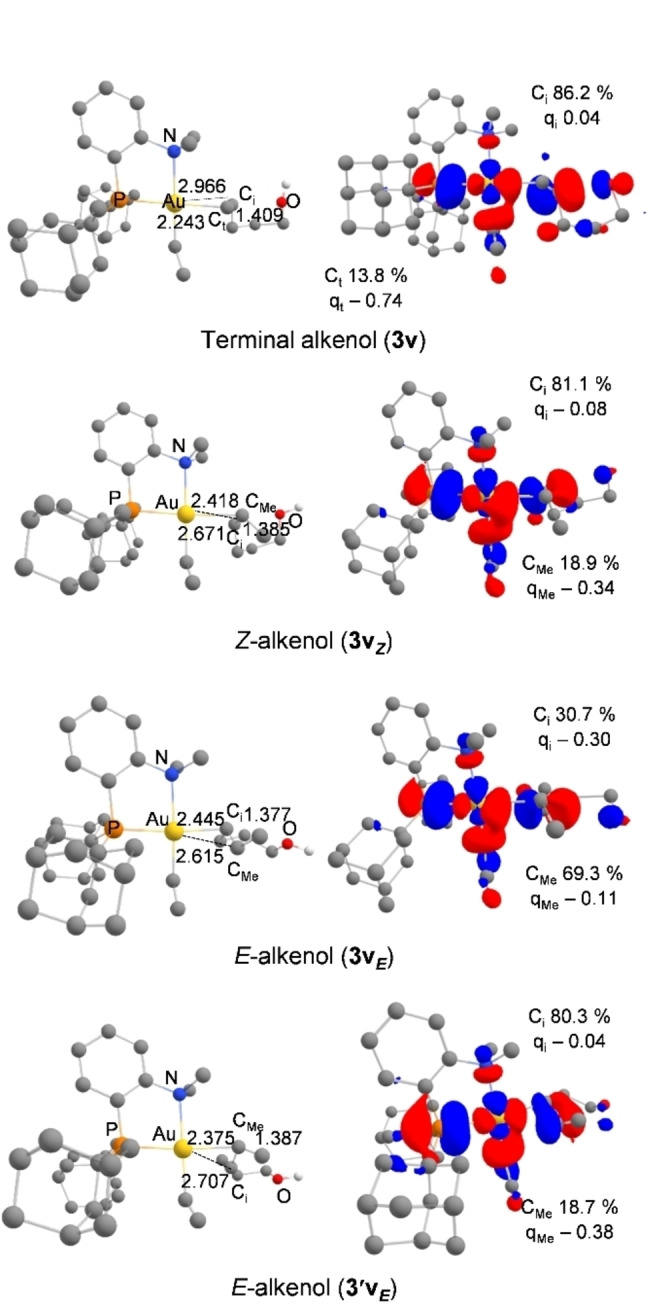
Main geometrical parameters (distances in Å, left) and plot of the LUMO (cutoff: 0.04, right) for the π‐complexes **3 v**, **3v_Z_**, **3v_E_** and **3’v_E_** associated to the cyclization step of the oxy‐vinylation reaction. Relative participation of the C_t/Me_ and C_i_ atoms (in %) in the LUMO orbital. NPA charges (q) of the C_t/Me_ and C_i_ atoms.

The structure of **3 v** (Figure 10) very much resembles that of **3**. The C=C bond is very dissymmetrically coordinated to gold with AuC_t_ and AuC_i_ distances of 2.243 and 2.966 Å, respectively (Δ(AuC) 0.72 Å). It is strongly polarized as apparent from the LUMO (relative contributions of C_i_ and C_t_ of 86.2 % and 13.8 %, respectively) as well as from the atomic charges (0.04 at C_i_, −0.74 at C_t_) (Figure S12).^[12]^

**Figure 11:**
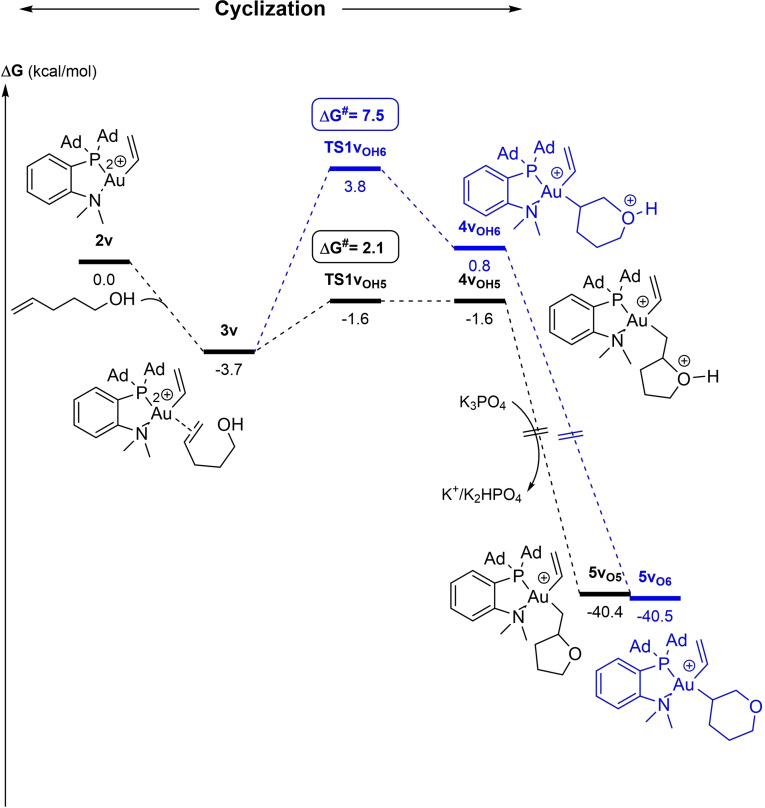
Energy profiles (ΔG in kcal/mol) for the 5‐*exo* (black)/6‐*endo* (blue) oxy‐vinylation of 4‐penten‐1‐ol by the (P,N)Au(vinyl)^2+^ gold complex **2 v** computed at the SMD(CH_2_Cl_2_)‐B3PW91‐D3(BJ)/SDD+f(Au), 6‐31+G**(other atoms)//B3PW91/SDD+f(Au), 6‐31G** (other atoms) level of theory in the presence of K_3_PO_4_ (K_3_PO_4_, K_2_HPO_4_ and K^+^ are included in all steps to ensure correct energy balance).

Given the conclusions drawn from the DFT study of the oxy‐arylation reaction, the structure of **3 v** may be anticipated to favor 5‐*exo* over 6‐*endo* cyclization, in line with experimental observations. This assumption was confirmed by computing the reaction profile for the oxy‐vinylation (Figure 11). Most relevant are the transition states for the nucleophilic attack of oxygen from the π‐complex **3 v**. The activation barrier for the addition to the internal carbon atom C_i_ (**TS1v_OH5_**) of the π‐complex **3 v** was found to be significantly lower than for the addition to the terminal carbon atom C_t_ (**TS1v_OH6_**) (Δ(ΔG^≠^)=5.4 kcal/mol). 5‐*Exo* cyclization via **TS1v_OH5_** is thus clearly favored over 6‐*endo* cyclization, and subsequent deprotonation with K_3_PO_4_ drives the reaction forward.^[23]^

**Figure 12:**
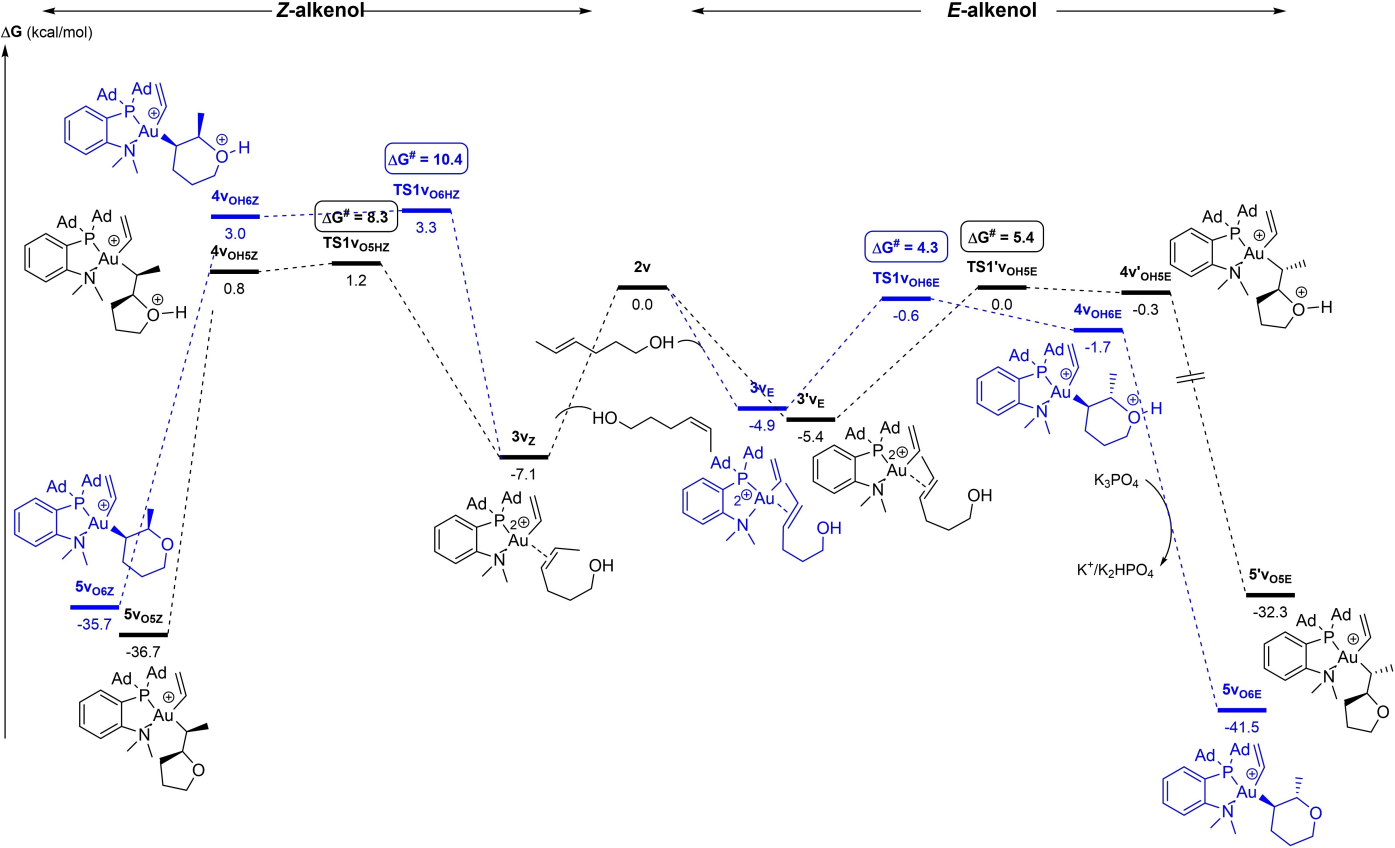
Energy profiles (ΔG in kcal/mol) for the 5‐*exo* (black)/6‐*endo* (blue) oxy‐vinylation of (*Z*)‐4‐hexen‐1‐ol (left) and (*E*)‐4‐hexen‐1‐ol (right) by the (P,N)Au(vinyl)^2+^ gold complex **2v** computed at the SMD(CH_2_Cl_2_)‐B3PW91‐D3(BJ)/SDD+f(Au), 6‐31+G**(other atoms)//B3PW91/SDD+f(Au), 6‐31G** (other atoms) level of theory in the presence of K_3_PO_4_ (K_3_PO_4_, K_2_HPO4 and K^+^ are included in all steps to ensure correct energy balance).

The oxy‐vinylation of (*Z*)‐4‐hexen‐1‐ol was then considered (Figure 12). The corresponding vinyl π‐complex **3v**_***Z***_ adopts a dissymmetric polarized structure. The difference between the AuC_Me_ and AuC_i_ distances (2.418 and 2.671 Å, respectively) is slightly less than in the corresponding π‐complex **3**_***Z***_ (Δ(AuC) 0.25 vs. 0.35), but the contribution of C_Me_ in the LUMO remains very important in **3v**_***Z***_ (>80 %) and the charge of C_i_ is again significantly higher than that of C_Me_ (Δq=0.26 |e|), consistent with 5‐*exo* regioselectivity upon cyclization. The reaction profile for the cyclization step (Figure 12, left) falls in line with the structure of the π‐complex **3v**_***Z***_. As for the corresponding oxy‐phenylation reaction, the activation barrier for the 5‐*exo* cyclization of (*Z*)‐4‐hexen‐1‐ol is higher than for 4‐penten‐1‐ol (8.3 vs. 2.1 kcal/mol), but it remains smaller than that required for the 6‐*endo* cyclization (by 2.1 kcal/mol).

Finally, we examined the oxy‐vinylation of (*E*)‐4‐hexen‐1‐ol and found again two *minima* on the potential energy surface of the π‐complex, namely **3v**_***E***_ and **3’v**_***E***_ (Figure 12). They adopt quite different structures. In **3’v**_***E***_, the C=C double bond slips towards η^1^ coordination with AuC_Me_ and AuC_i_ distances of 2.375 and 2.707 Å, respectively (Δ(AuC) 0.33 Å). It is polarized as indicated by the relative contributions of C_i_ and C_Me_ in the LUMO (80.3 % and 19.7 %, respectively), and the atomic charges (−0.04 at C_i_, −0.38 at C_Me_, Δq=0.34|e|). In contrast, **3v**_***E***_ adopts a more symmetric η^2^‐type structure, with AuC_Me_ and AuC_i_ distances of 2.445 and 2.615 Å, respectively (Δ(AuC) 0.17 Å). Here, the contribution of C_Me_ in the LUMO is predominant (C_Me_ 69.3 %, C_i_ 30.7 %) and the charge is higher at C_Me_ than C_i_ (Δq=0.19|e|). The two π‐complexes are quasi isoenergetic (ΔG=0.5 kcal/mol). To compare the 5‐*exo* and 6‐*endo* routes for cyclization, it is thus needed to locate the corresponding transition states (see Figure 12, right). As expected from the structure of the respective π‐complexes, **3’v**_***E***_ readily connects with the 5‐*exo* product (via **TS1’v_OH5*E*_**), while the 6‐*endo* product is easily obtained from **3v**_***E***_ (via **TS1v_OH6*E*_**). The corresponding transition states are low in energy (activation barriers of 4.3 and 5.4 kcal/mol, respectively) and actually very close (ΔG=0.6 kcal/mol), in agreement with the competitive occurrence of 5‐*exo* and 6‐*endo* cyclization observed experimentally.

Overall, the DFT study of the oxy‐vinylation nicely parallels the experimental results and corroborates the conclusions reached for the oxy‐phenylation. The regioselectivity of the reactions is dictated by the cyclization step and here, the π‐complex clearly plays a major role. When one coordination mode (η^1^ or η^2^) is unambiguously favored, it is possible to refer to the geometry and/or electronic structure of the π‐complex to explain and predict the regioselectivity. In this study, this is the case for 4‐penten‐1‐ol and (*Z*)‐4‐hexen‐1‐ol which distinctly form dissymmetric polarized π‐complexes and exclusively undergo 5‐*exo* cyclization. However, when several π‐complexes with η^1^ and η^2^‐like structures are close in energy, as for (*E*)‐4‐hexen‐1‐ol, it is necessary to perform a comprehensive mechanistic study and locate the transition states for the most favorable paths of 5‐*exo*/6‐*endo* cyclizations in order to describe accurately the reaction and come to reliable conclusions.

## Conclusion

This comprehensive DFT study provides mechanistic insights into the intramolecular oxy‐arylation/vinylation of alkenols catalyzed by the (MeDalphos)AuCl complex and unravels the factors dictating its regioselectivity. The way alkenols coordinate to the (P,N)Au(Ph)^2+^ and (P,N)Au(vinyl)^2+^ complexes (η^1^ or η^2^) plays a major role. With terminal and *Z*‐internal alkenols, the π‐complexes adopt η^1^‐like structures. The occurrence of 6‐*endo* cyclization with *E*‐internal alkenols likely finds its origin in competitive η^2^‐type coordination of the C=C double bond at gold. Here, orbital and charge effects fall in line to drive the nucleophilic attack of the pendant oxygen atom towards 5‐*exo* or 6‐*endo* cyclization. In the most favorable cases, referring to the π‐complexes may be enough to predict the preferred path of cyclization (5‐*exo* vs. 6‐*endo*).

Such theoretical studies shed light into the structure and reactivity of key intermediates within ligand‐enabled Au(I)/Au(III) catalytic cycles. This work also advances our understanding on the factors controlling the addition of nucleophiles to π‐substrates at transition metals, an elementary step involved in many catalytic transformations.

## Computational Details

All optimizations were performed using the Gaussian 16 package^[24]^ and the B3PW91 hybrid functional^[25]^ on the real systems, without taking into account the counter‐anion. All stationary points involved were fully optimized in gas phase. The gold atom was described with the relativistic electron core potential SDD and associated basis set, augmented by a set of f‐orbital polarization functions.^[26]^ The 6‐31G** basis set was employed for all other atoms. Frequency calculations were undertaken to confirm the nature of the stationary points, yielding one imaginary frequency for transition states (TS), corresponding to the expected process, and all of them positive for *minima*. The connectivity of the transition states and their adjacent *minima* was confirmed by intrinsic reaction coordinate (IRC) calculations.^[27]^

For better accuracy of the Gibbs free energies, the energy profiles were computed at the SMD(CH_2_Cl_2_)‐B3PW91‐D3(BJ)/SDD+f(Au), 6‐31+G**(other atoms)//B3PW91/SDD+f(Au), 6‐31G** (other atoms) level of theory. Dispersion effect has been included with D3 correction of Grimme with Becke–Johnson damping (DFT‐D3(BJ))^[28]^ and solvent effects were considered (Dichloromethane: CH_2_Cl_2_) by means of the universal Solvation Model based on solute electron Density (SMD).^[29]^

The geometrical structures and Molecular Orbitals (LUMO) were plotted with the Chemcraft 1.8^[30]^ program. For the key unoccupied molecular orbital (LUMO) of the π‐complexes, the atomic orbital composition of each MO (%) was computed thanks to Multiwfn 3.6 package.^[31]^ For the π‐complexes, Natural Bond Orbital analyses (NBO)^[32]^ were performed with NBO, 5.9 version^[33]^ in order to determine the NPA charges of the carbon atoms of the C=C double bond and the Wiberg bond indexes of the Au−C and C=C bonds. Second Order Perturbation Theory analyses were also performed to assess the strength of N→Au interactions in some cases (stabilizing interaction ΔE(2) in kcal/mol).

## Conflict of interest

The authors declare no conflict of interest.

1

## Supporting information

As a service to our authors and readers, this journal provides supporting information supplied by the authors. Such materials are peer reviewed and may be re‐organized for online delivery, but are not copy‐edited or typeset. Technical support issues arising from supporting information (other than missing files) should be addressed to the authors.

Supporting Information

## Data Availability

The data that support the findings of this study are available in the supplementary material of this article.
