# Supplementary material for: Mechanistic Insights about the Ligand‐Enabled Oxy‐arylation/vinylation of Alkenes via Au(I)/Au(III) Catalysis
Source: Chemistry. 2022 Aug 25;28(60):e202202110. doi: 10.1002/chem.202202110 (PMC9805180; doi:10.1002/chem.202202110)
Supplement: Supplementary file 1 — Supporting Information [file CHEM-28-0-s001.pdf]

Chemistry–A European Journal

Supporting Information

Mechanistic Insights about the Ligand-Enabled Oxy-arylation/vinylation of Alkenes via Au(I)/Au(III) Catalysis

Mathilde Rigoulet, Karinne Miqueu,* and Didier Bourissou*

Supporting Information

Figure S1. Geometrical structures (distances in Å and bond angles in °) of the two isomers **2** and **2'** of the (P,N)Au^{2+}-Ph complex computed at B3PW91/SDD+f(Au), 6-31G** (other atoms) level of theory. Relative stability (ΔG) in kcal/mol calculated at SMD(DCM)-B3PW91-D3(BJ)/SDD+f(Au),6-31+G**//B3PW91/SDD +f(Au), 6-31G** (other atoms) level.

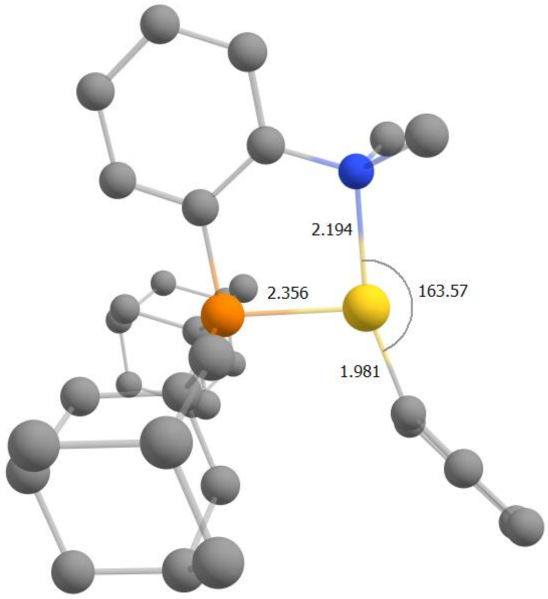

2

ΔG = 0 kcal/mol

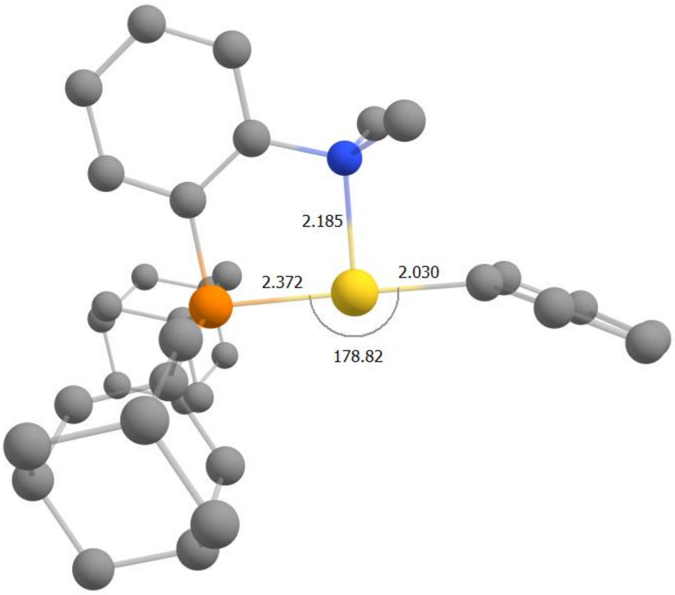

2'

This isomer has been located by imposing the $PAuC_{Ar}$ angle at 178.82°.

ΔG = 29.6 kcal/mol

Figure S2. Geometrical structures (distances in Å and bond angles in °) of the isomers associated to coordination of 4-penten-1-ol to (P,N)Au^{2+}-Ph complex **2**: π-coordination (**3**) or O→Au interaction (**3'**), computed at B3PW91/SDD+f(Au), 6-31G** (other atoms) level of theory. Relative stability (ΔG) in kcal/mol calculated at SMD(DCM)-B3PW91-D3(BJ)/SDD+f(Au),6-31+G**//B3PW91/SDD+f(Au), 6-31G** (other atoms) level.

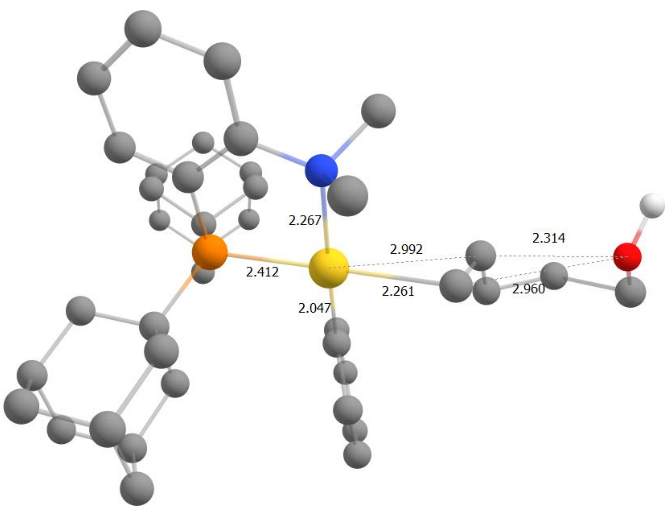

3
ΔG = 0 kcal/mol

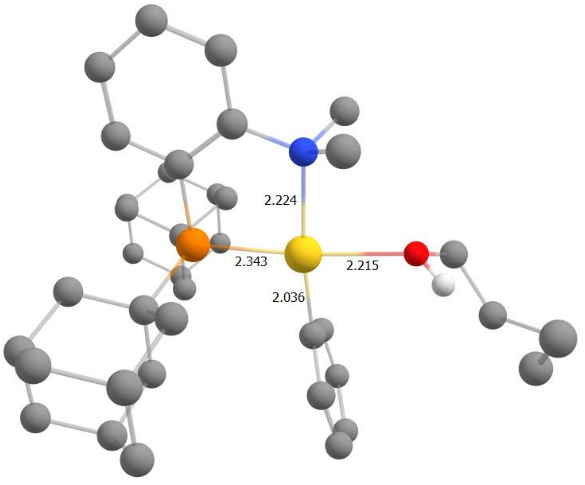

3'
ΔG = -5.3 kcal/mol

Figure S3. Energy profiles (ΔG in kcal/mol) for the oxy-arylation reaction involving (P,N)Au^{2+}-Ph gold complex **2** and 4-penten-1-ol computed at SMD(DCM)-B3PW91-D3(BJ)/SDD+f(Au), 6-31+G**(other atoms)//B3PW91/SDD+f(Au), 6-31G** (other atoms) level of theory. Direct reductive elimination from $\mathbf{4_{OH}}$. K_3PO_4 comes in after reductive elimination step. Formation of 5-*exo* and 6-*endo* products.

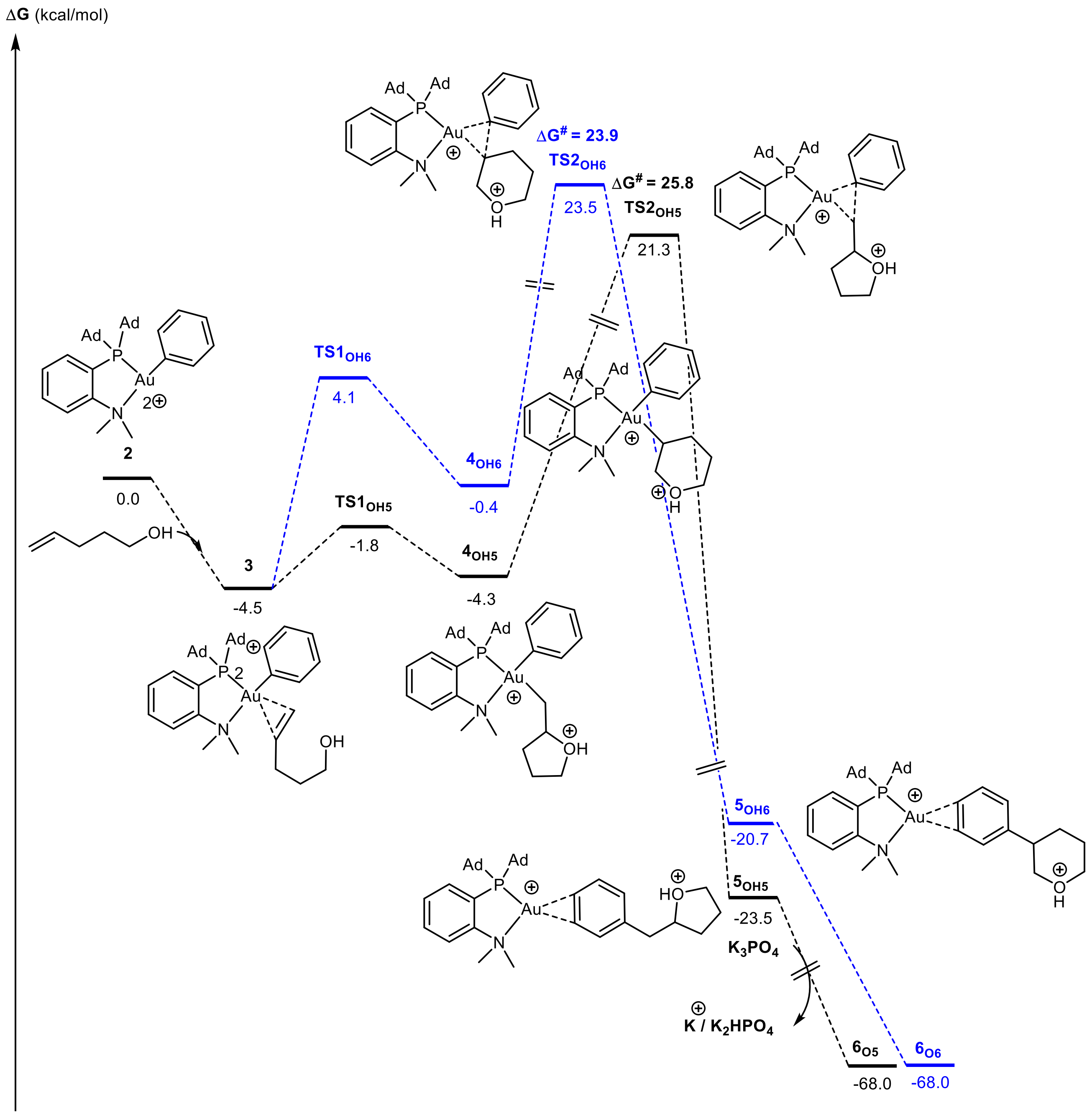

Direct reductive elimination from $\mathbf{4_{OH}}$ was found more energetically demanding ($ΔG^{\#}$ = 23.9-25.8 kcal/mol) than from non-protonated intermediates $\mathbf{5_{O}}$ ($ΔG^{\#}$ = 17.0-17.9 kcal/mol). Thus, K_3PO_4 facilitates the reductive elimination step through the formation of the non-protonated intermediate $\mathbf{5_{O}}$.

Figure S4. Energy profiles (ΔG in kcal/mol) of the cyclization step for the oxy-arylation reaction involving (P,N)Au^{2+}-Ph gold complex **2** and 4-pent-1-enolate computed at SMD(DCM)-B3PW91-D3(BJ)/SDD+f(Au), 6-31+G**(other atoms)//B3PW91/SDD+f(Au), 6-31G** (other atoms) level of theory. Formation of 5-*exo* product.

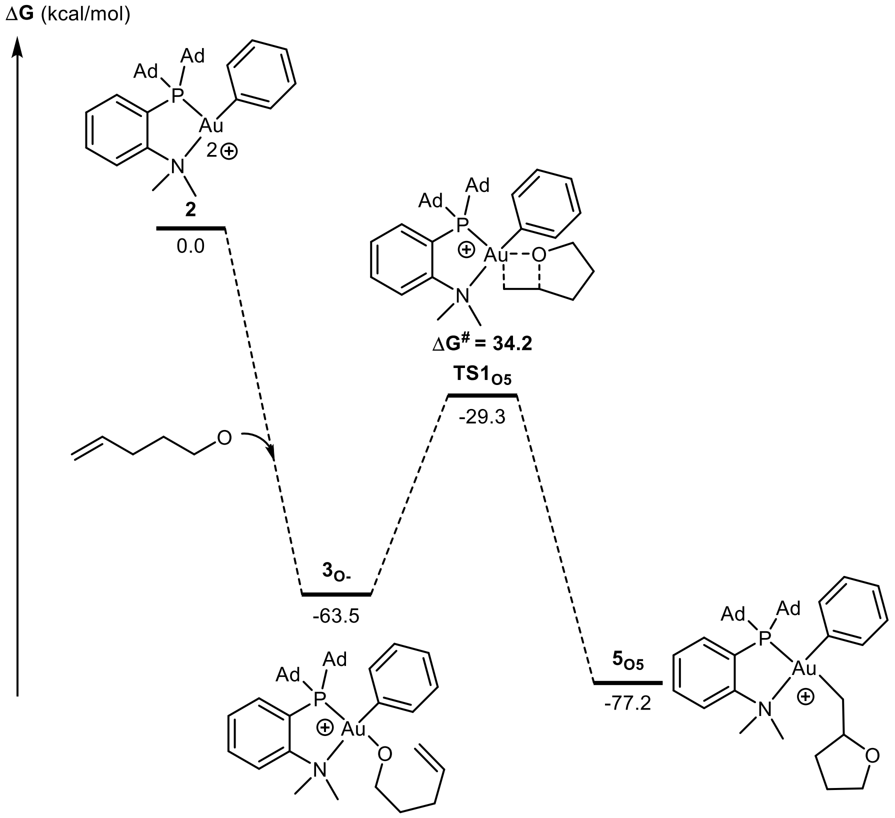

Reaction between 4-pent-1-enolate (abstraction of H^+ by K_3PO_4 on alcohol) and complex **2** has been studied. O-coordination occurs and is thermodynamically favoured. Cyclization step proceeds with a high activation barrier of $\Delta G^{\#}$ = 34.2 kcal/mol to give $\mathbf{5_{O5}}$ (exergonic step, ΔG = -13.7 kcal/mol). For a kinetic viewpoint, this cyclization is unfeasible under the reaction conditions and cannot compete with cyclization involving alcohol.

Figure S5. Energy profiles (ΔG in kcal/mol) for the reductive elimination step of the oxy-arylation reaction involving (P,N)Au^{2+}-Ph gold complex **2** and Z/E-hex-4-enols, computed at SMD(DCM)-B3PW91-D3(BJ)/SDD+f(Au), 6-31+G**(other atoms)//B3PW91/SDD+ f(Au), 6-31G** (other atoms) level of theory.

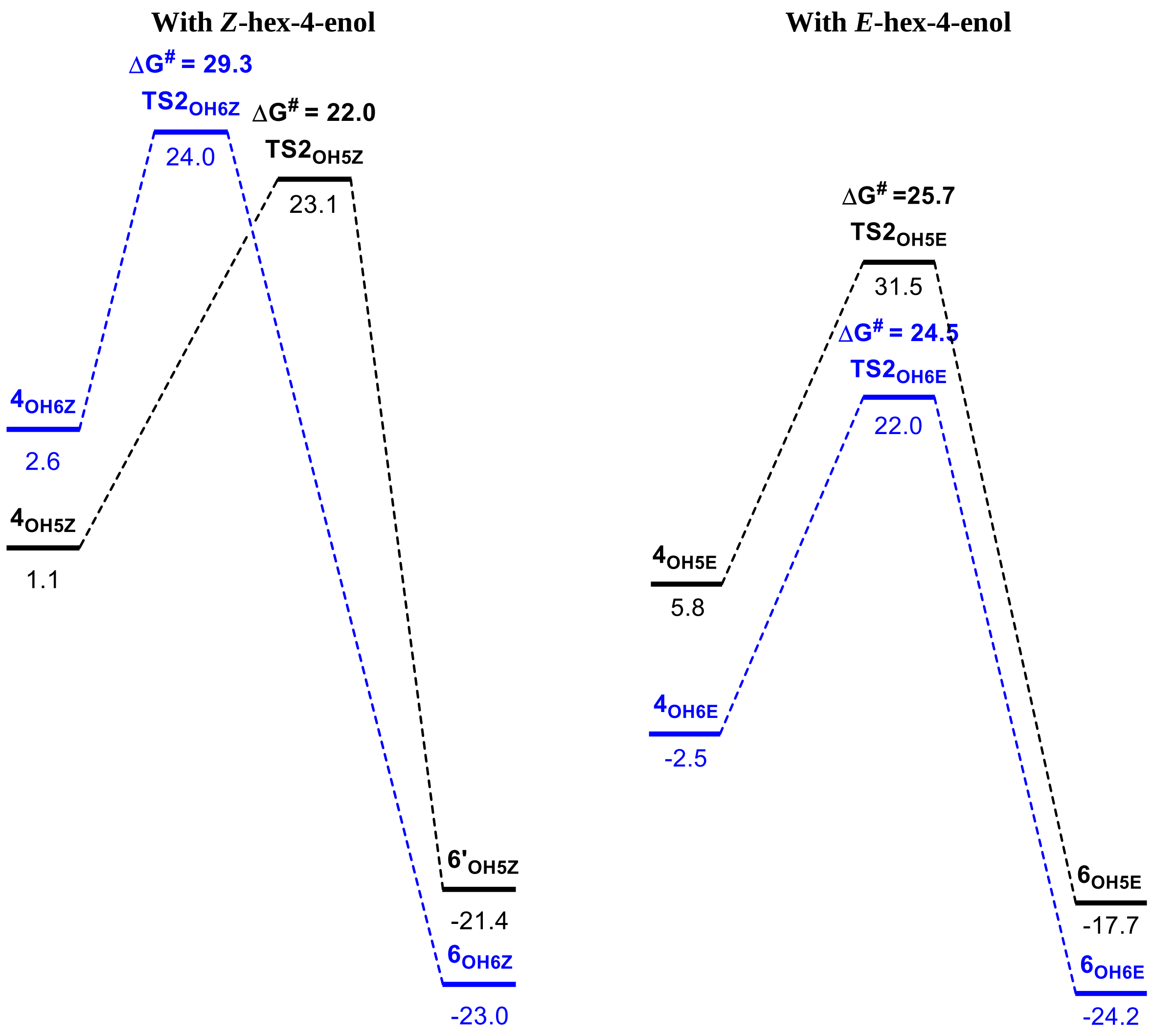

As previously observed for the 4-penten-1-ol, direct reductive elimination from $\mathbf{4}_{\mathbf{OHZ/E}}$ was found more energetically demanding ($\Delta G^{\#}$ = 22-29.3 kcal/mol) than from the non-protonated intermediates $\mathbf{5}_{\mathbf{OZ/E}}$ ($\Delta G^{\#}$ = 15.2-18.2 kcal/mol), meaning that K_3PO_4 facilitates the reductive elimination step however the nature of the alkenol (terminal or internal).

Figure S6. Energy profiles (ΔG in kcal/mol) for the oxy-arylation reaction involving (P,N)Au^{2+}-Ph gold complex **2** and the (*E*)-hex-4-enol, computed at SMD(DCM)-B3PW91-D3(BJ)/SDD+f(Au), 6-31+G**(other atoms)//B3PW91/SDD+ f(Au), 6-31G** (other atoms) level of theory and in the presence of K_3PO_4. Two orientations of the alkenol to gold have been considered, associated to the π-complexes $\mathbf{3}_E$ and $\mathbf{3'}_E$. The K_3PO_4, K_2HPO_4 and K^+ molecules are included in order to ensure the correct energetic balance in all the reaction steps. Geometrical structure and main distances in Å and bond angles in ° for complex $\mathbf{3'}_E$.

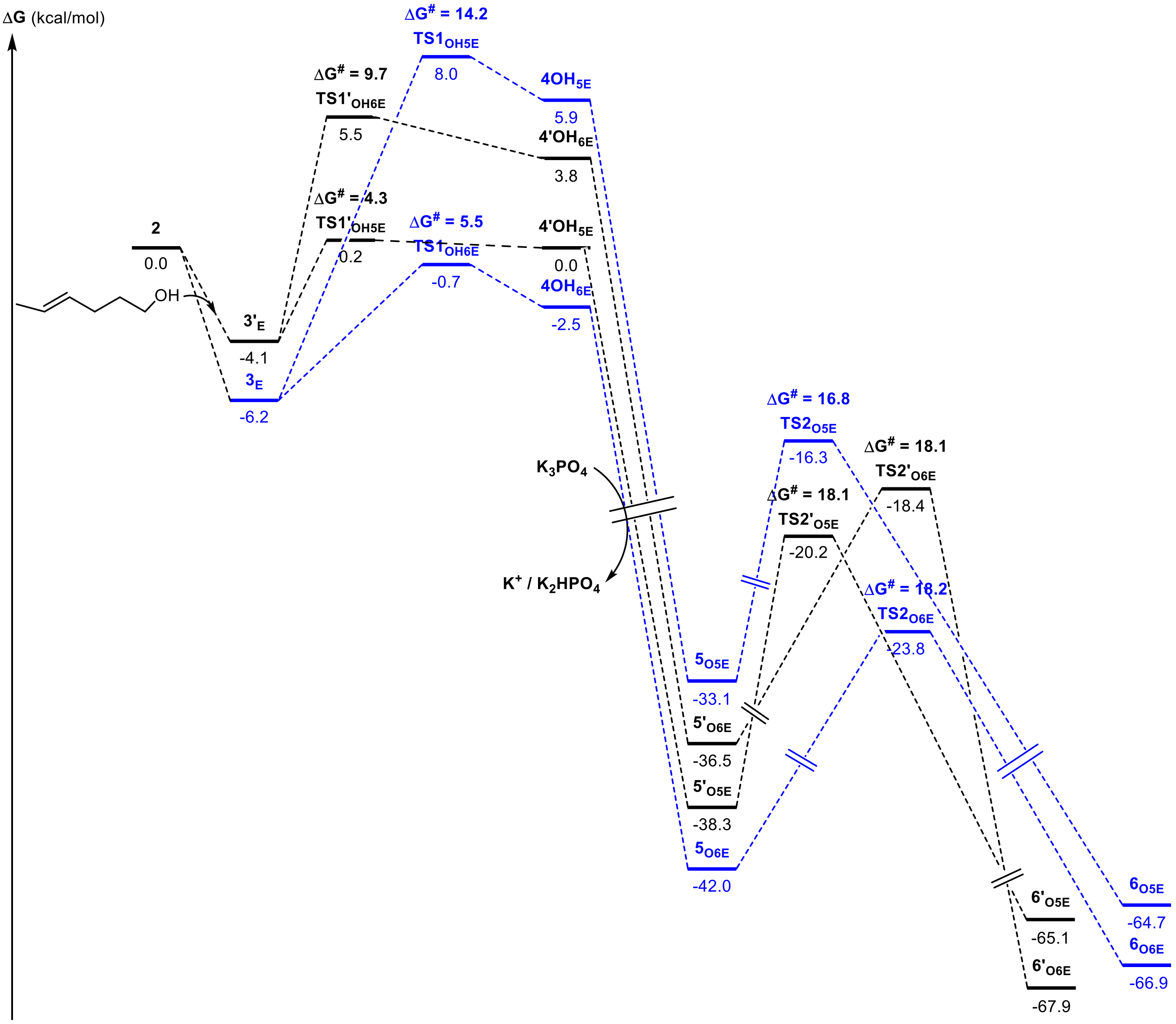

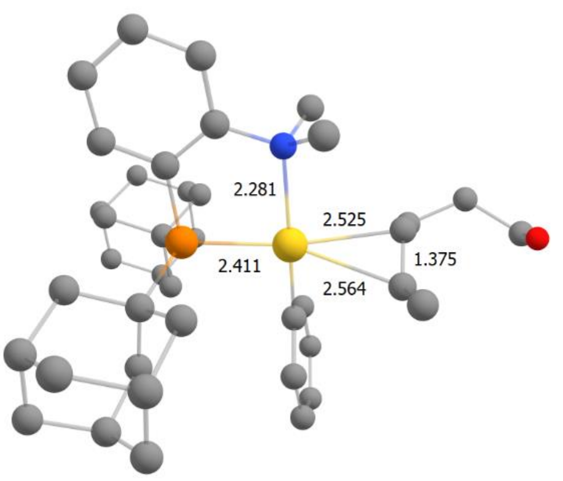

3_E

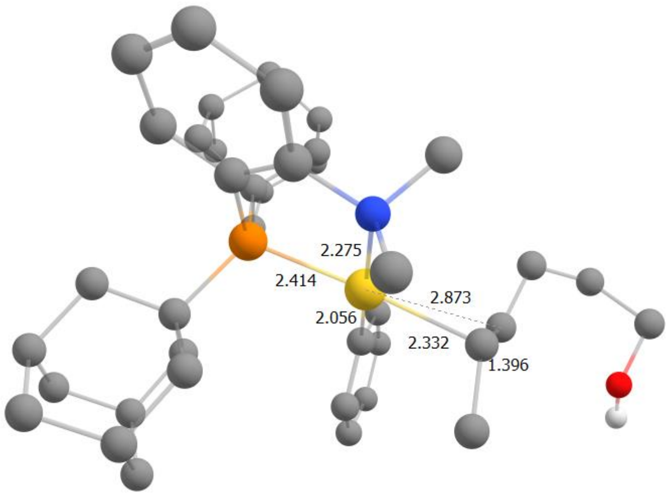

$3'_E$

The two π-complexes **3_E** and **$3'_E$** differ from the approach of the alkene to gold. **3_E** has been found lower in energy than **$3'_E$** by 2.1 kcal/mol. The computed profiles from these two π-complexes **3_E** and **$3'_E$** show that the privileged pathway is the 6-*endo* process through **3_E**. It is to note that the TS associated to the 5-*exo* cyclization step computed from **$3_{E'}$** (**$TS1'_{OH5E}$**) was found lower in energy than that computed from **3_E** (**$TS1_{OH5E}$**), with $\Delta\Delta G^{\#}$ = 7.8 kcal/mol. However, this TS remains higher in energy than the TS associated to the 6-*endo* cyclization step (**$TS1_{OH6E}$**) calculated from **3_E** ($\Delta G^{\#}$ = 6.4 kcal/mol *versus* 5.5 kcal/mol).

Figure S7. Geometrical features of the transition states associated to the 5-*exo* (**$TS1_{OH5}$**) and 6-*endo* (**$TS1_{OH6}$**) cyclization step for the reaction between complex **2** and the 3 alkenols. Main distances in Å and bond angles in°.

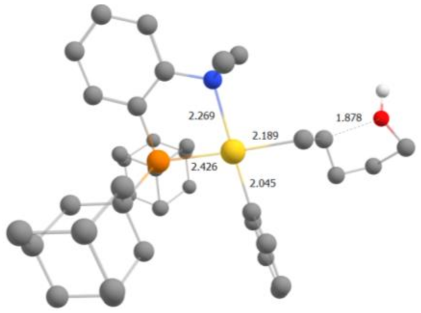

$TS1_{OH5}$

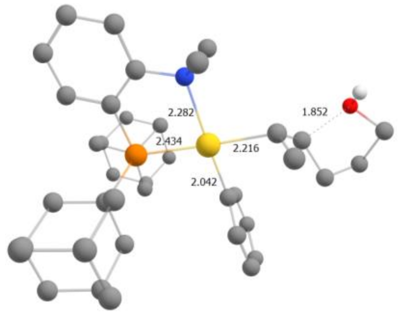

$TS1_{OH5Z}$

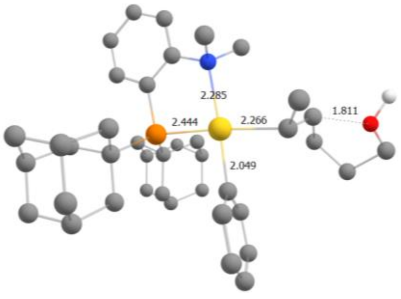

$TS1_{OH5E}$

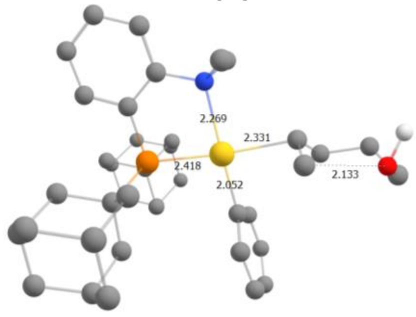

$TS1_{OH6}$

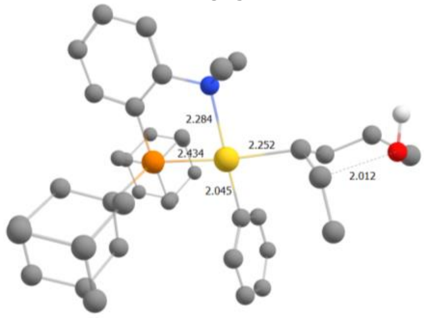

$TS1_{OH6Z}$

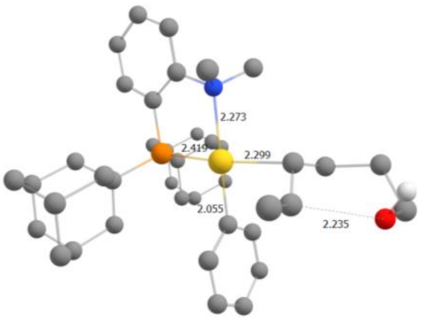

$TS1_{OH6E}$

Figure S8. Plot of the LUMO (cutoff :0.04) for the π-complexes **3**, **3*Z***, **3*E***, **3’*E*** and associated to the oxo-phenylation cyclization with the 3 alkenols. Participation of each atom (in %) for the main atoms.

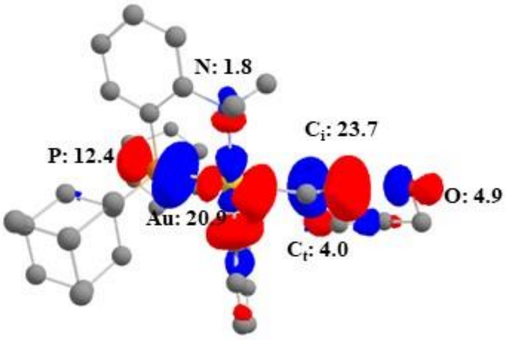

Terminal alkene (3)

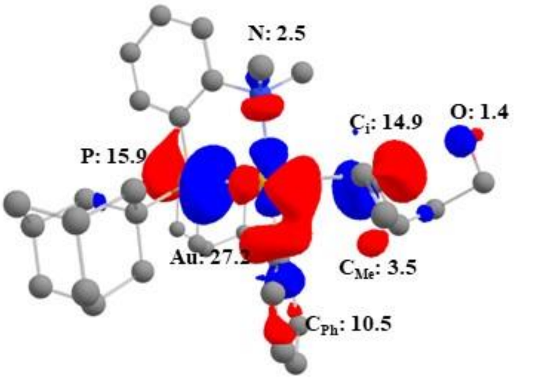

Z-alkene (3_Z)

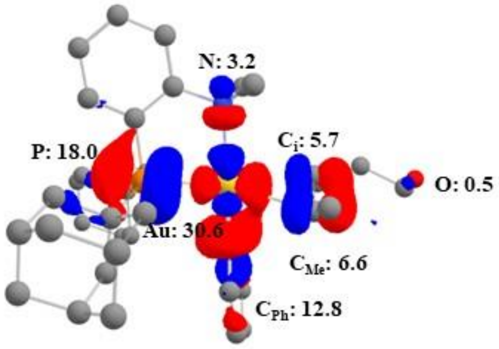

E-alkene (3_E)

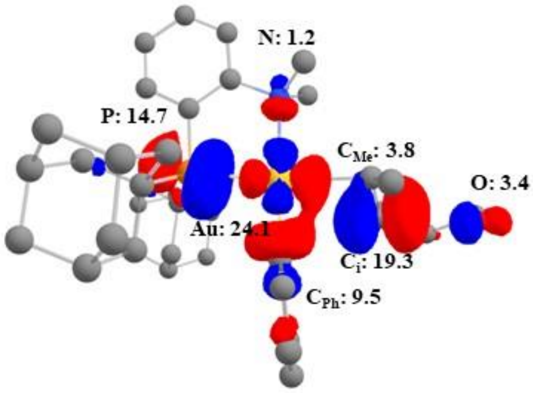

E’-alkene ($3'_E$)

Figure S9. Variation of the Au–C distances, of the coefficients of the C_i and C_t/C_{Me} carbons in the LUMO as well as of the NPA charges in the π-complexes **3**, **3**$_Z$, **3**$_E$ and **3'**$_E$ associated to the terminal and (*Z/E*)-internal alkenols.

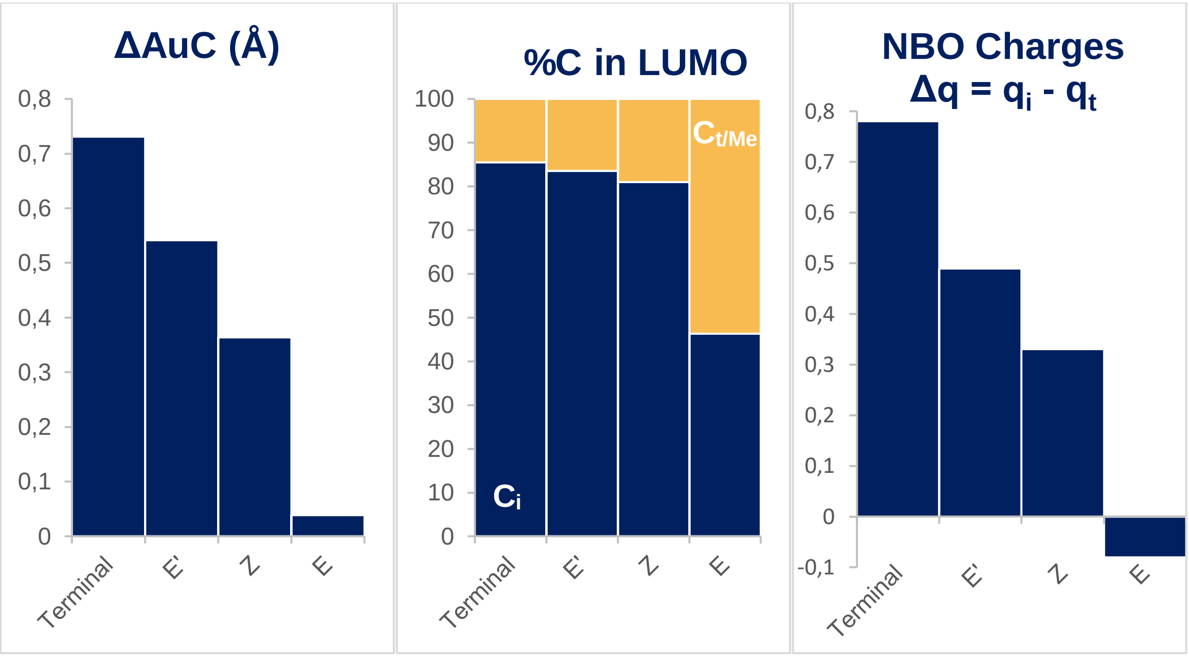

Figure S10. Geometrical structures of the two π-complexes (**3v** and **3v**^{B}) associated to the coordination of 4-penten-1-ol on the (P,N)Au^{2+}-vinyl catalyst (**2v**), computed at B3PW91/SDD+f(Au), 6-31G** (other atoms) level of theory. Relative stability (ΔG) in kcal/mol calculated at SMD(DCM)-B3PW91-D3(BJ)/SDD+f(Au),6-31+G**//B3PW91/SDD+f(Au), 6-31G** (other atoms) level.

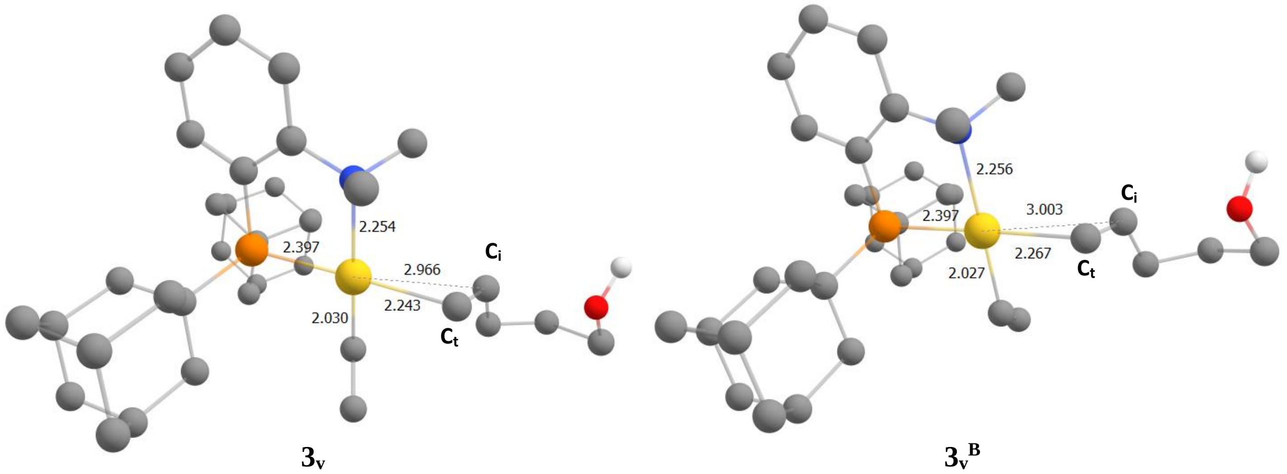

3$_v$ **ΔG = 0 kcal/mol**

3$_v$^{B} **ΔG = 0.6 kcal/mol**

Orientation 1 refers to vinyl substituent directed toward the CH_2 group side (C_t atom, **3**$_v$) of the alkenol and orientation 2 to the vinyl substituent directed toward the alkyl chain side (C_i atom, **3**$_v$^{B}) of the alkenol.

Figure S11. Energy profiles (ΔG in kcal/mol, orientation 2 from **2v^B^**) for the cyclization step of the oxy-vinylation reaction involving (P,N)Au^{2+}-vinyl gold complex **2v** and 4-penten-1-ol computed at SMD(CH_2Cl_2)-B3PW91-D3(BJ)/SDD+f(Au, 6-31+G**(other atoms)//B3PW91/SDD+f(Au), 6-31G** (other atoms) level of theory. Formation of 5-*exo* and 6-*endo* products.

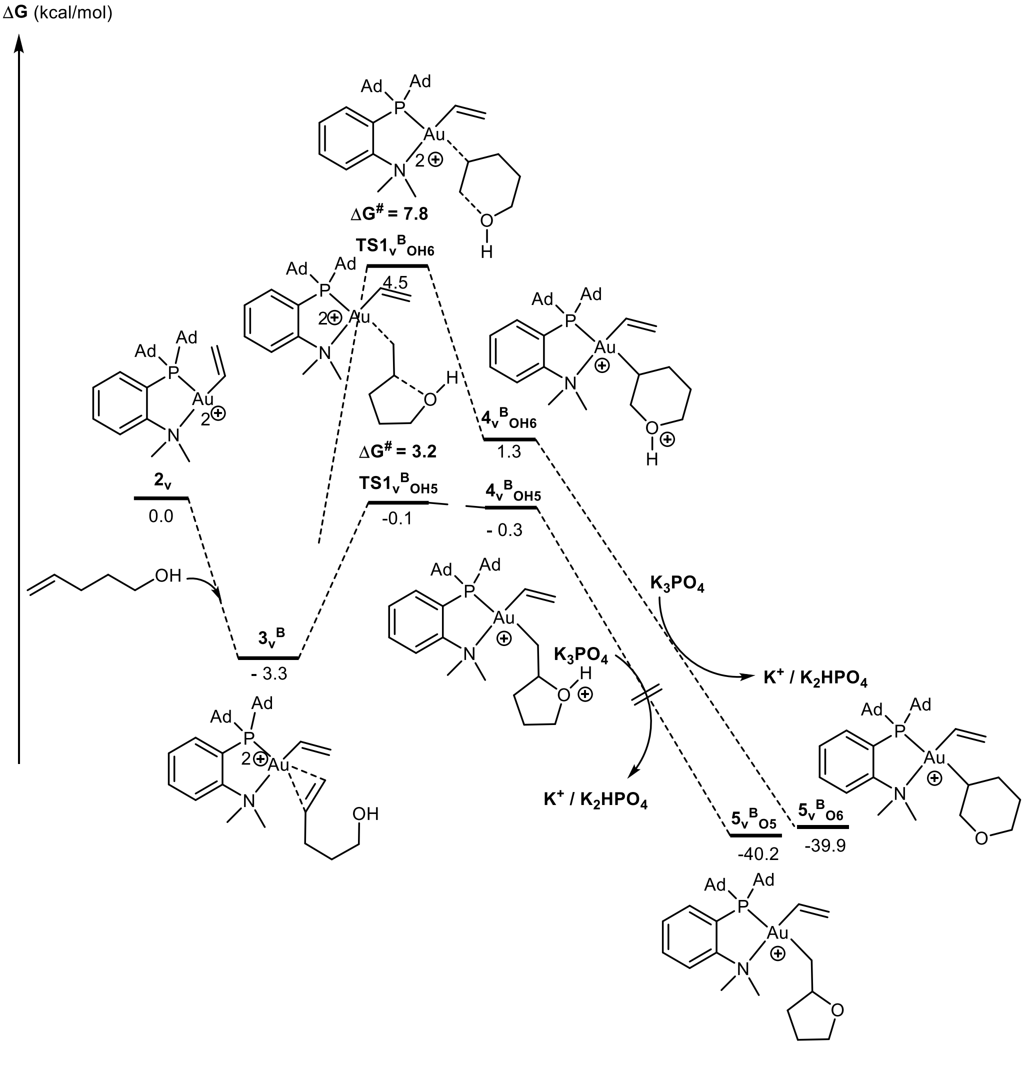

Similar conclusions are obtained for the 2 orientations: orientation 1: vinyl substituent directed toward the CH_2 group side (C_t atom, $\mathbf{3_v}$ see main text) of the alkenol; ii) orientation 2: vinyl substituent directed toward the alkyl chain side (C_i atom, $\mathbf{3_v^B}$) of the alkenol. For the 2nd orientation, the energy difference between the two activation barriers of the nucleophilic attack of 4-penten-1-ol on C_t or C_i atoms of the double bond is $\Delta\Delta G^{\#}$ = 4.6 kcal/mol and in favour of the formation of the 5-*exo* product, as observed for the orientation 1.

Figure S12. Plot of the LUMO (cutoff : 0.04) for the π-complexes **3v**, **3v*Z***, **3v*E*** and **3'v*E*** associated to the 6-*endo* and 5-*exo* cyclizations for the oxy-vinylation of the terminal and Z/E-alkenol substrates. Participation of each atom (in %) for the main atoms.

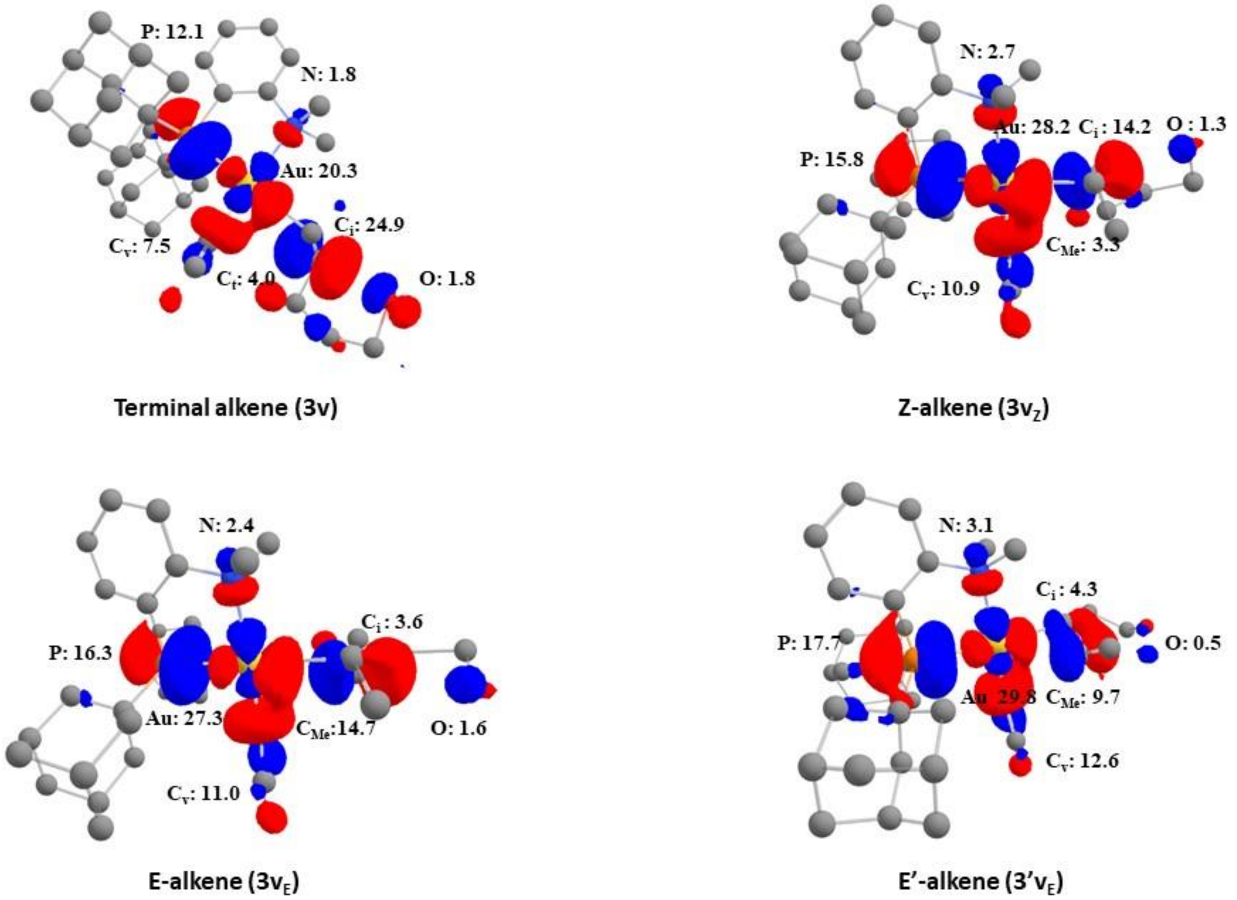

Figure S13. Energy profiles (ΔG in kcal/mol) for the cyclization step of the oxy-vinylation reaction involving (P,N)Au^{2+}-vinyl gold complex **2v** and (*E*)-hex-4-enol, computed at SMD(DCM)-B3PW91-D3(BJ)/SDD+f(Au), 6-31+G**(other atoms)//B3PW91/SDD+f(Au), 6-31G** (other atoms) level of theory. Formation of 5-*exo* and 6-*endo* products.

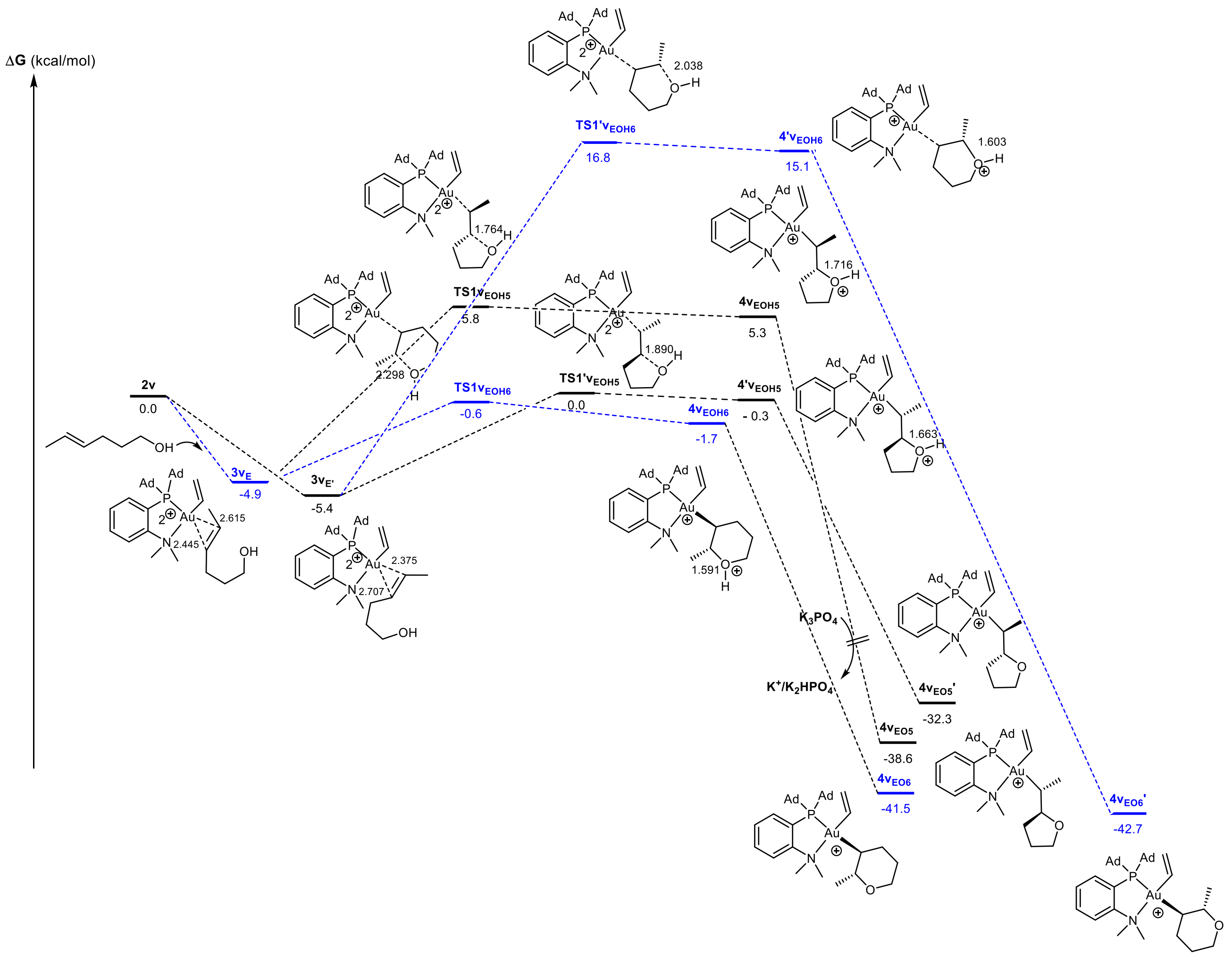

Z-matrices and energy in au

4-Penten-1-ol
Sum of electronic and zero-point Energies = -271.511760
Sum of electronic and thermal Free Energies = -271.543687
Esolv = -271.6981249

C	3.052185000	-0.103774000	-0.433761000
C	1.998412000	-0.229973000	0.371379000
H	3.059662000	0.610582000	-1.254372000
H	3.946343000	-0.706483000	-0.303197000
H	2.035339000	-0.963047000	1.179109000
C	0.724938000	0.555054000	0.261745000
H	0.583591000	1.141152000	1.182749000
H	0.809696000	1.279597000	-0.558249000
C	-0.504621000	-0.336578000	0.050584000
H	-0.583266000	-1.070848000	0.861928000
H	-0.397660000	-0.907396000	-0.878864000
C	-1.800060000	0.454694000	-0.006763000
H	-1.749704000	1.186582000	-0.830876000
H	-1.922343000	1.029657000	0.927271000
O	-2.865252000	-0.459960000	-0.193010000
H	-3.684767000	0.043356000	-0.240524000

4-Penten-1-olate
Sum of electronic and zero-point Energies = -270.896031
Sum of electronic and thermal Free Energies = -270.927562
Esolv = -271.1283776

C	3.006508000	-0.121391000	-0.408626000
C	1.939317000	-0.163986000	0.399974000
H	3.015808000	0.503687000	-1.300037000
H	3.897027000	-0.717831000	-0.221294000
H	1.985117000	-0.819767000	1.274186000
C	0.647216000	0.556729000	0.214238000
H	0.435382000	1.176532000	1.102618000
H	0.722422000	1.249048000	-0.635791000
C	-0.560076000	-0.374188000	0.016259000
H	-0.611289000	-1.110090000	0.832750000
H	-0.448591000	-0.947436000	-0.914620000
C	-1.945492000	0.379279000	-0.022275000
H	-1.773533000	1.198031000	-0.818978000
H	-1.903727000	1.010935000	0.947641000
O	-2.980432000	-0.400221000	-0.182987000

Z-4-Hexen-1-ol
Sum of electronic and zero-point Energies = -310.789480
Sum of electronic and thermal Free Energies = -310.824089
Esolv = -311.0107566

C	2.572059000	-0.423554000	-0.255636000
C	1.379793000	-0.927006000	0.082211000
H	3.308345000	-1.118533000	-0.660484000
H	1.224918000	-1.995392000	-0.075673000
C	0.195750000	-0.200536000	0.650350000
H	-0.035529000	-0.620213000	1.641460000
H	0.427837000	0.857700000	0.814908000
C	-1.048312000	-0.323845000	-0.238701000
H	-1.282905000	-1.380849000	-0.415540000
H	-0.851765000	0.120723000	-1.221035000
C	-2.271061000	0.344298000	0.365819000
H	-2.062235000	1.414609000	0.534544000
H	-2.484100000	-0.101136000	1.352821000
O	-3.356886000	0.165257000	-0.526194000
H	-4.129315000	0.594966000	-0.144247000
C	3.038647000	0.995671000	-0.145685000
H	2.271404000	1.668852000	0.243721000
H	3.912981000	1.069000000	0.513305000
H	3.354201000	1.378055000	-1.124378000

E-4-Hexen-1-ol
Sum of electronic and zero-point Energies = -310.791941
Sum of electronic and thermal Free Energies = -310.826144
Esolv = -311.012279

C	2.407584000	0.185409000	-0.360877000
C	1.348082000	0.116525000	0.448016000
H	2.316129000	0.746708000	-1.292961000
H	1.434575000	-0.447374000	1.379630000
C	0.012830000	0.746784000	0.183125000
H	-0.211677000	1.471868000	0.980570000
H	0.055960000	1.319824000	-0.752224000
C	-1.126193000	-0.278070000	0.115302000
H	-1.162811000	-0.863875000	1.042202000
H	-0.940254000	-0.989824000	-0.697161000
C	-2.484946000	0.366200000	-0.098365000
H	-2.475743000	0.945234000	-1.037601000
H	-2.686351000	1.080966000	0.718037000
O	-3.459025000	-0.661801000	-0.135816000
H	-4.318403000	-0.251823000	-0.279270000
C	3.737985000	-0.448801000	-0.098426000
H	4.538178000	0.301420000	-0.065789000
H	3.744653000	-0.990955000	0.851933000
H	4.005891000	-1.156039000	-0.893480000

K_3PO_4
Sum of electronic and zero-point Energies = -2441.859977
Sum of electronic and thermal Free Energies = -2441.899647
Esolv = -2441.9743451

P	0.19214300	0.05334000	0.00000000
O	-0.05842100	-1.51387800	0.00000000
O	-0.49051000	0.68282200	1.28317100
O	1.69545500	0.43141500	0.00000000
O	-0.49051000	0.68282200	-1.28317100
K	-0.49051000	-1.47644500	-2.45881400
K	-0.49051000	-1.47644500	2.45881400
K	0.55311100	2.79154600	0.00000000

K_2HPO_4
Sum of electronic and zero-point Energies = -1842.572401
Sum of electronic and thermal Free Energies = -1842.608443
Esolv = -1842.6824417

P	-0.000179000	0.703608000	-0.135794000
O	0.000649000	-0.766748000	0.415502000
O	-1.312772000	1.028930000	-0.854977000
O	-0.000152000	1.587169000	1.294068000
O	1.310693000	1.027045000	-0.858568000
H	-0.024187000	2.517904000	1.040232000
K	2.499083000	-0.948050000	0.027556000
K	-2.497002000	-0.951065000	0.026575000

K^+
Sum of electronic and zero-point Energies = -599.671397
Sum of electronic and thermal Free Energies = -599.686573
Esolv = -599.7547459

2

Sum of electronic and zero-point Energies = -1852.958836
Sum of electronic and thermal Free Energies = -1853.022044
Esolv = -1854.1912142

C	-0.917325000	2.644656000	0.199642000
C	-2.297626000	2.917839000	0.284265000
H	-3.040645000	2.184266000	-0.014972000
Au	-0.388758000	1.249130000	-1.103638000
C	0.033329000	3.567254000	0.671914000
H	1.094881000	3.342893000	0.656625000
C	-0.402110000	4.810757000	1.121582000
H	0.325508000	5.545796000	1.452056000
C	-1.767383000	5.108859000	1.154748000
H	-2.098051000	6.072161000	1.531292000
C	-2.710692000	4.171910000	0.729685000
H	-3.770306000	4.406342000	0.762218000
P	0.234869000	-0.718877000	0.031773000
C	0.586663000	-1.753383000	-1.438175000
C	1.846843000	-0.382821000	0.964334000
C	-1.285498000	-1.385385000	0.939357000
C	0.976591000	-3.096284000	-1.279286000
C	0.466852000	-1.235838000	-2.737359000
C	2.754408000	0.468805000	0.044115000
C	2.582034000	-1.718568000	1.268084000
C	1.578130000	0.363151000	2.287253000
C	-0.923105000	-2.706402000	1.674987000
C	-1.827663000	-0.372293000	1.971143000
C	-2.371662000	-1.668314000	-0.124379000
H	1.079951000	-3.525206000	-0.290466000
C	1.242624000	-3.896869000	-2.384402000
C	0.741751000	-2.042882000	-3.841158000
N	0.046076000	0.155166000	-2.955447000
H	2.952135000	-0.067226000	-0.893431000
H	2.258758000	1.417076000	-0.208936000
C	4.085663000	0.767085000	0.763108000
C	3.910965000	-1.401506000	1.993522000
H	2.809305000	-2.249991000	0.338998000
H	1.968133000	-2.371851000	1.897196000
C	2.921491000	0.671380000	2.980381000
H	0.973081000	-0.258691000	2.955381000
H	1.022655000	1.291645000	2.106991000
H	-0.540530000	-3.454721000	0.974356000
H	-0.158867000	-2.525389000	2.438482000
C	-2.199426000	-3.264293000	2.347294000
C	-3.091534000	-0.949158000	2.641031000
H	-1.079416000	-0.155529000	2.738171000
H	-2.069894000	0.576148000	1.480503000
C	-3.628131000	-2.238716000	0.564776000
H	-2.006104000	-2.381830000	-0.871965000
H	-2.633947000	-0.737333000	-0.649577000
H	1.541338000	-4.930066000	-2.239772000
C	1.127944000	-3.368301000	-3.667187000
H	0.655307000	-1.647758000	-4.848228000
C	-1.219816000	0.210181000	-3.749313000
C	1.112232000	0.934964000	-3.654835000
H	4.702843000	1.377634000	0.094198000
C	4.801356000	-0.553343000	1.077999000
C	3.801269000	1.531417000	2.063389000
H	4.397548000	-2.360471000	2.206393000
C	3.633126000	-0.649891000	3.300120000
H	2.704704000	1.213628000	3.907379000
H	-1.916229000	-4.195336000	2.852078000
C	-3.262447000	-3.547756000	1.278749000
C	-2.732323000	-2.252540000	3.367074000
C	-4.164922000	-1.220939000	1.579291000
H	-3.455194000	-0.208322000	3.361817000
H	-4.379487000	-2.432661000	-0.208951000
H	1.337050000	-3.984778000	-4.535601000

2’

Sum of electronic and zero-point Energies = -1852.910412
Sum of electronic and thermal Free Energies = -1852.974745
Esolv = -1854.1408768

C	-3.451831000	-0.066111000	-0.614584000
C	-3.993003000	-1.283999000	-1.046435000
H	-3.552047000	-2.243539000	-0.786414000
Au	-1.463053000	-0.031460000	-0.209847000
C	-4.085261000	1.165208000	-0.827599000
H	-3.716070000	2.093193000	-0.397296000
C	-5.184855000	1.187240000	-1.686776000
H	-5.655344000	2.136424000	-1.925986000
C	-5.690449000	-0.008214000	-2.206083000
H	-6.584836000	0.013225000	-2.822222000
C	-5.093981000	-1.235570000	-1.903084000
H	-5.493956000	-2.159840000	-2.309603000
P	0.869415000	0.021033000	0.216436000
C	0.708941000	-0.072561000	2.041413000
C	1.726524000	-1.546229000	-0.390834000
C	1.577206000	1.696835000	-0.281906000
C	1.851811000	-0.010165000	2.857210000
C	-0.542267000	-0.212442000	2.669337000
C	0.790330000	-2.727220000	-0.036208000
C	3.107679000	-1.805578000	0.249964000
C	1.872708000	-1.467348000	-1.928024000
C	3.117912000	1.786602000	-0.232368000
C	1.092088000	1.991090000	-1.723416000
C	0.971623000	2.761230000	0.663703000
H	2.824439000	0.116783000	2.395386000
C	1.769771000	-0.102479000	4.241092000
C	-0.624560000	-0.316970000	4.061535000
N	-1.850144000	-0.244168000	1.930503000
H	0.661940000	-2.796650000	1.052501000
H	-0.204889000	-2.566099000	-0.485984000
C	1.377507000	-4.045464000	-0.577540000
C	3.689791000	-3.122161000	-0.305314000
H	3.009879000	-1.891629000	1.338095000
H	3.796283000	-0.980255000	0.039864000
C	2.453601000	-2.792196000	-2.460065000
H	2.541313000	-0.645256000	-2.206541000
H	0.897453000	-1.272738000	-2.398890000
H	3.484977000	1.583725000	0.780981000
H	3.566893000	1.043945000	-0.901168000
C	3.559219000	3.199154000	-0.664558000
C	1.547657000	3.399376000	-2.151164000
H	1.476644000	1.247699000	-2.428879000
H	-0.010910000	1.935753000	-1.768129000
C	1.425552000	4.166831000	0.227048000
H	1.282332000	2.578414000	1.698503000
H	-0.129447000	2.705653000	0.637083000
H	2.670161000	-0.049388000	4.844395000
C	0.525871000	-0.265634000	4.841880000
H	-1.579561000	-0.434859000	4.562730000
C	-2.706042000	0.904886000	2.367740000
C	-2.537226000	-1.555007000	2.152602000
H	0.697475000	-4.862933000	-0.310446000
C	2.755744000	-4.284097000	0.057591000
C	1.513830000	-3.951241000	-2.103205000
H	4.672615000	-3.278830000	0.153189000
C	3.834635000	-3.024832000	-1.829993000

H	2.548486000	-2.710207000	-3.548634000
H	4.653385000	3.243299000	-0.622373000
C	2.959047000	4.241455000	0.288867000
C	3.083450000	3.466455000	-2.098755000
C	0.947598000	4.445170000	-1.203902000
H	1.201819000	3.576112000	-3.175859000
H	0.990292000	4.898610000	0.917449000
H	0.438819000	-0.347055000	5.920733000
H	-3.676122000	0.840714000	1.879161000
H	-2.207453000	1.841650000	2.117294000
H	-2.839585000	0.859136000	3.451355000
H	-2.663792000	-1.713971000	3.226686000
H	-1.920430000	-2.358782000	1.749896000
H	-3.513492000	-1.539083000	1.671756000
H	3.171552000	-5.230308000	-0.306250000
H	2.664980000	-4.377932000	1.147354000
H	0.530071000	-3.804279000	-2.569355000
H	1.910687000	-4.891850000	-2.500998000
H	4.519479000	-2.210961000	-2.099594000
H	4.274134000	-3.947937000	-2.224237000
H	3.293988000	5.245701000	0.006407000
H	3.309326000	4.070067000	1.314647000
H	3.521816000	2.737443000	-2.791781000
H	3.420016000	4.455758000	-2.428767000
H	-0.149753000	4.423109000	-1.254770000
H	1.253714000	5.450950000	-1.512477000

3

Sum of electronic and zero-point Energies = -2124.503628
Sum of electronic and thermal Free Energies = -2124.576513
Esolv = -2125.923284

C	1.504141000	-0.722707000	-1.469540000
C	1.651028000	-2.058705000	-1.854291000
H	1.482657000	-2.871603000	-1.152893000
Au	1.108028000	-0.365290000	0.506984000
C	1.732916000	0.302389000	-2.384145000
H	1.634825000	1.345234000	-2.101201000
C	2.100859000	-0.013167000	-3.697322000
H	2.273010000	0.789728000	-4.408529000
C	2.242033000	-1.340691000	-4.090420000
H	2.525586000	-1.580758000	-5.110334000
C	2.019452000	-2.361559000	-3.167319000
H	2.132933000	-3.401113000	-3.460903000
P	-1.226460000	0.189927000	0.261709000
C	-1.635338000	0.434344000	2.036089000
C	-1.462766000	1.889022000	-0.586199000
C	-2.254804000	-1.290278000	-0.359776000
C	-2.941338000	0.791335000	2.425045000
C	-0.669247000	0.284265000	3.039205000
C	-0.264966000	2.769249000	-0.154138000
C	-2.759751000	2.610725000	-0.143074000
C	-1.497998000	1.738843000	-2.122731000
C	-3.777233000	-1.018051000	-0.298251000
C	-1.878319000	-1.668714000	-1.809964000
C	-1.918591000	-2.476165000	0.575609000
H	-3.711772000	0.920304000	1.677920000
C	-3.274389000	0.998858000	3.757304000
C	-1.006941000	0.498970000	4.378815000
N	0.705329000	-0.156707000	2.728585000
H	-0.268381000	2.891765000	0.937816000
H	0.685054000	2.289338000	-0.426715000
C	-0.353460000	4.151151000	-0.831100000
C	-2.847266000	3.993063000	-0.827567000
H	-2.762299000	2.765641000	0.939525000
H	-3.643920000	2.018081000	-0.405358000
C	-1.556428000	3.131692000	-2.781677000
H	-2.392378000	1.178421000	-2.415733000
H	-0.631141000	1.181340000	-2.488694000
H	-4.088661000	-0.777088000	0.721604000
H	-4.044700000	-0.175597000	-0.946843000
C	-4.544682000	-2.281679000	-0.745273000
C	-2.652786000	-2.931220000	-2.237979000
H	-2.119527000	-0.853816000	-2.497179000
H	-0.804827000	-1.852980000	-1.891330000
C	-2.699471000	-3.729743000	0.132551000
H	-2.178074000	-2.230571000	1.613150000
H	-0.839025000	-2.688542000	0.540269000
H	-4.290208000	1.273465000	4.022425000
C	-2.299717000	0.857778000	4.739679000
H	-0.266994000	0.378080000	5.162732000
C	0.900058000	-1.504351000	3.347081000
C	1.686816000	0.815255000	3.287226000
H	0.513495000	4.744973000	-0.517463000
C	-1.649296000	4.848423000	-0.396944000
C	-0.344442000	3.972083000	-2.355956000
H	-3.779541000	4.465870000	-0.497979000
C	-2.850820000	3.832532000	-2.351949000
H	-1.549474000	2.992520000	-3.868754000
H	-5.615946000	-2.056580000	-0.689586000
C	-4.205811000	-3.441319000	0.200990000
C	-4.158966000	-2.649110000	-2.182409000
C	-2.304688000	-4.097141000	-1.303692000
H	-2.355944000	-3.174705000	-3.264607000
H	-2.445332000	-4.549681000	0.814489000
H	-2.541114000	1.019378000	5.785352000
H	1.901323000	-1.879110000	3.136500000
H	0.162488000	-2.195521000	2.937670000
H	0.774049000	-1.453348000	4.432063000
H	1.590770000	0.902279000	4.372503000
H	1.520235000	1.795109000	2.837083000
H	2.699116000	0.474873000	3.068958000
H	-1.711949000	5.841642000	-0.855677000
H	-1.656525000	5.001349000	0.689906000
H	0.589248000	3.493937000	-2.682129000
H	-0.378091000	4.951615000	-2.846216000
H	-3.724161000	3.253044000	-2.676679000
H	-2.928943000	4.814816000	-2.831711000
H	-4.769637000	-4.336028000	-0.086055000
H	-4.503335000	-3.197760000	1.229092000
H	-4.421807000	-1.835653000	-2.870559000
H	-4.722289000	-3.531153000	-2.507626000
H	-1.232004000	-4.325881000	-1.359270000
H	-2.835358000	-5.003232000	-1.617317000
C	3.250484000	-1.002121000	0.850353000
H	3.346161000	-1.885412000	0.222570000
H	3.203563000	-1.218675000	1.914106000
C	4.064786000	0.090283000	0.510642000
C	4.657293000	0.337234000	-0.824875000
H	4.136123000	0.913789000	1.221890000
C	6.874487000	-0.324456000	0.065582000
C	6.139221000	0.744617000	-0.727666000
H	4.528409000	-0.534949000	-1.471481000
H	4.101322000	1.167957000	-1.284410000
H	7.907208000	-0.034456000	0.284371000
H	6.895522000	-1.274920000	-0.474613000
H	6.559717000	0.856674000	-1.731480000
H	6.233295000	1.716885000	-0.228316000
H	6.482589000	0.026573000	1.963005000
O	6.146963000	-0.565649000	1.276712000

3'
Sum of electronic and zero-point Energies = -2124.518178
Sum of electronic and thermal Free Energies = -2124.591854
Esolv = -2125.9315961

C	-4.025786000	3.026361000	-1.960243000
C	-4.226455000	2.800223000	-0.457582000
C	-3.562885000	3.934345000	0.333774000
C	-2.060778000	3.955865000	0.020475000
C	-1.843609000	4.174800000	-1.482889000
C	-2.522966000	3.043986000	-2.266287000
C	-3.605843000	1.450561000	-0.030473000
C	-2.090695000	1.456080000	-0.355909000
C	-1.431559000	2.610945000	0.436695000
C	-1.915798000	1.681974000	-1.872830000
P	-1.237344000	-0.150810000	0.217664000
Au	1.063924000	0.194003000	0.488920000
C	-1.552095000	-1.798234000	-0.691187000
C	-1.281590000	-1.680341000	-2.206658000
C	-1.447186000	-3.062908000	-2.870462000
C	-2.891615000	-3.541370000	-2.678516000
C	-3.181270000	-3.664387000	-1.178546000
C	-3.005253000	-2.294084000	-0.485800000
C	-2.222146000	-4.678713000	-0.542492000
C	-0.776880000	-4.203515000	-0.744934000
C	-0.475351000	-4.070928000	-2.243609000
C	-0.587936000	-2.833457000	-0.062790000
C	-1.667371000	-0.403111000	1.986988000
C	-0.692859000	-0.346133000	2.993091000
C	-1.047658000	-0.540886000	4.329395000
C	-2.368083000	-0.794855000	4.682258000
C	-3.347524000	-0.855425000	3.695640000
C	-2.999549000	-0.660892000	2.364339000
N	0.716453000	-0.082663000	2.668588000
C	1.177840000	1.167258000	3.342019000
C	1.568319000	-1.229625000	3.100428000
C	3.986692000	1.698870000	0.700421000
C	5.055879000	1.496736000	-0.358063000
C	1.587567000	0.467116000	-1.459585000
C	1.649670000	1.760077000	-1.980248000
C	2.248989000	1.969423000	-3.226620000
C	2.773833000	0.896768000	-3.944045000
C	2.711308000	-0.390795000	-3.413537000
C	2.116580000	-0.613446000	-2.168306000
H	-0.296294000	-0.496589000	5.110954000
H	-2.626966000	-0.944119000	5.725598000
H	-4.381997000	-1.052661000	3.957468000
H	-3.779285000	-0.710025000	1.616345000
H	0.455388000	-2.507685000	-0.187923000
H	-0.781489000	-2.927921000	1.013741000
H	-0.081863000	-4.913667000	-0.281751000
H	1.223196000	-2.142251000	2.612384000
H	1.518880000	-1.365346000	4.184539000
H	2.602503000	-1.022471000	2.823658000
H	0.563713000	-3.751419000	-2.399604000
H	-0.578501000	-5.045941000	-2.732833000
H	-1.229690000	-2.949363000	-3.938499000
H	-3.592381000	-2.841180000	-3.150358000
H	-3.038330000	-4.510785000	-3.167944000
H	-4.218295000	-3.981654000	-1.020294000
H	-3.222792000	-2.422439000	0.577831000
H	-3.722624000	-1.576727000	-0.901320000
H	-2.358507000	-5.663449000	-1.003139000
H	-2.440829000	-4.797762000	0.526434000
H	-1.994013000	-0.989499000	-2.664890000
H	-0.279334000	-1.287538000	-2.397547000
H	-1.571272000	2.456564000	1.514861000
H	-0.349844000	2.641950000	0.241755000
H	-1.570990000	4.752874000	0.592075000
H	2.213198000	1.355906000	3.058035000
H	1.125029000	1.065689000	4.429700000
H	0.545585000	2.000705000	3.032615000
H	-4.014209000	4.895124000	0.062315000
H	-3.728609000	3.801238000	1.410526000
H	-5.296219000	2.758081000	-0.223099000
H	-4.524327000	2.238671000	-2.539021000
H	-4.485331000	3.975088000	-2.259465000
H	-2.361736000	3.185248000	-3.340951000
H	-2.438885000	0.898678000	-2.428520000
H	-0.861540000	1.637501000	-2.160605000
H	-4.113642000	0.631206000	-0.552684000
H	-3.768440000	1.327051000	1.043652000
H	-2.261582000	5.142228000	-1.782845000
H	-0.770954000	4.213609000	-1.715078000
H	2.081648000	-1.623415000	-1.770434000
H	3.121486000	-1.231152000	-3.966175000
H	2.296775000	2.975856000	-3.632141000
H	1.246033000	2.610554000	-1.438957000
H	3.229441000	1.063142000	-4.915215000
H	3.268901000	2.463528000	0.396239000
H	4.429147000	1.993580000	1.658420000
H	4.586729000	1.265401000	-1.321272000
H	3.785889000	-0.295697000	0.784275000
O	3.206972000	0.483547000	0.969882000
H	5.555402000	2.465322000	-0.479045000
C	6.102002000	0.428011000	0.001654000
H	6.925545000	0.500137000	-0.719098000
H	6.532446000	0.635514000	0.988747000
C	5.556231000	-0.973225000	-0.044523000
C	5.477918000	-1.811993000	0.998387000
H	5.841833000	-1.529671000	1.984577000
H	5.114650000	-2.829414000	0.882872000
H	5.224064000	-1.320859000	-1.024305000

3_Z
Sum of electronic and zero-point Energies = -2163.779103
Sum of electronic and thermal Free Energies = -2163.854292
Esolv = -2165.236817

Au	1.028735000	-0.482098000	0.409915000
P	-1.244952000	0.304589000	0.286625000
C	-1.505097000	0.656933000	2.069816000
C	-1.326325000	1.994399000	-0.615807000
C	-2.462442000	-1.084134000	-0.200545000
C	-2.737308000	1.178230000	2.512507000
C	-0.503795000	0.433695000	3.023322000
C	-0.015412000	2.741032000	-0.269442000
C	-2.510219000	2.868923000	-0.133412000
C	-1.458842000	1.810739000	-2.142902000
C	-3.935686000	-0.699320000	0.081746000
C	-2.331563000	-1.462777000	-1.692411000
C	-2.094806000	-2.303004000	0.678877000
H	-3.530295000	1.370050000	1.803470000
C	-2.966484000	1.470003000	3.850738000
C	-0.739102000	0.730107000	4.369427000
N	0.795599000	-0.164961000	2.657367000
H	0.054356000	2.889303000	0.816950000
H	0.858019000	2.149944000	-0.577731000
C	0.016367000	4.107706000	-0.982077000
C	-2.476189000	4.234793000	-0.855680000
H	-2.440339000	3.052515000	0.942149000

H	-3.467146000	2.373025000	-0.335370000
C	-1.390243000	3.184349000	-2.840732000
H	-2.427501000	1.356321000	-2.374229000
H	-0.685195000	1.144733000	-2.534769000
H	-4.077658000	-0.460243000	1.138823000
H	-4.228381000	0.174692000	-0.512469000
C	-4.854171000	-1.893778000	-0.261184000
C	-3.246910000	-2.663003000	-2.007414000
H	-2.626086000	-0.622881000	-2.326176000
H	-1.297560000	-1.710229000	-1.941849000
C	-3.022255000	-3.488657000	0.344502000
H	-2.196245000	-2.047139000	1.741618000
H	-1.049303000	-2.597050000	0.502287000
H	-3.926576000	1.871764000	4.157943000
C	-1.959667000	1.247860000	4.784342000
H	0.025774000	0.551960000	5.117436000
C	0.868659000	-1.510066000	3.308045000
C	1.902544000	0.707396000	3.144880000
H	0.959275000	4.606089000	-0.727282000
C	-1.169248000	4.957928000	-0.507325000
C	-0.071538000	3.891360000	-2.499338000
H	-3.331698000	4.817925000	-0.496018000
C	-2.574219000	4.038927000	-2.372561000
H	-1.454128000	3.016804000	-3.921900000
H	-5.885698000	-1.586856000	-0.053583000
C	-4.478092000	-3.089835000	0.623340000
C	-4.704606000	-2.270293000	-1.739078000
C	-2.858686000	-3.863192000	-1.134351000
H	-3.116361000	-2.914586000	-3.066118000
H	-2.738552000	-4.334886000	0.981230000
H	-2.120041000	1.471076000	5.834242000
H	1.813081000	-1.999731000	3.071356000
H	0.043405000	-2.126273000	2.949432000
H	0.795260000	-1.417381000	4.395101000
H	1.868922000	0.825834000	4.230594000
H	1.816789000	1.690773000	2.680129000
H	2.865681000	0.262411000	2.894574000
H	-1.142638000	5.940518000	-0.991598000
H	-1.104323000	5.136794000	0.573613000
H	0.784966000	3.302575000	-2.854711000
H	-0.019719000	4.855926000	-3.016807000
H	-3.522587000	3.555573000	-2.638832000
H	-2.564376000	5.011911000	-2.876777000
H	-5.142654000	-3.935230000	0.412732000
H	-4.611321000	-2.840902000	1.684033000
H	-4.999082000	-1.431100000	-2.381867000
H	-5.373787000	-3.103367000	-1.982430000
H	-1.824250000	-4.168607000	-1.340449000
H	-3.493070000	-4.724032000	-1.373855000
C	3.790433000	-0.183272000	0.286242000
H	3.706541000	0.690571000	0.930557000
C	3.250084000	-1.372207000	0.750543000
C	4.511157000	0.038119000	-0.990811000
H	4.789280000	-0.912234000	-1.455603000
H	3.822007000	0.529381000	-1.692971000
C	6.784828000	0.341870000	0.098632000
H	7.622005000	1.041654000	0.222063000
H	7.181987000	-0.585128000	-0.340130000
C	5.740475000	0.951276000	-0.816451000
H	5.427895000	1.921416000	-0.411687000
H	6.181085000	1.141026000	-1.799794000
C	3.510191000	-2.723934000	0.150362000
H	4.487179000	-3.077647000	0.503479000
H	2.771218000	-3.458712000	0.480411000
H	3.523956000	-2.717581000	-0.939277000
H	3.058333000	-1.387368000	1.820571000
C	1.201793000	-0.949719000	-1.573583000
C	1.534401000	-0.003312000	-2.541115000
C	1.061246000	-2.295501000	-1.920186000
C	1.742743000	-0.411821000	-3.863254000
H	1.634017000	1.048612000	-2.293414000
C	1.275721000	-2.692139000	-3.242960000
H	0.794657000	-3.045901000	-1.181876000
C	1.614192000	-1.752340000	-4.214727000
H	2.002846000	0.329214000	-4.613646000
H	1.173350000	-3.740988000	-3.505882000
H	1.774024000	-2.064519000	-5.241965000
H	6.762545000	-0.376267000	1.922958000
O	6.145198000	0.086004000	1.343656000

3_E

Sum of electronic and zero-point Energies = -2163.777977
Sum of electronic and thermal Free Energies = -2163.853178
Esolv = -2165,2396059

C	1.229063000	0.193498000	-1.805357000
C	1.078651000	-0.803802000	-2.767118000
H	0.820324000	-1.821449000	-2.492961000
Au	1.142725000	-0.201362000	0.213043000
C	1.599392000	1.486932000	-2.173383000
H	1.722009000	2.274813000	-1.436471000
C	1.815352000	1.780989000	-3.524075000
H	2.101562000	2.789440000	-3.808337000
C	1.660607000	0.793410000	-4.493547000
H	1.827982000	1.026334000	-5.540363000
C	1.295682000	-0.496292000	-4.113786000
H	1.183042000	-1.276457000	-4.860995000
P	-1.253304000	0.023903000	0.351225000
C	-1.441144000	-0.166224000	2.166252000
C	-1.845987000	1.789303000	-0.087905000
C	-2.101409000	-1.497733000	-0.451442000
C	-2.723140000	-0.124881000	2.750590000
C	-0.337939000	-0.347484000	3.008397000
C	-0.882244000	2.755001000	0.642042000
C	-3.285219000	2.064737000	0.413744000
C	-1.802318000	2.051982000	-1.609267000
C	-3.480418000	-1.806178000	0.183625000
C	-2.306201000	-1.298997000	-1.968449000
C	-1.174007000	-2.707423000	-0.184062000
H	-3.598318000	0.000730000	2.128779000
C	-2.902264000	-0.251119000	4.121723000
C	-0.523341000	-0.470271000	4.389111000
N	1.042118000	-0.390639000	2.483917000
H	-0.923601000	2.582167000	1.725518000
H	0.152867000	2.580006000	0.314675000
C	-1.274494000	4.214974000	0.337832000
C	-3.669173000	3.528530000	0.099373000
H	-3.350345000	1.927768000	1.496017000
H	-3.999212000	1.383522000	-0.064920000
C	-2.171341000	3.521966000	-1.894461000
H	-2.519047000	1.408166000	-2.124654000
H	-0.814283000	1.830647000	-2.017327000
H	-3.379258000	-1.986691000	1.257315000
H	-4.170429000	-0.965962000	0.039871000
C	-4.075790000	-3.078158000	-0.461653000
C	-2.870429000	-2.592261000	-2.591277000
H	-3.027523000	-0.493819000	-2.138590000
H	-1.374933000	-1.011439000	-2.463784000
C	-1.772175000	-3.980071000	-0.816022000
H	-1.051718000	-2.853995000	0.897949000
H	-0.176352000	-2.527616000	-0.608578000

H	-3.902929000	-0.216204000	4.540049000
C	-1.795012000	-0.422854000	4.946090000
H	0.324529000	-0.601161000	5.052661000
C	1.679540000	-1.670017000	2.908969000
C	1.788268000	0.774813000	3.050618000
H	-0.574264000	4.874136000	0.864435000
C	-2.704996000	4.470725000	0.831970000
C	-1.194051000	4.460327000	-1.174734000
H	-4.691240000	3.685618000	0.462773000
C	-3.602000000	3.784299000	-1.409843000
H	-2.108612000	3.678194000	-2.977412000
H	-5.051555000	-3.256630000	0.004520000
C	-3.143427000	-4.265059000	-0.189699000
C	-4.243082000	-2.882692000	-1.972590000
C	-1.922200000	-3.770153000	-2.329257000
H	-2.973084000	-2.429486000	-3.670147000
H	-1.089896000	-4.815201000	-0.617757000
H	-1.915275000	-0.520699000	6.020241000
H	2.712550000	-1.696055000	2.567918000
H	1.128294000	-2.507179000	2.478670000
H	1.683692000	-1.769629000	3.997060000
H	1.809761000	0.728977000	4.142814000
H	1.293740000	1.698373000	2.746786000
H	2.814276000	0.774285000	2.687077000
H	-2.983900000	5.513361000	0.642787000
H	-2.768986000	4.319283000	1.917194000
H	-0.170132000	4.297442000	-1.536186000
H	-1.442274000	5.504288000	-1.396869000
H	-4.310680000	3.137542000	-1.942331000
H	-3.894288000	4.817879000	-1.627367000
H	-3.566371000	-5.181018000	-0.617217000
H	-3.043993000	-4.437363000	0.889712000
H	-4.937619000	-2.059091000	-2.180534000
H	-4.679622000	-3.782933000	-2.419775000
H	-0.942637000	-3.587133000	-2.791233000
H	-2.318587000	-4.679717000	-2.794343000
C	3.661019000	-0.211482000	0.400262000
H	3.794569000	-0.504657000	1.439404000
C	3.387507000	-1.215909000	-0.497825000
H	3.410056000	-0.950054000	-1.551888000
C	4.179702000	1.150570000	0.035941000
H	4.114345000	1.299383000	-1.046092000
H	3.570824000	1.932265000	0.509575000
C	3.349978000	-2.676262000	-0.208181000
H	3.265568000	-2.918169000	0.851250000
H	2.568339000	-3.194239000	-0.771414000
H	4.308332000	-3.081364000	-0.560283000
H	7.100297000	-1.469747000	0.060522000
C	6.653708000	0.424106000	-0.117121000
H	7.654358000	0.714935000	0.228847000
H	6.640172000	0.532583000	-1.213113000
C	5.634511000	1.355583000	0.514761000
H	5.687432000	1.246569000	1.604782000
H	5.914701000	2.389610000	0.286586000
O	6.345216000	-0.908524000	0.270938000

3’$_E$

Sum of electronic and zero-point Energies = -2163.779086
Sum of electronic and thermal Free Energies = -2163.853564
Esolv = -2165.2373838

C	1.369011000	-0.463377000	-1.629444000
C	1.336842000	-1.714263000	-2.246014000
H	1.059174000	-2.608153000	-1.696234000
Au	1.068052000	-0.350644000	0.401302000
C	1.725279000	0.671355000	-2.358939000
H	1.764132000	1.652142000	-1.894699000
C	2.048449000	0.549333000	-3.713928000
H	2.324693000	1.436305000	-4.276934000
C	2.015977000	-0.696366000	-4.336981000
H	2.265610000	-0.786705000	-5.389473000
C	1.660031000	-1.824871000	-3.602641000
H	1.627248000	-2.800791000	-4.078360000
P	-1.283170000	0.182079000	0.275072000
C	-1.639984000	0.284288000	2.072020000
C	-1.576313000	1.935368000	-0.435931000
C	-2.282169000	-1.303100000	-0.399013000
C	-2.941781000	0.581283000	2.522842000
C	-0.641948000	0.077474000	3.031773000
C	-0.442945000	2.822911000	0.133145000
C	-2.928479000	2.546016000	0.006760000
C	-1.530136000	1.933944000	-1.979268000
C	-3.785044000	-1.225542000	-0.035715000
C	-2.156991000	-1.428448000	-1.933044000
C	-1.682864000	-2.558299000	0.280603000
H	-3.736879000	0.747063000	1.809347000
C	-3.240868000	0.672027000	3.875911000
C	-0.946883000	0.173061000	4.393223000
N	0.747002000	-0.253223000	2.651049000
H	-0.494349000	2.837567000	1.230272000
H	0.539449000	2.416198000	-0.146138000
C	-0.575055000	4.259243000	-0.410482000
C	-3.056424000	3.982415000	-0.548533000
H	-2.988151000	2.599903000	1.097066000
H	-3.765965000	1.936495000	-0.353408000
C	-1.631033000	3.379611000	-2.505724000
H	-2.377384000	1.365216000	-2.372952000
H	-0.618786000	1.457103000	-2.349309000
H	-3.917486000	-1.174462000	1.048185000
H	-4.246016000	-0.335802000	-0.481432000
C	-4.506346000	-2.494381000	-0.542692000
C	-2.866380000	-2.711642000	-2.410767000
H	-2.629273000	-0.571310000	-2.419993000
H	-1.109130000	-1.440851000	-2.242669000
C	-2.412230000	-3.823430000	-0.213673000
H	-1.780838000	-2.479396000	1.371506000
H	-0.611214000	-2.643084000	0.047376000
H	-4.253858000	0.901776000	4.190001000
C	-2.236482000	0.468694000	4.816676000
H	-0.180935000	0.015311000	5.144920000
C	1.088132000	-1.585850000	3.233862000
C	1.653303000	0.800180000	3.194449000
H	0.243352000	4.860391000	0.003525000
C	-1.925004000	4.846316000	0.023034000
C	-0.486339000	4.232396000	-1.942564000
H	-4.026081000	4.376691000	-0.223591000
C	-2.979777000	3.971829000	-2.079262000
H	-1.566853000	3.345489000	-3.599295000
H	-5.565237000	-2.401708000	-0.275305000
C	-3.901587000	-3.725913000	0.143886000
C	-4.357075000	-2.619281000	-2.062857000
C	-2.248156000	-3.942371000	-1.734682000
H	-2.739937000	-2.780610000	-3.497280000
H	-1.965140000	-4.692940000	0.282576000
H	-2.451251000	0.537403000	5.878278000
H	2.112695000	-1.854356000	2.982746000
H	0.404949000	-2.336041000	2.833426000
H	0.997428000	-1.571056000	4.322948000
H	1.589244000	0.852463000	4.284471000
H	1.371095000	1.767265000	2.775907000
H	2.685107000	0.574418000	2.927396000

H	-2.018408000	5.876059000	-0.339907000
H	-1.989902000	4.890359000	1.117817000
H	0.484861000	3.832867000	-2.264498000
H	-0.549375000	5.252554000	-2.337888000
H	-3.804743000	3.385157000	-2.502559000
H	-3.087443000	4.991498000	-2.466331000
H	-4.423670000	-4.632193000	-0.183018000
H	-4.031473000	-3.662170000	1.231933000
H	-4.814203000	-1.758320000	-2.566724000
H	-4.886618000	-3.510285000	-2.418950000
H	-1.186468000	-4.033874000	-2.000061000
H	-2.739201000	-4.854179000	-2.093028000
C	3.888831000	-0.219006000	-0.125174000
C	4.359223000	1.148435000	0.219727000
C	3.246630000	-1.114652000	0.731314000
H	3.968377000	-0.500572000	-1.173300000
C	5.828071000	1.366894000	-0.201361000
H	4.234516000	1.350113000	1.288723000
H	3.746980000	1.874090000	-0.333727000
H	3.359911000	-0.866694000	1.786008000
C	6.705333000	0.288535000	0.414082000
H	6.150537000	2.364736000	0.112737000
H	5.907064000	1.334388000	-1.294860000
H	7.741243000	0.369613000	0.067997000
H	6.713406000	0.363910000	1.505830000
H	6.534229000	-1.299310000	-0.733906000
O	6.159343000	-0.995455000	0.102609000
C	3.259029000	-2.595248000	0.423074000
H	4.218182000	-3.002903000	0.763056000
H	2.467052000	-3.146811000	0.936898000
H	3.178381000	-2.787482000	-0.648449000

TS1$_{OH5}$
Sum of electronic and zero-point Energies = -2124.501896
Sum of electronic and thermal Free Energies = -2124.573438
Esolv = -2125.921395

C	1.593114000	-0.759016000	-1.383503000
C	1.822295000	-2.105394000	-1.683791000
H	1.713756000	-2.877831000	-0.927078000
Au	1.140761000	-0.285606000	0.553864000
C	1.746141000	0.212191000	-2.371086000
H	1.588251000	1.263938000	-2.155697000
C	2.112393000	-0.167223000	-3.667203000
H	2.222713000	0.594933000	-4.433507000
C	2.329322000	-1.507381000	-3.975226000
H	2.610119000	-1.797886000	-4.982729000
C	2.184451000	-2.474509000	-2.981706000
H	2.353475000	-3.523190000	-3.208879000
P	-1.220747000	0.178497000	0.248197000
C	-1.672696000	0.514390000	1.997887000
C	-1.528379000	1.809974000	-0.700835000
C	-2.204404000	-1.360913000	-0.296785000
C	-2.997154000	0.833613000	2.355131000
C	-0.713318000	0.469290000	3.017091000
C	-0.369739000	2.764462000	-0.322269000
C	-2.855007000	2.508140000	-0.314640000
C	-1.543176000	1.560992000	-2.225325000
C	-3.732380000	-1.123530000	-0.333853000
C	-1.744898000	-1.843288000	-1.691834000
C	-1.892161000	-2.466587000	0.740493000
H	-3.764055000	0.880610000	1.594844000
C	-3.354513000	1.105907000	3.669227000
C	-1.073865000	0.751157000	4.338512000
N	0.685104000	0.076536000	2.746982000
H	-0.386427000	2.956972000	0.759554000
H	0.600567000	2.305257000	-0.556862000
C	-0.507003000	4.094781000	-1.087818000
C	-2.993001000	3.838169000	-1.086820000
H	-2.872912000	2.730587000	0.756249000
H	-3.712871000	1.864694000	-0.541787000
C	-1.653742000	2.903584000	-2.974379000
H	-2.408847000	0.942882000	-2.487132000
H	-0.649608000	1.018940000	-2.548354000
H	-4.101642000	-0.808630000	0.645956000
H	-3.983171000	-0.341213000	-1.059681000
C	-4.451601000	-2.434448000	-0.717692000
C	-2.473377000	-3.150144000	-2.061986000
H	-1.957876000	-1.087767000	-2.452720000
H	-0.665655000	-2.012601000	-1.699286000
C	-2.623850000	-3.768253000	0.357115000
H	-2.207070000	-2.150956000	1.742931000
H	-0.807896000	-2.654701000	0.773940000
H	-4.384820000	1.348037000	3.908952000
C	-2.385220000	1.071234000	4.666493000
H	-0.336978000	0.714289000	5.133801000
C	0.944207000	-1.206297000	3.468401000
C	1.605727000	1.141993000	3.231207000
H	0.333169000	4.742729000	-0.809937000
C	-1.832788000	4.766984000	-0.707435000
C	-0.479336000	3.817781000	-2.597817000
H	-3.945397000	4.295332000	-0.794802000
C	-2.978053000	3.578854000	-2.597737000
H	-1.632981000	2.695650000	-4.050351000
H	-5.529000000	-2.233426000	-0.732483000
C	-4.137294000	-3.511607000	0.329370000
C	-3.985841000	-2.899809000	-2.102011000
C	-2.150819000	-4.234472000	-1.025832000
H	-2.120641000	-3.463555000	-3.051437000
H	-2.388700000	-4.528593000	1.111250000
H	-2.645080000	1.285718000	5.698142000
H	1.962325000	-1.547637000	3.283246000
H	0.241253000	-1.961486000	3.114806000
H	0.814243000	-1.078665000	4.546634000
H	1.505601000	1.299566000	4.308421000
H	1.379001000	2.075500000	2.713770000
H	2.636339000	0.849979000	3.028114000
H	-1.930694000	5.725073000	-1.230488000
H	-1.854513000	4.989658000	0.367151000
H	0.475073000	3.356600000	-2.886904000
H	-0.547506000	4.761436000	-3.151084000
H	-3.825082000	2.944562000	-2.888024000
H	-3.091281000	4.523779000	-3.141497000
H	-4.668082000	-4.438588000	0.084414000
H	-4.490821000	-3.198310000	1.320217000
H	-4.228840000	-2.146083000	-2.861829000
H	-4.514411000	-3.816659000	-2.387194000
H	-1.072333000	-4.441716000	-1.011597000
H	-2.647876000	-5.173599000	-1.294564000
C	3.231467000	-0.791557000	0.960044000
H	3.384935000	-1.759626000	0.482211000
H	3.249053000	-0.890074000	2.045191000
C	4.185509000	0.204866000	0.514818000
C	4.698915000	0.288736000	-0.890773000
H	4.163410000	1.160505000	1.043934000
C	6.796949000	-0.268664000	0.170957000
C	6.185002000	0.664004000	-0.856953000
H	4.539886000	-0.660457000	-1.409986000
H	4.122485000	1.051690000	-1.425268000
H	7.754619000	0.065628000	0.575153000
H	6.895506000	-1.291704000	-0.195853000

H	6.651439000	0.541569000	-1.837671000
H	6.318718000	1.709573000	-0.556723000
H	6.073660000	0.276660000	1.967164000
O	5.820948000	-0.338223000	1.261200000

TS1$_{O5}$

Sum of electronic and zero-point Energies = -2124,157425
Sum of electronic and thermal Free Energies = -2124,227109
Esolv = -2125.4341403

C	1.208376000	-1.264719000	-1.643714000
C	0.583076000	-2.447345000	-2.034651000
H	0.009058000	-3.044191000	-1.333150000
Au	1.201177000	-0.716611000	0.335333000
C	1.973135000	-0.527047000	-2.542235000
H	2.511289000	0.345783000	-2.173224000
C	2.092821000	-0.975772000	-3.860156000
H	2.693161000	-0.403702000	-4.562533000
C	1.462616000	-2.149747000	-4.270733000
H	1.561722000	-2.493414000	-5.296050000
C	0.717710000	-2.888578000	-3.356071000
H	0.237662000	-3.815137000	-3.658595000
P	-0.939945000	0.333754000	0.302825000
C	-1.032460000	0.839487000	2.072185000
C	-0.851733000	1.970898000	-0.689322000
C	-2.443974000	-0.798475000	-0.016763000
C	-2.100694000	1.622763000	2.548372000
C	-0.011729000	0.506342000	2.972111000
C	0.346249000	2.754251000	-0.096231000
C	-2.131073000	2.832630000	-0.584675000
C	-0.579281000	1.695623000	-2.183975000
C	-2.622126000	-1.037188000	-1.532357000
C	-2.159840000	-2.136953000	0.704349000
C	-3.775091000	-0.244069000	0.547777000
H	-2.911625000	1.888780000	1.885524000
C	-2.147484000	2.075217000	3.860716000
C	-0.064421000	0.959723000	4.293929000
N	1.091960000	-0.376810000	2.575752000
H	0.162076000	2.974825000	0.962877000
H	1.270488000	2.160114000	-0.163130000
C	0.525181000	4.080097000	-0.861022000
C	-1.928875000	4.158883000	-1.349867000
H	-2.355417000	3.077774000	0.456539000
H	-2.993016000	2.299736000	-1.003750000
C	-0.378156000	3.023551000	-2.938643000
H	-1.412804000	1.151474000	-2.637793000
H	0.310427000	1.076989000	-2.298939000
H	-2.891185000	-0.095509000	-2.022965000
H	-1.690333000	-1.386450000	-1.986921000
C	-3.750084000	-2.056774000	-1.776134000
C	-3.298387000	-3.139024000	0.438775000
H	-1.203756000	-2.557161000	0.370590000
H	-2.076933000	-1.958008000	1.784996000
C	-4.919252000	-1.244262000	0.275765000
H	-4.020707000	0.722480000	0.093632000
H	-3.697713000	-0.098532000	1.629492000
H	-2.983644000	2.682729000	4.191837000
C	-1.119101000	1.746764000	4.737613000
H	0.720039000	0.695777000	4.994400000
C	0.869100000	-1.697239000	3.230170000
C	2.400697000	0.177579000	3.003611000
H	1.373357000	4.615291000	-0.417708000
C	-0.748924000	4.926526000	-0.737989000
C	0.810984000	3.785156000	-2.339083000
H	-2.849305000	4.747107000	-1.248902000
C	-1.650390000	3.871843000	-2.829529000
H	-0.176902000	2.785439000	-3.990095000
H	-3.833807000	-2.221906000	-2.856798000
C	-5.065986000	-1.487121000	-1.231268000
C	-3.424816000	-3.381174000	-1.071841000
C	-4.615025000	-2.570171000	0.984310000
H	-3.057740000	-4.079538000	0.949277000
H	-5.844184000	-0.812359000	0.676695000
H	-1.137213000	2.093229000	5.766107000
H	1.692255000	-2.367043000	2.989550000
H	-0.063855000	-2.130605000	2.869636000
H	0.814573000	-1.578780000	4.317759000
H	2.492231000	0.199748000	4.094216000
H	2.511439000	1.180909000	2.596350000
H	3.190829000	-0.452683000	2.599253000
H	-0.618844000	5.884602000	-1.255948000
H	-0.951560000	5.160466000	0.315624000
H	1.729939000	3.194054000	-2.433739000
H	0.972405000	4.721898000	-2.886763000
H	-2.500822000	3.346531000	-3.283999000
H	-1.529055000	4.813765000	-3.378196000
H	-5.890736000	-2.185070000	-1.419477000
H	-5.317737000	-0.551888000	-1.747913000
H	-2.494552000	-3.806197000	-1.470472000
H	-4.215610000	-4.115226000	-1.267940000
H	-4.544165000	-2.416996000	2.069156000
H	-5.431597000	-3.282730000	0.817025000
C	2.882134000	-2.580098000	0.542844000
H	2.228621000	-3.245137000	-0.013020000
H	3.012541000	-2.793909000	1.600212000
C	3.827025000	-1.830770000	-0.108669000
C	5.044119000	-1.239035000	0.504777000
H	3.760186000	-1.751418000	-1.190698000
C	4.233501000	1.134572000	0.327056000
C	5.381456000	0.166344000	0.006542000
H	4.990272000	-1.276537000	1.600276000
H	5.861916000	-1.922875000	0.221831000
H	4.494214000	2.132066000	-0.075419000
H	4.219694000	1.270530000	1.429130000
H	6.326181000	0.499836000	0.453420000
H	5.528941000	0.137797000	-1.080367000
O	3.043261000	0.678848000	-0.202587000

TS1$_{OH5Z}$

Sum of electronic and zero-point Energies = -2163.772596
Sum of electronic and thermal Free Energies = -2163.844738
Esolv = -2165.2304433

Au	1.069151000	-0.209617000	0.513813000
P	-1.333873000	0.055105000	0.233334000
C	-1.844665000	-0.052344000	1.994066000
C	-1.843563000	1.801959000	-0.355916000
C	-2.076812000	-1.473539000	-0.639478000
C	-3.204261000	0.017261000	2.356732000
C	-0.899497000	-0.188355000	3.018235000
C	-0.902691000	2.786956000	0.380192000
C	-3.299986000	2.180630000	0.002276000
C	-1.668675000	1.945651000	-1.883774000
C	-3.610027000	-1.596552000	-0.481569000
C	-1.738381000	-1.472248000	-2.146779000
C	-1.416277000	-2.702493000	0.032742000
H	-3.961172000	0.122594000	1.592014000
C	-3.610672000	-0.040388000	3.683502000
C	-1.311003000	-0.234817000	4.354164000
N	0.548445000	-0.285236000	2.733876000

H	-1.047944000	2.699334000	1.465533000
H	0.148061000	2.544320000	0.167703000
C	-1.196109000	4.233265000	-0.063954000
C	-3.591491000	3.627844000	-0.451820000
H	-3.454968000	2.127874000	1.083462000
H	-4.008811000	1.495260000	-0.477419000
C	-1.938035000	3.402853000	-2.308305000
H	-2.385970000	1.299763000	-2.399002000
H	-0.667781000	1.636536000	-2.199002000
H	-3.885857000	-1.640059000	0.575472000
H	-4.115358000	-0.733821000	-0.931813000
C	-4.100696000	-2.896381000	-1.155519000
C	-2.221501000	-2.784322000	-2.795524000
H	-2.234721000	-0.635661000	-2.645170000
H	-0.663742000	-1.349191000	-2.304217000
C	-1.917212000	-4.000951000	-0.629440000
H	-1.653073000	-2.720194000	1.104704000
H	-0.321284000	-2.644950000	-0.065107000
H	-4.666679000	0.014198000	3.927458000
C	-2.657206000	-0.162052000	4.689011000
H	-0.585370000	-0.329457000	5.154928000
C	1.056973000	-1.564903000	3.305864000
C	1.235300000	0.877152000	3.368900000
H	-0.510384000	4.903699000	0.468044000
C	-2.648017000	4.587936000	0.284272000
C	-0.979867000	4.353823000	-1.578692000
H	-4.631297000	3.856076000	-0.191003000
C	-3.389210000	3.761670000	-1.965465000
H	-1.781515000	3.474918000	-3.390819000
H	-5.188354000	-2.947998000	-1.030348000
C	-3.440684000	-4.100304000	-0.470244000
C	-3.744540000	-2.887213000	-2.646598000
C	-1.549658000	-3.986275000	-2.119101000
H	-1.951669000	-2.755610000	-3.857603000
H	-1.434202000	-4.850034000	-0.131126000
H	-2.955034000	-0.201635000	5.731882000
H	2.126752000	-1.655999000	3.122534000
H	0.533079000	-2.399422000	2.837668000
H	0.895880000	-1.607644000	4.385984000
H	1.096624000	0.873094000	4.453691000
H	0.823882000	1.802059000	2.962622000
H	2.305990000	0.837954000	3.162792000
H	-2.860774000	5.622510000	-0.008134000
H	-2.807152000	4.527010000	1.368602000
H	0.062134000	4.121697000	-1.837388000
H	-1.158392000	5.386246000	-1.900362000
H	-4.081857000	3.104292000	-2.506037000
H	-3.613380000	4.786120000	-2.284306000
H	-3.802578000	-5.032774000	-0.918146000
H	-3.713115000	-4.134036000	0.592528000
H	-4.234918000	-2.046649000	-3.153759000
H	-4.112326000	-3.802074000	-3.125305000
H	-0.459324000	-3.936903000	-2.243711000
H	-1.877053000	-4.917778000	-2.594880000
C	4.112683000	0.280994000	0.292833000
H	3.902084000	1.325771000	0.520536000
C	3.197384000	-0.677085000	0.915828000
C	4.840825000	0.072309000	-0.998809000
H	4.973298000	-0.992191000	-1.209156000
H	4.240166000	0.498684000	-1.809167000
C	6.790186000	0.320575000	0.419432000
H	7.536264000	1.000250000	0.835405000
H	7.197697000	-0.691712000	0.351742000
C	6.193218000	0.785928000	-0.895460000
H	6.059722000	1.872728000	-0.888264000
H	6.847716000	0.538851000	-1.734504000
C	3.476877000	-2.153542000	0.665399000
H	4.484214000	-2.445690000	0.992674000
H	2.776974000	-2.792310000	1.209566000
H	3.397139000	-2.411954000	-0.392601000
H	3.166696000	-0.444415000	1.984591000
C	1.585271000	-0.051148000	-1.455972000
C	1.793374000	1.212307000	-2.014805000
C	1.794716000	-1.196455000	-2.226275000
C	2.196467000	1.325092000	-3.349783000
H	1.648106000	2.116279000	-1.431124000
C	2.198772000	-1.074675000	-3.559244000
H	1.631131000	-2.187814000	-1.813980000
C	2.400081000	0.183621000	-4.123320000
H	2.347237000	2.311501000	-3.779329000
H	2.349571000	-1.970991000	-4.154109000
H	2.711384000	0.274201000	-5.159290000
H	5.684693000	-0.444124000	1.935738000
O	5.644654000	0.313554000	1.333043000

$TS1_{OH5E}$

Sum of electronic and zero-point Energies = -2163.761866

Sum of electronic and thermal Free Energies = -2163.834185

Esolv = -2165.21946072

Au	1.091925000	-0.514975000	0.358927000
P	-1.245667000	0.194471000	0.296680000
C	-1.624987000	0.121672000	2.090497000
C	-1.418273000	2.034307000	-0.203348000
C	-2.367983000	-1.105631000	-0.544061000
C	-2.906596000	0.470449000	2.576007000
C	-0.662043000	-0.274651000	3.018230000
C	-0.194954000	2.754585000	0.401954000
C	-2.693924000	2.716577000	0.359284000
C	-1.437720000	2.197866000	-1.738930000
C	-3.873421000	-0.886291000	-0.267814000
C	-2.149807000	-1.124819000	-2.074941000
C	-1.951536000	-2.470682000	0.048929000
H	-3.679857000	0.780258000	1.887211000
C	-3.212292000	0.435002000	3.930493000
C	-0.973503000	-0.309831000	4.382411000
N	0.681107000	-0.731951000	2.596158000
H	-0.195139000	2.663974000	1.496789000
H	0.738561000	2.301728000	0.029402000
C	-0.211406000	4.247084000	0.007757000
C	-2.709853000	4.199015000	-0.037382000
H	-2.700891000	2.655231000	1.451105000
H	-3.593806000	2.219560000	-0.013402000
C	-1.422313000	3.694330000	-2.108532000
H	-2.346612000	1.746351000	-2.139443000
H	-0.596384000	1.677953000	-2.199295000
H	-4.078816000	-0.896109000	0.808119000
H	-4.200311000	0.081529000	-0.669766000
C	-4.699787000	-2.014477000	-0.917616000
C	-2.970670000	-2.276330000	-2.701431000
H	-2.476577000	-0.182570000	-2.525231000
H	-1.092163000	-1.257630000	-2.313949000
C	-2.786221000	-3.604823000	-0.590741000
H	-2.113726000	-2.464612000	1.136796000
H	-0.881996000	-2.658333000	-0.129392000
H	-4.199832000	0.714134000	4.270883000
C	-2.233620000	0.047368000	4.840115000
H	-0.229519000	-0.637098000	5.118385000
C	0.764240000	-2.202463000	2.914359000
C	1.742968000	0.004267000	3.347072000
H	0.669770000	4.732960000	0.444510000

C	-1.489353000	4.902881000	0.569293000
C	-0.189976000	4.377432000	-1.521561000
H	-3.629715000	4.644640000	0.379212000
C	-2.698685000	4.346565000	-1.563155000
H	-1.409977000	3.773833000	-3.201561000
H	-5.758355000	-1.832101000	-0.709181000
C	-4.276323000	-3.355791000	-0.311324000
C	-4.461948000	-2.041140000	-2.433817000
C	-2.535394000	-3.618054000	-2.105990000
H	-2.776304000	-2.269123000	-3.781896000
H	-2.472870000	-4.556033000	-0.140265000
H	-2.453969000	0.006569000	5.902211000
H	1.724292000	-2.596295000	2.585032000
H	-0.049617000	-2.722865000	2.387790000
H	0.653920000	-2.372919000	3.991520000
H	1.583147000	-0.051018000	4.430495000
H	1.739981000	1.062007000	3.043589000
H	2.706854000	-0.443375000	3.130448000
H	-1.506789000	5.969773000	0.319180000
H	-1.511411000	4.834391000	1.664584000
H	0.731691000	3.937103000	-1.926091000
H	-0.179967000	5.435837000	-1.806141000
H	-3.585602000	3.887054000	-1.996962000
H	-2.731959000	5.407187000	-1.837761000
H	-4.868620000	-4.171262000	-0.741755000
H	-4.463332000	-3.366624000	0.770268000
H	-4.785434000	-1.096501000	-2.892895000
H	-5.058540000	-2.837305000	-2.893848000
H	-1.474663000	-3.811166000	-2.314171000
H	-3.095890000	-4.439503000	-2.566782000
C	3.257331000	-1.175490000	0.253259000
C	1.366805000	-0.456636000	-1.670610000
C	1.710678000	0.715994000	-2.341852000
C	1.319648000	-1.666622000	-2.358590000
C	1.990077000	0.680768000	-3.713053000
H	1.751130000	1.675983000	-1.827934000
C	1.599016000	-1.701033000	-3.727670000
H	1.084304000	-2.595138000	-1.855183000
C	1.926796000	-0.528435000	-4.407740000
H	2.240498000	1.601052000	-4.234739000
H	1.556552000	-2.643939000	-4.264336000
H	2.143715000	-0.551462000	-5.471018000
C	3.484190000	-2.582323000	0.791933000
H	3.630543000	-2.610494000	1.885719000
H	2.668592000	-3.279033000	0.547094000
H	4.398257000	-3.006958000	0.350739000
H	3.359004000	-1.169522000	-0.832039000
C	4.133738000	-0.158994000	0.818077000
C	4.320952000	1.206502000	0.183746000
H	4.254334000	-0.183109000	1.902481000
C	6.529275000	0.312224000	-0.247780000
H	6.295224000	-1.078165000	1.233636000
C	5.411315000	1.095720000	-0.893047000
H	4.610089000	1.935824000	0.940065000
H	3.381600000	1.540274000	-0.263493000
H	7.091855000	0.897277000	0.473184000
H	7.201713000	-0.173974000	-0.965480000
H	5.753065000	2.090086000	-1.215880000
H	5.025249000	0.578023000	-1.779339000
O	5.804160000	-0.755414000	0.454455000

TS1’$_{OH5E}$

Sum of electronic and zero-point Energies = -2163.775527

Sum of electronic and thermal Free Energies = -2163.848128

Esolv = -2165.23353

C	1.456772000	-0.493467000	-1.571745000
C	1.460684000	-1.744913000	-2.191395000
H	1.190717000	-2.643703000	-1.645165000
Au	1.080301000	-0.367040000	0.439299000
C	1.796325000	0.645993000	-2.303625000
H	1.793858000	1.630325000	-1.844955000
C	2.138306000	0.530059000	-3.654824000
H	2.396390000	1.422747000	-4.217531000
C	2.143049000	-0.716541000	-4.275980000
H	2.408325000	-0.803293000	-5.324905000
C	1.801260000	-1.851028000	-3.543307000
H	1.794458000	-2.827870000	-4.018249000
P	-1.278164000	0.182367000	0.263011000
C	-1.682387000	0.279910000	2.051779000
C	-1.577222000	1.932890000	-0.446469000
C	-2.276982000	-1.289856000	-0.434979000
C	-2.993645000	0.566176000	2.480300000
C	-0.702743000	0.074910000	3.031217000
C	-0.453100000	2.824243000	0.136026000
C	-2.934428000	2.548129000	-0.030005000
C	-1.502969000	1.927968000	-1.989241000
C	-3.797724000	-1.184589000	-0.173965000
C	-2.051743000	-1.450581000	-1.954963000
C	-1.740779000	-2.543196000	0.299686000
H	-3.774696000	0.733386000	1.751716000
C	-3.320803000	0.646099000	3.827672000
C	-1.034156000	0.161587000	4.387204000
N	0.699214000	-0.235102000	2.677615000
H	-0.523229000	2.843301000	1.232209000
H	0.533507000	2.414556000	-0.124000000
C	-0.574309000	4.258359000	-0.414534000
C	-3.053438000	3.981950000	-0.591607000
H	-3.012658000	2.603507000	1.059382000
H	-3.766684000	1.938157000	-0.401012000
C	-1.595568000	3.370734000	-2.523588000
H	-2.342871000	1.356554000	-2.395561000
H	-0.584435000	1.449612000	-2.341310000
H	-4.000534000	-1.108188000	0.897599000
H	-4.213700000	-0.296945000	-0.665517000
C	-4.505486000	-2.451412000	-0.702399000
C	-2.751695000	-2.729116000	-2.456859000
H	-2.468223000	-0.594277000	-2.491573000
H	-0.985112000	-1.490744000	-2.189388000
C	-2.457771000	-3.806101000	-0.216595000
H	-1.900977000	-2.444979000	1.381449000
H	-0.657947000	-2.646003000	0.131623000
H	-4.341350000	0.867165000	4.123055000
C	-2.334089000	0.444280000	4.787211000
H	-0.281099000	0.008257000	5.152857000
C	1.063887000	-1.547899000	3.285386000
C	1.573626000	0.845701000	3.216483000
H	0.237362000	4.861517000	0.010193000
C	-1.930873000	4.848251000	-0.005987000
C	-0.460323000	4.225816000	-1.944902000
H	-4.027730000	4.379885000	-0.285550000
C	-2.950967000	3.965119000	-2.121121000
H	-1.513753000	3.333570000	-3.615976000
H	-5.578246000	-2.340425000	-0.506901000
C	-3.965254000	-3.679030000	0.043103000
C	-4.259977000	-2.607096000	-2.207526000
C	-2.199849000	-3.957328000	-1.721527000

H	-2.557243000	-2.820144000	-3.531770000
H	-2.058295000	-4.673306000	0.322740000
H	-2.570204000	0.505539000	5.844732000
H	2.096463000	-1.795182000	3.043144000
H	0.400697000	-2.319963000	2.892721000
H	0.967052000	-1.519428000	4.373785000
H	1.488438000	0.917254000	4.304286000
H	1.279661000	1.798708000	2.774559000
H	2.614378000	0.635683000	2.969597000
H	-2.017746000	5.876607000	-0.374778000
H	-2.013401000	4.896650000	1.087479000
H	0.515923000	3.824052000	-2.248993000
H	-0.516304000	5.244470000	-2.345448000
H	-3.768823000	3.376411000	-2.555340000
H	-3.052119000	4.983100000	-2.514630000
H	-4.480217000	-4.583588000	-0.299840000
H	-4.162533000	-3.592308000	1.119406000
H	-4.668860000	-1.748149000	-2.754572000
H	-4.780465000	-3.496035000	-2.582003000
H	-1.124856000	-4.070391000	-1.916389000
H	-2.682874000	-4.867737000	-2.094412000
C	3.177360000	-1.051604000	0.758993000
H	3.335004000	-0.779172000	1.806282000
C	4.037972000	-0.264210000	-0.101944000
H	4.029178000	-0.539143000	-1.154356000
C	5.836090000	1.418316000	-0.253772000
C	6.635505000	0.275989000	0.338968000
H	5.915545000	-1.573968000	0.742483000
H	5.919891000	1.408034000	-1.345138000
H	6.202532000	2.384974000	0.099334000
H	6.772644000	0.384795000	1.419209000
H	7.601637000	0.118085000	-0.145591000
C	4.383767000	1.169012000	0.174501000
H	4.241941000	1.405530000	1.234721000
H	3.709638000	1.807037000	-0.406920000
O	5.803308000	-0.888429000	0.067922000
C	3.227308000	-2.561548000	0.540841000
H	3.218941000	-2.813715000	-0.521581000
H	4.137636000	-2.997315000	0.973012000
H	2.391316000	-3.081569000	1.016243000

TS1$_{OH6}$
Sum of electronic and zero-point Energies = -2124.488161
Sum of electronic and thermal Free Energies = -2124.558650
Esolv = -2125.9126147

C	1.562876000	0.219616000	-1.565298000
C	1.749942000	-0.894738000	-2.383460000
H	1.618726000	-1.904005000	-2.004898000
Au	1.197662000	0.024540000	0.443986000
C	1.744722000	1.506539000	-2.073460000
H	1.612091000	2.385134000	-1.448874000
C	2.110869000	1.674305000	-3.412994000
H	2.248049000	2.678284000	-3.804138000
C	2.295473000	0.566811000	-4.237123000
H	2.578676000	0.701545000	-5.276261000
C	2.117567000	-0.714589000	-3.720903000
H	2.263808000	-1.585508000	-4.353302000
P	-1.212323000	-0.054530000	0.259935000
C	-1.613984000	-0.186633000	2.046788000
C	-1.946523000	1.600789000	-0.348892000
C	-1.771943000	-1.699228000	-0.546244000
C	-2.954747000	-0.286361000	2.468014000
C	-0.614573000	-0.191814000	3.027812000
C	-1.194838000	2.707217000	0.430007000
C	-3.459196000	1.736097000	-0.049429000
C	-1.731168000	1.792243000	-1.866392000
C	-3.208267000	-2.110739000	-0.142185000
C	-1.705414000	-1.620446000	-2.086614000
C	-0.799178000	-2.785208000	-0.025698000
H	-3.751841000	-0.289048000	1.737638000
C	-3.290646000	-0.388360000	3.811906000
C	-0.956579000	-0.292758000	4.379657000
N	0.815307000	-0.060469000	2.679045000
H	-1.357037000	2.584686000	1.509072000
H	-0.112640000	2.636012000	0.244350000
C	-1.697807000	4.095916000	-0.011650000
C	-3.953270000	3.128440000	-0.502021000
H	-3.650591000	1.644051000	1.023053000
H	-4.027817000	0.955312000	-0.568621000
C	-2.213251000	3.194144000	-2.289571000
H	-2.302851000	1.046684000	-2.424779000
H	-0.680003000	1.660381000	-2.133304000
H	-3.287860000	-2.213885000	0.943475000
H	-3.934994000	-1.357465000	-0.469303000
C	-3.555673000	-3.475696000	-0.777719000
C	-2.028801000	-3.000667000	-2.693774000
H	-2.446211000	-0.903099000	-2.451732000
H	-0.724026000	-1.275992000	-2.424799000
C	-1.151568000	-4.151087000	-0.647860000
H	-0.860972000	-2.850927000	1.069141000
H	0.237586000	-2.525878000	-0.284480000
H	-4.333314000	-0.464779000	4.102750000
C	-2.285345000	-0.392630000	4.773304000
H	-0.189961000	-0.289239000	5.147102000
C	1.563381000	-1.222546000	3.238278000
C	1.319817000	1.218473000	3.265193000
H	-1.147317000	4.856307000	0.554888000
C	-3.199878000	4.210295000	0.282916000
C	-1.446342000	4.273691000	-1.514872000
H	-5.025226000	3.187138000	-0.281294000
C	-3.715135000	3.314764000	-2.004739000
H	-2.026868000	3.303316000	-3.364095000
H	-4.579485000	-3.728320000	-0.478999000
C	-2.582485000	-4.538408000	-0.251540000
C	-3.459658000	-3.392259000	-2.305226000
C	-1.041834000	-4.055353000	-2.175832000
H	-1.946866000	-2.917184000	-3.783479000
H	-0.443475000	-4.896097000	-0.265765000
H	-2.529288000	-0.470698000	5.827926000
H	2.620905000	-1.135274000	2.988797000
H	1.159516000	-2.145389000	2.819152000
H	1.480768000	-1.262890000	4.327359000
H	1.227855000	1.210200000	4.354794000
H	0.734815000	2.048448000	2.867421000
H	2.369174000	1.363183000	3.009929000
H	-3.561552000	5.203655000	-0.005509000
H	-3.388488000	4.106250000	1.359213000
H	-0.371828000	4.213403000	-1.733695000
H	-1.774488000	5.269638000	-1.833215000
H	-4.274142000	2.565225000	-2.578987000
H	-4.085216000	4.295947000	-2.323208000
H	-2.833606000	-5.518974000	-0.671310000
H	-2.666728000	-4.630923000	0.838915000
H	-4.175766000	-2.658916000	-2.696895000
H	-3.723649000	-4.358517000	-2.749941000
H	-0.015539000	-3.800552000	-2.472500000
H	-1.262962000	-5.029086000	-2.627320000
C	3.678706000	-1.106675000	0.302531000
H	3.761226000	-1.277846000	-0.765374000
H	3.501047000	-1.988312000	0.913658000

C	3.495651000	0.184055000	0.798021000
C	4.225974000	1.323332000	0.112688000
H	3.505026000	0.257636000	1.885159000
C	6.335565000	-0.092005000	-0.275032000
C	5.742514000	1.174011000	0.338303000
H	4.020571000	1.322486000	-0.961531000
H	3.889998000	2.287430000	0.507308000
H	7.425039000	-0.108375000	-0.168884000
H	6.108129000	-0.154117000	-1.343179000
H	6.252049000	2.031311000	-0.115894000
H	5.978101000	1.216046000	1.410185000
H	6.159188000	-1.423315000	1.181787000
O	5.801621000	-1.312222000	0.289239000

TS1$_{OH6Z}$
Sum of electronic and zero-point Energies = -2163.766037
Sum of electronic and thermal Free Energies = -2163.837968
Esolv = -2165.2272322

C	1.399296000	0.145210000	-1.692692000
C	1.283264000	-0.948103000	-2.550273000
H	1.025543000	-1.933740000	-2.175437000
Au	1.166349000	-0.040263000	0.330483000
C	1.722007000	1.403654000	-2.205695000
H	1.803281000	2.272019000	-1.558465000
C	1.938424000	1.559931000	-3.577574000
H	2.188604000	2.542214000	-3.968021000
C	1.828786000	0.468667000	-4.437392000
H	1.994553000	0.594351000	-5.502735000
C	1.500591000	-0.782170000	-3.922332000
H	1.407955000	-1.639424000	-4.583043000
P	-1.267002000	-0.007123000	0.327411000
C	-1.555376000	-0.085467000	2.140082000
C	-1.964595000	1.667261000	-0.268284000
C	-2.004554000	-1.620586000	-0.394164000
C	-2.865108000	-0.087015000	2.659426000
C	-0.491661000	-0.154374000	3.047623000
C	-1.186785000	2.752889000	0.514335000
C	-3.475199000	1.836185000	0.019958000
C	-1.730421000	1.865443000	-1.782838000
C	-3.395801000	-1.976010000	0.185387000
C	-2.135822000	-1.531702000	-1.930313000
C	-1.028167000	-2.755602000	0.000184000
H	-3.711530000	-0.044170000	1.988636000
C	-3.110016000	-0.154249000	4.024781000
C	-0.741302000	-0.226733000	4.421860000
N	0.915840000	-0.121639000	2.598743000
H	-1.346616000	2.630810000	1.593443000
H	-0.107177000	2.655383000	0.322723000
C	-1.657666000	4.154712000	0.079023000
C	-3.934343000	3.242193000	-0.424777000
H	-3.679098000	1.740244000	1.089609000
H	-4.056593000	1.072416000	-0.510192000
C	-2.185314000	3.276727000	-2.204065000
H	-2.296717000	1.128094000	-2.357142000
H	-0.676189000	1.727942000	-2.032038000
H	-3.343694000	-2.081252000	1.272779000
H	-4.125187000	-1.190735000	-0.046322000
C	-3.881052000	-3.318882000	-0.404314000
C	-2.590799000	-2.891127000	-2.496889000
H	-2.889236000	-0.780574000	-2.189304000
H	-1.195285000	-1.221406000	-2.394348000
C	-1.512827000	-4.097918000	-0.581209000
H	-0.966714000	-2.828371000	1.094905000
H	-0.016418000	-2.541318000	-0.371150000
H	-4.131860000	-0.153059000	4.390086000
C	-2.041067000	-0.227911000	4.911827000
H	0.077288000	-0.277211000	5.131860000
C	1.611309000	-1.341320000	3.097345000
C	1.544057000	1.112081000	3.158553000
H	-1.092759000	4.899903000	0.651691000
C	-3.157963000	4.301140000	0.369456000
C	-1.398322000	4.336437000	-1.422237000
H	-5.005516000	3.326640000	-0.208405000
C	-3.685353000	3.429735000	-1.925503000
H	-1.991821000	3.385875000	-3.277445000
H	-4.869099000	-3.529570000	0.020817000
C	-2.898323000	-4.429024000	-0.011072000
C	-3.976189000	-3.227032000	-1.931497000
C	-1.591494000	-3.990202000	-2.110620000
H	-2.643778000	-2.803091000	-3.588098000
H	-0.795669000	-4.877093000	-0.296139000
H	-2.212872000	-0.284110000	5.981949000
H	2.650822000	-1.334627000	2.768387000
H	1.109909000	-2.226465000	2.703272000
H	1.605393000	-1.387119000	4.189605000
H	1.527157000	1.098971000	4.251807000
H	0.989987000	1.983440000	2.807158000
H	2.581378000	1.190830000	2.833870000
H	-3.495975000	5.304582000	0.086966000
H	-3.352259000	4.193899000	1.444476000
H	-0.324645000	4.252797000	-1.637989000
H	-1.704191000	5.341009000	-1.735987000
H	-4.258448000	2.694774000	-2.504814000
H	-4.031902000	4.420208000	-2.241984000
H	-3.243517000	-5.392920000	-0.401834000
H	-2.849023000	-4.530106000	1.080791000
H	-4.704221000	-2.461051000	-2.226938000
H	-4.334945000	-4.177737000	-2.342240000
H	-0.600379000	-3.774917000	-2.532594000
H	-1.907436000	-4.949521000	-2.536015000
C	3.408283000	0.020445000	0.530054000
H	3.431079000	0.029485000	1.622241000
C	3.953663000	-1.229984000	0.070928000
C	4.077592000	1.286186000	-0.004152000
H	4.082989000	1.296881000	-1.097310000
H	3.512714000	2.170311000	0.309546000
H	5.956299000	-1.068489000	1.591218000
C	6.407440000	0.234237000	0.157792000
H	7.417877000	0.342571000	0.563465000
H	6.495098000	0.118029000	-0.924667000
C	5.513806000	1.407749000	0.531231000
H	5.500864000	1.526759000	1.623754000
H	5.977420000	2.318435000	0.135479000
O	5.878214000	-1.036980000	0.625776000
C	4.239635000	-1.602196000	-1.335415000
H	5.062745000	-2.318888000	-1.378405000
H	3.352870000	-2.115386000	-1.726288000
H	4.435243000	-0.753029000	-1.989391000
H	3.804795000	-2.088206000	0.728233000

TS1$_{OH6E}$
Sum of electronic and zero-point Energies = -2163.774393
Sum of electronic and thermal Free Energies = -2163.846374
Esolv = -2165.234833

C	1.452500000	-0.115178000	-1.624337000
C	1.472285000	-1.318706000	-2.330396000
H	1.233544000	-2.260989000	-1.846709000
Au	1.129355000	-0.096122000	0.404864000

C	1.769078000	1.080986000	-2.269531000
H	1.750219000	2.029485000	-1.741196000
C	2.103600000	1.068121000	-3.627841000
H	2.341363000	2.004019000	-4.125256000
C	2.127257000	-0.130204000	-4.336876000
H	2.389359000	-0.136313000	-5.390144000
C	1.811483000	-1.321260000	-3.686984000
H	1.827272000	-2.262138000	-4.229520000
P	-1.281213000	0.061279000	0.273184000
C	-1.653099000	0.112126000	2.069779000
C	-1.827406000	1.748625000	-0.444136000
C	-2.060413000	-1.554159000	-0.395643000
C	-2.984702000	0.211518000	2.519869000
C	-0.636736000	0.054431000	3.030910000
C	-0.909625000	2.805250000	0.217697000
C	-3.295730000	2.099438000	-0.103946000
C	-1.657926000	1.797334000	-1.978606000
C	-3.528610000	-1.751326000	0.054480000
C	-2.017559000	-1.602078000	-1.938438000
C	-1.220866000	-2.711420000	0.197914000
H	-3.794702000	0.254578000	1.805211000
C	-3.295668000	0.253609000	3.872710000
C	-0.954058000	0.094524000	4.392283000
N	0.787979000	-0.049010000	2.651507000
H	-1.035997000	2.784657000	1.308333000
H	0.145848000	2.583587000	0.000340000
C	-1.254735000	4.210079000	-0.315245000
C	-3.632579000	3.505557000	-0.648314000
H	-3.447942000	2.113253000	0.978434000
H	-3.980762000	1.361430000	-0.538517000
C	-1.979633000	3.214268000	-2.494944000
H	-2.344116000	1.092490000	-2.455053000
H	-0.644843000	1.511096000	-2.271313000
H	-3.599040000	-1.761076000	1.145621000
H	-4.162215000	-0.939641000	-0.322728000
C	-4.055403000	-3.106215000	-0.469277000
C	-2.521175000	-2.972771000	-2.432981000
H	-2.671662000	-0.827912000	-2.349781000
H	-1.009220000	-1.408768000	-2.314383000
C	-1.751206000	-4.065318000	-0.313794000
H	-1.272556000	-2.686184000	1.294914000
H	-0.164244000	-2.602901000	-0.085698000
H	-4.331792000	0.331118000	4.185818000
C	-2.273747000	0.194144000	4.814647000
H	-0.174069000	0.049270000	5.144755000
C	1.339901000	-1.311207000	3.224318000
C	1.512208000	1.133772000	3.204161000
H	-0.588895000	4.934110000	0.169319000
C	-2.715976000	4.537979000	0.020968000
C	-1.050246000	4.241585000	-1.835713000
H	-4.677941000	3.717000000	-0.395722000
C	-3.440498000	3.547124000	-2.168302000
H	-1.829316000	3.218548000	-3.580621000
H	-5.096258000	-3.205622000	-0.140595000
C	-3.210896000	-4.239811000	0.126555000
C	-3.981288000	-3.152107000	-1.999538000
C	-1.661376000	-4.099842000	-1.845571000
H	-2.452102000	-2.983158000	-3.526741000
H	-1.133665000	-4.862285000	0.117572000
H	-2.497774000	0.224522000	5.876123000
H	2.391891000	-1.409400000	2.958433000
H	0.782827000	-2.160049000	2.825670000
H	1.262834000	-1.316559000	4.314835000
H	1.430931000	1.170553000	4.293590000
H	1.080378000	2.044694000	2.787280000
H	2.568291000	1.078694000	2.944502000
H	-2.963229000	5.545061000	-0.333297000
H	-2.868017000	4.539739000	1.108009000
H	-0.002520000	4.028025000	-2.086374000
H	-1.265307000	5.244788000	-2.220854000
H	-4.113744000	2.834442000	-2.661169000
H	-3.698938000	4.540993000	-2.551194000
H	-3.590249000	-5.209927000	-0.213720000
H	-3.283541000	-4.238994000	1.221758000
H	-4.608777000	-2.366868000	-2.439585000
H	-4.372000000	-4.108838000	-2.364470000
H	-0.617675000	-3.997091000	-2.172056000
H	-2.009466000	-5.069970000	-2.217912000
C	3.408081000	-0.048884000	0.708858000
H	3.461148000	-0.283322000	1.773597000
C	3.799738000	-1.128562000	-0.103813000
H	3.934371000	-0.934101000	-1.164865000
C	3.940985000	1.336458000	0.359420000
H	3.749302000	1.550406000	-0.697155000
H	3.413676000	2.101699000	0.938939000
C	3.786115000	-2.558162000	0.286509000
H	3.694760000	-2.716670000	1.362636000
H	2.961269000	-3.070788000	-0.224240000
H	4.697875000	-3.038498000	-0.081808000
H	6.396722000	-1.114738000	0.770737000
C	6.305018000	0.564066000	-0.259004000
H	7.372561000	0.737131000	-0.085646000
H	6.109261000	0.759024000	-1.317658000
C	5.450608000	1.462073000	0.626737000
H	5.670521000	1.262223000	1.684345000
H	5.753278000	2.499797000	0.445493000
O	6.015017000	-0.833904000	-0.072545000

TS1'$_{OH6E}$

Sum of electronic and zero-point Energies = -2163.765846
Sum of electronic and thermal Free Energies = -2163.837468
Esolv = -2165.2260346

C	-1.214569000	-0.357685000	-1.853335000
C	-1.192603000	0.736157000	-2.713634000
H	-1.079365000	1.747560000	-2.339184000
Au	-1.189401000	-0.189880000	0.189839000
C	-1.382751000	-1.650143000	-2.360256000
H	-1.416887000	-2.514587000	-1.702309000
C	-1.508844000	-1.841011000	-3.738420000
H	-1.634747000	-2.847619000	-4.126580000
C	-1.474865000	-0.750852000	-4.606615000
H	-1.572671000	-0.902666000	-5.676910000
C	-1.319870000	0.533310000	-4.093066000
H	-1.295407000	1.390176000	-4.760322000
P	1.221545000	0.078235000	0.379425000
C	1.364030000	0.112967000	2.210015000
C	2.214229000	-1.434726000	-0.232181000
C	1.755745000	1.817076000	-0.229057000
C	2.615489000	0.292954000	2.831493000
C	0.238619000	-0.034752000	3.030685000
C	1.596015000	-2.666569000	0.470871000
C	3.714903000	-1.353004000	0.136762000
C	2.092448000	-1.611713000	-1.762653000
C	3.011117000	2.356367000	0.499862000
C	2.048817000	1.809366000	-1.745172000
C	0.582464000	2.775671000	0.086307000
H	3.504691000	0.417905000	2.229991000
C	2.746105000	0.328602000	4.213617000
C	0.374648000	-0.002357000	4.422148000
N	-1.096225000	-0.313291000	2.464382000

H	1.678216000	-2.562736000	1.560505000
H	0.527705000	-2.746437000	0.219361000
C	2.323575000	-3.949364000	0.021583000
C	4.431681000	-2.642236000	-0.321084000
H	3.844112000	-1.262670000	1.218458000
H	4.182664000	-0.482708000	-0.338631000
C	2.809511000	-2.904962000	-2.198940000
H	2.544142000	-0.765026000	-2.285014000
H	1.043473000	-1.654469000	-2.063101000
H	2.835820000	2.409563000	1.577996000
H	3.872014000	1.700085000	0.326427000
C	3.340575000	3.778699000	-0.004590000
C	2.345129000	3.243144000	-2.228963000
H	2.928978000	1.189036000	-1.944203000
H	1.215460000	1.381903000	-2.310221000
C	0.910702000	4.198239000	-0.407789000
H	0.394148000	2.792494000	1.168876000
H	-0.336722000	2.423783000	-0.402443000
H	3.725215000	0.471021000	4.659315000
C	1.617890000	0.183124000	5.013798000
H	-0.486396000	-0.136353000	5.067573000
C	-2.105319000	0.607398000	3.051413000
C	-1.436486000	-1.728005000	2.806557000
H	1.866400000	-4.801561000	0.538246000
C	3.809282000	-3.848570000	0.394630000
C	2.178484000	-4.114075000	-1.496746000
H	5.488619000	-2.550875000	-0.045491000
C	4.296911000	-2.808874000	-1.838576000
H	2.693851000	-3.002512000	-3.284559000
H	4.237530000	4.118317000	0.525938000
C	2.164960000	4.711518000	0.311410000
C	3.599641000	3.761032000	-1.515224000
C	1.156376000	4.164555000	-1.922866000
H	2.518489000	3.206688000	-3.310551000
H	0.058017000	4.849394000	-0.179766000
H	1.700399000	0.206130000	6.095706000
H	-3.088674000	0.333950000	2.674569000
H	-1.867414000	1.635111000	2.772065000
H	-2.136126000	0.535565000	4.141225000
H	-1.497882000	-1.857387000	3.891677000
H	-0.659582000	-2.386836000	2.418269000
H	-2.393727000	-1.996108000	2.364203000
H	4.330082000	-4.767424000	0.102438000
H	3.925870000	-3.752229000	1.481843000
H	1.119518000	-4.207271000	-1.772371000
H	2.670228000	-5.038133000	-1.821401000
H	4.763073000	-1.963661000	-2.360726000
H	4.825964000	-3.711483000	-2.165121000
H	2.394068000	5.731335000	-0.017814000
H	1.992871000	4.760086000	1.394355000
H	4.462115000	3.124940000	-1.750786000
H	3.847428000	4.769778000	-1.864865000
H	0.256882000	3.820674000	-2.451592000
H	1.363153000	5.176375000	-2.289626000
C	-3.467492000	-0.330700000	-0.127592000
C	-4.178154000	-1.451077000	0.629788000
C	-3.935177000	0.993279000	0.130788000
H	-3.435657000	-0.541304000	-1.196580000
C	-5.637740000	-1.596999000	0.170499000
H	-4.181772000	-1.265841000	1.711186000
H	-3.661924000	-2.402359000	0.465695000
H	-4.165047000	1.253842000	1.163067000
C	-6.477473000	-0.366537000	0.472804000
H	-6.097726000	-2.450811000	0.680676000
H	-5.674910000	-1.825791000	-0.902599000
H	-7.514574000	-0.483608000	0.143650000
H	-6.491342000	-0.141341000	1.542857000
H	-6.087898000	0.774025000	-1.105540000
O	-5.940194000	0.828620000	-0.148872000
C	-3.717309000	2.164333000	-0.761394000
H	-4.538522000	2.877614000	-0.653069000
H	-2.812300000	2.693686000	-0.436795000
H	-3.593440000	1.880364000	-1.807602000

4_{OH5}

Sum of electronic and zero-point Energies = -2124.506375
Sum of electronic and thermal Free Energies = -2124.578148
Esolv = -2125.9263434

C	1.561858000	-0.837985000	-1.413828000
C	1.693708000	-2.187361000	-1.756341000
H	1.493115000	-2.972616000	-1.032431000
Au	1.130654000	-0.369083000	0.528759000
C	1.838456000	0.150966000	-2.358733000
H	1.761379000	1.204441000	-2.108618000
C	2.221555000	-0.213397000	-3.654216000
H	2.426541000	0.562209000	-4.386781000
C	2.336178000	-1.556274000	-4.004731000
H	2.631313000	-1.834715000	-5.011486000
C	2.072115000	-2.540885000	-3.054028000
H	2.159657000	-3.591511000	-3.315618000
P	-1.207976000	0.212479000	0.249812000
C	-1.632005000	0.539756000	2.007594000
C	-1.450053000	1.861088000	-0.679538000
C	-2.270352000	-1.273602000	-0.298363000
C	-2.938996000	0.902812000	2.385959000
C	-0.667943000	0.421797000	3.016552000
C	-0.260465000	2.767534000	-0.279133000
C	-2.753893000	2.605732000	-0.304091000
C	-1.448327000	1.626775000	-2.206617000
C	-3.786278000	-0.970693000	-0.327798000
C	-1.840767000	-1.768738000	-1.698517000
C	-2.001400000	-2.398022000	0.731181000
H	-3.707159000	1.010776000	1.632983000
C	-3.277293000	1.138250000	3.712186000
C	-1.009144000	0.664687000	4.350775000
N	0.726039000	0.031598000	2.716671000
H	-0.282074000	2.950716000	0.804176000
H	0.693497000	2.271877000	-0.508138000
C	-0.336501000	4.109083000	-1.032791000
C	-2.830638000	3.946338000	-1.065893000
H	-2.776655000	2.818371000	0.769009000
H	-3.632641000	1.997264000	-0.547023000
C	-1.500461000	2.978419000	-2.944750000
H	-2.329487000	1.040535000	-2.489083000
H	-0.566809000	1.057741000	-2.518656000
H	-4.137849000	-0.641881000	0.653941000
H	-4.004912000	-0.176013000	-1.050553000
C	-4.564286000	-2.247159000	-0.712496000
C	-2.626296000	-3.041246000	-2.071346000
H	-2.026055000	-1.000865000	-2.454413000
H	-0.769675000	-1.982751000	-1.714666000
C	-2.790035000	-3.664648000	0.345457000
H	-2.296766000	-2.074953000	1.736954000
H	-0.925715000	-2.631548000	0.759218000
H	-4.294731000	1.415107000	3.968729000
C	-2.305611000	1.020202000	4.700865000
H	-0.267245000	0.576402000	5.137401000
C	1.034428000	-1.233707000	3.446660000
C	1.637351000	1.124203000	3.160345000
H	0.523392000	4.722501000	-0.737100000

C	-1.641227000	4.827437000	-0.663448000
C	-0.298504000	3.845412000	-2.544853000
H	-3.768729000	4.437983000	-0.783780000
C	-2.804279000	3.698703000	-2.578910000
H	-1.473335000	2.779709000	-4.022353000
H	-5.632142000	-2.000163000	-0.720322000
C	-4.291130000	-3.342284000	0.327594000
C	-4.126918000	-2.726276000	-2.101534000
C	-2.345589000	-4.143980000	-1.042464000
H	-2.292928000	-3.364536000	-3.064394000
H	-2.584499000	-4.438681000	1.094401000
H	-2.551113000	1.202685000	5.742145000
H	2.050335000	-1.555502000	3.218639000
H	0.330196000	-2.006063000	3.134511000
H	0.951521000	-1.094751000	4.528011000
H	1.546460000	1.302933000	4.235268000
H	1.384205000	2.041058000	2.626133000
H	2.670139000	0.851072000	2.944763000
H	-1.696116000	5.793015000	-1.179033000
H	-1.668665000	5.041552000	0.412761000
H	0.641465000	3.350506000	-2.825416000
H	-0.324118000	4.795832000	-3.090136000
H	-3.669922000	3.098168000	-2.885704000
H	-2.875683000	4.651455000	-3.116120000
H	-4.863325000	-4.244058000	0.081575000
H	-4.624659000	-3.018678000	1.322057000
H	-4.341476000	-1.959431000	-2.856768000
H	-4.695872000	-3.618407000	-2.387560000
H	-1.276942000	-4.398070000	-1.035349000
H	-2.884396000	-5.059261000	-1.313175000
C	3.174508000	-0.978199000	0.889389000
H	3.104465000	-2.064574000	0.763737000
H	3.415260000	-0.735434000	1.928664000
C	4.189862000	-0.402130000	-0.026520000
H	4.045942000	-0.683086000	-1.067955000
C	6.120495000	1.057885000	-0.288470000
C	6.717223000	-0.174766000	0.360324000
H	5.567110000	-1.785659000	0.972317000
H	6.212531000	0.997644000	-1.377032000
H	6.636945000	1.961756000	0.042370000
H	6.920981000	-0.041962000	1.425494000
H	7.581633000	-0.599446000	-0.150373000
C	4.649197000	1.029847000	0.139923000
H	4.539351000	1.353260000	1.181126000
H	4.036969000	1.685514000	-0.485061000
O	5.617715000	-1.167712000	0.223813000

4_{OH5Z}

Sum of electronic and zero-point Energies = -2163.771816
Sum of electronic and thermal Free Energies = -2163.844594
Esolv = -2165.231684

Au	1.076312000	-0.232733000	0.522426000
P	-1.330197000	0.058637000	0.234096000
C	-1.853623000	-0.066247000	1.990450000
C	-1.826145000	1.812958000	-0.337744000
C	-2.098919000	-1.442039000	-0.663259000
C	-3.213818000	0.005662000	2.349600000
C	-0.913885000	-0.225506000	3.016426000
C	-0.869791000	2.786259000	0.393990000
C	-3.274681000	2.210240000	0.031425000
C	-1.658062000	1.957562000	-1.866428000
C	-3.642463000	-1.493354000	-0.596813000
C	-1.673200000	-1.471998000	-2.148285000
C	-1.531148000	-2.693524000	0.050734000
H	-3.966537000	0.132218000	1.584057000
C	-3.626614000	-0.074415000	3.673210000
C	-1.331183000	-0.293434000	4.349736000
N	0.535951000	-0.317394000	2.737949000
H	-1.011362000	2.698871000	1.479951000
H	0.176843000	2.530618000	0.177067000
C	-1.147216000	4.236608000	-0.046820000
C	-3.552090000	3.660988000	-0.418870000
H	-3.421617000	2.156418000	1.113954000
H	-3.996300000	1.534862000	-0.442802000
C	-1.914024000	3.417812000	-2.287654000
H	-2.384717000	1.318530000	-2.378150000
H	-0.661167000	1.638651000	-2.186554000
H	-3.984836000	-1.509498000	0.441312000
H	-4.078233000	-0.614543000	-1.086551000
C	-4.152169000	-2.777172000	-1.286501000
C	-2.180070000	-2.764537000	-2.817362000
H	-2.090909000	-0.614604000	-2.682246000
H	-0.586400000	-1.407030000	-2.239465000
C	-2.053181000	-3.973633000	-0.630016000
H	-1.824198000	-2.693230000	1.108423000
H	-0.430687000	-2.683932000	0.010727000
H	-4.683313000	-0.017661000	3.913689000
C	-2.678362000	-0.220418000	4.680379000
H	-0.609414000	-0.404396000	5.151908000
C	1.050200000	-1.595540000	3.306055000
C	1.213765000	0.845178000	3.381326000
H	-0.449654000	4.898194000	0.481012000
C	-2.592187000	4.609494000	0.310925000
C	-0.939515000	4.356069000	-1.562977000
H	-4.587229000	3.902248000	-0.151272000
C	-3.358468000	3.793379000	-1.933962000
H	-1.764224000	3.490443000	-3.371112000
H	-5.246422000	-2.780007000	-1.223559000
C	-3.586262000	-4.002242000	-0.555803000
C	-3.712060000	-2.797678000	-2.755040000
C	-1.603524000	-3.990185000	-2.096926000
H	-1.849402000	-2.756738000	-3.862558000
H	-1.638450000	-4.839358000	-0.099861000
H	-2.980814000	-0.277487000	5.721108000
H	2.121198000	-1.676844000	3.124577000
H	0.533416000	-2.431556000	2.832560000
H	0.887562000	-1.645041000	4.385665000
H	1.066261000	0.837329000	4.465051000
H	0.802449000	1.769882000	2.974555000
H	2.285977000	0.809752000	3.182488000
H	-2.794022000	5.646745000	0.020073000
H	-2.744899000	4.550146000	1.396278000
H	0.097695000	4.110836000	-1.828923000
H	-1.107188000	5.391054000	-1.882391000
H	-4.062529000	3.144444000	-2.470019000
H	-3.572792000	4.820574000	-2.250875000
H	-3.964883000	-4.922265000	-1.015564000
H	-3.918407000	-4.012321000	0.490343000
H	-4.134530000	-1.940355000	-3.294311000
H	-4.092975000	-3.699378000	-3.248307000
H	-0.506676000	-3.992086000	-2.160286000
H	-1.946967000	-4.910020000	-2.584071000
C	4.156084000	0.262564000	0.285839000
H	3.876138000	1.311000000	0.397803000
C	3.181047000	-0.679182000	0.917787000
C	4.876596000	-0.009587000	-1.012352000
H	5.027446000	-1.082587000	-1.159087000
H	4.283443000	0.363702000	-1.849982000
C	6.738382000	0.362141000	0.470438000
H	7.383583000	1.107450000	0.935671000

H	7.200072000	-0.627008000	0.506715000
C	6.219161000	0.721189000	-0.907635000
H	6.085317000	1.803796000	-0.996867000
H	6.922005000	0.407984000	-1.683016000
C	3.477068000	-2.159999000	0.693406000
H	4.490974000	-2.445475000	1.010548000
H	2.790537000	-2.795656000	1.257619000
H	3.385816000	-2.438859000	-0.358647000
H	3.182880000	-0.442105000	1.988597000
C	1.623796000	-0.060057000	-1.434932000
C	1.838452000	1.208369000	-1.980541000
C	1.840750000	-1.197444000	-2.215804000
C	2.253831000	1.334263000	-3.310541000
H	1.685900000	2.107070000	-1.390505000
C	2.256002000	-1.063421000	-3.543755000
H	1.673328000	-2.192599000	-1.814179000
C	2.463471000	0.200440000	-4.093757000
H	2.408747000	2.324692000	-3.729265000
H	2.411143000	-1.953850000	-4.146315000
H	2.783270000	0.301041000	-5.126236000
H	5.496156000	-0.451674000	1.890860000
O	5.496324000	0.312544000	1.291743000

4_{OH5E}

Sum of electronic and zero-point Energies = -2163.761686

Sum of electronic and thermal Free Energies = -2163.834697

Esolv = -2165.22397465

Au	1.112535000	-0.584493000	0.351051000
P	-1.208626000	0.205124000	0.324421000
C	-1.557611000	0.174984000	2.128041000
C	-1.304844000	2.047962000	-0.192224000
C	-2.447643000	-0.990201000	-0.501215000
C	-2.802458000	0.593698000	2.636521000
C	-0.591239000	-0.264437000	3.042499000
C	-0.000452000	2.713877000	0.309222000
C	-2.491571000	2.815467000	0.438778000
C	-1.411813000	2.179547000	-1.727550000
C	-3.921231000	-0.543133000	-0.360939000
C	-2.125439000	-1.167218000	-2.003067000
C	-2.268925000	-2.354739000	0.206445000
H	-3.570719000	0.940874000	1.960275000
C	-3.077644000	0.587644000	3.997669000
C	-0.872363000	-0.272322000	4.412783000
N	0.699276000	-0.824188000	2.589986000
H	0.059909000	2.636947000	1.403821000
H	0.875180000	2.192713000	-0.102541000
C	0.040459000	4.195277000	-0.112896000
C	-2.453208000	4.297348000	0.007093000
H	-2.434755000	2.776477000	1.530439000
H	-3.446629000	2.372553000	0.133501000
C	-1.342454000	3.664079000	-2.136015000
H	-2.370978000	1.771682000	-2.064080000
H	-0.624445000	1.610089000	-2.230296000
H	-4.197007000	-0.437642000	0.691958000
H	-4.078429000	0.424337000	-0.851986000
C	-4.849540000	-1.601046000	-0.994932000
C	-3.061071000	-2.224271000	-2.621846000
H	-2.251024000	-0.223745000	-2.540228000
H	-1.086300000	-1.477195000	-2.135545000
C	-3.207859000	-3.403418000	-0.421573000
H	-2.489853000	-2.261022000	1.277305000
H	-1.226734000	-2.693914000	0.105697000
H	-4.047466000	0.919280000	4.354168000
C	-2.103675000	0.156810000	4.892018000
H	-0.141259000	-0.631676000	5.128426000
C	0.646243000	-2.297791000	2.834945000
C	1.821048000	-0.229005000	3.358824000
H	0.979310000	4.633848000	0.247497000
C	-1.151518000	4.936138000	0.507145000
C	-0.029666000	4.288984000	-1.643687000
H	-3.312766000	4.800739000	0.464434000
C	-2.533123000	4.408473000	-1.519733000
H	-1.392898000	3.719376000	-3.229589000
H	-5.882917000	-1.254974000	-0.877301000
C	-4.663388000	-2.940137000	-0.268588000
C	-4.517251000	-1.763826000	-2.482647000
C	-2.868726000	-3.568643000	-1.908623000
H	-2.801288000	-2.324764000	-3.682125000
H	-3.062228000	-4.354048000	0.105134000
H	-2.299755000	0.142826000	5.959381000
H	1.562575000	-2.765548000	2.481467000
H	-0.206153000	-2.717907000	2.301077000
H	0.528280000	-2.505031000	3.903348000
H	1.703413000	-0.374089000	4.435515000
H	1.879335000	0.840902000	3.151003000
H	2.742971000	-0.724287000	3.060186000
H	-1.119914000	5.995817000	0.229018000
H	-1.099809000	4.894846000	1.602788000
H	0.831596000	3.781761000	-2.100308000
H	0.026272000	5.337963000	-1.956651000
H	-3.477024000	3.986721000	-1.887318000
H	-2.520569000	5.462403000	-1.820798000
H	-5.340300000	-3.692480000	-0.689218000
H	-4.923756000	-2.839930000	0.793091000
H	-4.671389000	-0.816866000	-3.015672000
H	-5.192105000	-2.496470000	-2.940119000
H	-1.835410000	-3.921012000	-2.028444000
H	-3.515438000	-4.331019000	-2.357955000
C	3.250235000	-1.180391000	0.287107000
C	1.365853000	-0.641791000	-1.679592000
C	1.637370000	0.480610000	-2.458296000
C	1.344083000	-1.910691000	-2.268496000
C	1.861896000	0.336175000	-3.832775000
H	1.662440000	1.476140000	-2.027007000
C	1.565819000	-2.044649000	-3.640611000
H	1.154124000	-2.801665000	-1.675735000
C	1.823967000	-0.922054000	-4.425918000
H	2.060252000	1.218644000	-4.434625000
H	1.539003000	-3.033179000	-4.089885000
H	1.997446000	-1.029378000	-5.492001000
C	3.551215000	-2.526569000	0.942109000
H	3.578197000	-2.485288000	2.036934000
H	2.822862000	-3.288177000	0.651255000
H	4.527627000	-2.901455000	0.613017000
H	3.390340000	-1.284115000	-0.789725000
C	4.139350000	-0.081174000	0.755737000
C	4.213540000	1.227777000	-0.016150000
H	4.162572000	0.048595000	1.839420000
C	6.419816000	0.348850000	-0.370186000
H	6.163582000	-0.825512000	1.312813000
C	5.294072000	1.051532000	-1.092338000
H	4.479783000	2.048754000	0.656631000
H	3.247236000	1.455983000	-0.465789000
H	6.999257000	1.015916000	0.272832000
H	7.075085000	-0.256042000	-0.997773000
H	5.628957000	2.009797000	-1.496142000
H	4.925807000	0.443507000	-1.923214000
O	5.679827000	-0.601248000	0.501021000

4’$_{OH5E}$

Sum of electronic and zero-point Energies = -2163.774766
Sum of electronic and thermal Free Energies = -2163.847685
Esolv = -2165.2347096

C	1.479436000	-0.517745000	-1.570343000
C	1.496048000	-1.771376000	-2.187228000
H	1.238276000	-2.670908000	-1.635983000
Au	1.095141000	-0.384603000	0.435102000
C	1.797987000	0.622165000	-2.311867000
H	1.781291000	1.609038000	-1.859123000
C	2.131654000	0.504995000	-3.665171000
H	2.371212000	1.398868000	-4.234249000
C	2.149359000	-0.743909000	-4.281223000
H	2.407317000	-0.831607000	-5.331907000
C	1.827706000	-1.879485000	-3.540817000
H	1.829038000	-2.858276000	-4.011837000
P	-1.268572000	0.183286000	0.270724000
C	-1.669221000	0.274265000	2.061463000
C	-1.567651000	1.935740000	-0.429313000
C	-2.295756000	-1.265285000	-0.433223000
C	-2.977099000	0.561017000	2.499291000
C	-0.686726000	0.056304000	3.035399000
C	-0.423060000	2.818737000	0.125506000
C	-2.907932000	2.568868000	0.013434000
C	-1.524790000	1.924258000	-1.973449000
C	-3.821993000	-1.095519000	-0.254884000
C	-2.000349000	-1.475986000	-1.935279000
C	-1.842721000	-2.520112000	0.353263000
H	-3.759666000	0.741740000	1.775691000
C	-3.299302000	0.626717000	3.848600000
C	-1.012255000	0.129889000	4.393718000
N	0.714785000	-0.249328000	2.674265000
H	-0.471837000	2.843465000	1.222868000
H	0.553863000	2.396982000	-0.150746000
C	-0.539674000	4.251431000	-0.429344000
C	-3.024531000	4.000987000	-0.551766000
H	-2.961079000	2.628011000	1.104407000
H	-3.755462000	1.967217000	-0.335278000
C	-1.615251000	3.365119000	-2.512445000
H	-2.377825000	1.357185000	-2.359379000
H	-0.616787000	1.436529000	-2.341672000
H	-4.077947000	-0.978840000	0.801504000
H	-4.176015000	-0.207883000	-0.792507000
C	-4.551591000	-2.348646000	-0.785333000
C	-2.726619000	-2.736918000	-2.443642000
H	-2.344581000	-0.616399000	-2.516054000
H	-0.925814000	-1.570274000	-2.108195000
C	-2.583275000	-3.767166000	-0.167675000
H	-2.046787000	-2.391561000	1.424009000
H	-0.757747000	-2.666775000	0.236584000
H	-4.317921000	0.848248000	4.150262000
C	-2.309789000	0.411082000	4.802204000
H	-0.256231000	-0.032182000	5.154671000
C	1.089891000	-1.560037000	3.278468000
C	1.586407000	0.834654000	3.209853000
H	0.287411000	4.848052000	-0.025374000
C	-1.880607000	4.858589000	0.004568000
C	-0.458897000	4.210593000	-1.961766000
H	-3.987658000	4.411343000	-0.227296000
C	-2.955367000	3.975445000	-2.083201000
H	-1.557416000	3.323427000	-3.606243000
H	-5.627729000	-2.193763000	-0.646289000
C	-4.095388000	-3.575889000	0.015146000
C	-4.239325000	-2.551644000	-2.272455000
C	-2.260294000	-3.966950000	-1.654185000
H	-2.483714000	-2.861607000	-3.505326000
H	-2.244936000	-4.635196000	0.410767000
H	-2.541552000	0.461436000	5.861263000
H	2.122103000	-1.800811000	3.027382000
H	0.427652000	-2.335188000	2.890212000
H	1.002101000	-1.533890000	4.367726000
H	1.502987000	0.907405000	4.297823000
H	1.288349000	1.786085000	2.767283000
H	2.627277000	0.627335000	2.960577000
H	-1.964261000	5.885883000	-0.368083000
H	-1.939210000	4.913614000	1.099283000
H	0.506128000	3.796548000	-2.284834000
H	-0.512386000	5.228012000	-2.365916000
H	-3.788420000	3.392834000	-2.496306000
H	-3.054758000	4.992360000	-2.480029000
H	-4.628767000	-4.469004000	-0.329814000
H	-4.339767000	-3.453262000	1.078225000
H	-4.587236000	-1.691607000	-2.858640000
H	-4.775115000	-3.429408000	-2.651816000
H	-1.182268000	-4.125956000	-1.793112000
H	-2.760646000	-4.867255000	-2.028910000
C	3.169102000	-1.037392000	0.730814000
H	3.353861000	-0.766459000	1.776598000
C	4.105693000	-0.272977000	-0.130280000
H	4.018892000	-0.510288000	-1.189734000
C	5.847092000	1.436850000	-0.251024000
C	6.592300000	0.254887000	0.331877000
H	5.696365000	-1.556007000	0.758291000
H	5.963542000	1.460464000	-1.338709000
H	6.230328000	2.378546000	0.148211000
H	6.745773000	0.337500000	1.410797000
H	7.524706000	-0.000537000	-0.172676000
C	4.384888000	1.188532000	0.137049000
H	4.217029000	1.425963000	1.193399000
H	3.708976000	1.804320000	-0.461032000
O	5.654520000	-0.863234000	0.079628000
C	3.253809000	-2.553853000	0.538337000
H	3.236226000	-2.826112000	-0.519339000
H	4.170552000	-2.983361000	0.968788000
H	2.431604000	-3.079920000	1.030579000

4$_{OH6}$

Sum of electronic and zero-point Energies = -2124.494385
Sum of electronic and thermal Free Energies = -2124.565145
Esolv = -2125.9225978

C	-1.614802000	-0.283652000	1.570753000
C	-1.643430000	-1.543610000	2.176134000
H	-1.402421000	-2.441324000	1.613515000
Au	-1.221383000	-0.122801000	-0.427986000
C	-1.935810000	0.851526000	2.318076000
H	-1.913091000	1.841414000	1.871176000
C	-2.270856000	0.725315000	3.670127000
H	-2.506173000	1.615999000	4.245814000
C	-2.296701000	-0.528519000	4.276366000
H	-2.556854000	-0.622733000	5.325970000
C	-1.983431000	-1.661023000	3.527355000
H	-1.997767000	-2.643940000	3.989682000
P	1.200517000	0.082724000	-0.263939000
C	1.598087000	0.226755000	-2.051594000
C	1.704180000	1.746482000	0.527993000
C	2.039610000	-1.525304000	0.337504000
C	2.927185000	0.386645000	-2.489601000
C	0.590423000	0.176969000	-3.023125000
C	0.752655000	2.804070000	-0.084977000

C	3.159257000	2.175476000	0.228047000
C	1.513882000	1.709180000	2.060755000
C	3.542710000	-1.623512000	-0.011100000
C	1.883393000	-1.691081000	1.865443000
C	1.300725000	-2.676761000	-0.388442000
H	3.730521000	0.428854000	-1.767107000
C	3.245000000	0.492521000	-3.837648000
C	0.913031000	0.280217000	-4.379917000
N	-0.833587000	0.007040000	-2.659681000
H	0.888352000	2.845622000	-1.174006000
H	-0.295464000	2.529858000	0.108412000
C	1.035795000	4.190598000	0.524977000
C	3.435359000	3.560850000	0.851371000
H	3.323347000	2.251617000	-0.850551000
H	3.868731000	1.442228000	0.629767000
C	1.772586000	3.106107000	2.657890000
H	2.222680000	1.007350000	2.508548000
H	0.509214000	1.365803000	2.323234000
H	3.692019000	-1.548688000	-1.091902000
H	4.104566000	-0.812480000	0.467286000
C	4.099643000	-2.986273000	0.455108000
C	2.422821000	-3.066555000	2.303726000
H	2.455284000	-0.916638000	2.384047000
H	0.838504000	-1.578677000	2.168253000
C	1.863371000	-4.037848000	0.064242000
H	1.420166000	-2.572208000	-1.475255000
H	0.223625000	-2.636311000	-0.167610000
H	4.279801000	0.615787000	-4.140401000
C	2.231412000	0.437917000	-4.788814000
H	0.139010000	0.238232000	-5.138930000
C	-1.326358000	-1.264625000	-3.262683000
C	-1.605538000	1.167772000	-3.188309000
H	0.347849000	4.914285000	0.071279000
C	2.486779000	4.592866000	0.227215000
C	0.812719000	4.132374000	2.042075000
H	4.474308000	3.828270000	0.626890000
C	3.223218000	3.512478000	2.369020000
H	1.610299000	3.047198000	3.740399000
H	5.165343000	-3.017323000	0.200926000
C	3.357722000	-4.111811000	-0.277790000
C	3.917562000	-3.145531000	1.969308000
C	1.667869000	-4.188557000	1.578973000
H	2.277292000	-3.158715000	3.386282000
H	1.320139000	-4.829831000	-0.465409000
H	2.460782000	0.516885000	-5.846647000
H	-2.372441000	-1.420049000	-2.996222000
H	-0.728701000	-2.095146000	-2.884780000
H	-1.250790000	-1.240040000	-4.353390000
H	-1.528598000	1.232883000	-4.276892000
H	-1.213186000	2.086801000	-2.750690000
H	-2.658169000	1.060185000	-2.927986000
H	2.690210000	5.588141000	0.638596000
H	2.650801000	4.659301000	-0.856095000
H	-0.229185000	3.865891000	2.266068000
H	0.984653000	5.120542000	2.484005000
H	3.917723000	2.799907000	2.831712000
H	3.438251000	4.493161000	2.808902000
H	3.761510000	-5.085823000	0.021221000
H	3.506663000	-4.027426000	-1.362028000
H	4.470024000	-2.364811000	2.507288000
H	4.330470000	-4.106535000	2.297119000
H	0.599340000	-4.158021000	1.832274000
H	2.039159000	-5.165576000	1.908961000
C	-4.008975000	-1.321721000	0.031921000
H	-3.967681000	-1.250602000	1.117808000
H	-3.714536000	-2.320808000	-0.291971000
C	-3.381401000	-0.181176000	-0.699960000
C	-4.066262000	1.142256000	-0.338934000
H	-3.488537000	-0.367511000	-1.776416000
C	-6.227089000	-0.058672000	0.155404000
C	-5.582298000	1.086376000	-0.589761000
H	-3.888742000	1.372380000	0.717690000
H	-3.641721000	1.969327000	-0.916035000
H	-7.279744000	-0.226493000	-0.078310000
H	-6.099342000	-0.003166000	1.238074000
H	-6.067563000	2.009410000	-0.251513000
H	-5.800681000	1.002308000	-1.662570000
H	-5.749758000	-1.586629000	-1.120825000
O	-5.549643000	-1.349015000	-0.197836000

4_{OH6Z}

Sum of electronic and zero-point Energies = -2163.765516
Sum of electronic and thermal Free Energies = -2163.765516
Esolv = -2165.2307731

C	1.426887000	0.455369000	-1.646995000
C	1.326779000	-0.518253000	-2.639650000
H	1.111548000	-1.553089000	-2.392936000
Au	1.201138000	0.020781000	0.337909000
C	1.689141000	1.781695000	-2.000781000
H	1.752259000	2.561695000	-1.247556000
C	1.858198000	2.125474000	-3.344591000
H	2.056204000	3.160438000	-3.608405000
C	1.766896000	1.152110000	-4.337466000
H	1.898333000	1.421499000	-5.380722000
C	1.500040000	-0.167268000	-3.982352000
H	1.420981000	-0.934575000	-4.747227000
P	-1.243920000	-0.080318000	0.323937000
C	-1.536929000	-0.338942000	2.120674000
C	-2.065431000	1.585672000	-0.121600000
C	-1.898553000	-1.658400000	-0.541580000
C	-2.843844000	-0.457119000	2.633302000
C	-0.472497000	-0.423703000	3.026050000
C	-1.397419000	2.642542000	0.791413000
C	-3.592046000	1.612434000	0.121774000
C	-1.798864000	1.957126000	-1.598460000
C	-3.250610000	-2.162440000	0.016870000
C	-2.066077000	-1.424595000	-2.059235000
C	-0.843722000	-2.762152000	-0.284390000
H	-3.690416000	-0.409443000	1.963242000
C	-3.086244000	-0.647762000	3.987279000
C	-0.718192000	-0.618840000	4.389383000
N	0.933167000	-0.282047000	2.590192000
H	-1.575820000	2.401027000	1.847084000
H	-0.308651000	2.645005000	0.628667000
C	-1.963391000	4.041988000	0.480516000
C	-4.146312000	3.017779000	-0.196628000
H	-3.822345000	1.389975000	1.166906000
H	-4.094938000	0.866334000	-0.505084000
C	-2.352228000	3.364421000	-1.896314000
H	-2.281066000	1.240236000	-2.267624000
H	-0.728773000	1.930062000	-1.814312000
H	-3.169570000	-2.370606000	1.087745000
H	-4.033174000	-1.407322000	-0.123288000
C	-3.663386000	-3.467157000	-0.698359000
C	-2.448948000	-2.745198000	-2.755927000
H	-2.867636000	-0.698107000	-2.230294000
H	-1.154616000	-1.011220000	-2.501203000
C	-1.254794000	-4.067827000	-0.992054000
H	-0.750632000	-2.941626000	0.795968000
H	0.142637000	-2.443178000	-0.649451000

H	-4.106845000	-0.734090000	4.345859000
C	-2.015802000	-0.730847000	4.871667000
H	0.101546000	-0.679943000	5.097337000
C	1.687174000	-1.499636000	2.993706000
C	1.497408000	0.931520000	3.250751000
H	-1.476295000	4.765658000	1.145040000
C	-3.478664000	4.045414000	0.727402000
C	-1.675261000	4.395668000	-0.984345000
H	-5.226980000	3.001297000	-0.014077000
C	-3.867343000	3.375131000	-1.660889000
H	-2.135741000	3.596425000	-2.945616000
H	-4.626455000	-3.785385000	-0.282930000
C	-2.603504000	-4.544880000	-0.437280000
C	-3.797406000	-3.226454000	-2.206481000
C	-1.374245000	-3.811075000	-2.500969000
H	-2.531872000	-2.550855000	-3.831483000
H	-0.483912000	-4.824153000	-0.800145000
H	-2.184898000	-0.880869000	5.933147000
H	2.728301000	-1.404896000	2.683828000
H	1.240820000	-2.373110000	2.516100000
H	1.671598000	-1.641748000	4.077661000
H	1.488950000	0.827007000	4.339390000
H	0.895047000	1.797992000	2.974757000
H	2.526640000	1.092544000	2.929091000
H	-3.885624000	5.044449000	0.533798000
H	-3.695767000	3.814470000	1.778355000
H	-0.592393000	4.415411000	-1.167122000
H	-2.050270000	5.401089000	-1.207724000
H	-4.362993000	2.661440000	-2.331240000
H	-4.281046000	4.363723000	-1.891222000
H	-2.895286000	-5.484946000	-0.919258000
H	-2.523299000	-4.752262000	0.637668000
H	-4.578423000	-2.482935000	-2.409676000
H	-4.104637000	-4.151293000	-2.708214000
H	-0.408217000	-3.489598000	-2.913943000
H	-1.638637000	-4.740711000	-3.017821000
C	3.385061000	0.084856000	0.532346000
H	3.458357000	0.086186000	1.627466000
C	4.073356000	-1.166482000	0.070769000
C	4.079818000	1.355273000	0.031156000
H	4.043540000	1.418948000	-1.059923000
H	3.554710000	2.239742000	0.404729000
H	5.679215000	-1.092692000	1.517190000
C	6.326026000	0.197495000	0.092727000
H	7.327086000	0.134914000	0.524166000
H	6.399111000	0.073711000	-0.987944000
C	5.539346000	1.419466000	0.508719000
H	5.584531000	1.534342000	1.600242000
H	6.045828000	2.296585000	0.089845000
O	5.635459000	-1.042944000	0.546741000
C	4.200000000	-1.521748000	-1.384332000
H	4.973632000	-2.280171000	-1.526756000
H	3.252536000	-1.955602000	-1.708491000
H	4.393832000	-0.663916000	-2.029131000
H	3.806522000	-2.046836000	0.661404000

4_{OH6E}

Sum of electronic and zero-point Energies = -2163.766765
Sum of electronic and thermal Free Energies = -2163.838402
Esolv = -2165.234833

C	-1.292669000	-0.319912000	-1.849983000
C	-1.273268000	0.800299000	-2.677654000
H	-1.154863000	1.799081000	-2.271480000
Au	-1.223746000	-0.207431000	0.192729000
C	-1.469908000	-1.593599000	-2.400400000
H	-1.506555000	-2.478225000	-1.769727000
C	-1.591350000	-1.741409000	-3.784123000
H	-1.716001000	-2.735215000	-4.204547000
C	-1.554003000	-0.624947000	-4.618029000
H	-1.649686000	-0.742582000	-5.692815000
C	-1.398468000	0.641906000	-4.062826000
H	-1.370528000	1.519435000	-4.702674000
P	1.204537000	0.066101000	0.378215000
C	1.365301000	0.081247000	2.208812000
C	2.224463000	-1.418270000	-0.255116000
C	1.753269000	1.806466000	-0.206423000
C	2.617106000	0.256036000	2.830036000
C	0.243168000	-0.077882000	3.032462000
C	1.693087000	-2.661561000	0.497510000
C	3.741203000	-1.280848000	0.012681000
C	2.008276000	-1.629489000	-1.771342000
C	3.018599000	2.336959000	0.509360000
C	2.022710000	1.810800000	-1.727494000
C	0.589346000	2.770179000	0.127707000
H	3.504663000	0.383458000	2.226627000
C	2.751924000	0.283099000	4.211993000
C	0.381899000	-0.052619000	4.424072000
N	-1.089839000	-0.375574000	2.468184000
H	1.830782000	-2.540814000	1.579498000
H	0.615610000	-2.780160000	0.306217000
C	2.436668000	-3.925536000	0.023093000
C	4.472662000	-2.552229000	-0.468054000
H	3.938357000	-1.159922000	1.081392000
H	4.146267000	-0.405862000	-0.508863000
C	2.745614000	-2.900426000	-2.236987000
H	2.381668000	-0.773424000	-2.339117000
H	0.943090000	-1.722597000	-1.994807000
H	2.855051000	2.383057000	1.589915000
H	3.874653000	1.677965000	0.324206000
C	3.350149000	3.760262000	0.011650000
C	2.324710000	3.245020000	-2.204810000
H	2.891356000	1.181185000	-1.948073000
H	1.173897000	1.398111000	-2.281336000
C	0.917600000	4.194760000	-0.359776000
H	0.412182000	2.779601000	1.212355000
H	-0.335930000	2.424649000	-0.353785000
H	3.732197000	0.422142000	4.656245000
C	1.625735000	0.133674000	5.014168000
H	-0.476300000	-0.195537000	5.071343000
C	-2.118239000	0.504890000	3.078422000
C	-1.389783000	-1.804293000	2.786571000
H	2.043149000	-4.784918000	0.578974000
C	3.938030000	-3.767496000	0.301800000
C	2.205227000	-4.120894000	-1.480811000
H	5.541468000	-2.422903000	-0.262217000
C	4.248294000	-2.746896000	-1.972003000
H	2.566611000	-3.019081000	-3.311999000
H	4.255153000	4.093594000	0.532424000
C	2.183172000	4.697414000	0.347150000
C	3.590746000	3.750587000	-1.502345000
C	1.145565000	4.171754000	-1.877875000
H	2.485134000	3.216291000	-3.288738000
H	0.071112000	4.849070000	-0.117277000
H	1.710529000	0.151278000	6.096009000
H	-3.094388000	0.214143000	2.695298000
H	-1.906990000	1.544509000	2.822333000
H	-2.147633000	0.408276000	4.166535000
H	-1.437238000	-1.955395000	3.869776000
H	-0.598933000	-2.434514000	2.379579000
H	-2.342857000	-2.090927000	2.346390000

H	4.472142000	-4.672861000	-0.008307000
H	4.117143000	-3.648734000	1.378276000
H	1.135265000	-4.257061000	-1.688550000
H	2.710427000	-5.031525000	-1.822673000
H	4.650238000	-1.893623000	-2.533133000
H	4.787590000	-3.635360000	-2.320211000
H	2.413286000	5.718351000	0.021701000
H	2.024446000	4.739466000	1.432461000
H	4.446086000	3.110075000	-1.751761000
H	3.841034000	4.759779000	-1.849245000
H	0.238076000	3.836698000	-2.398677000
H	1.354310000	5.185275000	-2.239153000
C	-3.414895000	-0.315637000	-0.066828000
C	-4.123742000	-1.509653000	0.584964000
C	-4.110280000	0.976559000	0.243319000
H	-3.463854000	-0.451260000	-1.151715000
C	-5.577433000	-1.628579000	0.101468000
H	-4.137056000	-1.421039000	1.678952000
H	-3.598664000	-2.439824000	0.347249000
H	-4.275886000	1.154721000	1.308313000
C	-6.353691000	-0.365183000	0.387220000
H	-6.092026000	-2.454338000	0.606284000
H	-5.607719000	-1.855437000	-0.971707000
H	-7.354665000	-0.335168000	-0.047491000
H	-6.420565000	-0.127868000	1.450929000
H	-5.694226000	0.790396000	-1.159175000
O	-5.650016000	0.817290000	-0.185783000
C	-3.697428000	2.237289000	-0.469629000
H	-2.727458000	2.557935000	-0.079701000
H	-3.582338000	2.077566000	-1.545638000
H	-4.409927000	3.045242000	-0.286236000

4’$_{OH6E}$

Sum of electronic and zero-point Energies = -2163.766765
Sum of electronic and thermal Free Energies = -2163.838402
Esolv = -2165.2309776

C	-1.292669000	-0.319912000	-1.849983000
C	-1.273268000	0.800299000	-2.677654000
H	-1.154863000	1.799081000	-2.271480000
Au	-1.223746000	-0.207431000	0.192729000
C	-1.469908000	-1.593599000	-2.400400000
H	-1.506555000	-2.478225000	-1.769727000
C	-1.591350000	-1.741409000	-3.784123000
H	-1.716001000	-2.735215000	-4.204547000
C	-1.554003000	-0.624947000	-4.618029000
H	-1.649686000	-0.742582000	-5.692815000
C	-1.398468000	0.641906000	-4.062826000
H	-1.370528000	1.519435000	-4.702674000
P	1.204537000	0.066101000	0.378215000
C	1.365301000	0.081247000	2.208812000
C	2.224463000	-1.418270000	-0.255116000
C	1.753269000	1.806466000	-0.206423000
C	2.617106000	0.256036000	2.830036000
C	0.243168000	-0.077882000	3.032462000
C	1.693087000	-2.661561000	0.497510000
C	3.741203000	-1.280848000	0.012681000
C	2.008276000	-1.629489000	-1.771342000
C	3.018599000	2.336959000	0.509360000
C	2.022710000	1.810800000	-1.727494000
C	0.589346000	2.770179000	0.127707000
H	3.504663000	0.383458000	2.226627000
C	2.751924000	0.283099000	4.211993000
C	0.381899000	-0.052619000	4.424072000
N	-1.089839000	-0.375574000	2.468184000
H	1.830782000	-2.540814000	1.579498000
H	0.615610000	-2.780160000	0.306217000
C	2.436668000	-3.925536000	0.023093000
C	4.472662000	-2.552229000	-0.468054000
H	3.938357000	-1.159922000	1.081392000
H	4.146267000	-0.405862000	-0.508863000
C	2.745614000	-2.900426000	-2.236987000
H	2.381668000	-0.773424000	-2.339117000
H	0.943090000	-1.722597000	-1.994807000
H	2.855051000	2.383057000	1.589915000
H	3.874653000	1.677965000	0.324206000
C	3.350149000	3.760262000	0.011650000
C	2.324710000	3.245020000	-2.204810000
H	2.891356000	1.181185000	-1.948073000
H	1.173897000	1.398111000	-2.281336000
C	0.917600000	4.194760000	-0.359776000
H	0.412182000	2.779601000	1.212355000
H	-0.335930000	2.424649000	-0.353785000
H	3.732197000	0.422142000	4.656245000
C	1.625735000	0.133674000	5.014168000
H	-0.476300000	-0.195537000	5.071343000
C	-2.118239000	0.504890000	3.078422000
C	-1.389783000	-1.804293000	2.786571000
H	2.043149000	-4.784918000	0.578974000
C	3.938030000	-3.767496000	0.301800000
C	2.205227000	-4.120894000	-1.480811000
H	5.541468000	-2.422903000	-0.262217000
C	4.248294000	-2.746896000	-1.972003000
H	2.566611000	-3.019081000	-3.311999000
H	4.255153000	4.093594000	0.532424000
C	2.183172000	4.697414000	0.347150000
C	3.590746000	3.750587000	-1.502345000
C	1.145565000	4.171754000	-1.877875000
H	2.485134000	3.216291000	-3.288738000
H	0.071112000	4.849070000	-0.117277000
H	1.710529000	0.151278000	6.096009000
H	-3.094388000	0.214143000	2.695298000
H	-1.906990000	1.544509000	2.822333000
H	-2.147633000	0.408276000	4.166535000
H	-1.437238000	-1.955395000	3.869776000
H	-0.598933000	-2.434514000	2.379579000
H	-2.342857000	-2.090927000	2.346390000
H	4.472142000	-4.672861000	-0.008307000
H	4.117143000	-3.648734000	1.378276000
H	1.135265000	-4.257061000	-1.688550000
H	2.710427000	-5.031525000	-1.822673000
H	4.650238000	-1.893623000	-2.533133000
H	4.787590000	-3.635360000	-2.320211000
H	2.413286000	5.718351000	0.021701000
H	2.024446000	4.739466000	1.432461000
H	4.446086000	3.110075000	-1.751761000
H	3.841034000	4.759779000	-1.849245000
H	0.238076000	3.836698000	-2.398677000
H	1.354310000	5.185275000	-2.239153000
C	-3.414895000	-0.315637000	-0.066828000
C	-4.123742000	-1.509653000	0.584964000
C	-4.110280000	0.976559000	0.243319000
H	-3.463854000	-0.451260000	-1.151715000
C	-5.577433000	-1.628579000	0.101468000
H	-4.137056000	-1.421039000	1.678952000
H	-3.598664000	-2.439824000	0.347249000
H	-4.275886000	1.154721000	1.308313000
C	-6.353691000	-0.365183000	0.387220000
H	-6.092026000	-2.454338000	0.606284000
H	-5.607719000	-1.855437000	-0.971707000
H	-7.354665000	-0.335168000	-0.047491000

H	-6.420565000	-0.127868000	1.450929000
H	-5.694226000	0.790396000	-1.159175000
O	-5.650016000	0.817290000	-0.185783000
C	-3.697428000	2.237289000	-0.469629000
H	-2.727458000	2.557935000	-0.079701000
H	-3.582338000	2.077566000	-1.545638000
H	-4.409927000	3.045242000	-0.286236000

5_{O5}

Sum of electronic and zero-point Energies = -2124.246872
Sum of electronic and thermal Free Energies = -2124.318565
Esolv = -2125.510931

C	1.365442000	-1.591166000	-1.268422000
C	1.155571000	-2.971772000	-1.334747000
H	0.876459000	-3.534240000	-0.447754000
Au	1.143489000	-0.622024000	0.505951000
C	1.765844000	-0.899258000	-2.413313000
H	1.978459000	0.164778000	-2.371886000
C	1.918041000	-1.580327000	-3.624034000
H	2.228665000	-1.031604000	-4.508950000
C	1.684025000	-2.951309000	-3.696580000
H	1.806953000	-3.477885000	-4.638069000
C	1.306990000	-3.645080000	-2.548756000
H	1.137817000	-4.717575000	-2.588744000
P	-1.091346000	0.369416000	0.232983000
C	-1.290187000	1.140994000	1.891268000
C	-1.107307000	1.810683000	-1.010266000
C	-2.504499000	-0.894946000	0.060707000
C	-2.463239000	1.830168000	2.253174000
C	-0.263172000	1.051203000	2.842060000
C	0.251653000	2.534170000	-0.839318000
C	-2.226929000	2.853010000	-0.793557000
C	-1.217052000	1.260191000	-2.449968000
C	-3.916465000	-0.289894000	-0.100354000
C	-2.225468000	-1.816074000	-1.150561000
C	-2.470806000	-1.759439000	1.344959000
H	-3.272666000	1.917043000	1.540893000
C	-2.615777000	2.414604000	3.503299000
C	-0.416487000	1.650444000	4.097632000
N	0.993831000	0.330386000	2.570919000
H	0.329062000	2.941252000	0.178017000
H	1.083067000	1.829254000	-0.968564000
C	0.381415000	3.676418000	-1.863371000
C	-2.099257000	3.990103000	-1.828064000
H	-2.151156000	3.286406000	0.208852000
H	-3.215501000	2.388114000	-0.881958000
C	-1.068042000	2.407698000	-3.466369000
H	-2.195810000	0.788496000	-2.594061000
H	-0.456239000	0.493210000	-2.632121000
H	-4.164388000	0.357004000	0.746748000
H	-3.969279000	0.318234000	-1.010735000
C	-4.966802000	-1.416592000	-0.176582000
C	-3.281351000	-2.934109000	-1.225504000
H	-2.240661000	-1.244014000	-2.083109000
H	-1.228617000	-2.256743000	-1.067685000
C	-3.526731000	-2.876961000	1.262580000
H	-2.663705000	-1.138485000	2.227853000
H	-1.472264000	-2.204293000	1.467756000
H	-3.534981000	2.937299000	3.748138000
C	-1.581586000	2.328190000	4.430429000
H	0.379388000	1.591377000	4.832344000
C	1.116733000	-0.789639000	3.545433000
C	2.141176000	1.272936000	2.713222000
H	1.354439000	4.161773000	-1.719823000
C	-0.747379000	4.692739000	-1.645649000
C	0.290236000	3.098712000	-3.282767000
H	-2.914472000	4.702736000	-1.653644000
C	-2.198548000	3.422203000	-3.249780000
H	-1.135745000	1.984107000	-4.475642000
H	-5.955982000	-0.955262000	-0.284133000
C	-4.919777000	-2.248894000	1.112462000
C	-4.675885000	-2.315226000	-1.385140000
C	-3.232472000	-3.777112000	0.055476000
H	-3.051035000	-3.563365000	-2.093576000
H	-3.481840000	-3.464519000	2.187751000
H	-1.678246000	2.783951000	5.410874000
H	2.033740000	-1.345369000	3.353295000
H	0.260371000	-1.456862000	3.435204000
H	1.147155000	-0.415838000	4.573688000
H	2.263835000	1.584621000	3.755686000
H	1.951711000	2.150578000	2.094796000
H	3.055502000	0.802353000	2.354100000
H	-0.652579000	5.519651000	-2.359952000
H	-0.677661000	5.130667000	-0.641301000
H	1.107388000	2.386713000	-3.457753000
H	0.406706000	3.900191000	-4.022443000
H	-3.174064000	2.942321000	-3.402751000
H	-2.126078000	4.233647000	-3.984195000
H	-5.684033000	-3.034793000	1.079891000
H	-5.149363000	-1.619112000	1.982091000
H	-4.730548000	-1.733531000	-2.314586000
H	-5.435022000	-3.103464000	-1.459350000
H	-2.246804000	-4.248828000	0.163556000
H	-3.968338000	-4.588808000	0.003173000
C	3.073648000	-1.409104000	0.836205000
H	3.043750000	-2.479686000	0.617900000
H	3.276108000	-1.280510000	1.904965000
C	4.174892000	-0.747592000	0.037511000
C	5.598458000	-1.135634000	0.444102000
H	4.037977000	-0.962576000	-1.032544000
C	5.436558000	1.228300000	0.082617000
C	6.401937000	0.047554000	-0.101202000
H	5.675089000	-1.191131000	1.537039000
H	5.908441000	-2.098307000	0.029734000
H	5.441606000	1.907649000	-0.777847000
H	5.667635000	1.815967000	0.979138000
H	7.351187000	0.199438000	0.418521000
H	6.626226000	-0.105597000	-1.161667000
O	4.123958000	0.669796000	0.248564000

5_{O5Z}

Sum of electronic and zero-point Energies = -2163.485240
Sum of electronic and thermal Free Energies = -2163.561322
Esolv = -2164.8142625

Au	1.009066000	-0.285206000	0.174130000
P	-1.361090000	0.009476000	0.340938000
C	-1.846844000	-0.392022000	2.068055000
C	-1.858814000	1.820260000	0.027823000
C	-2.209647000	-1.277941000	-0.767606000
C	-3.185464000	-0.259299000	2.481805000
C	-0.897963000	-0.819033000	3.018533000
C	-1.407866000	2.628211000	1.268963000
C	-3.363929000	2.065977000	-0.214379000
C	-1.072878000	2.344770000	-1.198457000
C	-3.724062000	-1.466264000	-0.529088000
C	-1.971674000	-0.907100000	-2.248621000
C	-1.513352000	-2.630325000	-0.474349000
H	-3.934799000	0.071797000	1.773902000

C	-3.583004000	-0.541456000	3.782698000
C	-1.311306000	-1.104554000	4.325445000
N	0.504978000	-0.966628000	2.682885000
H	-1.940928000	2.284946000	2.162612000
H	-0.335046000	2.465753000	1.448773000
C	-1.677622000	4.128389000	1.054391000
C	-3.626421000	3.571468000	-0.418219000
H	-3.959722000	1.715950000	0.635033000
H	-3.701310000	1.518677000	-1.101954000
C	-1.346419000	3.844818000	-1.408200000
H	-1.351178000	1.793841000	-2.102735000
H	0.000607000	2.183271000	-1.050700000
H	-3.906686000	-1.767600000	0.507869000
H	-4.266544000	-0.530825000	-0.702556000
C	-4.274151000	-2.559853000	-1.466656000
C	-2.518671000	-2.015581000	-3.166238000
H	-2.480538000	0.033748000	-2.487713000
H	-0.900799000	-0.754989000	-2.436159000
C	-2.065672000	-3.730682000	-1.398007000
H	-1.671531000	-2.910777000	0.575387000
H	-0.428570000	-2.534756000	-0.627785000
H	-4.624407000	-0.426889000	4.066871000
C	-2.638765000	-0.970628000	4.711132000
H	-0.579276000	-1.436027000	5.055812000
C	0.951658000	-2.359409000	2.834231000
C	1.347790000	-0.057318000	3.478090000
H	-1.354146000	4.669990000	1.951654000
C	-3.180948000	4.346107000	0.829845000
C	-0.895246000	4.626960000	-0.167665000
H	-4.701892000	3.713878000	-0.579202000
C	-2.846451000	4.067913000	-1.642603000
H	-0.779164000	4.178189000	-2.285774000
H	-5.351150000	-2.657559000	-1.284347000
C	-3.573743000	-3.890512000	-1.162434000
C	-4.027639000	-2.168738000	-2.929986000
C	-1.808985000	-3.341729000	-2.860739000
H	-2.332567000	-1.725946000	-4.207563000
H	-1.550646000	-4.671106000	-1.165969000
H	-2.932244000	-1.198245000	5.731407000
H	1.984835000	-2.448371000	2.484980000
H	0.319061000	-3.015672000	2.233121000
H	0.923019000	-2.701878000	3.878202000
H	1.326096000	-0.294104000	4.551110000
H	1.005096000	0.970681000	3.341740000
H	2.386653000	-0.131745000	3.141985000
H	-3.391167000	5.415088000	0.703211000
H	-3.748774000	4.013059000	1.708454000
H	0.183521000	4.497443000	-0.006980000
H	-1.067497000	5.700432000	-0.313256000
H	-3.174558000	3.535349000	-2.544868000
H	-3.048691000	5.132635000	-1.811638000
H	-3.972662000	-4.683896000	-1.806205000
H	-3.767372000	-4.194661000	-0.125559000
H	-4.546105000	-1.230486000	-3.167149000
H	-4.439006000	-2.935266000	-3.598038000
H	-0.730605000	-3.247111000	-3.046230000
H	-2.177512000	-4.129098000	-3.529479000
C	4.340581000	0.182170000	0.429498000
H	3.915717000	1.183940000	0.592444000
C	3.193524000	-0.813419000	0.216306000
C	5.468313000	0.258565000	-0.599909000
H	5.660590000	-0.726796000	-1.036369000
H	5.251341000	0.951134000	-1.414354000
C	6.390559000	-0.087434000	1.560678000
H	6.740178000	0.433035000	2.458036000
H	6.857312000	-1.081282000	1.536790000
C	6.650562000	0.687675000	0.271504000
H	6.616954000	1.766790000	0.457602000
H	7.621249000	0.455099000	-0.173391000
C	3.568472000	-2.177608000	-0.324511000
H	4.343060000	-2.594779000	0.330104000
H	2.721103000	-2.869599000	-0.307968000
H	3.963983000	-2.144033000	-1.341022000
H	2.836326000	-0.970272000	1.248944000
C	2.255790000	0.133237000	-1.466276000
C	2.569299000	1.485528000	-1.664069000
C	2.195845000	-0.724783000	-2.571085000
C	2.807787000	1.967056000	-2.950967000
H	2.624299000	2.171984000	-0.824862000
C	2.436134000	-0.233271000	-3.854057000
H	1.953810000	-1.774817000	-2.442540000
C	2.746813000	1.110989000	-4.049311000
H	3.046639000	3.017853000	-3.089148000
H	2.381548000	-0.910533000	-4.701701000
H	2.943543000	1.487704000	-5.048205000
O	4.966543000	-0.244298000	1.647684000

5_{O5E}

Sum of electronic and zero-point Energies = -2163.497720

Sum of electronic and thermal Free Energies = -2163.570712

Esolv = -2164.80982983

Au	1.175903000	-0.602460000	0.362611000
P	-1.180896000	0.237092000	0.321683000
C	-1.503540000	0.386470000	2.125765000
C	-1.273210000	2.023833000	-0.354074000
C	-2.498914000	-0.959452000	-0.362599000
C	-2.729824000	0.869822000	2.620499000
C	-0.521805000	0.014110000	3.056914000
C	0.042330000	2.712349000	0.084968000
C	-2.446991000	2.875539000	0.180433000
C	-1.350827000	2.002936000	-1.896924000
C	-3.945087000	-0.417111000	-0.358876000
C	-2.127052000	-1.364950000	-1.808768000
C	-2.438228000	-2.227798000	0.522516000
H	-3.506502000	1.161495000	1.927069000
C	-2.977763000	0.995814000	3.980672000
C	-0.777367000	0.140931000	4.427652000
N	0.750627000	-0.605755000	2.641527000
H	0.099580000	2.741388000	1.181786000
H	0.907339000	2.135954000	-0.268033000
C	0.116839000	4.142889000	-0.479100000
C	-2.375851000	4.304471000	-0.396643000
H	-2.399769000	2.943139000	1.271654000
H	-3.409376000	2.422955000	-0.085238000
C	-1.254988000	3.436046000	-2.453640000
H	-2.305657000	1.568756000	-2.213853000
H	-0.555963000	1.379530000	-2.319401000
H	-4.255198000	-0.141985000	0.653900000
H	-4.019439000	0.478961000	-0.985593000
C	-4.914298000	-1.493642000	-0.888047000
C	-3.103785000	-2.434852000	-2.330920000
H	-2.156827000	-0.497640000	-2.474315000
H	-1.105351000	-1.753065000	-1.840656000
C	-3.415347000	-3.295115000	-0.004066000
H	-2.689784000	-1.980944000	1.561335000
H	-1.416547000	-2.634383000	0.515123000
H	-3.934952000	1.374982000	4.324283000
C	-1.990116000	0.633834000	4.890620000
H	-0.033479000	-0.161006000	5.156191000
C	0.670405000	-2.052060000	2.999939000

C	1.894760000	0.020089000	3.349100000
H	1.061654000	4.596156000	-0.154834000
C	-1.065312000	4.966018000	0.048958000
C	0.063406000	4.086588000	-2.012350000
H	-3.229200000	4.871529000	-0.005420000
C	-2.438005000	4.259467000	-1.928512000
H	-1.294459000	3.382788000	-3.548293000
H	-5.929394000	-1.078784000	-0.869672000
C	-4.843566000	-2.732510000	0.015575000
C	-4.532252000	-1.875612000	-2.323648000
C	-3.030872000	-3.680621000	-1.438458000
H	-2.809956000	-2.693908000	-3.355154000
H	-3.353004000	-4.173921000	0.649430000
H	-2.161579000	0.723124000	5.958783000
H	1.571788000	-2.562587000	2.666333000
H	-0.199064000	-2.493660000	2.512139000
H	0.566487000	-2.175482000	4.083829000
H	1.797131000	-0.054377000	4.435913000
H	1.965247000	1.071588000	3.066904000
H	2.805468000	-0.497122000	3.058712000
H	-1.010483000	5.992207000	-0.334175000
H	-1.024652000	5.032854000	1.144014000
H	0.919061000	3.520549000	-2.403832000
H	0.140262000	5.098656000	-2.427966000
H	-3.386178000	3.816575000	-2.260185000
H	-2.403832000	5.277057000	-2.336368000
H	-5.551082000	-3.493970000	-0.334184000
H	-5.137444000	-2.473848000	1.041403000
H	-4.602142000	-1.000129000	-2.982369000
H	-5.234427000	-2.622518000	-2.713741000
H	-2.018756000	-4.105802000	-1.460510000
H	-3.709178000	-4.456463000	-1.813957000
C	3.281805000	-1.139520000	0.352525000
C	1.431698000	-0.914581000	-1.633393000
C	1.691131000	0.110561000	-2.542170000
C	1.358284000	-2.236144000	-2.088043000
C	1.841338000	-0.180264000	-3.901574000
H	1.787927000	1.139446000	-2.211584000
C	1.506041000	-2.518673000	-3.447017000
H	1.193326000	-3.054994000	-1.392893000
C	1.744694000	-1.491615000	-4.358254000
H	2.041479000	0.628072000	-4.599527000
H	1.443575000	-3.548606000	-3.787142000
H	1.867204000	-1.714045000	-5.413778000
C	3.569891000	-2.437434000	1.097189000
H	3.517225000	-2.337962000	2.186884000
H	2.907968000	-3.252836000	0.787200000
H	4.597418000	-2.740644000	0.870300000
H	3.485871000	-1.322490000	-0.703068000
C	4.230798000	-0.003084000	0.776271000
C	4.156392000	1.285856000	-0.058632000
H	4.121064000	0.221954000	1.843051000
C	6.278557000	0.302529000	-0.345748000
C	5.220102000	1.061596000	-1.135531000
H	4.428396000	2.145813000	0.563436000
H	3.159544000	1.471357000	-0.464275000
H	6.939855000	0.996227000	0.194547000
H	6.901056000	-0.355889000	-0.958711000
H	5.598139000	1.991735000	-1.567819000
H	4.825510000	0.443484000	-1.948996000
O	5.562000000	-0.503910000	0.590432000

5’$_{O5E}$

Sum of electronic and zero-point Energies = -2163.507892

Sum of electronic and thermal Free Energies = -2163.581156

Esolv = -2164.8194512

C	-1.498577000	0.845846000	-1.590088000
C	-1.423985000	2.154653000	-2.074174000
H	-1.212770000	2.982891000	-1.404262000
Au	-1.162159000	0.476613000	0.385385000
C	-1.780220000	-0.195211000	-2.477639000
H	-1.856311000	-1.219882000	-2.127876000
C	-1.981372000	0.071707000	-3.834254000
H	-2.205789000	-0.748113000	-4.511113000
C	-1.902841000	1.376083000	-4.315365000
H	-2.064601000	1.581630000	-5.369056000
C	-1.621674000	2.415418000	-3.431911000
H	-1.560604000	3.438525000	-3.792318000
P	1.216626000	-0.234566000	0.301153000
C	1.534428000	-0.477124000	2.097019000
C	1.460586000	-1.944855000	-0.501838000
C	2.425955000	1.129281000	-0.258444000
C	2.802378000	-0.857754000	2.576985000
C	0.517660000	-0.259810000	3.038273000
C	0.223800000	-2.783880000	-0.095426000
C	2.720730000	-2.716761000	-0.050096000
C	1.497187000	-1.800657000	-2.039980000
C	3.919085000	0.734761000	-0.267258000
C	2.040716000	1.608882000	-1.677606000
C	2.223876000	2.307774000	0.725830000
H	3.605396000	-1.038066000	1.875552000
C	3.060543000	-1.016132000	3.931772000
C	0.780411000	-0.426701000	4.403420000
N	-0.848540000	0.135913000	2.644818000
H	0.201083000	-2.903895000	0.996523000
H	-0.699609000	-2.264951000	-0.385318000
C	0.269332000	-4.168570000	-0.766497000
C	2.768840000	-4.099328000	-0.732387000
H	2.705107000	-2.866150000	1.034280000
H	3.629641000	-2.155940000	-0.296703000
C	1.525512000	-3.190957000	-2.701756000
H	2.397868000	-1.252554000	-2.338773000
H	0.634023000	-1.228677000	-2.398199000
H	4.243947000	0.408287000	0.725079000
H	4.090884000	-0.093389000	-0.964666000
C	4.781446000	1.943709000	-0.684650000
C	2.907479000	2.813149000	-2.089023000
H	2.176663000	0.804881000	-2.406828000
H	0.984713000	1.887458000	-1.709731000
C	3.092682000	3.507466000	0.306134000
H	2.490092000	2.002850000	1.744953000
H	1.164924000	2.605474000	0.738638000
H	4.051635000	-1.308756000	4.263719000
C	2.040025000	-0.799370000	4.852297000
H	-0.003237000	-0.265437000	5.135965000
C	-1.193850000	1.423138000	3.310128000
C	-1.790908000	-0.933524000	3.073851000
H	-0.621542000	-4.732340000	-0.463291000
C	1.534852000	-4.912696000	-0.319766000
C	0.283231000	-3.993836000	-2.291688000
H	3.679143000	-4.612235000	-0.399161000
C	2.792049000	-3.934271000	-2.257403000
H	1.536056000	-3.053861000	-3.789688000
H	5.833123000	1.632678000	-0.675077000
C	4.570546000	3.090840000	0.313560000
C	4.385743000	2.403664000	-2.093291000
C	2.692016000	3.967406000	-1.101578000

H	2.603590000	3.123736000	-3.095874000
H	2.932066000	4.319798000	1.025495000
H	2.218901000	-0.919766000	5.916149000
H	-2.191981000	1.734796000	3.004869000
H	-0.469404000	2.183171000	3.013273000
H	-1.181006000	1.325755000	4.399944000
H	-1.760055000	-1.076965000	4.158523000
H	-1.517989000	-1.868669000	2.582857000
H	-2.806978000	-0.661248000	2.790540000
H	1.567842000	-5.908003000	-0.779189000
H	1.524104000	-5.064728000	0.767514000
H	-0.628756000	-3.482032000	-2.626051000
H	0.291196000	-4.975165000	-2.781359000
H	3.686858000	-3.379557000	-2.568538000
H	2.844958000	-4.917369000	-2.740844000
H	5.202334000	3.945298000	0.042465000
H	4.873446000	2.779853000	1.322096000
H	4.555750000	1.598038000	-2.819431000
H	5.013322000	3.248005000	-2.403610000
H	1.641780000	4.287769000	-1.112950000
H	3.291544000	4.836646000	-1.398232000
C	-3.189781000	1.095547000	0.661701000
C	-4.244665000	0.365930000	-0.164986000
H	-4.155588000	0.661204000	-1.220864000
C	-5.757091000	-1.453646000	-0.358368000
C	-6.459069000	-0.275332000	0.317563000
H	-5.927710000	-1.435701000	-1.440121000
H	-6.096416000	-2.422123000	0.018191000
H	-6.743597000	-0.514156000	1.351515000
H	-7.360925000	0.046112000	-0.214963000
C	-4.287973000	-1.158530000	-0.053507000
H	-4.042652000	-1.471560000	0.969387000
H	-3.597948000	-1.657930000	-0.737601000
O	-5.517877000	0.801721000	0.332801000
C	-3.343619000	2.607775000	0.561334000
H	-2.592781000	3.155114000	1.141957000
H	-3.288877000	2.949029000	-0.474934000
H	-4.332618000	2.885803000	0.941324000
H	-3.361160000	0.790785000	1.701647000

5$_{O6}$
Sum of electronic and zero-point Energies = -2124.235207
Sum of electronic and thermal Free Energies = -2124.305966
Esolv = -2125.5110876

C	1.684842000	-0.037954000	-1.664272000
C	1.829219000	-1.247919000	-2.348667000
H	1.762346000	-2.195813000	-1.821918000
Au	1.292172000	-0.027947000	0.331819000
C	1.811895000	1.165337000	-2.362041000
H	1.732227000	2.117848000	-1.845819000
C	2.057637000	1.155949000	-3.737534000
H	2.153299000	2.098878000	-4.268796000
C	2.188302000	-0.050250000	-4.421486000
H	2.385359000	-0.054953000	-5.489118000
C	2.076964000	-1.250526000	-3.723400000
H	2.190766000	-2.197939000	-4.242845000
P	-1.183514000	0.013083000	0.295260000
C	-1.517585000	0.008290000	2.104805000
C	-1.876636000	1.667892000	-0.348892000
C	-1.959841000	-1.593902000	-0.374178000
C	-2.830460000	0.024615000	2.613709000
C	-0.459681000	-0.021647000	3.025816000
C	-0.955683000	2.758858000	0.253917000
C	-3.329766000	1.992249000	0.061618000
C	-1.780617000	1.718961000	-1.890395000
C	-3.476984000	-1.758001000	-0.139043000
C	-1.683926000	-1.724128000	-1.889928000
C	-1.222200000	-2.738147000	0.366226000
H	-3.667469000	0.052953000	1.929453000
C	-3.091530000	0.008789000	3.977552000
C	-0.727060000	-0.036497000	4.399634000
N	0.953245000	-0.038928000	2.599737000
H	-1.024016000	2.739804000	1.350045000
H	0.091550000	2.559927000	-0.013192000
C	-1.360838000	4.150387000	-0.265289000
C	-3.733016000	3.382896000	-0.471282000
H	-3.424608000	2.002914000	1.151763000
H	-4.020474000	1.235039000	-0.328056000
C	-2.166131000	3.120048000	-2.400448000
H	-2.470537000	0.990692000	-2.328476000
H	-0.772332000	1.456113000	-2.227703000
H	-3.709947000	-1.705754000	0.928536000
H	-4.032352000	-0.959294000	-0.645047000
C	-3.947811000	-3.129265000	-0.666612000
C	-2.147772000	-3.101488000	-2.399343000
H	-2.224416000	-0.949403000	-2.440994000
H	-0.620274000	-1.583091000	-2.102365000
C	-1.700318000	-4.106444000	-0.152369000
H	-1.408025000	-2.669179000	1.445827000
H	-0.137074000	-2.646284000	0.212341000
H	-4.117137000	0.021632000	4.332591000
C	-2.030851000	-0.022415000	4.877101000
H	0.087038000	-0.060200000	5.116196000
C	1.595755000	-1.280712000	3.116474000
C	1.632580000	1.168675000	3.147513000
H	-0.689833000	4.895905000	0.178943000
C	-2.811060000	4.446444000	0.140359000
C	-1.234074000	4.177715000	-1.794592000
H	-4.770264000	3.574858000	-0.171273000
C	-3.617172000	3.417673000	-2.000411000
H	-2.072157000	3.124555000	-3.492962000
H	-5.026633000	-3.211469000	-0.487315000
C	-3.210300000	-4.243970000	0.088165000
C	-3.658000000	-3.243330000	-2.168293000
C	-1.402290000	-4.215763000	-1.653506000
H	-1.927600000	-3.160539000	-3.471972000
H	-1.163297000	-4.891434000	0.394098000
H	-2.212553000	-0.035212000	5.947187000
H	2.630175000	-1.330802000	2.776863000
H	1.049458000	-2.146116000	2.738515000
H	1.589633000	-1.305240000	4.210529000
H	1.617553000	1.170709000	4.241748000
H	1.120498000	2.060304000	2.782823000
H	2.670645000	1.188783000	2.817054000
H	-3.102555000	5.444239000	-0.209148000
H	-2.907578000	4.452070000	1.234011000
H	-0.195409000	3.987025000	-2.094682000
H	-1.495355000	5.172512000	-2.175345000
H	-4.294401000	2.681195000	-2.452171000
H	-3.921714000	4.401508000	-2.377654000
H	-3.557156000	-5.225351000	-0.257228000
H	-3.431627000	-4.189072000	1.162164000
H	-4.201871000	-2.467314000	-2.722474000
H	-4.011610000	-4.210118000	-2.546828000
H	-0.321885000	-4.139238000	-1.833754000
H	-1.716469000	-5.197278000	-2.028745000
C	4.125679000	-1.261309000	0.033263000
H	4.122501000	-1.278770000	-1.066606000
H	3.661872000	-2.187219000	0.390442000
C	3.408443000	-0.020586000	0.558518000

C	4.126758000	1.238522000	0.079226000
H	3.502788000	-0.062662000	1.650487000
C	6.204934000	-0.176331000	0.018135000
C	5.611876000	1.152418000	0.473745000
H	4.047752000	1.321294000	-1.010588000
H	3.678272000	2.146847000	0.501661000
H	7.224576000	-0.306780000	0.390361000
H	6.237579000	-0.218650000	-1.083952000
H	6.172624000	1.985422000	0.033512000
H	5.715508000	1.232404000	1.563292000
O	5.457910000	-1.277124000	0.516632000

5$_{O6Z}$

Sum of electronic and zero-point Energies = -2163.503331
Sum of electronic and thermal Free Energies = -2163.574568
Esolv = -2164.8181261

C	1.461873000	-1.084910000	1.482624000
C	1.492226000	-0.371726000	2.680270000
H	1.450613000	0.711914000	2.685387000
Au	1.241885000	-0.202029000	-0.337855000
C	1.533665000	-2.481850000	1.513925000
H	1.522697000	-3.059970000	0.594062000
C	1.625705000	-3.153757000	2.733871000
H	1.680181000	-4.238842000	2.742298000
C	1.655273000	-2.439698000	3.930017000
H	1.734457000	-2.962736000	4.878134000
C	1.589468000	-1.049540000	3.898791000
H	1.618672000	-0.479859000	4.823544000
P	-1.205824000	0.225607000	-0.303785000
C	-1.448317000	0.817465000	-2.029610000
C	-2.330261000	-1.301644000	-0.108851000
C	-1.627729000	1.715520000	0.811577000
C	-2.718093000	1.199829000	-2.503698000
C	-0.371226000	0.874995000	-2.926603000
C	-1.978400000	-2.245123000	-1.285205000
C	-3.847817000	-1.015574000	-0.133364000
C	-1.991162000	-2.028728000	1.214512000
C	-2.899132000	2.499553000	0.414839000
C	-1.777125000	1.253447000	2.278125000
C	-0.422969000	2.682679000	0.699298000
H	-3.566091000	1.176655000	-1.833293000
C	-2.922364000	1.617224000	-3.811738000
C	-0.582822000	1.292747000	-4.246334000
N	0.995856000	0.473823000	-2.543091000
H	-2.204417000	-1.760905000	-2.242986000
H	-0.901328000	-2.467705000	-1.273682000
C	-2.775189000	-3.557783000	-1.172231000
C	-4.635944000	-2.336657000	-0.021540000
H	-4.137390000	-0.522446000	-1.065872000
H	-4.125444000	-0.353719000	0.695248000
C	-2.784660000	-3.344183000	1.319163000
H	-2.232461000	-1.397383000	2.074333000
H	-0.920326000	-2.240619000	1.267453000
H	-2.811203000	2.871595000	-0.610918000
H	-3.783826000	1.853662000	0.460974000
C	-3.094958000	3.705958000	1.356008000
C	-1.949376000	2.472531000	3.203514000
H	-2.660894000	0.613014000	2.375761000
H	-0.911006000	0.659908000	2.590133000
C	-0.616920000	3.894941000	1.627980000
H	-0.315559000	3.024025000	-0.339585000
H	0.507019000	2.163537000	0.968605000
H	-3.916290000	1.904693000	-4.139666000
C	-1.844862000	1.662534000	-4.690531000
H	0.242684000	1.328281000	-4.948956000
C	1.939232000	1.584141000	-2.841704000
C	1.366843000	-0.734279000	-3.334241000
H	-2.509773000	-4.198131000	-2.022391000
C	-4.278086000	-3.245469000	-1.205892000
C	-2.422555000	-4.262086000	0.144458000
H	-5.706357000	-2.099109000	-0.046833000
C	-4.288039000	-3.040847000	1.295799000
H	-2.516482000	-3.828922000	2.265523000
H	-4.009944000	4.229005000	1.052680000
C	-1.891838000	4.649480000	1.228523000
C	-3.225200000	3.227245000	2.807589000
C	-0.737297000	3.406017000	3.078285000
H	-2.034830000	2.111652000	4.235517000
H	0.254124000	4.553959000	1.527160000
H	-1.981434000	1.982594000	-5.718727000
H	2.952989000	1.282726000	-2.582663000
H	1.656506000	2.461746000	-2.258418000
H	1.928035000	1.845363000	-3.903735000
H	1.363417000	-0.518319000	-4.407669000
H	0.648423000	-1.529902000	-3.130773000
H	2.365610000	-1.067325000	-3.049958000
H	-4.855502000	-4.176352000	-1.152914000
H	-4.545256000	-2.760163000	-2.153892000
H	-1.352876000	-4.508425000	0.170132000
H	-2.968610000	-5.210178000	0.221950000
H	-4.562429000	-2.408516000	2.150257000
H	-4.864838000	-3.969044000	1.389998000
H	-2.028564000	5.525251000	1.874342000
H	-1.807349000	5.023342000	0.199733000
H	-4.101887000	2.575828000	2.918336000
H	-3.381533000	4.084691000	3.473398000
H	0.180097000	2.884802000	3.382558000
H	-0.851014000	4.261832000	3.754633000
C	3.369561000	-0.298779000	-0.520575000
H	3.446911000	-0.348588000	-1.614827000
C	4.068889000	1.018842000	-0.126326000
C	4.139831000	-1.503968000	0.008909000
H	4.140063000	-1.525129000	1.103163000
H	3.677366000	-2.441661000	-0.317931000
C	6.196586000	-0.072846000	-0.203173000
H	7.163931000	0.050609000	-0.697674000
H	6.363506000	0.035608000	0.879107000
C	5.585421000	-1.433038000	-0.511807000
H	5.594931000	-1.589801000	-1.598205000
H	6.192742000	-2.229144000	-0.065270000
O	5.381886000	0.986649000	-0.691645000
C	4.094898000	1.379110000	1.356078000
H	4.811385000	2.191908000	1.505398000
H	3.112793000	1.735974000	1.675401000
H	4.371722000	0.548026000	2.008130000
H	3.593626000	1.856313000	-0.648747000

5$_{O6E}$

Sum of electronic and zero-point Energies = -2163.513830
Sum of electronic and thermal Free Energies = -2163.584911
Esolv = -2164.8283372

C	1.605785000	0.314808000	-1.665229000
C	1.643703000	-0.782003000	-2.529068000
H	1.521086000	-1.792797000	-2.152899000
Au	1.251893000	0.068539000	0.326661000
C	1.795968000	1.599217000	-2.183158000
H	1.779575000	2.468771000	-1.532015000
C	2.009140000	1.782572000	-3.551250000

H	2.153973000	2.787442000	-3.938234000
C	2.042417000	0.687821000	-4.411629000
H	2.215416000	0.831154000	-5.473804000
C	1.860269000	-0.593022000	-3.896536000
H	1.893260000	-1.456863000	-4.554677000
P	-1.229825000	-0.047915000	0.318178000
C	-1.538947000	-0.182740000	2.127246000
C	-2.098046000	1.557683000	-0.235833000
C	-1.897376000	-1.663256000	-0.441566000
C	-2.844892000	-0.243346000	2.650498000
C	-0.470030000	-0.202946000	3.034925000
C	-1.580259000	2.669180000	0.710210000
C	-3.641449000	1.535745000	-0.184225000
C	-1.665590000	1.905099000	-1.679322000
C	-3.293941000	-2.098296000	0.055085000
C	-1.936608000	-1.542390000	-1.981571000
C	-0.889254000	-2.768600000	-0.041647000
H	-3.689957000	-0.237829000	1.975886000
C	-3.089390000	-0.316895000	4.015087000
C	-0.720487000	-0.283472000	4.410071000
N	0.937572000	-0.155623000	2.594572000
H	-1.872203000	2.454506000	1.745206000
H	-0.481079000	2.703399000	0.679786000
C	-2.150239000	4.035935000	0.289157000
C	-4.202761000	2.910227000	-0.603521000
H	-3.996715000	1.314363000	0.826446000
H	-4.034730000	0.762441000	-0.854359000
C	-2.230697000	3.276666000	-2.092190000
H	-2.022324000	1.146121000	-2.381581000
H	-0.575887000	1.916845000	-1.756044000
H	-3.285504000	-2.227811000	1.142009000
H	-4.046396000	-1.338327000	-0.184886000
C	-3.693126000	-3.440059000	-0.592741000
C	-2.318612000	-2.896132000	-2.608978000
H	-2.684239000	-0.797224000	-2.275480000
H	-0.969124000	-1.204953000	-2.370033000
C	-1.286032000	-4.113247000	-0.677698000
H	-0.865300000	-2.870576000	1.052033000
H	0.123073000	-2.493629000	-0.366434000
H	-4.110405000	-0.360424000	4.380750000
C	-2.017515000	-0.337516000	4.901875000
H	0.102363000	-0.306063000	5.116524000
C	1.605422000	-1.418020000	3.017268000
C	1.605575000	1.018374000	3.222228000
H	-1.770780000	4.797513000	0.981416000
C	-3.683466000	3.989298000	0.356940000
C	-1.705040000	4.360996000	-1.142604000
H	-5.297176000	2.862464000	-0.550604000
C	-3.763124000	3.237289000	-2.036006000
H	-1.900310000	3.487337000	-3.116409000
H	-4.691397000	-3.710902000	-0.228283000
C	-2.681735000	-4.520207000	-0.186568000
C	-3.715377000	-3.301590000	-2.120384000
C	-1.296957000	-3.968266000	-2.205959000
H	-2.324193000	-2.780189000	-3.699396000
H	-0.550114000	-4.870091000	-0.379629000
H	-2.185526000	-0.397517000	5.972591000
H	2.644078000	-1.412363000	2.689766000
H	1.090590000	-2.263798000	2.559767000
H	1.583260000	-1.531543000	4.105626000
H	1.609560000	0.939984000	4.313665000
H	1.074776000	1.926852000	2.933237000
H	2.637834000	1.076519000	2.878713000
H	-4.100815000	4.966315000	0.084907000
H	-4.012990000	3.778184000	1.382808000
H	-0.609684000	4.417083000	-1.196954000
H	-2.087706000	5.344445000	-1.441605000
H	-4.150633000	2.484920000	-2.735250000
H	-4.180159000	4.203150000	-2.346273000
H	-2.966091000	-5.486203000	-0.621019000
H	-2.679590000	-4.650390000	0.903583000
H	-4.457114000	-2.552088000	-2.425862000
H	-4.017154000	-4.251039000	-2.579118000
H	-0.297369000	-3.698635000	-2.571265000
H	-1.554483000	-4.927266000	-2.671573000
C	3.378203000	0.141558000	0.530096000
H	3.500765000	-0.132576000	1.586516000
C	4.186354000	-0.895251000	-0.261966000
H	4.133138000	-0.654874000	-1.335524000
C	3.956075000	1.541880000	0.327505000
H	3.828446000	1.845499000	-0.717130000
H	3.433190000	2.286853000	0.941096000
C	3.769040000	-2.337425000	-0.033801000
H	3.902806000	-2.609535000	1.018080000
H	2.723151000	-2.502813000	-0.308404000
H	4.394823000	-3.006365000	-0.629623000
C	6.149104000	0.430886000	-0.112449000
H	7.198874000	0.332604000	0.177392000
H	6.113700000	0.637214000	-1.195712000
C	5.456995000	1.546619000	0.657008000
H	5.611312000	1.387609000	1.731696000
H	5.899376000	2.517305000	0.403907000
O	5.545616000	-0.822720000	0.161243000

5'$_{O6E}$

Sum of electronic and zero-point Energies = -2163.505397
Sum of electronic and thermal Free Energies = -2163.576932
Esolv = -2164.8190634

C	1.374070000	0.036851000	1.927020000
C	1.305366000	1.251773000	2.612172000
H	1.203731000	2.190040000	2.075626000
Au	1.282083000	0.041608000	-0.108739000
C	1.566601000	-1.148558000	2.639427000
H	1.661165000	-2.099726000	2.123489000
C	1.647779000	-1.120633000	4.033257000
H	1.792432000	-2.049926000	4.577208000
C	1.555707000	0.087389000	4.720758000
H	1.627448000	0.107129000	5.803819000
C	1.389690000	1.271548000	4.006944000
H	1.336145000	2.222341000	4.529958000
P	-1.197015000	-0.023246000	-0.384315000
C	-1.329083000	-0.029126000	-2.217423000
C	-1.961738000	-1.681918000	0.178041000
C	-2.093451000	1.554223000	0.200478000
C	-2.574154000	-0.127813000	-2.867776000
C	-0.176412000	0.031982000	-3.013639000
C	-1.207921000	-2.780558000	-0.611927000
C	-3.477812000	-1.840012000	-0.072238000
C	-1.698062000	-1.895045000	1.686296000
C	-3.470732000	1.813421000	-0.449469000
C	-2.272318000	1.518350000	1.734789000
C	-1.165370000	2.733693000	-0.180191000
H	-3.480809000	-0.164514000	-2.280015000
C	-2.681253000	-0.175282000	-4.250948000
C	-0.290390000	-0.005354000	-4.408947000
N	1.170361000	0.198224000	-2.433316000
H	-1.379536000	-2.660039000	-1.688311000
H	-0.124980000	-2.688475000	-0.441081000
C	-1.682855000	-4.176813000	-0.170149000
C	-3.943497000	-3.242666000	0.370821000

H	-3.714147000	-1.723725000	-1.133605000
H	-4.037689000	-1.077944000	0.482426000
C	-2.163473000	-3.296972000	2.120681000
H	-2.229055000	-1.145306000	2.279739000
H	-0.635586000	-1.774190000	1.906831000
H	-3.369605000	1.880869000	-1.537355000
H	-4.165523000	0.995069000	-0.228467000
C	-4.060062000	3.141845000	0.068627000
C	-2.842319000	2.859276000	2.233355000
H	-2.971870000	0.719906000	2.005852000
H	-1.320525000	1.303145000	2.232808000
C	-1.750120000	4.064176000	0.327931000
H	-1.053485000	2.776106000	-1.272265000
H	-0.165423000	2.583203000	0.247589000
H	-3.658492000	-0.255100000	-4.716444000
C	-1.528512000	-0.114940000	-5.027215000
H	0.593026000	0.059998000	-5.034466000
C	1.646439000	1.562515000	-2.797184000
C	2.093734000	-0.820719000	-2.994634000
H	-1.135630000	-4.928671000	-0.751921000
C	-3.189328000	-4.308838000	-0.435954000
C	-1.403070000	-4.366652000	1.326261000
H	-5.019664000	-3.321870000	0.174725000
C	-3.669761000	-3.436138000	1.867211000
H	-1.953550000	-3.409363000	3.191192000
H	-5.039617000	3.286616000	-0.402829000
C	-3.124438000	4.296258000	-0.314104000
C	-4.217588000	3.090002000	1.593737000
C	-1.894566000	4.005591000	1.855060000
H	-2.942519000	2.805550000	3.324009000
H	-1.065866000	4.874699000	0.048259000
H	-1.588012000	-0.145506000	-6.110651000
H	2.653816000	1.711239000	-2.415911000
H	0.979450000	2.305290000	-2.358160000
H	1.656070000	1.695043000	-3.884422000
H	2.165394000	-0.752125000	-4.084063000
H	1.741023000	-1.816836000	-2.722723000
H	3.085737000	-0.659596000	-2.579005000
H	-3.534457000	-5.309869000	-0.150314000
H	-3.398079000	-4.195147000	-1.507880000
H	-0.325142000	-4.294771000	1.523749000
H	-1.716570000	-5.368565000	1.643947000
H	-4.224961000	-2.694295000	2.455890000
H	-4.020588000	-4.424117000	2.189527000
H	-3.542899000	5.250470000	0.028121000
H	-3.028909000	4.364404000	-1.405737000
H	-4.909071000	2.286767000	1.879911000
H	-4.653683000	4.028084000	1.958175000
H	-0.913924000	3.859401000	2.326215000
H	-2.286974000	4.958175000	2.231101000
C	3.421673000	-0.081239000	0.190422000
C	3.905977000	-1.513493000	-0.051681000
C	4.290633000	0.961462000	-0.525424000
H	3.555273000	0.131435000	1.253359000
C	5.410929000	-1.612370000	0.247251000
H	3.737859000	-1.833532000	-1.086588000
H	3.357299000	-2.217652000	0.583760000
H	4.232809000	0.837731000	-1.622971000
C	6.153703000	-0.512971000	-0.495346000
H	5.794899000	-2.597873000	-0.041511000
H	5.584559000	-1.492966000	1.322957000
H	7.215998000	-0.497524000	-0.237167000
H	6.075337000	-0.665426000	-1.586896000
O	5.647829000	0.764680000	-0.147429000
C	3.967614000	2.394645000	-0.134894000
H	2.936791000	2.664000000	-0.383770000
H	4.100752000	2.516039000	0.943887000
H	4.645543000	3.087214000	-0.640196000

TS2$_{O5}$
Sum of electronic and zero-point Energies = -2124.221161
Sum of electronic and thermal Free Energies = -2124.294728
Esolv = -2125.4796285

C	1.938045000	2.054873000	0.554916000
C	1.518206000	3.372892000	0.324186000
H	1.193021000	3.682359000	-0.665194000
Au	1.021994000	0.520329000	-0.565742000
C	2.408541000	1.683408000	1.820489000
H	2.771758000	0.674618000	1.990311000
C	2.411824000	2.618335000	2.856404000
H	2.769107000	2.320757000	3.838298000
C	1.971155000	3.921771000	2.637161000
H	1.987994000	4.647355000	3.444362000
C	1.528139000	4.294915000	1.367895000
H	1.200783000	5.313693000	1.180906000
P	-1.119251000	-0.458533000	-0.149034000
C	-1.445264000	-1.645590000	-1.514278000
C	-1.024555000	-1.487742000	1.443359000
C	-2.499120000	0.850239000	-0.207518000
C	-2.660964000	-2.351362000	-1.578187000
C	-0.497704000	-1.864024000	-2.533681000
C	0.274358000	-2.325737000	1.336215000
C	-2.198902000	-2.463032000	1.677280000
C	-0.906525000	-0.537162000	2.655829000
C	-3.867707000	0.400370000	0.348152000
C	-2.032917000	2.095951000	0.584438000
C	-2.665470000	1.259639000	-1.691089000
H	-3.409760000	-2.199824000	-0.810906000
C	-2.936072000	-3.246986000	-2.604131000
C	-0.782881000	-2.773251000	-3.559257000
N	0.774209000	-1.166401000	-2.552847000
H	0.203138000	-3.010709000	0.480838000
H	1.138185000	-1.670245000	1.158556000
C	0.499246000	-3.131059000	2.628519000
C	-1.973154000	-3.257738000	2.979309000
H	-2.272919000	-3.169003000	0.843293000
H	-3.149955000	-1.923338000	1.740054000
C	-0.671341000	-1.348900000	3.942616000
H	-1.827881000	0.045819000	2.768875000
H	-0.085981000	0.175624000	2.503856000
H	-4.240557000	-0.476656000	-0.191316000
H	-3.777516000	0.124082000	1.404838000
C	-4.894503000	1.542028000	0.209492000
C	-3.065679000	3.229801000	0.455506000
H	-1.889612000	1.852054000	1.642074000
H	-1.062493000	2.435958000	0.206287000
C	-3.694783000	2.397241000	-1.817041000
H	-2.992673000	0.402075000	-2.289726000
H	-1.697227000	1.588636000	-2.096049000
H	-3.885294000	-3.773347000	-2.622386000
C	-1.988033000	-3.462527000	-3.600378000
H	-0.048529000	-2.944524000	-4.340706000
C	0.909726000	-0.338173000	-3.760131000
C	1.904058000	-2.100729000	-2.427394000
H	1.425670000	-3.708594000	2.520792000
C	-0.684221000	-4.080983000	2.856598000
C	0.622799000	-2.164100000	3.814355000
H	-2.829085000	-3.927929000	3.124553000
C	-1.859369000	-2.294376000	4.168392000
H	-0.588068000	-0.648866000	4.782866000

H	-5.855293000	1.190085000	0.604322000
C	-5.047989000	1.919837000	-1.270299000
C	-4.418134000	2.761721000	1.009191000
C	-3.218044000	3.619066000	-1.020756000
H	-2.705198000	4.090210000	1.032385000
H	-3.794490000	2.658165000	-2.877769000
H	-2.185075000	-4.161968000	-4.407138000
H	1.837110000	0.240237000	-3.701489000
H	0.068277000	0.354555000	-3.828197000
H	0.949350000	-0.935833000	-4.681643000
H	2.012667000	-2.747682000	-3.309634000
H	1.762279000	-2.726645000	-1.545026000
H	2.827762000	-1.532384000	-2.293238000
H	-0.524250000	-4.670301000	3.767848000
H	-0.765705000	-4.794508000	2.026247000
H	1.480941000	-1.494391000	3.670322000
H	0.808098000	-2.724151000	4.739163000
H	-2.786807000	-1.718025000	4.283102000
H	-1.721189000	-2.860022000	5.097991000
H	-5.798204000	2.712437000	-1.379694000
H	-5.409266000	1.059105000	-1.848230000
H	-4.326726000	2.507421000	2.073309000
H	-5.156233000	3.570040000	0.938512000
H	-2.261503000	3.983016000	-1.419254000
H	-3.937737000	4.440462000	-1.123854000
C	3.052908000	1.341800000	-1.001258000
H	3.235862000	2.379031000	-1.271376000
H	2.849589000	0.809652000	-1.947819000
C	4.255602000	0.703078000	-0.343520000
C	5.464264000	0.506178000	-1.261772000
H	4.561862000	1.303674000	0.526353000
C	5.087870000	-1.435622000	0.101266000
C	6.219073000	-0.602068000	-0.520948000
H	5.138857000	0.164426000	-2.251893000
H	6.052258000	1.418276000	-1.390687000
H	5.307187000	-1.729159000	1.134060000
H	4.873887000	-2.344250000	-0.472627000
H	6.861419000	-1.193072000	-1.178098000
H	6.853144000	-0.169611000	0.259153000
O	3.906489000	-0.615489000	0.074988000

$TS2_{O5Z}$

Sum of electronic and zero-point Energies = -2163.485240
Sum of electronic and thermal Free Energies = -2163.561322
Esolv = -2164.784024

Au	1.009066000	-0.285206000	0.174130000
P	-1.361090000	0.009476000	0.340938000
C	-1.846844000	-0.392022000	2.068055000
C	-1.858814000	1.820260000	0.027823000
C	-2.209647000	-1.277941000	-0.767606000
C	-3.185464000	-0.259299000	2.481805000
C	-0.897963000	-0.819033000	3.018533000
C	-1.407866000	2.628211000	1.268963000
C	-3.363929000	2.065977000	-0.214379000
C	-1.072878000	2.344770000	-1.198457000
C	-3.724062000	-1.466264000	-0.529088000
C	-1.971674000	-0.907100000	-2.248621000
C	-1.513352000	-2.630325000	-0.474349000
H	-3.934799000	0.071797000	1.773902000
C	-3.583004000	-0.541456000	3.782698000
C	-1.311306000	-1.104554000	4.325445000
N	0.504978000	-0.966628000	2.682885000
H	-1.940928000	2.284946000	2.162612000
H	-0.335046000	2.465753000	1.448773000
C	-1.677622000	4.128389000	1.054391000
C	-3.626421000	3.571468000	-0.418219000
H	-3.959722000	1.715950000	0.635033000
H	-3.701310000	1.518677000	-1.101954000
C	-1.346419000	3.844818000	-1.408200000
H	-1.351178000	1.793841000	-2.102735000
H	0.000607000	2.183271000	-1.050700000
H	-3.906686000	-1.767600000	0.507869000
H	-4.266544000	-0.530825000	-0.702556000
C	-4.274151000	-2.559853000	-1.466656000
C	-2.518671000	-2.015581000	-3.166238000
H	-2.480538000	0.033748000	-2.487713000
H	-0.900799000	-0.754989000	-2.436159000
C	-2.065672000	-3.730682000	-1.398007000
H	-1.671531000	-2.910777000	0.575387000
H	-0.428570000	-2.534756000	-0.627785000
H	-4.624407000	-0.426889000	4.066871000
C	-2.638765000	-0.970628000	4.711132000
H	-0.579276000	-1.436027000	5.055812000
C	0.951658000	-2.359409000	2.834231000
C	1.347790000	-0.057318000	3.478090000
H	-1.354146000	4.669990000	1.951654000
C	-3.180948000	4.346107000	0.829845000
C	-0.895246000	4.626960000	-0.167665000
H	-4.701892000	3.713878000	-0.579202000
C	-2.846451000	4.067913000	-1.642603000
H	-0.779164000	4.178189000	-2.285774000
H	-5.351150000	-2.657559000	-1.284347000
C	-3.573743000	-3.890512000	-1.162434000
C	-4.027639000	-2.168738000	-2.929986000
C	-1.808985000	-3.341729000	-2.860739000
H	-2.332567000	-1.725946000	-4.207563000
H	-1.550646000	-4.671106000	-1.165969000
H	-2.932244000	-1.198245000	5.731407000
H	1.984835000	-2.448371000	2.484980000
H	0.319061000	-3.015672000	2.233121000
H	0.923019000	-2.701878000	3.878202000
H	1.326096000	-0.294104000	4.551110000
H	1.005096000	0.970681000	3.341740000
H	2.386653000	-0.131745000	3.141985000
H	-3.391167000	5.415088000	0.703211000
H	-3.748774000	4.013059000	1.708454000
H	0.183521000	4.497443000	-0.006980000
H	-1.067497000	5.700432000	-0.313256000
H	-3.174558000	3.535349000	-2.544868000
H	-3.048691000	5.132635000	-1.811638000
H	-3.972662000	-4.683896000	-1.806205000
H	-3.767372000	-4.194661000	-0.125559000
H	-4.546105000	-1.230486000	-3.167149000
H	-4.439006000	-2.935266000	-3.598038000
H	-0.730605000	-3.247111000	-3.046230000
H	-2.177512000	-4.129098000	-3.529479000
C	4.340581000	0.182170000	0.429498000
H	3.915717000	1.183940000	0.592444000
C	3.193524000	-0.813419000	0.216306000
C	5.468313000	0.258565000	-0.599909000
H	5.660590000	-0.726796000	-1.036369000
H	5.251341000	0.951134000	-1.414354000
C	6.390559000	-0.087434000	1.560678000
H	6.740178000	0.433035000	2.458036000
H	6.857312000	-1.081282000	1.536790000
C	6.650562000	0.687675000	0.271504000
H	6.616954000	1.766790000	0.457602000
H	7.621249000	0.455099000	-0.173391000
C	3.568472000	-2.177608000	-0.324511000
H	4.343060000	-2.594779000	0.330104000

H	2.721103000	-2.869599000	-0.307968000
H	3.963983000	-2.144033000	-1.341022000
H	2.836326000	-0.970272000	1.248944000
C	2.255790000	0.133237000	-1.466276000
C	2.569299000	1.485528000	-1.664069000
C	2.195845000	-0.724783000	-2.571085000
C	2.807787000	1.967056000	-2.950967000
H	2.624299000	2.171984000	-0.824862000
C	2.436134000	-0.233271000	-3.854057000
H	1.953810000	-1.774817000	-2.442540000
C	2.746813000	1.110989000	-4.049311000
H	3.046639000	3.017853000	-3.089148000
H	2.381548000	-0.910533000	-4.701701000
H	2.943543000	1.487704000	-5.048205000
O	4.966543000	-0.244298000	1.647684000

TS2$_{O5E}$
Sum of electronic and zero-point Energies = -2163.479977
Sum of electronic and thermal Free Energies = -2163.554924
Esolv = -2164.77776907

Au	1.029956000	-0.497944000	0.255766000
P	-1.168273000	0.361201000	0.209451000
C	-1.871842000	0.850671000	1.842911000
C	-1.216723000	1.929667000	-0.871710000
C	-2.252957000	-1.085352000	-0.431837000
C	-3.223721000	1.244829000	1.896905000
C	-1.139336000	0.843156000	3.048422000
C	-0.149670000	2.892632000	-0.297265000
C	-2.564953000	2.685603000	-0.893827000
C	-0.823525000	1.560027000	-2.319183000
C	-3.637507000	-0.686636000	-0.989816000
C	-1.489669000	-1.845671000	-1.545822000
C	-2.447717000	-2.052860000	0.760947000
H	-3.816604000	1.253273000	0.992524000
C	-3.836920000	1.629994000	3.081896000
C	-1.770796000	1.242535000	4.234880000
N	0.237775000	0.424094000	3.104757000
H	-0.412928000	3.169345000	0.731924000
H	0.824030000	2.387268000	-0.258852000
C	-0.042667000	4.158416000	-1.167811000
C	-2.455835000	3.943036000	-1.778829000
H	-2.836585000	2.996375000	0.119760000
H	-3.368653000	2.044968000	-1.271411000
C	-0.712105000	2.829281000	-3.183334000
H	-1.576154000	0.895888000	-2.759078000
H	0.130802000	1.015456000	-2.322083000
H	-4.227724000	-0.154728000	-0.237862000
H	-3.523440000	-0.020840000	-1.852837000
C	-4.420131000	-1.942834000	-1.422263000
C	-2.277405000	-3.092576000	-1.987571000
H	-1.318527000	-1.195792000	-2.410808000
H	-0.503130000	-2.153790000	-1.179477000
C	-3.231006000	-3.300704000	0.314466000
H	-2.988765000	-1.553210000	1.571976000
H	-1.467780000	-2.351641000	1.159996000
H	-4.881590000	1.925205000	3.077650000
C	-3.101696000	1.634598000	4.263332000
H	-1.200001000	1.239774000	5.158863000
C	0.394807000	-0.762583000	3.949248000
C	1.115882000	1.513991000	3.536012000
H	0.723101000	4.814241000	-0.735099000
C	-1.394535000	4.883776000	-1.193997000
C	0.353166000	3.766143000	-2.598568000
H	-3.432155000	4.442571000	-1.783026000
C	-2.069261000	3.545364000	-3.209074000
H	-0.428573000	2.532476000	-4.200585000
H	-5.399153000	-1.622447000	-1.798902000
C	-4.606982000	-2.873778000	-0.216209000
C	-3.650048000	-2.673786000	-2.528466000
C	-2.457120000	-4.033561000	-0.789118000
H	-1.704618000	-3.597945000	-2.774778000
H	-3.356314000	-3.958908000	1.182951000
H	-3.561123000	1.936754000	5.199723000
H	1.428961000	-1.113922000	3.896132000
H	-0.258902000	-1.559725000	3.586894000
H	0.161519000	-0.580299000	5.010467000
H	0.927731000	1.845471000	4.569802000
H	0.987518000	2.373496000	2.873511000
H	2.157906000	1.188531000	3.477564000
H	-1.320852000	5.795543000	-1.799306000
H	-1.679883000	5.196036000	-0.181132000
H	1.333591000	3.272890000	-2.604664000
H	0.447387000	4.663968000	-3.221647000
H	-2.836404000	2.891387000	-3.644085000
H	-2.013900000	4.436243000	-3.846517000
H	-5.187776000	-3.756648000	-0.510053000
H	-5.177976000	-2.366306000	0.572270000
H	-3.532928000	-2.023129000	-3.405141000
H	-4.211657000	-3.555616000	-2.860456000
H	-1.478475000	-4.361416000	-0.414763000
H	-3.000761000	-4.935924000	-1.095214000
C	3.617661000	-0.493458000	0.511780000
C	2.455337000	-2.037566000	-0.364433000
C	2.613366000	-2.183372000	-1.750413000
C	2.296033000	-3.166679000	0.449618000
C	2.540882000	-3.451219000	-2.320397000
H	2.786768000	-1.319482000	-2.383290000
C	2.215033000	-4.431783000	-0.134015000
H	2.220205000	-3.066265000	1.527673000
C	2.339121000	-4.574331000	-1.514857000
H	2.654565000	-3.564505000	-3.394601000
H	2.071589000	-5.305307000	0.495251000
H	2.295914000	-5.561967000	-1.963942000
C	3.812615000	-0.855063000	1.961748000
H	2.938287000	-0.623893000	2.572369000
H	4.069177000	-1.907744000	2.089478000
H	4.651593000	-0.257253000	2.333773000
H	4.412043000	-0.918656000	-0.101636000
C	3.560240000	1.016319000	0.217829000
C	3.517059000	1.410611000	-1.261709000
H	2.772652000	1.498558000	0.807772000
C	5.646701000	1.940195000	-0.384909000
C	4.995710000	1.410777000	-1.657392000
H	3.109810000	2.422958000	-1.346764000
H	2.892026000	0.752650000	-1.869164000
H	5.678063000	3.037682000	-0.377924000
H	6.660691000	1.565733000	-0.218600000
H	5.203906000	2.036729000	-2.528425000
H	5.345767000	0.398119000	-1.885164000
O	4.816753000	1.497731000	0.700672000

TS2'$_{O5E}$
Sum of electronic and zero-point Energies = -2163.483415
Sum of electronic and thermal Free Energies = -2163.558652
Esolv = -2164.7860373

C	2.104308000	-1.508698000	-1.023642000
C	1.933811000	-2.897842000	-0.974731000
H	1.718512000	-3.397197000	-0.036492000

Au	1.026221000	-0.330872000	0.352271000
C	2.371143000	-0.897915000	-2.258024000
H	2.484969000	0.177400000	-2.328368000
C	2.466506000	-1.668055000	-3.416126000
H	2.671518000	-1.178292000	-4.363973000
C	2.303084000	-3.051098000	-3.362095000
H	2.390492000	-3.648663000	-4.264103000
C	2.028657000	-3.659375000	-2.139558000
H	1.891344000	-4.735432000	-2.082154000
P	-1.269814000	0.344134000	0.240325000
C	-1.678044000	1.230682000	1.799006000
C	-1.482709000	1.607630000	-1.161782000
C	-2.405567000	-1.181997000	0.155352000
C	-2.989751000	1.678514000	2.043083000
C	-0.703978000	1.463353000	2.790232000
C	-0.334784000	2.633774000	-0.994496000
C	-2.816765000	2.385806000	-1.157424000
C	-1.317721000	0.893989000	-2.522270000
C	-3.874827000	-0.904064000	-0.229610000
C	-1.813587000	-2.184856000	-0.864903000
C	-2.365191000	-1.840507000	1.555782000
H	-3.760017000	1.510170000	1.301275000
C	-3.334746000	2.336667000	3.216956000
C	-1.062006000	2.131750000	3.967282000
N	0.668272000	1.025367000	2.626732000
H	-0.436426000	3.151427000	-0.031444000
H	0.634123000	2.113874000	-0.986440000
C	-0.357788000	3.658469000	-2.142548000
C	-2.839131000	3.401590000	-2.317550000
H	-2.933058000	2.927723000	-0.212906000
H	-3.667497000	1.702684000	-1.254097000
C	-1.332545000	1.925371000	-3.665476000
H	-2.137526000	0.183176000	-2.676592000
H	-0.381921000	0.320620000	-2.542049000
H	-4.338945000	-0.203765000	0.472510000
H	-3.928372000	-0.455126000	-1.227985000
C	-4.679741000	-2.219356000	-0.221149000
C	-2.625799000	-3.492454000	-0.863594000
H	-1.813737000	-1.757605000	-1.872870000
H	-0.768587000	-2.399506000	-0.615177000
C	-3.174297000	-3.149832000	1.552236000
H	-2.774623000	-1.159328000	2.310167000
H	-1.322871000	-2.049480000	1.838384000
H	-4.357296000	2.666832000	3.371150000
C	-2.362429000	2.567669000	4.185888000
H	-0.308627000	2.313014000	4.728151000
C	1.059278000	0.069276000	3.673420000
C	1.593120000	2.165837000	2.587864000
H	0.467026000	4.366571000	-1.994584000
C	-1.696482000	4.408916000	-2.134439000
C	-0.183363000	2.925597000	-3.480083000
H	-3.802307000	3.925497000	-2.295860000
C	-2.674311000	2.670881000	-3.656911000
H	-1.210105000	1.390728000	-4.615230000
H	-5.717728000	-1.988216000	-0.489446000
C	-4.632221000	-2.843367000	1.180475000
C	-4.082401000	-3.194809000	-1.243603000
C	-2.575060000	-4.127030000	0.532413000
H	-2.180908000	-4.173697000	-1.599044000
H	-3.131546000	-3.586013000	2.557722000
H	-2.613791000	3.082920000	5.107957000
H	2.061475000	-0.312093000	3.456886000
H	0.359264000	-0.768808000	3.684184000
H	1.081081000	0.520387000	4.675625000
H	1.631003000	2.713951000	3.540191000
H	1.292392000	2.857394000	1.798310000
H	2.604043000	1.805636000	2.372003000
H	-1.715402000	5.152814000	-2.940223000
H	-1.819821000	4.957788000	-1.191756000
H	0.782713000	2.403662000	-3.505316000
H	-0.174944000	3.647050000	-4.306163000
H	-3.501328000	1.965576000	-3.811454000
H	-2.712674000	3.389761000	-4.484448000
H	-5.227078000	-3.764568000	1.201621000
H	-5.077199000	-2.161563000	1.917020000
H	-4.133064000	-2.766641000	-2.253286000
H	-4.665663000	-4.123520000	-1.263398000
H	-1.538775000	-4.370522000	0.802673000
H	-3.134775000	-5.070323000	0.540283000
C	3.218040000	-0.850495000	0.672722000
C	4.471376000	-0.366600000	-0.078951000
H	4.750952000	-1.103765000	-0.839453000
C	5.694281000	1.721704000	-0.168914000
C	5.932932000	0.994550000	1.144190000
H	6.512630000	1.513669000	-0.865140000
H	5.609480000	2.805833000	-0.056977000
H	5.341114000	1.439565000	1.961307000
H	6.978708000	0.966172000	1.460700000
C	4.397605000	1.063304000	-0.655012000
H	3.522071000	1.592803000	-0.257118000
H	4.321855000	1.068189000	-1.743177000
O	5.524615000	-0.346905000	0.892756000
C	3.440694000	-2.140102000	1.441963000
H	2.536218000	-2.487814000	1.950265000
H	3.809378000	-2.940339000	0.796457000
H	4.206238000	-1.951946000	2.200083000
H	3.011376000	-0.045420000	1.402511000

$TS2_{O6}$

Sum of electronic and zero-point Energies = -2124.212115
Sum of electronic and thermal Free Energies = -2124.285254
Esolv = -2125.4790443

C	-2.334280000	0.408734000	-1.498718000
C	-2.398961000	1.729808000	-1.963599000
H	-2.318805000	2.565339000	-1.275342000
Au	-1.165608000	0.049029000	0.212341000
C	-2.460360000	-0.647774000	-2.410096000
H	-2.430010000	-1.677818000	-2.068873000
C	-2.620242000	-0.382645000	-3.769721000
H	-2.708717000	-1.211531000	-4.466322000
C	-2.675667000	0.931064000	-4.231863000
H	-2.814093000	1.133349000	-5.289320000
C	-2.564341000	1.983151000	-3.324627000
H	-2.612583000	3.011574000	-3.671091000
P	1.222686000	-0.087987000	0.337937000
C	1.661272000	-0.416848000	2.092300000
C	1.800215000	-1.602122000	-0.652717000
C	2.029218000	1.568637000	-0.136828000
C	3.008541000	-0.512876000	2.487344000
C	0.668463000	-0.562598000	3.081566000
C	0.873625000	-2.770931000	-0.234258000
C	3.259136000	-2.044312000	-0.405123000
C	1.602738000	-1.326435000	-2.160352000
C	3.550378000	1.518708000	-0.396489000
C	1.330393000	2.113726000	-1.405872000
C	1.747275000	2.551079000	1.025839000
H	3.792683000	-0.395908000	1.750038000
C	3.370881000	-0.755113000	3.806531000
C	1.045072000	-0.812399000	4.406575000
N	-0.743353000	-0.448276000	2.767498000

H	0.998658000	-2.981234000	0.836218000
H	-0.177740000	-2.490294000	-0.392759000
C	1.195639000	-4.031957000	-1.055959000
C	3.579439000	-3.299820000	-1.241138000
H	3.405555000	-2.281056000	0.653978000
H	3.959818000	-1.243794000	-0.665827000
C	1.918174000	-2.593975000	-2.975341000
H	2.270484000	-0.520785000	-2.486403000
H	0.574042000	-0.997829000	-2.356784000
H	4.085506000	1.146523000	0.483526000
H	3.772967000	0.841899000	-1.229249000
C	4.073093000	2.929001000	-0.735933000
C	1.863337000	3.516065000	-1.749920000
H	1.491335000	1.443656000	-2.256490000
H	0.247585000	2.159658000	-1.243264000
C	2.275939000	3.954156000	0.677256000
H	2.224177000	2.198736000	1.947345000
H	0.665266000	2.599134000	1.217766000
H	4.420429000	-0.822213000	4.075455000
C	2.381201000	-0.911037000	4.772906000
H	0.278497000	-0.927844000	5.167030000
C	-1.358433000	0.682919000	3.480932000
C	-1.463266000	-1.699182000	3.048991000
H	0.521902000	-4.836854000	-0.736932000
C	2.653344000	-4.445774000	-0.813136000
C	0.983825000	-3.733758000	-2.546920000
H	4.623835000	-3.577917000	-1.055218000
C	3.377944000	-3.001668000	-2.732902000
H	1.766133000	-2.368535000	-4.037821000
H	5.154229000	2.862558000	-0.907656000
C	3.790586000	3.879203000	0.436076000
C	3.375630000	3.445003000	-2.001309000
C	1.576701000	4.473539000	-0.585808000
H	1.352072000	3.865958000	-2.655027000
H	2.067162000	4.622325000	1.521615000
H	2.646419000	-1.104446000	5.807845000
H	-2.396534000	0.799953000	3.154740000
H	-0.812594000	1.600542000	3.252021000
H	-1.365210000	0.541077000	4.570621000
H	-1.483586000	-1.944774000	4.120111000
H	-0.992293000	-2.523578000	2.509856000
H	-2.498934000	-1.602677000	2.709334000
H	2.886293000	-5.353324000	-1.383297000
H	2.809780000	-4.686743000	0.246381000
H	-0.062291000	-3.457890000	-2.735492000
H	1.187225000	-4.631956000	-3.142611000
H	4.054778000	-2.200289000	-3.056996000
H	3.627317000	-3.887276000	-3.330044000
H	4.185406000	4.877884000	0.213281000
H	4.303155000	3.530454000	1.342141000
H	3.589981000	2.783805000	-2.851149000
H	3.761729000	4.436876000	-2.266125000
H	0.494574000	4.553746000	-0.415707000
H	1.934956000	5.481679000	-0.827402000
C	-4.307785000	1.308035000	0.240980000
H	-4.750599000	1.433912000	-0.757967000
H	-3.759400000	2.218046000	0.497677000
C	-3.412754000	0.069601000	0.288138000
C	-4.210503000	-1.189934000	-0.022367000
H	-3.111622000	0.006400000	1.350361000
C	-6.176705000	0.065554000	0.923094000
C	-5.414453000	-1.254608000	0.935721000
H	-4.573508000	-1.156014000	-1.055468000
H	-3.598939000	-2.093892000	0.077666000
H	-6.961126000	0.081329000	1.683693000
H	-6.651115000	0.222545000	-0.059612000
H	-6.078560000	-2.076790000	0.645320000
H	-5.074694000	-1.460528000	1.958425000
O	-5.317578000	1.160746000	1.218474000

TS2$_{O6Z}$

Sum of electronic and zero-point Energies = -2163.481154
Sum of electronic and thermal Free Energies = -2163.553645
Esolv = -2164.7880798

C	-2.233814000	1.076538000	-1.026109000
C	-2.294181000	2.424573000	-0.639332000
H	-2.197936000	2.706729000	0.405023000
Au	-1.071969000	-0.109754000	0.237765000
C	-2.401569000	0.734700000	-2.371151000
H	-2.365322000	-0.302733000	-2.687831000
C	-2.605809000	1.734464000	-3.322923000
H	-2.734196000	1.458202000	-4.365735000
C	-2.647606000	3.074941000	-2.944002000
H	-2.812685000	3.848215000	-3.687749000
C	-2.486765000	3.415757000	-1.600718000
H	-2.525108000	4.456834000	-1.292528000
P	1.325668000	-0.210479000	0.255814000
C	1.827539000	-1.149556000	1.752096000
C	1.953954000	-1.189159000	-1.250412000
C	2.035173000	1.539606000	0.454923000
C	3.184525000	-1.411111000	2.017130000
C	0.867365000	-1.640359000	2.658398000
C	1.630816000	-2.679850000	-0.986869000
C	3.461847000	-1.047084000	-1.550510000
C	1.157250000	-0.741500000	-2.500018000
C	3.539745000	1.618990000	0.794617000
C	1.762908000	2.341143000	-0.838112000
C	1.252576000	2.191798000	1.621984000
H	3.942720000	-1.059238000	1.328714000
C	3.588091000	-2.115990000	3.144293000
C	1.285824000	-2.340917000	3.795904000
N	-0.554853000	-1.453764000	2.435949000
H	2.173705000	-3.038331000	-0.105251000
H	0.557374000	-2.795722000	-0.776151000
C	2.015603000	-3.532782000	-2.209192000
C	3.841054000	-1.911124000	-2.769444000
H	4.062301000	-1.363236000	-0.690638000
H	3.711134000	-0.000858000	-1.760046000
C	1.548319000	-1.595216000	-3.719397000
H	1.336864000	0.316251000	-2.718541000
H	0.082529000	-0.844350000	-2.306791000
H	3.741713000	1.089550000	1.731645000
H	4.144018000	1.149591000	0.011144000
C	3.966563000	3.091740000	0.959166000
C	2.187885000	3.808755000	-0.648717000
H	2.329585000	1.912895000	-1.673102000
H	0.699188000	2.289057000	-1.102529000
C	1.681452000	3.659525000	1.800138000
H	1.435669000	1.634987000	2.550514000
H	0.173385000	2.148082000	1.420297000
H	4.643636000	-2.300864000	3.317570000
C	2.631719000	-2.578074000	4.044240000
H	0.545298000	-2.712344000	4.497965000
C	-1.165621000	-0.641951000	3.501378000
C	-1.244783000	-2.745931000	2.302057000
H	1.780659000	-4.581044000	-1.987723000
C	3.520123000	-3.383468000	-2.477843000
C	1.223102000	-3.068062000	-3.438051000
H	4.916027000	-1.794625000	-2.952691000
C	3.049930000	-1.440832000	-3.997113000

H	0.972794000	-1.245010000	-4.585047000
H	5.039884000	3.113875000	1.183353000
C	3.181616000	3.722379000	2.116823000
C	3.689402000	3.865869000	-0.336462000
C	1.393302000	4.433791000	0.506157000
H	1.979686000	4.351361000	-1.578768000
H	1.107257000	4.091325000	2.629215000
H	2.930110000	-3.125649000	4.933045000
H	-2.218699000	-0.463428000	3.263987000
H	-0.652611000	0.319480000	3.569256000
H	-1.122055000	-1.134100000	4.483045000
H	-1.233013000	-3.331653000	3.231750000
H	-0.771880000	-3.334105000	1.512844000
H	-2.289210000	-2.567221000	2.032540000
H	3.813340000	-4.008057000	-3.330370000
H	4.097555000	-3.735713000	-1.613121000
H	0.145550000	-3.194269000	-3.266197000
H	1.479581000	-3.685504000	-4.307555000
H	3.289379000	-0.394252000	-4.226523000
H	3.333596000	-2.031088000	-4.876947000
H	3.492146000	4.764434000	2.260809000
H	3.395910000	3.194002000	3.054938000
H	4.266751000	3.439241000	-1.167207000
H	4.013831000	4.908016000	-0.227901000
H	0.319229000	4.412488000	0.280318000
H	1.672429000	5.487429000	0.628805000
C	-3.318437000	-0.308733000	0.249531000
H	-3.060030000	-0.629201000	1.273374000
C	-4.541060000	0.603005000	0.508329000
C	-3.711816000	-1.541242000	-0.553980000
H	-4.078763000	-1.251067000	-1.543685000
H	-2.861845000	-2.214123000	-0.716829000
C	-5.925053000	-1.340823000	0.666687000
H	-6.581999000	-1.825231000	1.393773000
H	-6.546941000	-1.040140000	-0.187533000
C	-4.818146000	-2.290330000	0.217817000
H	-4.381648000	-2.768165000	1.103544000
H	-5.235391000	-3.091334000	-0.403478000
O	-5.398724000	-0.197197000	1.331501000
C	-5.301702000	1.176726000	-0.684371000
H	-6.269069000	1.535045000	-0.320775000
H	-4.774020000	2.019702000	-1.128983000
H	-5.487193000	0.448476000	-1.476706000
H	-4.238819000	1.427826000	1.161496000

TS2$_{O6E}$

Sum of electronic and zero-point Energies = -2163.488707

Sum of electronic and thermal Free Energies = -2163.561985

Esolv = -2164.79351

C	2.280686000	1.214322000	-1.068846000
C	2.395320000	0.852327000	-2.416214000
H	2.355799000	-0.186247000	-2.722167000
Au	1.098020000	0.038908000	0.223143000
C	2.336235000	2.572475000	-0.719709000
H	2.267817000	2.879126000	0.319285000
C	2.479597000	3.546673000	-1.705362000
H	2.511908000	4.593531000	-1.417045000
C	2.592523000	3.182970000	-3.046462000
H	2.720024000	3.942703000	-3.811226000
C	2.549561000	1.835514000	-3.394808000
H	2.638131000	1.536482000	-4.435395000
P	-1.286184000	-0.203772000	0.276976000
C	-1.737871000	-0.970137000	1.887249000
C	-2.125181000	1.505679000	0.215892000
C	-1.829693000	-1.436762000	-1.061662000
C	-3.089571000	-1.157647000	2.231602000
C	-0.756627000	-1.375601000	2.814171000
C	-1.847453000	2.180258000	1.581411000
C	-3.647544000	1.498899000	-0.043220000
C	-1.448028000	2.354055000	-0.888240000
C	-3.290269000	-1.929371000	-0.962515000
C	-1.602180000	-0.807840000	-2.454294000
C	-0.904678000	-2.669472000	-0.911473000
H	-3.865513000	-0.855460000	1.539961000
C	-3.467904000	-1.725257000	3.441948000
C	-1.149837000	-1.949517000	4.029247000
N	0.658827000	-1.208571000	2.548870000
H	-2.311270000	1.606419000	2.391712000
H	-0.764618000	2.197438000	1.774128000
C	-2.398669000	3.617246000	1.587681000
C	-4.192823000	2.941119000	-0.024725000
H	-4.174263000	0.915538000	0.718766000
H	-3.864699000	1.044608000	-1.016571000
C	-2.001507000	3.790475000	-0.875479000
H	-1.617210000	1.910199000	-1.874587000
H	-0.364128000	2.377281000	-0.733711000
H	-3.459162000	-2.411960000	0.005869000
H	-3.992395000	-1.092806000	-1.042762000
C	-3.579344000	-2.950312000	-2.081883000
C	-1.885425000	-1.844443000	-3.556388000
H	-2.270552000	0.049371000	-2.594247000
H	-0.573222000	-0.434161000	-2.539939000
C	-1.194085000	-3.697542000	-2.019603000
H	-1.057845000	-3.130859000	0.073008000
H	0.147466000	-2.357835000	-0.965516000
H	-4.520761000	-1.853319000	3.673224000
C	-2.490053000	-2.126771000	4.347400000
H	-0.391669000	-2.262841000	4.740799000
C	1.360655000	-2.499215000	2.519354000
C	1.281465000	-0.297406000	3.521075000
H	-2.192256000	4.063102000	2.568413000
C	-3.914029000	3.579832000	1.342898000
C	-1.716882000	4.438725000	0.485690000
H	-5.274440000	2.900891000	-0.201772000
C	-3.514498000	3.762623000	-1.128509000
H	-1.500906000	4.357385000	-1.669721000
H	-4.625220000	-3.268119000	-1.993388000
C	-2.653073000	-4.162509000	-1.919110000
C	-3.346741000	-2.303285000	-3.453861000
C	-0.950574000	-3.050158000	-3.390204000
H	-1.711894000	-1.370806000	-4.530308000
H	-0.520093000	-4.552604000	-1.885932000
H	-2.767424000	-2.574577000	5.296828000
H	2.403193000	-2.339410000	2.227405000
H	0.889576000	-3.157766000	1.787139000
H	1.362736000	-3.003862000	3.496039000
H	1.277699000	-0.700329000	4.544050000
H	0.751417000	0.657560000	3.521391000
H	2.323135000	-0.122977000	3.234685000
H	-4.324911000	4.596301000	1.373275000
H	-4.413772000	3.011805000	2.138483000
H	-0.634379000	4.489122000	0.663478000
H	-2.089564000	5.470312000	0.499438000
H	-3.728324000	3.327172000	-2.113413000
H	-3.916003000	4.783335000	-1.139091000
H	-2.865132000	-4.907271000	-2.695879000
H	-2.831365000	-4.651385000	-0.952518000
H	-4.023246000	-1.449959000	-3.593611000
H	-3.574041000	-3.021301000	-4.251224000
H	0.097516000	-2.734652000	-3.481799000

H	-1.131556000	-3.780074000	-4.188550000
C	3.368171000	-0.022331000	0.288259000
H	3.046387000	-0.642660000	1.145959000
C	4.119992000	-0.994052000	-0.634970000
H	4.520130000	-0.437981000	-1.497812000
C	4.283148000	1.059577000	0.855251000
H	4.627559000	1.720778000	0.053577000
H	3.749478000	1.683404000	1.580062000
C	3.318336000	-2.197852000	-1.097312000
H	2.980674000	-2.777475000	-0.233000000
H	2.442408000	-1.916014000	-1.685893000
H	3.952797000	-2.845622000	-1.707088000
C	6.145511000	-0.569747000	0.543523000
H	6.961613000	-1.129985000	1.006860000
H	6.552888000	-0.029917000	-0.327084000
C	5.503719000	0.404427000	1.518601000
H	5.208298000	-0.138516000	2.424957000
H	6.220181000	1.176863000	1.820531000
O	5.199767000	-1.538621000	0.114810000

TS2’$_{O6E}$

Sum of electronic and zero-point Energies = -2163.481167
Sum of electronic and thermal Free Energies = -2163.553890
Esolv = -2164.786478

C	2.180979000	1.324699000	1.283461000
C	2.322907000	2.642789000	0.829503000
H	2.436430000	2.858313000	-0.226146000
Au	1.167638000	0.009342000	-0.025371000
C	2.086628000	1.067116000	2.655502000
H	2.003432000	0.051541000	3.027827000
C	2.091470000	2.126791000	3.561449000
H	2.009429000	1.916380000	4.624022000
C	2.208125000	3.441689000	3.113495000
H	2.223340000	4.262095000	3.823979000
C	2.322019000	3.692924000	1.747559000
H	2.425127000	4.711984000	1.385679000
P	-1.194103000	-0.270746000	-0.316235000
C	-1.651932000	-1.253794000	-1.798547000
C	-1.884432000	-1.224790000	1.188436000
C	-1.924807000	1.466839000	-0.579868000
C	-3.008968000	-1.530780000	-2.054013000
C	-0.689556000	-1.776191000	-2.683224000
C	-1.579960000	-2.725009000	0.959322000
C	-3.398112000	-1.051513000	1.444205000
C	-1.119441000	-0.774507000	2.455883000
C	-3.410422000	1.509777000	-1.000962000
C	-1.738740000	2.297734000	0.709933000
C	-1.095220000	2.115578000	-1.716174000
H	-3.769458000	-1.151738000	-1.383785000
C	-3.412036000	-2.285028000	-3.148079000
C	-1.110647000	-2.530801000	-3.786668000
N	0.726535000	-1.561105000	-2.482889000
H	-2.102899000	-3.089725000	0.068815000
H	-0.503530000	-2.863460000	0.781409000
C	-2.016196000	-3.550716000	2.183579000
C	-3.828773000	-1.889821000	2.664063000
H	-3.981377000	-1.371699000	0.574865000
H	-3.635800000	0.001717000	1.630495000
C	-1.562809000	-1.598278000	3.677856000
H	-1.274817000	0.292350000	2.649072000
H	-0.042998000	-0.913661000	2.295887000
H	-3.551571000	0.960744000	-1.937584000
H	-4.046820000	1.042036000	-0.242311000
C	-3.858276000	2.969653000	-1.215381000
C	-2.184152000	3.752212000	0.470653000
H	-2.336330000	1.871524000	1.524255000
H	-0.689221000	2.277385000	1.029086000
C	-1.544566000	3.570127000	-1.944901000
H	-1.215524000	1.537407000	-2.641862000
H	-0.027448000	2.100788000	-1.456868000
H	-4.467538000	-2.478308000	-3.312221000
C	-2.454105000	-2.786175000	-4.025086000
H	-0.368238000	-2.928858000	-4.471910000
C	1.296557000	-0.789198000	-3.594940000
C	1.428142000	-2.837808000	-2.301259000
H	-1.792909000	-4.606177000	1.985772000
C	-3.525151000	-3.371770000	2.404399000
C	-1.254563000	-3.080702000	3.429709000
H	-4.906732000	-1.751916000	2.811508000
C	-3.068479000	-1.413238000	3.908130000
H	-1.006826000	-1.243207000	4.554445000
H	-4.918326000	2.963547000	-1.496537000
C	-3.026344000	3.595926000	-2.341952000
C	-3.667448000	3.772267000	0.078189000
C	-1.341964000	4.373386000	-0.652301000
H	-2.036425000	4.315511000	1.399993000
H	-0.935451000	3.999217000	-2.750178000
H	-2.750033000	-3.375321000	-4.887846000
H	2.354140000	-0.597955000	-3.403579000
H	0.779847000	0.169553000	-3.677174000
H	1.218814000	-1.309041000	-4.561688000
H	1.396786000	-3.480278000	-3.194015000
H	0.981687000	-3.385189000	-1.467813000
H	2.478324000	-2.644944000	-2.068084000
H	-3.854905000	-3.977993000	3.256858000
H	-4.082528000	-3.727185000	1.527952000
H	-0.174520000	-3.227792000	3.293881000
H	-1.548900000	-3.679830000	4.300039000
H	-3.296396000	-0.359071000	4.113397000
H	-3.388584000	-1.984249000	4.788132000
H	-3.349632000	4.628450000	-2.522098000
H	-3.179860000	3.046058000	-3.279602000
H	-4.280177000	3.348303000	0.884637000
H	-4.006361000	4.805325000	-0.067013000
H	-0.281408000	4.378816000	-0.369265000
H	-1.636014000	5.418368000	-0.809546000
C	3.461926000	-0.058504000	0.360400000
C	3.558880000	-1.433438000	1.018019000
C	3.995967000	-0.086983000	-1.085306000
H	4.122315000	0.627539000	0.888397000
C	5.022037000	-1.907223000	0.942253000
H	2.918252000	-2.161901000	0.503677000
H	3.236335000	-1.400594000	2.062701000
H	3.444553000	-0.844879000	-1.662654000
C	5.528855000	-1.802085000	-0.488342000
H	5.104401000	-2.938216000	1.304610000
H	5.644619000	-1.280045000	1.590863000
H	6.599741000	-2.010843000	-0.551918000
H	5.004888000	-2.525510000	-1.136340000
O	5.356077000	-0.487091000	-0.996429000
C	3.987204000	1.235632000	-1.826274000
H	2.965496000	1.576466000	-2.018471000
H	4.520903000	2.001466000	-1.257163000
H	4.496839000	1.113612000	-2.785411000

6_{O5}

Sum of electronic and zero-point Energies = -2124.294544
Sum of electronic and thermal Free Energies = -2124.372885
Esolv = -2125.5449823

C	3.542948000	1.817822000	0.055580000
C	3.552257000	3.074993000	-0.543284000
H	4.248112000	3.269763000	-1.355614000
Au	0.456699000	1.122257000	0.346762000
C	2.657284000	1.604679000	1.145074000
H	2.766578000	0.692035000	1.726245000
C	1.805139000	2.645427000	1.587180000
H	1.257871000	2.531041000	2.520323000
C	1.829844000	3.901138000	0.944283000
H	1.196047000	4.704735000	1.305759000
C	2.706641000	4.106518000	-0.108864000
H	2.759367000	5.078844000	-0.590006000
P	-1.283543000	-0.283237000	-0.287753000
C	-2.149961000	0.330576000	-1.789700000
C	-0.521818000	-1.962164000	-0.746727000
C	-2.558176000	-0.313057000	1.130202000
C	-3.271604000	-0.374802000	-2.265621000
C	-1.749017000	1.492763000	-2.484498000
C	0.600356000	-1.663169000	-1.771889000
C	-1.489058000	-2.981167000	-1.388937000
C	0.111815000	-2.589986000	0.514824000
C	-3.549782000	-1.497354000	1.105915000
C	-1.805143000	-0.336540000	2.484245000
C	-3.351656000	1.013490000	1.046129000
H	-3.607110000	-1.263908000	-1.746784000
C	-3.974180000	0.034948000	-3.391970000
C	-2.464127000	1.887788000	-3.622651000
N	-0.631332000	2.289108000	-2.048468000
H	0.168698000	-1.215063000	-2.676409000
H	1.305570000	-0.933846000	-1.349238000
C	1.352737000	-2.955867000	-2.134057000
C	-0.731571000	-4.278432000	-1.738294000
H	-1.916541000	-2.566593000	-2.307838000
H	-2.318605000	-3.214766000	-0.713006000
C	0.869241000	-3.877075000	0.142871000
H	-0.666149000	-2.834192000	1.247606000
H	0.797093000	-1.874366000	0.988406000
H	-4.114052000	-1.516410000	0.167787000
H	-3.010122000	-2.447844000	1.185988000
C	-4.543159000	-1.378573000	2.278763000
C	-2.798732000	-0.232128000	3.654710000
H	-1.214114000	-1.253047000	2.584067000
H	-1.097741000	0.502668000	2.524993000
C	-4.340354000	1.117874000	2.221288000
H	-3.900959000	1.069154000	0.099870000
H	-2.655335000	1.864207000	1.066689000
H	-4.835249000	-0.534186000	-3.728298000
C	-3.563986000	1.173542000	-4.079692000
H	-2.149358000	2.779348000	-4.157411000
C	-1.030946000	3.650435000	-1.687479000
C	0.464315000	2.290855000	-3.019050000
H	2.138931000	-2.708145000	-2.858261000
C	0.375587000	-3.964586000	-2.752749000
C	1.980122000	-3.550189000	-0.864843000
H	-1.447363000	-4.984316000	-2.176671000
C	-0.113702000	-4.883481000	-0.470869000
H	1.307801000	-4.295755000	1.056990000
H	-5.237249000	-2.226190000	2.227164000
C	-5.322478000	-0.061078000	2.163691000
C	-3.776866000	-1.413698000	3.607047000
C	-3.574662000	1.087425000	3.550556000
H	-2.230339000	-0.257566000	4.592534000
H	-4.889252000	2.062884000	2.128254000
H	-4.098942000	1.505876000	-4.964356000
H	-0.171474000	4.165453000	-1.249739000
H	-1.829317000	3.614621000	-0.942190000
H	-1.384411000	4.244372000	-2.545613000
H	0.205896000	2.791456000	-3.966379000
H	0.760277000	1.263347000	-3.242973000
H	1.322158000	2.809701000	-2.581968000
H	0.907146000	-4.883737000	-3.028218000
H	-0.057727000	-3.558429000	-3.676003000
H	2.693248000	-2.841775000	-0.423403000
H	2.540390000	-4.459961000	-1.114314000
H	-0.899135000	-5.137498000	0.253104000
H	0.405473000	-5.818232000	-0.715731000
H	-6.051435000	0.016072000	2.979572000
H	-5.892267000	-0.035744000	1.225641000
H	-3.234043000	-2.362599000	3.709017000
H	-4.478095000	-1.357474000	4.448549000
H	-2.885378000	1.940225000	3.612502000
H	-4.273368000	1.182570000	4.390717000
C	4.480771000	0.731076000	-0.414271000
H	5.233496000	1.176602000	-1.073477000
H	3.929435000	-0.002150000	-1.018190000
C	5.201149000	-0.039720000	0.686937000
C	6.307178000	-0.975012000	0.198422000
H	5.614707000	0.670377000	1.425314000
C	5.050941000	-1.866185000	2.072129000
C	6.401157000	-2.008901000	1.333901000
H	5.992740000	-1.449847000	-0.737817000
H	7.249874000	-0.453366000	0.013679000
H	5.197125000	-1.508546000	3.100729000
H	4.473166000	-2.793043000	2.111172000
H	6.556131000	-3.019795000	0.949868000
H	7.234046000	-1.784773000	2.005705000
O	4.278245000	-0.915599000	1.332907000

6_{O5Z}

Sum of electronic and zero-point Energies = -2163.563953
Sum of electronic and thermal Free Energies = -2163.642327
Esolv = -2164.8550453

Au	0.504672000	0.822538000	0.080085000
P	-1.535980000	-0.270641000	0.257222000
C	-1.912206000	-0.612648000	2.027434000
C	-2.901099000	0.885744000	-0.406005000
C	-1.403938000	-1.958005000	-0.603238000
C	-3.156728000	-1.183500000	2.355706000
C	-1.016896000	-0.320129000	3.080500000
C	-3.141239000	1.968065000	0.674246000
C	-4.244340000	0.202431000	-0.744922000
C	-2.378570000	1.590303000	-1.683338000
C	-2.592934000	-2.916073000	-0.368292000
C	-1.220046000	-1.738597000	-2.121360000
C	-0.130057000	-2.636468000	-0.041052000
H	-3.865863000	-1.412264000	1.570150000
C	-3.514532000	-1.468112000	3.667583000
C	-1.391337000	-0.618372000	4.397178000
N	0.270239000	0.286225000	2.845633000
H	-3.512906000	1.509916000	1.597390000
H	-2.191600000	2.465298000	0.919093000
C	-4.159501000	3.006570000	0.169946000
C	-5.261325000	1.251209000	-1.237694000
H	-4.659819000	-0.303880000	0.132369000
H	-4.101113000	-0.553720000	-1.525032000

C	-3.400604000	2.627155000	-2.182279000
H	-2.180658000	0.860011000	-2.475121000
H	-1.424058000	2.086421000	-1.465268000
H	-2.711243000	-3.112012000	0.702538000
H	-3.528425000	-2.476689000	-0.730277000
C	-2.342174000	-4.254036000	-1.092636000
C	-0.964130000	-3.083813000	-2.824099000
H	-2.118189000	-1.278814000	-2.549740000
H	-0.382539000	-1.051005000	-2.302776000
C	0.119131000	-3.979147000	-0.751115000
H	-0.241085000	-2.800795000	1.038663000
H	0.737688000	-1.977174000	-0.183790000
H	-4.484161000	-1.907602000	3.879938000
C	-2.622156000	-1.186807000	4.697828000
H	-0.698490000	-0.395542000	5.203519000
C	1.378187000	-0.586176000	3.239967000
C	0.378014000	1.606171000	3.471053000
H	-4.311769000	3.752712000	0.959285000
C	-5.488213000	2.303617000	-0.143470000
C	-3.624888000	3.687854000	-1.096547000
H	-6.204130000	0.736473000	-1.458987000
C	-4.727966000	1.928587000	-2.506205000
H	-2.998719000	3.099177000	-3.087332000
H	-3.208569000	-4.903804000	-0.919803000
C	-1.078118000	-4.911957000	-0.524334000
C	-2.167061000	-4.010151000	-2.597484000
C	0.304596000	-3.733303000	-2.255147000
H	-0.836524000	-2.895399000	-3.897075000
H	1.027101000	-4.430021000	-0.332106000
H	-2.882886000	-1.405575000	5.728988000
H	2.323253000	-0.123596000	2.939358000
H	1.289339000	-1.549127000	2.732540000
H	1.425001000	-0.768123000	4.325290000
H	0.362087000	1.571018000	4.571803000
H	-0.444858000	2.241694000	3.135109000
H	1.321864000	2.070387000	3.167389000
H	-6.232560000	3.037034000	-0.476630000
H	-5.891274000	1.831531000	0.762001000
H	-2.685801000	4.213803000	-0.877969000
H	-4.337732000	4.443980000	-1.447607000
H	-4.584289000	1.186953000	-3.302923000
H	-5.457432000	2.657694000	-2.879412000
H	-0.901322000	-5.877275000	-1.014249000
H	-1.204057000	-5.116906000	0.546691000
H	-3.076148000	-3.562588000	-3.020279000
H	-2.012424000	-4.963502000	-3.117318000
H	1.173430000	-3.085205000	-2.431586000
H	0.505790000	-4.680944000	-2.769490000
C	5.536726000	0.374757000	0.281184000
H	5.265578000	1.235566000	0.917825000
C	4.217562000	-0.269680000	-0.217362000
C	6.544364000	0.842945000	-0.771204000
H	6.553657000	0.168211000	-1.634139000
H	6.336901000	1.853147000	-1.133192000
C	7.626694000	-0.528928000	0.833290000
H	8.148989000	-0.514342000	1.795319000
H	7.934280000	-1.433522000	0.290840000
C	7.861432000	0.729465000	-0.000155000
H	7.998794000	1.600638000	0.649518000
H	8.738350000	0.651496000	-0.647693000
C	4.453450000	-1.523840000	-1.056035000
H	5.064712000	-2.222890000	-0.481543000
H	3.506919000	-2.013486000	-1.303448000
H	4.977919000	-1.320673000	-1.994223000
H	3.707094000	-0.588196000	0.701294000
C	3.343990000	0.782262000	-0.867460000
C	2.634478000	1.699044000	-0.037454000
C	3.264191000	0.963398000	-2.247327000
C	1.895911000	2.765779000	-0.607766000
H	2.841255000	1.703378000	1.032290000
C	2.535731000	2.021664000	-2.806613000
H	3.794655000	0.285406000	-2.907628000
C	1.848194000	2.919625000	-2.002459000
H	1.456211000	3.521001000	0.038317000
H	2.517413000	2.141382000	-3.885986000
H	1.297354000	3.746685000	-2.438231000
O	6.216049000	-0.586523000	1.078962000

6$_{O5E}$

Sum of electronic and zero-point Energies =-2163.560449
Sum of electronic and thermal Free Energies =-2163.637747
Esolv = -2164.85528557

Au	0.499862000	-1.082908000	0.009773000
P	-1.279765000	0.399825000	0.259250000
C	-1.576436000	0.729343000	2.047679000
C	-0.860286000	2.069626000	-0.541087000
C	-2.845752000	-0.460023000	-0.417872000
C	-2.683118000	1.519162000	2.414959000
C	-0.759918000	0.211969000	3.077898000
C	0.529444000	2.477966000	0.005041000
C	-1.845537000	3.219473000	-0.232979000
C	-0.757454000	1.885758000	-2.071564000
C	-4.053291000	0.467406000	-0.678416000
C	-2.492624000	-1.180226000	-1.743266000
C	-3.249327000	-1.535274000	0.620099000
H	-3.331444000	1.923685000	1.648380000
C	-2.979516000	1.803496000	3.741988000
C	-1.070193000	0.512460000	4.410816000
N	0.382649000	-0.624699000	2.804706000
H	0.478342000	2.612321000	1.093427000
H	1.255356000	1.676642000	-0.190461000
C	1.003932000	3.781819000	-0.662094000
C	-1.371183000	4.518242000	-0.914931000
H	-1.896982000	3.389523000	0.847295000
H	-2.855383000	2.974671000	-0.578851000
C	-0.276300000	3.190442000	-2.731630000
H	-1.735248000	1.617375000	-2.487578000
H	-0.065547000	1.065322000	-2.306531000
H	-4.348388000	0.993159000	0.235166000
H	-3.797790000	1.224865000	-1.428001000
C	-5.254957000	-0.357250000	-1.182178000
C	-3.696950000	-1.992841000	-2.251839000
H	-2.189411000	-0.457896000	-2.508367000
H	-1.636702000	-1.847815000	-1.582457000
C	-4.450058000	-2.349280000	0.105734000
H	-3.508563000	-1.064650000	1.574840000
H	-2.398591000	-2.205583000	0.810223000
H	-3.842604000	2.416069000	3.983370000
C	-2.163274000	1.298688000	4.749763000
H	-0.437282000	0.116080000	5.199506000
C	0.222823000	-1.968835000	3.364888000
C	1.636933000	-0.008848000	3.244604000
H	1.989183000	4.041869000	-0.254893000
C	0.005784000	4.908293000	-0.362323000
C	1.103628000	3.572144000	-2.179977000
H	-2.098351000	5.307643000	-0.689659000
C	-1.281801000	4.310873000	-2.432549000
H	-0.211672000	3.027397000	-3.814323000
H	-6.095237000	0.327959000	-1.347081000
C	-5.638546000	-1.406104000	-0.129161000

C	-4.885653000	-1.054148000	-2.497616000
C	-4.079366000	-3.050065000	-1.207817000
H	-3.410343000	-2.482500000	-3.190788000
H	-4.713018000	-3.095241000	0.865603000
H	-2.377014000	1.512631000	5.792677000
H	1.060671000	-2.594190000	3.040730000
H	-0.705365000	-2.413966000	2.998459000
H	0.202992000	-1.983437000	4.465971000
H	1.707234000	0.101032000	4.338227000
H	1.742205000	0.979413000	2.792154000
H	2.476444000	-0.627762000	2.916150000
H	0.346136000	5.844802000	-0.820814000
H	-0.054660000	5.086501000	0.719100000
H	1.833168000	2.784735000	-2.410307000
H	1.461793000	4.489184000	-2.663661000
H	-2.268145000	4.055878000	-2.841871000
H	-0.967087000	5.240837000	-2.921580000
H	-6.511082000	-1.977314000	-0.468770000
H	-5.926170000	-0.916132000	0.810177000
H	-4.632307000	-0.310873000	-3.264922000
H	-5.743613000	-1.622220000	-2.877336000
H	-3.244656000	-3.745342000	-1.045516000
H	-4.924918000	-3.648891000	-1.567739000
C	4.563568000	-1.359470000	-0.234894000
C	3.360943000	-1.984239000	-0.910173000
C	3.285241000	-2.079673000	-2.298179000
C	2.303746000	-2.565943000	-0.151293000
C	2.226488000	-2.736605000	-2.940062000
H	4.095121000	-1.674959000	-2.899454000
C	1.245544000	-3.250600000	-0.802282000
H	2.438877000	-2.706744000	0.918256000
C	1.207749000	-3.323078000	-2.205713000
H	2.223828000	-2.809952000	-4.023649000
H	0.543444000	-3.835965000	-0.213580000
H	0.409619000	-3.868182000	-2.698845000
C	5.054353000	-2.172093000	0.968338000
H	4.345284000	-2.148209000	1.802762000
H	5.224913000	-3.216860000	0.692274000
H	5.987700000	-1.747206000	1.338675000
H	5.373822000	-1.346195000	-0.976344000
C	4.284213000	0.120317000	0.157648000
C	4.331658000	1.096545000	-1.028057000
H	3.308319000	0.151555000	0.668065000
C	6.029280000	1.651499000	0.509815000
C	5.779458000	1.591448000	-0.991249000
H	3.649250000	1.934063000	-0.849402000
H	4.047087000	0.635511000	-1.977241000
H	5.678103000	2.604174000	0.932665000
H	7.078239000	1.519428000	0.790693000
H	5.918649000	2.555601000	-1.486549000
H	6.453370000	0.869457000	-1.465296000
O	5.275969000	0.572466000	1.070356000

6'$_{O5E}$

Sum of electronic and zero-point Energies = -2163.565387

Sum of electronic and thermal Free Energies = -2163.643148

Esolv = -2164.8570718

C	-3.416376000	-0.379404000	-0.982134000
C	-3.354038000	-0.039958000	-2.335371000
H	-3.901109000	0.826793000	-2.694267000
Au	-0.562747000	-0.726172000	-0.157397000
C	-2.690948000	-1.526270000	-0.552311000
H	-2.887683000	-1.928131000	0.440658000
C	-1.951438000	-2.300631000	-1.483463000
H	-1.498785000	-3.235442000	-1.163825000
C	-1.925275000	-1.935097000	-2.837641000
H	-1.378065000	-2.541143000	-3.552264000
C	-2.631439000	-0.812779000	-3.251439000
H	-2.631221000	-0.532497000	-4.300781000
P	1.518254000	0.194585000	0.309655000
C	1.801490000	0.228627000	2.128567000
C	2.854133000	-0.946068000	-0.417244000
C	1.551237000	1.995041000	-0.315069000
C	2.969844000	0.841883000	2.619384000
C	0.898090000	-0.324733000	3.063059000
C	2.528199000	-2.376223000	0.080156000
C	4.303880000	-0.619324000	0.005390000
C	2.756516000	-0.921952000	-1.958832000
C	2.956650000	2.611958000	-0.490205000
C	0.818420000	2.058587000	-1.679418000
C	0.748925000	2.842935000	0.700974000
H	3.676101000	1.281177000	1.926060000
C	3.253298000	0.907307000	3.977803000
C	1.202999000	-0.257410000	4.428839000
N	-0.335946000	-0.950504000	2.656544000
H	2.595153000	-2.412835000	1.175267000
H	1.497497000	-2.641595000	-0.193596000
C	3.503828000	-3.394133000	-0.536603000
C	5.277925000	-1.635361000	-0.624909000
H	4.397679000	-0.670093000	1.095213000
H	4.584727000	0.392611000	-0.304510000
C	3.730365000	-1.946694000	-2.567824000
H	3.004886000	0.075509000	-2.339483000
H	1.727715000	-1.143838000	-2.273609000
H	3.513366000	2.596134000	0.452583000
H	3.535444000	2.039702000	-1.223826000
C	2.836962000	4.072259000	-0.969360000
C	0.713314000	3.515512000	-2.164859000
H	1.344660000	1.458326000	-2.429448000
H	-0.187299000	1.629070000	-1.578289000
C	0.637897000	4.297595000	0.209477000
H	1.236297000	2.824847000	1.681934000
H	-0.254842000	2.413955000	0.831538000
H	4.163897000	1.391059000	4.317231000
C	2.364072000	0.348327000	4.890956000
H	0.509032000	-0.689292000	5.144186000
C	-1.508527000	-0.267512000	3.206960000
C	-0.359639000	-2.382855000	2.959159000
H	3.242251000	-4.393473000	-0.167423000
C	4.939898000	-3.045413000	-0.123663000
C	3.381498000	-3.354705000	-2.066416000
H	6.296379000	-1.367003000	-0.319262000
C	5.164948000	-1.589399000	-2.154590000
H	3.637053000	-1.907021000	-3.659972000
H	3.848625000	4.482970000	-1.072716000
C	2.046401000	4.890723000	0.060874000
C	2.119067000	4.110084000	-2.324230000
C	-0.083921000	4.337713000	-1.143866000
H	0.196116000	3.519624000	-3.132282000
H	0.070248000	4.870960000	0.952397000
H	2.571052000	0.385518000	5.956274000
H	-2.412147000	-0.706027000	2.772879000
H	-1.477135000	0.791726000	2.942001000
H	-1.589831000	-0.350067000	4.302183000
H	-0.355178000	-2.598902000	4.039417000
H	0.505857000	-2.871460000	2.506525000
H	-1.266694000	-2.823942000	2.533622000
H	5.641136000	-3.775210000	-0.546426000
H	5.045854000	-3.097313000	0.967694000
H	2.362747000	-3.626380000	-2.374204000

H	4.056096000	-4.092889000	-2.516950000
H	5.430134000	-0.591348000	-2.527451000
H	5.874174000	-2.294324000	-2.605245000
H	1.982036000	5.937255000	-0.260978000
H	2.564405000	4.887844000	1.028811000
H	2.689629000	3.546005000	-3.073747000
H	2.052991000	5.142951000	-2.687321000
H	-1.101451000	3.937589000	-1.044677000
H	-0.182295000	5.374726000	-1.487549000
C	-4.288805000	0.382685000	-0.010148000
C	-5.781282000	0.170721000	-0.338244000
H	-5.980162000	0.595915000	-1.338440000
C	-7.758232000	-1.079427000	0.047594000
C	-7.705313000	0.114160000	1.000611000
H	-8.322600000	-0.826340000	-0.856251000
H	-8.222725000	-1.960606000	0.497255000
H	-7.599082000	-0.208241000	2.045514000
H	-8.587890000	0.758630000	0.932291000
C	-6.279448000	-1.275200000	-0.289404000
H	-5.774174000	-1.821186000	0.518124000
H	-6.111788000	-1.817044000	-1.223754000
O	-6.549398000	0.874172000	0.629157000
C	-3.950692000	1.876380000	0.048086000
H	-2.905900000	2.029273000	0.335878000
H	-4.109708000	2.364116000	-0.919399000
H	-4.596651000	2.367899000	0.778070000
H	-4.135679000	-0.041284000	0.990932000

6_{O6}

Sum of electronic and zero-point Energies = -2124.289998
Sum of electronic and thermal Free Energies = -2124.365322
Esolv = -2125.5486976

C	-3.413200000	1.426611000	-0.697213000
C	-2.558375000	2.169667000	0.167009000
H	-2.744273000	2.134361000	1.239868000
Au	-0.568580000	0.997120000	0.167457000
C	-3.343140000	1.693003000	-2.065321000
H	-3.991051000	1.156193000	-2.750959000
C	-2.476968000	2.666751000	-2.577625000
H	-2.468355000	2.860637000	-3.646230000
C	-1.642919000	3.393576000	-1.738937000
H	-0.986390000	4.160006000	-2.137496000
C	-1.682450000	3.153458000	-0.356624000
H	-1.124377000	3.792389000	0.322631000
P	1.316131000	-0.358482000	0.227884000
C	1.747138000	-0.744784000	1.976610000
C	0.921962000	-2.017016000	-0.607454000
C	2.777320000	0.613777000	-0.521158000
C	2.930388000	-1.461951000	2.237192000
C	0.955915000	-0.346327000	3.077341000
C	-0.386611000	-2.530790000	0.042070000
C	1.996099000	-3.114660000	-0.438169000
C	0.669591000	-1.780699000	-2.113199000
C	3.999392000	-0.235179000	-0.936528000
C	2.275081000	1.382651000	-1.769293000
C	3.217315000	1.652675000	0.538551000
H	3.559871000	-1.774557000	1.413629000
C	3.327201000	-1.786655000	3.528356000
C	1.367525000	-0.686941000	4.372464000
N	-0.262420000	0.407657000	2.912546000
H	-0.225972000	-2.705918000	1.113822000
H	-1.173118000	-1.768528000	-0.050150000
C	-0.845833000	-3.833793000	-0.636533000
C	1.535437000	-4.413505000	-1.129861000
H	2.158528000	-3.320224000	0.625020000
H	2.953145000	-2.796080000	-0.864243000
C	0.205092000	-3.086294000	-2.783613000
H	1.588085000	-1.436531000	-2.602523000
H	-0.086901000	-0.995767000	-2.249497000
H	4.397440000	-0.795748000	-0.084463000
H	3.714711000	-0.962201000	-1.705473000
C	5.111990000	0.676886000	-1.491390000
C	3.390176000	2.282519000	-2.331215000
H	1.940456000	0.685115000	-2.544637000
H	1.405544000	1.995468000	-1.498975000
C	4.328559000	2.553942000	-0.029002000
H	3.581165000	1.147646000	1.440003000
H	2.355146000	2.266213000	0.837361000
H	4.247697000	-2.339527000	3.687816000
C	2.536739000	-1.398860000	4.606153000
H	0.753997000	-0.382842000	5.215652000
C	-0.192433000	1.719682000	3.559763000
C	-1.442831000	-0.342065000	3.346518000
H	-1.773860000	-4.167506000	-0.155999000
C	0.238748000	-4.907450000	-0.475529000
C	-1.095193000	-3.571339000	-2.128516000
H	2.323902000	-5.165392000	-1.004867000
C	1.295884000	-4.154390000	-2.623233000
H	0.033176000	-2.886837000	-3.848336000
H	5.966431000	0.046381000	-1.765567000
C	5.535468000	1.687350000	-0.416537000
C	4.596377000	1.421479000	-2.729281000
C	3.811419000	3.301942000	-1.264742000
H	2.999580000	2.805146000	-3.213093000
H	4.621119000	3.271982000	0.746728000
H	2.828745000	-1.646665000	5.622236000
H	-1.088671000	2.293592000	3.302955000
H	0.683303000	2.263239000	3.197266000
H	-0.136164000	1.664210000	4.658231000
H	-1.457141000	-0.542667000	4.429513000
H	-1.489341000	-1.296737000	2.818013000
H	-2.341868000	0.232223000	3.101913000
H	-0.087187000	-5.844871000	-0.942531000
H	0.406785000	-5.122930000	0.587617000
H	-1.886573000	-2.820886000	-2.257389000
H	-1.444380000	-4.488365000	-2.618636000
H	2.224474000	-3.824808000	-3.107503000
H	0.991301000	-5.082651000	-3.121802000
H	6.347111000	2.320187000	-0.795574000
H	5.926794000	1.164391000	0.465829000
H	4.313151000	0.707116000	-3.513409000
H	5.389907000	2.052950000	-3.147034000
H	2.961668000	3.942676000	-0.993276000
H	4.592682000	3.962348000	-1.660453000
C	-5.788901000	1.237727000	0.074221000
H	-6.161433000	1.543115000	-0.920002000
H	-5.664589000	2.141556000	0.678857000
C	-4.439819000	0.498986000	-0.097274000
C	-4.689575000	-0.804587000	-0.870148000
H	-4.109083000	0.233710000	0.915806000
C	-7.039356000	-0.742757000	0.007396000
C	-5.802580000	-1.610783000	-0.194436000
H	-4.996476000	-0.573916000	-1.899123000
H	-3.766282000	-1.391021000	-0.938863000
H	-7.805898000	-1.265630000	0.584751000
H	-7.475665000	-0.470594000	-0.968951000
H	-6.059070000	-2.489701000	-0.796356000
H	-5.460727000	-1.974474000	0.782584000
O	-6.729003000	0.433841000	0.739402000

6_{O6Z}

Sum of electronic and zero-point Energies = -2163.564952
Sum of electronic and thermal Free Energies = -2163.641641
Esolv = -2164.8602838

C	3.380677000	0.921989000	0.989228000
C	2.546224000	1.938800000	0.437866000
H	2.712757000	2.251494000	-0.592355000
Au	0.501773000	0.913868000	0.116272000
C	3.327828000	0.719137000	2.367623000
H	3.953190000	-0.042455000	2.821342000
C	2.506884000	1.499526000	3.193183000
H	2.512644000	1.324451000	4.265095000
C	1.699878000	2.495240000	2.662447000
H	1.078897000	3.109814000	3.305855000
C	1.717152000	2.724673000	1.276459000
H	1.181579000	3.573493000	0.859448000
P	-1.439417000	-0.293666000	-0.289798000
C	-1.712239000	-0.467999000	-2.102067000
C	-1.230169000	-2.043892000	0.435112000
C	-2.915278000	0.699031000	0.379992000
C	-2.791314000	-1.253468000	-2.550400000
C	-0.886998000	0.144175000	-3.071758000
C	-0.317311000	-2.830424000	-0.536147000
C	-2.539759000	-2.834113000	0.651920000
C	-0.498783000	-1.932121000	1.797046000
C	-4.308883000	0.153841000	-0.004643000
C	-2.810760000	0.786100000	1.918870000
C	-2.786513000	2.125616000	-0.209556000
H	-3.435172000	-1.741900000	-1.829630000
C	-3.063816000	-1.429492000	-3.901221000
C	-1.181048000	-0.036747000	-4.429598000
N	0.257059000	0.943808000	-2.709140000
H	-0.798859000	-2.931701000	-1.514870000
H	0.620589000	-2.278501000	-0.693069000
C	-0.013501000	-4.227724000	0.033790000
C	-2.225330000	-4.236080000	1.210364000
H	-3.091772000	-2.945141000	-0.287122000
H	-3.191403000	-2.305009000	1.356321000
C	-0.198360000	-3.332172000	2.361602000
H	-1.103342000	-1.368155000	2.515578000
H	0.439425000	-1.375952000	1.667438000
H	-4.412375000	0.122977000	-1.094339000
H	-4.449230000	-0.865218000	0.370451000
C	-5.409426000	1.067121000	0.572434000
C	-3.911802000	1.707825000	2.473490000
H	-2.921871000	-0.209639000	2.363748000
H	-1.820253000	1.163310000	2.207569000
C	-3.889561000	3.039638000	0.352906000
H	-2.859767000	2.083610000	-1.304043000
H	-1.800676000	2.544295000	0.036662000
H	-3.904263000	-2.044600000	-4.207338000
C	-2.254512000	-0.811164000	-4.849742000
H	-0.548707000	0.441087000	-5.172322000
C	0.096951000	2.349165000	-3.088394000
C	1.506588000	0.387686000	-3.232818000
H	0.627560000	-4.760120000	-0.679418000
C	-1.329512000	-4.996081000	0.222467000
C	0.704151000	-4.095325000	1.383383000
H	-3.172376000	-4.773200000	1.342008000
C	-1.511206000	-4.101455000	2.561226000
H	0.312546000	-3.212925000	3.325004000
H	-6.383158000	0.644888000	0.296331000
C	-5.266619000	2.474789000	-0.020554000
C	-5.286312000	1.134917000	2.100478000
C	-3.757985000	3.115069000	1.880462000
H	-3.810528000	1.750865000	3.564795000
H	-3.766766000	4.039385000	-0.081560000
H	-2.455286000	-0.932681000	-5.909953000
H	0.941307000	2.924607000	-2.695633000
H	-0.822907000	2.747312000	-2.654776000
H	0.062591000	2.503846000	-4.178535000
H	1.574382000	0.419398000	-4.331541000
H	1.610870000	-0.651204000	-2.911146000
H	2.348426000	0.961458000	-2.833492000
H	-1.125220000	-6.005133000	0.600677000
H	-1.840898000	-5.116139000	-0.741504000
H	1.658777000	-3.567414000	1.256195000
H	0.941367000	-5.088672000	1.783651000
H	-2.153888000	-3.578637000	3.281637000
H	-1.307053000	-5.094192000	2.980531000
H	-6.058737000	3.129090000	0.363480000
H	-5.381823000	2.442066000	-1.111705000
H	-5.413084000	0.136247000	2.538736000
H	-6.082854000	1.765423000	2.513942000
H	-2.784285000	3.540591000	2.158317000
H	-4.524637000	3.782834000	2.291892000
C	4.330686000	0.186361000	0.074828000
H	3.802214000	0.016415000	-0.873166000
C	5.555710000	1.068036000	-0.319723000
C	4.807211000	-1.183388000	0.573524000
H	5.370138000	-1.080494000	1.509892000
H	3.945988000	-1.824925000	0.794796000
C	6.828925000	-0.875692000	-0.878567000
H	7.394086000	-1.260642000	-1.731399000
H	7.536212000	-0.752043000	-0.044427000
C	5.707545000	-1.828761000	-0.482868000
H	5.114851000	-2.072751000	-1.373709000
H	6.131639000	-2.767285000	-0.108827000
O	6.319267000	0.382984000	-1.299171000
C	6.399277000	1.562343000	0.853244000
H	7.266779000	2.101999000	0.464587000
H	5.825014000	2.249427000	1.481450000
H	6.764644000	0.755102000	1.493699000
H	5.178229000	1.950288000	-0.851773000

6_{O6E}

Sum of electronic and zero-point Energies = -2163.567580
Sum of electronic and thermal Free Energies = -2163.643385
Esolv = -2164.8625418

C	3.509011000	1.188102000	-0.925228000
C	3.488260000	1.062484000	-2.313154000
H	4.218870000	0.429270000	-2.807034000
Au	0.529790000	0.906041000	-0.059235000
C	2.547937000	2.051753000	-0.324573000
H	2.679836000	2.326491000	0.721192000
C	1.637018000	2.785842000	-1.126571000
H	1.023590000	3.560216000	-0.672692000
C	1.651231000	2.633551000	-2.524516000
H	0.966877000	3.208937000	-3.139161000
C	2.571716000	1.772695000	-3.101455000
H	2.602989000	1.661389000	-4.181411000
P	-1.445336000	-0.279299000	0.285607000
C	-1.706899000	-0.557311000	2.086927000
C	-2.895375000	0.797580000	-0.335364000
C	-1.304895000	-1.995297000	-0.513489000
C	-2.904524000	-1.163774000	2.511780000
C	-0.769087000	-0.177742000	3.072488000
C	-3.115803000	1.910791000	0.717219000
C	-4.225964000	0.047011000	-0.564085000

C	-2.484252000	1.471921000	-1.668253000
C	-2.445289000	-2.981382000	-0.174987000
C	-1.212187000	-1.831502000	-2.047140000
C	0.019639000	-2.608984000	0.002592000
H	-3.645298000	-1.458475000	1.779461000
C	-3.176010000	-1.400547000	3.853443000
C	-1.055821000	-0.428574000	4.420720000
N	0.476033000	0.468467000	2.736514000
H	-3.409759000	1.474399000	1.678044000
H	-2.175306000	2.455741000	0.883963000
C	-4.207639000	2.885795000	0.240594000
C	-5.316404000	1.032479000	-1.028973000
H	-4.564206000	-0.442362000	0.354923000
H	-4.097927000	-0.731558000	-1.324449000
C	-3.579386000	2.445251000	-2.139609000
H	-2.302506000	0.719687000	-2.443253000
H	-1.541581000	2.016231000	-1.528820000
H	-2.495966000	-3.138666000	0.907408000
H	-3.414575000	-2.588562000	-0.498963000
C	-2.188796000	-4.338275000	-0.860688000
C	-0.949427000	-3.195037000	-2.710778000
H	-2.147360000	-1.417824000	-2.442087000
H	-0.409440000	-1.126006000	-2.302153000
C	0.276405000	-3.969965000	-0.668961000
H	-0.028236000	-2.735027000	1.092146000
H	0.855136000	-1.928758000	-0.211331000
H	-4.111819000	-1.869753000	4.140706000
C	-2.241409000	-1.033221000	4.817005000
H	-0.329877000	-0.139473000	5.175181000
C	1.637434000	-0.347981000	3.095626000
C	0.568762000	1.813272000	3.309723000
H	-4.343238000	3.655717000	1.009859000
C	-5.521184000	2.116630000	0.038198000
C	-3.782539000	3.537787000	-1.081578000
H	-6.247555000	0.470752000	-1.171070000
C	-4.892546000	1.680621000	-2.352884000
H	-3.255208000	2.897490000	-3.085069000
H	-3.021479000	-5.008118000	-0.614335000
C	-0.873297000	-4.931725000	-0.340025000
C	-2.105710000	-4.149423000	-2.381229000
C	0.370375000	-3.779316000	-2.189359000
H	-0.887628000	-3.046065000	-3.795704000
H	1.221250000	-4.373234000	-0.284849000
H	-2.434391000	-1.213538000	5.870262000
H	2.544310000	0.138391000	2.724771000
H	1.557492000	-1.330836000	2.626550000
H	1.750644000	-0.489280000	4.182141000
H	0.618516000	1.817161000	4.409923000
H	-0.295933000	2.406219000	3.002296000
H	1.474816000	2.300410000	2.935624000
H	-6.316128000	2.804910000	-0.273639000
H	-5.846274000	1.664156000	0.984110000
H	-2.855820000	4.110753000	-0.942416000
H	-4.547835000	4.249420000	-1.414679000
H	-4.766264000	0.914592000	-3.129172000
H	-5.674521000	2.363961000	-2.705736000
H	-0.690287000	-5.909389000	-0.802383000
H	-0.933080000	-5.098150000	0.743364000
H	-3.051595000	-3.748553000	-2.768725000
H	-1.947547000	-5.117441000	-2.872062000
H	1.205703000	-3.111834000	-2.439116000
H	0.575434000	-4.740171000	-2.676977000
C	4.596268000	0.589270000	-0.064416000
H	4.210570000	0.494384000	0.960370000
C	5.077681000	-0.822528000	-0.466891000
H	5.521841000	-0.771080000	-1.478234000
C	5.810424000	1.547781000	-0.024469000
H	6.174921000	1.692601000	-1.050989000
H	5.499922000	2.534838000	0.337472000
C	3.986601000	-1.876488000	-0.445896000
H	3.543637000	-1.940031000	0.552992000
H	3.198570000	-1.647006000	-1.167752000
H	4.412523000	-2.852649000	-0.690242000
C	7.240137000	-0.452913000	0.421404000
H	7.949390000	-0.930462000	1.102416000
H	7.682416000	-0.461803000	-0.589431000
C	6.928143000	0.974966000	0.846260000
H	6.619640000	0.974434000	1.898925000
H	7.827484000	1.596449000	0.771095000
O	6.071380000	-1.257689000	0.442771000

6’$_{O6E}$

Sum of electronic and zero-point Energies = -2163.565852
Sum of electronic and thermal Free Energies = -2163.643104
Esolv = -2164.8626656

C	3.591150000	-1.558195000	1.004650000
C	3.323900000	-2.091706000	2.264791000
H	3.947168000	-1.794486000	3.103910000
Au	0.610333000	-0.890801000	-0.093977000
C	2.771273000	-1.982725000	-0.075593000
H	3.051238000	-1.717414000	-1.091825000
C	1.762623000	-2.957854000	0.123888000
H	1.282333000	-3.419342000	-0.736264000
C	1.530930000	-3.477494000	1.412816000
H	0.776403000	-4.243576000	1.558628000
C	2.307636000	-3.033464000	2.471538000
H	2.150593000	-3.439246000	3.466498000
P	-1.366905000	0.325984000	-0.252355000
C	-1.726296000	0.657495000	-2.030884000
C	-2.761166000	-0.803424000	0.408453000
C	-1.235097000	2.018988000	0.597692000
C	-2.968275000	1.228511000	-2.369211000
C	-0.830374000	0.352697000	-3.080604000
C	-3.006538000	-1.893394000	-0.662879000
C	-4.098352000	-0.096962000	0.724843000
C	-2.266313000	-1.501346000	1.700662000
C	-2.410728000	2.981260000	0.313017000
C	-1.101644000	1.815686000	2.123241000
C	0.058613000	2.683637000	0.068568000
H	-3.678929000	1.466753000	-1.588358000
C	-3.323862000	1.500601000	-3.684289000
C	-1.201069000	0.641607000	-4.400521000
N	0.457594000	-0.249284000	-2.840678000
H	-3.360812000	-1.439302000	-1.594788000
H	-2.062000000	-2.407345000	-0.892997000
C	-4.046241000	-2.911093000	-0.159794000
C	-5.137100000	-1.124922000	1.216514000
H	-4.497656000	0.405466000	-0.161865000
H	-3.953630000	0.665108000	1.498628000
C	-3.309533000	-2.518016000	2.197513000
H	-2.068804000	-0.765595000	2.487247000
H	-1.316278000	-2.013778000	1.502486000
H	-2.491569000	3.165865000	-0.763137000
H	-3.360445000	2.551803000	0.648426000
C	-2.177313000	4.325881000	1.029755000
C	-0.865593000	3.168697000	2.818904000
H	-2.014311000	1.363388000	2.527613000
H	-0.272524000	1.128598000	2.340992000
C	0.291852000	4.033304000	0.772951000
H	-0.021692000	2.840880000	-1.015096000

H	0.917620000	2.021871000	0.241466000
H	-4.292628000	1.939338000	-3.901990000
C	-2.430300000	1.208329000	-4.710107000
H	-0.504989000	0.414355000	-5.202762000
C	1.559448000	0.665962000	-3.142886000
C	0.616142000	-1.526865000	-3.537500000
H	-4.200657000	-3.663276000	-0.942989000
C	-5.367662000	-2.185039000	0.130656000
C	-3.537098000	-3.586557000	1.120199000
H	-6.074092000	-0.592867000	1.420781000
C	-4.629983000	-1.796823000	2.498389000
H	-2.925162000	-2.986223000	3.112091000
H	-3.034029000	4.977549000	0.819647000
C	-0.891447000	4.970127000	0.495498000
C	-2.056248000	4.096687000	2.541993000
C	0.423250000	3.807982000	2.286034000
H	-0.774496000	2.991398000	3.897415000
H	1.216221000	4.474125000	0.379814000
H	-2.687696000	1.418834000	-5.743800000
H	2.504407000	0.200805000	-2.845331000
H	1.439364000	1.590623000	-2.574614000
H	1.629086000	0.920777000	-4.212506000
H	0.659605000	-1.429841000	-4.633623000
H	-0.213892000	-2.190756000	-3.284062000
H	1.550298000	-1.996938000	-3.212739000
H	-6.126923000	-2.903547000	0.462590000
H	-5.752898000	-1.716485000	-0.784337000
H	-2.603639000	-4.129065000	0.918094000
H	-4.265155000	-4.328355000	1.470571000
H	-4.484738000	-1.049310000	3.289229000
H	-5.374867000	-2.511014000	2.869957000
H	-0.724784000	5.940068000	0.979675000
H	-0.981980000	5.163217000	-0.581363000
H	-2.981978000	3.657543000	2.936743000
H	-1.915251000	5.054278000	3.057785000
H	1.283362000	3.163413000	2.504182000
H	0.606148000	4.763535000	2.792512000
C	4.780533000	-0.651674000	0.788097000
C	5.733807000	-1.194247000	-0.296593000
C	4.390654000	0.814381000	0.470200000
H	5.340735000	-0.611382000	1.731969000
C	6.904243000	-0.237879000	-0.516882000
H	5.190662000	-1.308153000	-1.245284000
H	6.086182000	-2.192706000	-0.015411000
H	3.781010000	0.813300000	-0.455725000
C	6.375425000	1.162102000	-0.787528000
H	7.524483000	-0.576477000	-1.354263000
H	7.543891000	-0.212163000	0.373330000
H	7.183974000	1.893902000	-0.861565000
H	5.814996000	1.179607000	-1.739236000
O	5.543391000	1.605991000	0.271659000
C	3.619167000	1.478313000	1.595564000
H	2.675082000	0.962460000	1.791912000
H	4.216907000	1.476884000	2.512347000
H	3.409230000	2.518053000	1.333434000

$TS2_{OH5}$

Sum of electronic and zero-point Energies = -2124.464027
Sum of electronic and thermal Free Energies = -2124.537614
Esolv = -2125.8810373

C	-2.279486000	0.965692000	-0.989723000
C	-2.308253000	2.362679000	-1.141662000
H	-2.178562000	3.017149000	-0.284392000
Au	-1.014783000	0.138276000	0.506584000
C	-2.481378000	0.137492000	-2.108356000
H	-2.465651000	-0.943449000	-2.006235000
C	-2.642841000	0.709622000	-3.368958000
H	-2.766027000	0.063486000	-4.233399000
C	-2.644819000	2.096423000	-3.525362000
H	-2.783804000	2.534146000	-4.508558000
C	-2.475534000	2.916531000	-2.409931000
H	-2.476418000	3.996888000	-2.520789000
P	1.339658000	-0.212936000	0.238268000
C	1.870852000	-0.829929000	1.882381000
C	1.643041000	-1.611548000	-1.007716000
C	2.237755000	1.431993000	-0.079786000
C	3.230313000	-1.094587000	2.131775000
C	0.953670000	-1.033270000	2.931202000
C	0.637572000	-2.737253000	-0.662615000
C	3.065477000	-2.214939000	-0.962932000
C	1.353599000	-1.097459000	-2.435565000
C	3.720036000	1.288807000	-0.491373000
C	1.486480000	2.206861000	-1.188674000
C	2.150396000	2.252511000	1.229557000
H	3.960091000	-0.940570000	1.346967000
C	3.672346000	-1.551438000	3.367315000
C	1.407056000	-1.498520000	4.169821000
N	-0.472654000	-0.765867000	2.769543000
H	0.830909000	-3.113321000	0.350845000
H	-0.389707000	-2.340802000	-0.676727000
C	0.755735000	-3.888572000	-1.678479000
C	3.179755000	-3.358975000	-1.991604000
H	3.273693000	-2.616783000	0.034144000
H	3.820361000	-1.451747000	-1.179452000
C	1.464187000	-2.257033000	-3.443242000
H	2.074777000	-0.319573000	-2.709926000
H	0.353501000	-0.646820000	-2.485046000
H	4.289060000	0.751003000	0.274068000
H	3.804051000	0.723180000	-1.426083000
C	4.343422000	2.686322000	-0.684699000
C	2.122055000	3.595294000	-1.388691000
H	1.510157000	1.654629000	-2.133606000
H	0.431643000	2.321896000	-0.912060000
C	2.780362000	3.642691000	1.023900000
H	2.667994000	1.732605000	2.043364000
H	1.097185000	2.364059000	1.530916000
H	4.728473000	-1.744624000	3.525912000
C	2.753998000	-1.758447000	4.392327000
H	0.701711000	-1.660209000	4.979399000
C	-0.926101000	0.273924000	3.714228000
C	-1.261090000	-2.000671000	2.936327000
H	0.030856000	-4.666391000	-1.408675000
C	2.177773000	-4.464756000	-1.636134000
C	0.453754000	-3.355778000	-3.086174000
H	4.200720000	-3.755032000	-1.946694000
C	2.888369000	-2.827986000	-3.401327000
H	1.250169000	-1.865192000	-4.444674000
H	5.393755000	2.554120000	-0.968822000
C	4.254728000	3.476233000	0.628149000
C	3.593417000	3.434170000	-1.794470000
C	2.028604000	4.394438000	-0.082263000
H	1.572127000	4.113105000	-2.183815000
H	2.709464000	4.196079000	1.967815000
H	3.082080000	-2.117939000	5.362551000
H	-1.975362000	0.513043000	3.510732000
H	-0.324107000	1.175063000	3.584067000
H	-0.853444000	-0.048724000	4.760323000
H	-1.183840000	-2.412364000	3.950469000
H	-0.919337000	-2.754097000	2.224685000
H	-2.318581000	-1.786013000	2.746266000

H	2.265688000	-5.296486000	-2.344736000
H	2.395019000	-4.872732000	-0.640698000
H	-0.571190000	-2.961370000	-3.133692000
H	0.510635000	-4.171278000	-3.816427000
H	3.616199000	-2.054555000	-3.677852000
H	2.992814000	-3.635265000	-4.135535000
H	4.723146000	4.459755000	0.506683000
H	4.806973000	2.959907000	1.423782000
H	3.670630000	2.887871000	-2.743367000
H	4.048444000	4.417782000	-1.958952000
H	0.977457000	4.540642000	0.202771000
H	2.459763000	5.393072000	-0.218190000
C	-3.267754000	0.553829000	0.682866000
H	-3.197522000	1.513505000	1.203023000
H	-3.198934000	-0.277444000	1.405276000
C	-4.603663000	0.435555000	-0.008252000
H	-4.786574000	1.245308000	-0.713845000
C	-6.624950000	-0.878546000	-0.307697000
C	-6.820088000	-0.251000000	1.054656000
H	-5.361038000	0.927414000	1.969849000
H	-7.100892000	-0.270251000	-1.082286000
H	-7.067967000	-1.876321000	-0.345188000
H	-6.644532000	-0.942437000	1.881773000
H	-7.748267000	0.303135000	1.193426000
C	-5.102314000	-0.912262000	-0.490704000
H	-4.653266000	-1.721231000	0.096762000
H	-4.831344000	-1.058148000	-1.537217000
O	-5.723814000	0.770635000	1.081432000

TS2$_{OH5Z}$

Sum of electronic and zero-point Energies = -2163.736006
Sum of electronic and thermal Free Energies = -2163.810033
Esolv = -2165.1926657

Au	0.930693000	-0.321950000	0.381346000
P	-1.444321000	-0.057273000	0.257666000
C	-2.063760000	-0.686962000	1.864815000
C	-1.875372000	1.788317000	0.172238000
C	-2.164038000	-1.166859000	-1.109928000
C	-3.447700000	-0.729223000	2.116707000
C	-1.192266000	-1.155852000	2.866518000
C	-1.022435000	2.493818000	1.254996000
C	-3.359947000	2.117971000	0.449448000
C	-1.480490000	2.336832000	-1.217171000
C	-3.631104000	-0.854768000	-1.481173000
C	-1.290065000	-1.028848000	-2.378925000
C	-2.065890000	-2.629096000	-0.613162000
H	-4.142626000	-0.387830000	1.359717000
C	-3.957888000	-1.203493000	3.318966000
C	-1.715432000	-1.623464000	4.076609000
N	0.253631000	-1.175831000	2.679220000
H	-1.291846000	2.114500000	2.249564000
H	0.043998000	2.274694000	1.095077000
C	-1.245380000	4.016999000	1.199429000
C	-3.577582000	3.643590000	0.379596000
H	-3.642958000	1.768482000	1.447751000
H	-4.013654000	1.622478000	-0.275595000
C	-1.698976000	3.860915000	-1.260153000
H	-2.091832000	1.868212000	-1.996636000
H	-0.431106000	2.100325000	-1.437190000
H	-4.284187000	-0.949032000	-0.607212000
H	-3.720919000	0.171988000	-1.852533000
C	-4.111871000	-1.832652000	-2.572346000
C	-1.783525000	-1.993693000	-3.472809000
H	-1.308317000	-0.001252000	-2.756803000
H	-0.246217000	-1.258490000	-2.129622000
C	-2.552387000	-3.593781000	-1.710931000
H	-2.669260000	-2.769037000	0.290280000
H	-1.023483000	-2.864067000	-0.347498000
H	-5.030924000	-1.223524000	3.480469000
C	-3.085491000	-1.648336000	4.308007000
H	-1.045732000	-1.978163000	4.854314000
C	0.784597000	-2.545415000	2.794734000
C	0.921821000	-0.273993000	3.635273000
H	-0.626526000	4.486705000	1.973736000
C	-2.726044000	4.328275000	1.456180000
C	-0.838506000	4.539559000	-0.185522000
H	-4.639437000	3.843397000	0.563552000
C	-3.181694000	4.166608000	-1.007505000
H	-1.408401000	4.222872000	-2.253513000
H	-5.154603000	-1.592261000	-2.809564000
C	-4.014696000	-3.273587000	-2.053354000
C	-3.241910000	-1.669762000	-3.825299000
C	-1.681088000	-3.437630000	-2.964032000
H	-1.150293000	-1.863414000	-4.358883000
H	-2.476635000	-4.618201000	-1.327525000
H	-3.467330000	-2.017274000	5.254758000
H	1.853657000	-2.539275000	2.556275000
H	0.267224000	-3.200233000	2.090920000
H	0.673124000	-2.959468000	3.804903000
H	0.786753000	-0.592974000	4.676858000
H	0.525990000	0.737092000	3.524898000
H	1.998069000	-0.256720000	3.430069000
H	-2.889700000	5.411980000	1.435215000
H	-3.021660000	3.982721000	2.455037000
H	0.226318000	4.341711000	-0.370607000
H	-0.969955000	5.626974000	-0.229406000
H	-3.803413000	3.702531000	-1.783800000
H	-3.359312000	5.246764000	-1.065902000
H	-4.382950000	-3.972075000	-2.813617000
H	-4.650936000	-3.405749000	-1.168941000
H	-3.322671000	-0.647413000	-4.216556000
H	-3.594396000	-2.339611000	-4.618127000
H	-0.636002000	-3.690108000	-2.735936000
H	-2.011006000	-4.136586000	-3.741319000
C	4.478649000	0.057564000	0.276598000
H	4.336233000	1.058915000	0.678816000
C	3.216973000	-0.773045000	0.357957000
C	5.404210000	0.043367000	-0.920047000
H	5.391649000	-0.930067000	-1.418667000
H	5.088413000	0.797297000	-1.644469000
C	6.894234000	-0.520507000	0.884649000
H	7.513451000	-0.129557000	1.691561000
H	7.148611000	-1.558753000	0.661699000
C	6.793671000	0.350287000	-0.350158000
H	6.885441000	1.407629000	-0.084124000
H	7.592145000	0.113839000	-1.057178000
C	3.349251000	-2.201161000	-0.155287000
H	4.154954000	-2.747383000	0.355883000
H	2.436014000	-2.775309000	0.016358000
H	3.572010000	-2.241546000	-1.222642000
H	2.949290000	-0.777837000	1.430718000
C	2.319474000	0.404724000	-1.055538000
C	2.557297000	1.787198000	-0.921433000
C	2.386117000	-0.187979000	-2.324601000
C	2.824655000	2.559333000	-2.050316000
H	2.504520000	2.269323000	0.051226000
C	2.660087000	0.597703000	-3.443585000
H	2.187656000	-1.245863000	-2.457918000
C	2.888877000	1.967861000	-3.313046000
H	2.984232000	3.627837000	-1.937997000

H	2.689851000	0.131162000	-4.423958000
H	3.110834000	2.570433000	-4.187978000
H	5.215279000	-1.390233000	1.725783000
O	5.486370000	-0.512682000	1.409000000

TS2$_{OH5E}$

Sum of electronic and zero-point Energies = -2163.721715
Sum of electronic and thermal Free Energies = -2163.796400
Esolv = -2165.18304156

Au	1.017353000	-0.470960000	0.483833000
P	-1.188772000	0.456226000	0.197088000
C	-1.695359000	1.180666000	1.803804000
C	-1.078634000	1.903717000	-1.034086000
C	-2.429430000	-0.926651000	-0.233311000
C	-2.964467000	1.778050000	1.926384000
C	-0.858349000	1.163033000	2.936886000
C	0.093598000	2.798153000	-0.563364000
C	-2.340780000	2.794143000	-1.116077000
C	-0.758275000	1.351942000	-2.440607000
C	-3.801189000	-0.422648000	-0.734874000
C	-1.818589000	-1.848120000	-1.316213000
C	-2.632982000	-1.759208000	1.055240000
H	-3.635308000	1.793954000	1.077322000
C	-3.392711000	2.355063000	3.115309000
C	-1.300649000	1.748726000	4.128815000
N	0.437390000	0.499542000	2.925897000
H	-0.120044000	3.201335000	0.435040000
H	1.009596000	2.192869000	-0.480982000
C	0.322811000	3.951496000	-1.557883000
C	-2.111761000	3.939853000	-2.122888000
H	-2.557241000	3.229926000	-0.135816000
H	-3.214064000	2.209629000	-1.421588000
C	-0.524153000	2.511297000	-3.425938000
H	-1.589441000	0.738232000	-2.804481000
H	0.127545000	0.702097000	-2.400427000
H	-4.274083000	0.231779000	0.004185000
H	-3.681068000	0.151253000	-1.660519000
C	-4.733400000	-1.621830000	-1.003138000
C	-2.759289000	-3.036823000	-1.592335000
H	-1.649549000	-1.295286000	-2.246040000
H	-0.845321000	-2.225542000	-0.983207000
C	-3.568949000	-2.949464000	0.772792000
H	-3.059745000	-1.137301000	1.850174000
H	-1.662087000	-2.132275000	1.414605000
H	-4.378077000	2.806271000	3.173128000
C	-2.551205000	2.346610000	4.223319000
H	-0.664814000	1.728077000	5.008536000
C	0.375618000	-0.714382000	3.764497000
C	1.508790000	1.394869000	3.382395000
H	1.160643000	4.562431000	-1.198228000
C	-0.945031000	4.812561000	-1.643620000
C	0.648792000	3.375374000	-2.943299000
H	-3.028082000	4.539338000	-2.168088000
C	-1.796299000	3.366171000	-3.510081000
H	-0.294862000	2.088453000	-4.411320000
H	-5.697983000	-1.231483000	-1.347689000
C	-4.928246000	-2.422690000	0.291296000
C	-4.116877000	-2.518359000	-2.084063000
C	-2.949741000	-3.849049000	-0.304685000
H	-2.298268000	-3.664351000	-2.364359000
H	-3.697771000	-3.514139000	1.703772000
H	-2.868321000	2.792394000	5.160906000
H	1.325533000	-1.251685000	3.710541000
H	-0.419028000	-1.368159000	3.400487000
H	0.174752000	-0.480780000	4.818826000
H	1.389224000	1.722544000	4.423562000
H	1.543499000	2.282368000	2.745582000
H	2.465087000	0.864499000	3.326231000
H	-0.785384000	5.646398000	-2.337067000
H	-1.173368000	5.253403000	-0.665023000
H	1.570116000	2.775564000	-2.903363000
H	0.835796000	4.188457000	-3.654305000
H	-2.636037000	2.761706000	-3.875426000
H	-1.657014000	4.179845000	-4.231310000
H	-5.613376000	-3.259934000	0.114948000
H	-5.389660000	-1.794883000	1.064310000
H	-3.996787000	-1.959721000	-3.021399000
H	-4.785361000	-3.359414000	-2.301765000
H	-1.986546000	-4.248943000	0.040000000
H	-3.600353000	-4.710682000	-0.494526000
C	3.359788000	-1.086284000	0.466746000
C	1.917726000	-2.021664000	-0.663234000
C	2.059381000	-1.854784000	-2.047436000
C	1.635396000	-3.282119000	-0.125870000
C	1.862828000	-2.946015000	-2.888807000
H	2.296366000	-0.889905000	-2.481972000
C	1.433864000	-4.364057000	-0.987184000
H	1.536915000	-3.433300000	0.943927000
C	1.553229000	-4.202868000	-2.364233000
H	1.957549000	-2.810817000	-3.962254000
H	1.193283000	-5.335664000	-0.566274000
H	1.417177000	-5.051111000	-3.027204000
C	3.611637000	-1.735481000	1.816888000
H	3.134970000	-1.201256000	2.642023000
H	3.266518000	-2.769442000	1.835935000
H	4.689534000	-1.776351000	2.011451000
H	3.957656000	-1.591387000	-0.289726000
C	3.735660000	0.372255000	0.420649000
C	3.764113000	1.129150000	-0.896702000
H	3.366960000	0.951197000	1.268709000
C	6.106394000	0.985693000	-0.399429000
C	5.128337000	0.848477000	-1.542996000
H	3.650384000	2.200609000	-0.707755000
H	2.937373000	0.821801000	-1.537676000
H	6.311351000	2.024583000	-0.130797000
H	7.030322000	0.415144000	-0.498087000
H	5.355604000	1.562835000	-2.337674000
H	5.172182000	-0.156418000	-1.973010000
O	5.352544000	0.362533000	0.726723000
H	5.592176000	0.727870000	1.594928000

TS2$_{OH6}$

Sum of electronic and zero-point Energies = -2124.454347
Sum of electronic and thermal Free Energies = -2124.525671
Esolv = -2125.8812386

C	-2.291890000	1.178414000	-0.966690000
C	-2.273593000	2.568197000	-0.732891000
H	-2.163822000	2.959660000	0.275346000
Au	-1.118099000	0.059395000	0.400292000
C	-2.434893000	0.700242000	-2.279348000
H	-2.434951000	-0.364788000	-2.487644000
C	-2.511419000	1.601987000	-3.339895000
H	-2.595126000	1.221172000	-4.353647000
C	-2.475090000	2.977881000	-3.110096000
H	-2.546963000	3.672021000	-3.941110000
C	-2.348803000	3.454878000	-1.806051000
H	-2.311179000	4.523415000	-1.615413000
P	1.253854000	-0.252955000	0.237298000

C	1.701664000	-1.136596000	1.781122000
C	1.609844000	-1.437418000	-1.203838000
C	2.185284000	1.404625000	0.228061000
C	3.045763000	-1.461035000	2.043519000
C	0.734960000	-1.494001000	2.740477000
C	0.585657000	-2.593557000	-1.093700000
C	3.026203000	-2.056979000	-1.191899000
C	1.389010000	-0.697313000	-2.541765000
C	3.678650000	1.298639000	-0.153406000
C	1.486612000	2.365419000	-0.763148000
C	2.064673000	2.002519000	1.650359000
H	3.812874000	-1.192217000	1.328333000
C	3.425812000	-2.122834000	3.204670000
C	1.126789000	-2.163123000	3.904666000
N	-0.677165000	-1.169749000	2.567147000
H	0.725505000	-3.129511000	-0.145523000
H	-0.438295000	-2.187215000	-1.095891000
C	0.749186000	-3.567332000	-2.275570000
C	3.186302000	-3.019515000	-2.386475000
H	3.183945000	-2.618544000	-0.265302000
H	3.793973000	-1.278230000	-1.244901000
C	1.544453000	-1.679362000	-3.717993000
H	2.122592000	0.108815000	-2.653384000
H	0.392619000	-0.236101000	-2.563064000
H	4.211954000	0.630562000	0.530882000
H	3.786850000	0.892502000	-1.165233000
C	4.331223000	2.694823000	-0.095483000
C	2.151124000	3.753927000	-0.712997000
H	1.534568000	1.973685000	-1.784361000
H	0.424890000	2.456568000	-0.505413000
C	2.724573000	3.393540000	1.695098000
H	2.544116000	1.345810000	2.384726000
H	1.003482000	2.086794000	1.931019000
H	4.471631000	-2.357290000	3.375496000
C	2.459158000	-2.480237000	4.139938000
H	0.383548000	-2.440475000	4.646134000
C	-1.129893000	-0.241195000	3.620394000
C	-1.506324000	-2.387084000	2.553940000
H	0.010220000	-4.371048000	-2.168989000
C	2.165885000	-4.157968000	-2.261368000
C	0.515780000	-2.811509000	-3.591097000
H	4.202975000	-3.428017000	-2.359092000
C	2.963590000	-2.264163000	-3.703186000
H	1.379031000	-1.128553000	-4.651483000
H	5.388623000	2.587688000	-0.363473000
C	4.209092000	3.261926000	1.325323000
C	3.633139000	3.629437000	-1.091804000
C	2.024677000	4.331447000	0.702907000
H	1.638089000	4.406168000	-1.430036000
H	2.629149000	3.787381000	2.713919000
H	2.738674000	-2.999680000	5.051135000
H	-2.168681000	0.049643000	3.427725000
H	-0.505238000	0.653924000	3.613518000
H	-1.089184000	-0.689661000	4.621254000
H	-1.485494000	-2.923937000	3.510900000
H	-1.157680000	-3.060662000	1.768805000
H	-2.546519000	-2.110339000	2.352074000
H	2.284320000	-4.865222000	-3.090417000
H	2.334851000	-4.723462000	-1.336072000
H	-0.505110000	-2.404945000	-3.622512000
H	0.605857000	-3.498972000	-4.440081000
H	3.705830000	-1.463736000	-3.816269000
H	3.100664000	-2.942746000	-4.553105000
H	4.697271000	4.241714000	1.381728000
H	4.724476000	2.610976000	2.043110000
H	3.735235000	3.243311000	-2.114198000
H	4.109420000	4.616636000	-1.078494000
H	0.966635000	4.452121000	0.974175000
H	2.476525000	5.329265000	0.745900000
C	-4.519360000	1.202757000	0.187344000
H	-4.828789000	1.379793000	-0.842463000
H	-4.343604000	2.144205000	0.703360000
C	-3.447176000	0.148204000	0.340912000
C	-3.924749000	-1.183248000	-0.243809000
H	-3.261614000	0.041811000	1.424908000
C	-6.325220000	-0.604403000	0.284990000
C	-5.239285000	-1.644910000	0.409495000
H	-4.083308000	-1.073270000	-1.322199000
H	-3.170057000	-1.963306000	-0.113780000
H	-7.232741000	-0.812986000	0.853645000
H	-6.588984000	-0.363629000	-0.746524000
H	-5.603865000	-2.556952000	-0.077256000
H	-5.086571000	-1.901875000	1.465355000
H	-5.780817000	0.692797000	1.793915000
O	-5.834762000	0.709851000	0.821644000

$TS2_{OH6Z}$
Sum of electronic and zero-point Energies = -2163.730212
Sum of electronic and thermal Free Energies = -2163.802696
Esolv = -2165.1940651

C	2.207057000000	-1.023150000000	-0.980541000000
C	2.232639000000	-2.389488000000	-0.642052000000
H	2.144591000000	-2.709222000000	0.392763000000
Au	1.040581000000	0.128393000000	0.344211000000
C	2.335378000000	-0.633805000000	-2.320755000000
H	2.318898000000	0.414311000000	-2.601036000000
C	2.443792000000	-1.606210000000	-3.314326000000
H	2.523515000000	-1.297698000000	-4.352679000000
C	2.450244000000	-2.962354000000	-2.985042000000
H	2.544933000000	-3.712405000000	-3.763656000000
C	2.337038000000	-3.348793000000	-1.648248000000
H	2.334973000000	-4.401813000000	-1.382067000000
P	-1.358061000000	0.211460000000	0.242547000000
C	-1.848917000000	1.137841000000	1.746310000000
C	-1.929808000000	1.224488000000	-1.263097000000
C	-2.066852000000	-1.541437000000	0.409267000000
C	-3.205646000000	1.417210000000	1.994437000000
C	-0.896598000000	1.603144000000	2.672245000000
C	-1.597253000000	2.707070000000	-0.970232000000
C	-3.435332000000	1.097147000000	-1.586180000000
C	-1.115739000000	0.783735000000	-2.502232000000
C	-3.578164000000	-1.600940000000	0.728570000000
C	-1.788720000000	-2.325507000000	-0.892752000000
C	-1.308419000000	-2.216111000000	1.578549000000
H	-3.959988000000	1.084507000000	1.292565000000
C	-3.613328000000	2.116754000000	3.123655000000
C	-1.316768000000	2.299061000000	3.810509000000
N	0.533776000000	1.390067000000	2.474492000000
H	-2.151149000000	3.058942000000	-0.093099000000
H	-0.525014000000	2.810484000000	-0.741468000000
C	-1.954381000000	3.579575000000	-2.188209000000
C	-3.784375000000	1.981962000000	-2.800053000000
H	-4.046260000000	1.408937000000	-0.732462000000
H	-3.690116000000	0.056272000000	-1.813686000000
C	-1.479362000000	1.657685000000	-3.716724000000
H	-1.299315000000	-0.269902000000	-2.736983000000
H	-0.044022000000	0.880229000000	-2.287295000000
H	-3.784816000000	-1.082363000000	1.670478000000
H	-4.164516000000	-1.113351000000	-0.056908000000
C	-4.025318000000	-3.070967000000	0.866860000000

C	-2.235768000000	-3.790295000000	-0.727687000000
H	-2.339088000000	-1.877973000000	-1.728054000000
H	-0.720799000000	-2.284166000000	-1.143647000000
C	-1.759624000000	-3.681181000000	1.730332000000
H	-1.497895000000	-1.669813000000	2.512081000000
H	-0.224671000000	-2.185780000000	1.391186000000
H	-4.667974000000	2.315982000000	3.284641000000
C	-2.663062000000	2.554998000000	4.041359000000
H	-0.585244000000	2.652771000000	4.530863000000
C	1.094713000000	0.555650000000	3.552866000000
C	1.247234000000	2.676577000000	2.384445000000
H	-1.714028000000	4.622034000000	-1.947807000000
C	-3.455752000000	3.446869000000	-2.482224000000
C	-1.145418000000	3.123373000000	-3.409386000000
H	-4.856632000000	1.876559000000	-3.001196000000
C	-2.977672000000	1.519297000000	-4.020114000000
H	-0.893487000000	1.313103000000	-4.577544000000
H	-5.101086000000	-3.079671000000	1.076509000000
C	-3.264737000000	-3.726243000000	2.026646000000
C	-3.742013000000	-3.830064000000	-0.436007000000
C	-1.465020000000	-4.440907000000	0.429248000000
H	-2.023481000000	-4.322566000000	-1.662448000000
H	-1.202964000000	-4.131493000000	2.561305000000
H	-2.965313000000	3.097990000000	4.931314000000
H	2.152298000000	0.356341000000	3.345594000000
H	0.559651000000	-0.394471000000	3.598492000000
H	1.033853000000	1.041762000000	4.534928000000
H	1.209138000000	3.247757000000	3.320638000000
H	0.810803000000	3.282859000000	1.588401000000
H	2.300213000000	2.486625000000	2.152475000000
H	-3.729144000000	4.085600000000	-3.330015000000
H	-4.044372000000	3.793722000000	-1.623330000000
H	-0.069059000000	3.239003000000	-3.218969000000
H	-1.381280000000	3.754304000000	-4.274158000000
H	-3.222848000000	0.478630000000	-4.268330000000
H	-3.240867000000	2.122848000000	-4.896450000000
H	-3.590247000000	-4.765311000000	2.153065000000
H	-3.484514000000	-3.208788000000	2.969247000000
H	-4.302617000000	-3.385631000000	-1.268325000000
H	-4.081642000000	-4.868490000000	-0.346657000000
H	-0.386824000000	-4.433941000000	0.218630000000
H	-1.759573000000	-5.491574000000	0.533970000000
C	3.362013000000	0.177159000000	0.251703000000
H	3.163071000000	0.352386000000	1.326046000000
C	4.530199000000	-0.804110000000	0.238641000000
C	3.744798000000	1.506981000000	-0.401431000000
H	4.021318000000	1.353449000000	-1.448829000000
H	2.896603000000	2.197570000000	-0.406877000000
H	5.425900000000	0.016814000000	2.000637000000
C	6.103454000000	1.255371000000	0.517008000000
H	6.856063000000	1.615616000000	1.220619000000
H	6.591596000000	0.999443000000	-0.422633000000
C	4.909121000000	2.169315000000	0.359122000000
H	4.572946000000	2.508015000000	1.347209000000
H	5.250566000000	3.066352000000	-0.170254000000
O	5.679266000000	-0.071410000000	1.064938000000
C	5.232516000000	-1.212987000000	-1.033354000000
H	6.216225000000	-1.622741000000	-0.789608000000
H	4.660720000000	-2.006945000000	-1.515820000000
H	5.343502000000	-0.402329000000	-1.754091000000
H	4.356968000000	-1.667153000000	0.884116000000

$TS2_{OH6E}$
Sum of electronic and zero-point Energies = -2163.734218
Sum of electronic and thermal Free Energies = -2163.808128
Esolv = -2165.1965859

C	2.256089000000	1.142832000000	-1.055744000000
C	2.257277000000	0.762683000000	-2.407311000000
H	2.184351000000	-0.276833000000	-2.705524000000
Au	1.067292000000	0.011247000000	0.312837000000
C	2.309251000000	2.508099000000	-0.727999000000
H	2.309298000000	2.834135000000	0.306890000000
C	2.332042000000	3.469520000000	-1.736712000000
H	2.353756000000	4.520571000000	-1.464387000000
C	2.335655000000	3.089917000000	-3.077858000000
H	2.372153000000	3.841372000000	-3.859876000000
C	2.293617000000	1.736366000000	-3.405611000000
H	2.281530000000	1.425527000000	-4.446195000000
P	-1.320831000000	-0.201261000000	0.278960000000
C	-1.761153000000	-0.903580000000	1.917116000000
C	-2.152698000000	1.504151000000	0.136452000000
C	-1.814242000000	-1.495933000000	-1.020674000000
C	-3.113877000000	-1.090758000000	2.258157000000
C	-0.784674000000	-1.255138000000	2.869447000000
C	-1.910964000000	2.233743000000	1.479851000000
C	-3.669517000000	1.463161000000	-0.154680000000
C	-1.458597000000	2.310315000000	-0.987570000000
C	-3.268979000000	-2.006029000000	-0.902029000000
C	-1.593917000000	-0.914962000000	-2.434788000000
C	-0.871986000000	-2.707248000000	-0.820310000000
H	-3.888268000000	-0.827338000000	1.549140000000
C	-3.494653000000	-1.608784000000	3.489964000000
C	-1.178319000000	-1.779070000000	4.105469000000
N	0.638334000000	-1.080847000000	2.611082000000
H	-2.385362000000	1.688451000000	2.303330000000
H	-0.831848000000	2.274203000000	1.694272000000
C	-2.479816000000	3.663567000000	1.413764000000
C	-4.229773000000	2.899310000000	-0.207631000000
H	-4.204957000000	0.905982000000	0.620577000000
H	-3.860558000000	0.966780000000	-1.112665000000
C	-2.029752000000	3.739793000000	-1.045716000000
H	-1.599255000000	1.823596000000	-1.958012000000
H	-0.379303000000	2.354732000000	-0.804258000000
H	-3.431515000000	-2.454795000000	0.083371000000
H	-3.982139000000	-1.183144000000	-1.014884000000
C	-3.539175000000	-3.072438000000	-1.983595000000
C	-1.859912000000	-1.997676000000	-3.496979000000
H	-2.273780000000	-0.073523000000	-2.607630000000
H	-0.569889000000	-0.529285000000	-2.533696000000
C	-1.143054000000	-3.781033000000	-1.889927000000
H	-1.017977000000	-3.132761000000	0.181310000000
H	0.176762000000	-2.377622000000	-0.886667000000
H	-4.547511000000	-1.738324000000	3.719349000000
C	-2.519941000000	-1.958122000000	4.419778000000
H	-0.426666000000	-2.051296000000	4.840443000000
C	1.346205000000	-2.368585000000	2.647817000000
C	1.236656000000	-0.127166000000	3.563092000000
H	-2.299900000000	4.150724000000	2.379447000000
C	-3.988819000000	3.596112000000	1.138351000000
C	-1.782619000000	4.446422000000	0.293558000000
H	-5.305879000000	2.837126000000	-0.406563000000
C	-3.536250000000	3.680797000000	-1.330643000000
H	-1.519391000000	4.279347000000	-1.852581000000
H	-4.579708000000	-3.401585000000	-1.883108000000
C	-2.595660000000	-4.263226000000	-1.772429000000
C	-3.314521000000	-2.473860000000	-3.378329000000
C	-0.907117000000	-3.181575000000	-3.283379000000

H	-1.692363000000	-1.558697000000	-4.487630000000
H	-0.458352000000	-4.621719000000	-1.722236000000
H	-2.799910000000	-2.365920000000	5.386000000000
H	2.395551000000	-2.212228000000	2.369506000000
H	0.889874000000	-3.060893000000	1.937801000000
H	1.338515000000	-2.830554000000	3.643818000000
H	1.215363000000	-0.493124000000	4.598011000000
H	0.697754000000	0.821269000000	3.519139000000
H	2.283729000000	0.045517000000	3.291696000000
H	-4.411440000000	4.607299000000	1.118252000000
H	-4.499943000000	3.056647000000	1.946021000000
H	-0.704815000000	4.519575000000	0.494072000000
H	-2.166955000000	5.472390000000	0.256121000000
H	-3.723454000000	3.203042000000	-2.300980000000
H	-3.948373000000	4.694629000000	-1.393456000000
H	-2.793637000000	-5.040188000000	-2.519874000000
H	-2.767995000000	-4.718663000000	-0.788918000000
H	-4.003150000000	-1.637345000000	-3.552300000000
H	-3.529652000000	-3.223910000000	-4.148219000000
H	0.137372000000	-2.853258000000	-3.387843000000
H	-1.073663000000	-3.943524000000	-4.053510000000
C	3.401274000000	0.028710000000	0.222275000000
H	3.107768000000	-0.650698000000	1.048146000000
C	4.124887000000	-0.843429000000	-0.786084000000
H	4.475869000000	-0.278110000000	-1.651705000000
C	4.288961000000	1.115926000000	0.836485000000
H	4.566801000000	1.852687000000	0.076225000000
H	3.729962000000	1.653696000000	1.606719000000
C	3.486231000000	-2.157831000000	-1.160774000000
H	3.279678000000	-2.768225000000	-0.273611000000
H	2.524132000000	-1.980260000000	-1.644080000000
H	4.115041000000	-2.721557000000	-1.853865000000
H	5.357629000000	-2.015723000000	0.451423000000
C	6.357396000000	-0.221457000000	0.423007000000
H	7.228671000000	-0.759174000000	0.800869000000
H	6.648167000000	0.385568000000	-0.436347000000
C	5.565695000000	0.535281000000	1.457543000000
H	5.345474000000	-0.112362000000	2.315904000000
H	6.198994000000	1.344790000000	1.837854000000
O	5.492994000000	-1.285439000000	-0.179161000000

6_{OH5}

Sum of electronic and zero-point Energies = -2124,550272
Sum of electronic and thermal Free Energies = -2124.626303
Esolv = -2125.9528997

C	-4.038315000	0.338881000	0.206060000
C	-3.858753000	0.579365000	1.588196000
H	-4.464228000	0.043606000	2.315255000
Au	-0.272971000	0.940138000	0.618592000
C	-3.243416000	1.007906000	-0.711634000
H	-3.366756000	0.836713000	-1.777417000
C	-2.277033000	1.944866000	-0.280226000
H	-1.790744000	2.576895000	-1.017259000
C	-2.127299000	2.216133000	1.103586000
H	-1.588182000	3.102979000	1.431537000
C	-2.934155000	1.504586000	2.030452000
H	-2.846528000	1.718371000	3.090935000
P	1.664326000	-0.225700000	0.005706000
C	2.798285000	-0.116881000	1.445881000
C	1.258301000	-2.061632000	-0.241831000
C	2.496888000	0.661050000	-1.459565000
C	4.095392000	-0.653611000	1.340120000
C	2.431687000	0.505176000	2.659001000
C	0.426211000	-2.497005000	0.989447000
C	2.484531000	-2.997360000	-0.340757000
C	0.392317000	-2.222978000	-1.510601000
C	3.592987000	-0.156615000	-2.178223000
C	1.413824000	1.047273000	-2.497244000
C	3.116700000	1.965479000	-0.902749000
H	4.406159000	-1.127254000	0.417217000
C	5.003850000	-0.592860000	2.389245000
C	3.355757000	0.552880000	3.709612000
N	1.124189000	1.103205000	2.860173000
H	1.026548000	-2.389118000	1.901941000
H	-0.453070000	-1.844160000	1.096228000
C	-0.031160000	-3.958583000	0.836367000
C	2.017910000	-4.457783000	-0.506638000
H	3.089093000	-2.921194000	0.568878000
H	3.121647000	-2.719889000	-1.186694000
C	-0.068534000	-3.684686000	-1.653941000
H	0.966626000	-1.941162000	-2.400448000
H	-0.477645000	-1.553117000	-1.457684000
H	4.385229000	-0.447302000	-1.480678000
H	3.169679000	-1.075768000	-2.599272000
C	4.212846000	0.682023000	-3.314270000
C	2.036732000	1.870813000	-3.639636000
H	0.933266000	0.152518000	-2.908060000
H	0.628347000	1.635427000	-2.003264000
C	3.732878000	2.789841000	-2.047901000
H	3.889251000	1.735444000	-0.161161000
H	2.342914000	2.557785000	-0.391781000
H	5.996481000	-1.015527000	2.270772000
C	4.629306000	0.012000000	3.585340000
H	3.074330000	1.025581000	4.645864000
C	1.226638000	2.540354000	3.158353000
C	0.368338000	0.399249000	3.905917000
H	-0.617574000	-4.234167000	1.721655000
C	1.195748000	-4.873198000	0.720089000
C	-0.895295000	-4.090316000	-0.425830000
H	2.907347000	-5.092958000	-0.589425000
C	1.162489000	-4.594095000	-1.773320000
H	-0.680313000	-3.768800000	-2.560764000
H	4.991553000	0.080169000	-3.797142000
C	4.829285000	1.962352000	-2.734118000
C	3.126655000	1.043698000	-4.335147000
C	2.646914000	3.157098000	-3.067483000
H	1.245031000	2.120017000	-4.357025000
H	4.167812000	3.701232000	-1.620959000
H	5.324405000	0.066137000	4.417484000
H	0.220534000	2.964603000	3.226130000
H	1.769619000	3.044790000	2.356220000
H	1.739274000	2.745602000	4.108408000
H	0.832537000	0.488224000	4.898429000
H	0.289882000	-0.660037000	3.653520000
H	-0.639113000	0.820351000	3.965428000
H	0.878298000	-5.918584000	0.626462000
H	1.806418000	-4.807603000	1.629414000
H	-1.788000000	-3.453397000	-0.341998000
H	-1.248888000	-5.122862000	-0.533943000
H	1.747847000	-4.324895000	-2.661800000
H	0.850113000	-5.636827000	-1.905449000
H	5.297445000	2.548726000	-3.533418000
H	5.623137000	1.713855000	-2.018179000
H	2.695644000	0.133653000	-4.772751000
H	3.562614000	1.616163000	-5.162256000
H	1.869392000	3.770555000	-2.591961000
H	3.076732000	3.762302000	-3.874310000
C	-5.117247000	-0.624576000	-0.255450000
H	-5.071290000	-1.534194000	0.357457000
H	-4.945532000	-0.906334000	-1.300742000

C	-6.490254000	0.013557000	-0.111579000
H	-6.711080000	0.267927000	0.927145000
C	-8.417073000	0.985133000	-1.195624000
C	-8.646120000	-0.505999000	-1.311096000
H	-7.212848000	-1.909256000	-0.698240000
H	-8.924376000	1.385664000	-0.313261000
H	-8.807998000	1.504939000	-2.073351000
H	-8.449200000	-0.904891000	-2.308227000
H	-9.588480000	-0.879365000	-0.911671000
C	-6.893838000	1.116782000	-1.069670000
H	-6.405915000	0.979933000	-2.041143000
H	-6.598158000	2.091994000	-0.676692000
O	-7.575335000	-1.057653000	-0.399043000

6_{OH5Z}

Sum of electronic and zero-point Energies = -2163.823585
Sum of electronic and thermal Free Energies = -2163.903045
Esolv = -2165.2610859

Au	-0.189644000	0.424206000	1.021957000
P	1.791335000	-0.110355000	-0.087426000
C	3.096408000	-0.296014000	1.192905000
C	1.599992000	-1.802511000	-0.925533000
C	2.280794000	1.343037000	-1.216600000
C	4.419809000	-0.537667000	0.776785000
C	2.841264000	-0.199516000	2.578841000
C	1.016939000	-2.758922000	0.143917000
C	2.914953000	-2.422523000	-1.451007000
C	0.593648000	-1.684957000	-2.090960000
C	3.367544000	1.019574000	-2.266235000
C	1.019577000	1.850144000	-1.959261000
C	2.786297000	2.478350000	-0.293955000
H	4.646485000	-0.606683000	-0.279792000
C	5.460258000	-0.691716000	1.684170000
C	3.899890000	-0.364700000	3.480374000
N	1.521028000	0.078735000	3.106622000
H	1.718578000	-2.853609000	0.982543000
H	0.080230000	-2.344398000	0.545275000
C	0.745056000	-4.144420000	-0.469121000
C	2.631325000	-3.804152000	-2.074381000
H	3.625988000	-2.547129000	-0.628107000
H	3.382646000	-1.773583000	-2.198826000
C	0.318584000	-3.074658000	-2.693932000
H	0.993096000	-1.029374000	-2.873307000
H	-0.342661000	-1.233786000	-1.732342000
H	4.283565000	0.662530000	-1.784778000
H	3.021248000	0.230930000	-2.943889000
C	3.698084000	2.283381000	-3.085724000
C	1.354575000	3.103086000	-2.788986000
H	0.618047000	1.070459000	-2.615879000
H	0.235129000	2.087409000	-1.227503000
C	3.114335000	3.731246000	-1.126453000
H	3.680036000	2.156937000	0.251686000
H	2.018226000	2.719760000	0.455793000
H	6.468028000	-0.875234000	1.325363000
C	5.197061000	-0.609983000	3.048183000
H	3.704194000	-0.295740000	4.546381000
C	1.497593000	1.342401000	3.855436000
C	1.011451000	-1.030852000	3.921744000
H	0.332169000	-4.794869000	0.311624000
C	2.055662000	-4.738582000	-1.002691000
C	-0.263421000	-4.001654000	-1.617875000
H	3.578025000	-4.209151000	-2.450199000
C	1.631790000	-3.662459000	-3.229697000
H	-0.398423000	-2.960532000	-3.516583000
H	4.476717000	2.024594000	-3.812699000
C	4.206717000	3.388020000	-2.149202000
C	2.439554000	2.762796000	-3.819791000
C	1.854726000	4.214697000	-1.857002000
H	0.442828000	3.430259000	-3.304473000
H	3.474577000	4.512334000	-0.446557000
H	5.996295000	-0.732080000	3.772705000
H	0.469646000	1.563008000	4.160340000
H	1.856813000	2.154406000	3.219395000
H	2.113100000	1.316155000	4.766178000
H	1.599655000	-1.199816000	4.835545000
H	1.013135000	-1.951375000	3.334291000
H	-0.017025000	-0.810183000	4.224395000
H	1.872554000	-5.733168000	-1.426417000
H	2.774861000	-4.870139000	-0.184419000
H	-1.213445000	-3.597340000	-1.239693000
H	-0.485634000	-4.985472000	-2.048252000
H	2.043931000	-3.016703000	-4.015754000
H	1.447200000	-4.640653000	-3.689381000
H	4.471627000	4.279498000	-2.729782000
H	5.120295000	3.062876000	-1.635310000
H	2.081537000	1.987742000	-4.510155000
H	2.671207000	3.645271000	-4.427713000
H	1.074744000	4.485766000	-1.132171000
H	2.077834000	5.119765000	-2.434236000
C	-6.326634000	0.653081000	-0.425417000
H	-6.210551000	1.728757000	-0.276441000
C	-5.011066000	0.042029000	-0.922764000
C	-7.114519000	0.020784000	0.704524000
H	-7.025146000	-1.069874000	0.691861000
H	-6.751186000	0.381185000	1.669315000
C	-8.735034000	0.268706000	-1.051743000
H	-9.422022000	0.956561000	-1.543232000
H	-8.927802000	-0.764277000	-1.347737000
C	-8.563398000	0.451225000	0.441406000
H	-8.722158000	1.496241000	0.722742000
H	-9.285439000	-0.157819000	0.990187000
C	-5.113221000	-1.423788000	-1.359979000
H	-5.779897000	-1.563585000	-2.221030000
H	-4.131820000	-1.778106000	-1.682971000
H	-5.462727000	-2.089764000	-0.567777000
H	-4.710283000	0.636596000	-1.795879000
C	-3.957854000	0.303233000	0.153694000
C	-3.217042000	1.481248000	0.108812000
C	-3.736166000	-0.602166000	1.210606000
C	-2.296011000	1.795332000	1.123441000
H	-3.359476000	2.187403000	-0.705344000
C	-2.833804000	-0.309490000	2.221042000
H	-4.282285000	-1.539024000	1.256618000
C	-2.110195000	0.908151000	2.209192000
H	-1.824401000	2.773740000	1.130778000
H	-2.708679000	-0.999248000	3.049879000
H	-1.596868000	1.240673000	3.108931000
H	-7.084146000	0.038427000	-2.306527000
O	-7.363833000	0.623324000	-1.580848000

6_{OH5E}

Sum of electronic and zero-point Energies = -2163.820858
Sum of electronic and thermal Free Energies = -2163.899368
Esolv = -2165.26141145

Au	0.117622000	-1.187040000	-0.628000000
P	-1.590540000	0.225129000	0.081942000
C	-3.190652000	-0.567258000	-0.353408000
C	-1.540057000	0.342835000	1.975542000

C	-1.475358000	1.871935000	-0.870216000
C	-4.379183000	0.147985000	-0.110084000
C	-3.292733000	-1.854409000	-0.926875000
C	-1.476616000	-1.112442000	2.501785000
C	-2.766270000	1.027873000	2.621216000
C	-0.256724000	1.084394000	2.408854000
C	-2.280328000	3.041804000	-0.260468000
C	0.011267000	2.298654000	-0.955585000
C	-1.981266000	1.599575000	-2.307498000
H	-4.330990000	1.139508000	0.322543000
C	-5.629391000	-0.378266000	-0.409311000
C	-4.561364000	-2.372049000	-1.216462000
N	-2.130034000	-2.658367000	-1.235487000
H	-2.381495000	-1.656305000	2.202584000
H	-0.617581000	-1.634966000	2.055491000
C	-1.344779000	-1.121705000	4.035338000
C	-2.620694000	1.024708000	4.156465000
H	-3.680814000	0.489611000	2.352686000
H	-2.871871000	2.058658000	2.267547000
C	-0.126899000	1.064751000	3.943052000
H	-0.285349000	2.125039000	2.064474000
H	0.620200000	0.607979000	1.946581000
H	-3.342557000	2.789450000	-0.184474000
H	-1.921765000	3.265816000	0.750736000
C	-2.131720000	4.298447000	-1.141455000
C	0.157843000	3.562301000	-1.822902000
H	0.412361000	2.490214000	0.046563000
H	0.601312000	1.478640000	-1.388039000
C	-1.827815000	2.863691000	-3.172590000
H	-3.032765000	1.294253000	-2.289871000
H	-1.411786000	0.770420000	-2.752469000
H	-6.523273000	0.203751000	-0.208667000
C	-5.721613000	-1.651486000	-0.963544000
H	-4.640894000	-3.363368000	-1.652838000
C	-2.032751000	-2.940910000	-2.673018000
C	-2.095333000	-3.896302000	-0.447959000
H	-1.299780000	-2.163918000	4.373586000
C	-2.559291000	-0.422526000	4.660881000
C	-0.059278000	-0.386207000	4.439318000
H	-3.495190000	1.529806000	4.582563000
C	-1.342237000	1.769717000	4.562320000
H	0.791173000	1.597694000	4.222123000
H	-2.721444000	5.104358000	-0.689365000
C	-2.650731000	4.003718000	-2.555486000
C	-0.655147000	4.708410000	-1.205356000
C	-0.350116000	3.270802000	-3.241173000
H	1.220054000	3.838263000	-1.858035000
H	-2.199655000	2.640137000	-4.179365000
H	-6.689987000	-2.081349000	-1.200389000
H	-1.089710000	-3.459126000	-2.876188000
H	-2.045674000	-2.004045000	-3.234687000
H	-2.846375000	-3.580060000	-3.045941000
H	-2.919537000	-4.584081000	-0.687953000
H	-2.142158000	-3.658871000	0.616792000
H	-1.156940000	-4.424409000	-0.645995000
H	-2.481162000	-0.439315000	5.754405000
H	-3.481827000	-0.956689000	4.401033000
H	0.818156000	-0.894671000	4.015021000
H	0.058685000	-0.406099000	5.529266000
H	-1.386267000	2.814859000	4.229286000
H	-1.249663000	1.789876000	5.654637000
H	-2.574769000	4.902830000	-3.178258000
H	-3.713186000	3.731314000	-2.523049000
H	-0.281732000	4.944892000	-0.200023000
H	-0.542147000	5.617693000	-1.807482000
H	0.243900000	2.470306000	-3.703209000
H	-0.232485000	4.158268000	-3.874239000
C	5.452004000	-0.678626000	-0.593590000
C	4.077637000	-1.323257000	-0.744480000
C	3.320992000	-1.080033000	-1.890538000
C	3.595666000	-2.243420000	0.206067000
C	2.143195000	-1.798879000	-2.140571000
H	3.675714000	-0.376530000	-2.638839000
C	2.423229000	-2.950849000	-0.015902000
H	4.165115000	-2.456866000	1.106850000
C	1.690737000	-2.763554000	-1.212767000
H	1.642525000	-1.687578000	-3.097948000
H	2.097100000	-3.698315000	0.700604000
H	0.942441000	-3.497888000	-1.504951000
C	6.561184000	-1.741709000	-0.635795000
H	6.504710000	-2.424489000	0.218604000
H	6.462668000	-2.344388000	-1.541165000
H	7.555013000	-1.285982000	-0.658754000
H	5.592791000	0.005157000	-1.440023000
C	5.498953000	0.162401000	0.679749000
C	4.686147000	1.452355000	0.706245000
H	5.376457000	-0.447837000	1.578288000
C	6.948339000	2.212788000	0.934216000
C	5.646420000	2.565513000	0.257399000
H	4.321316000	1.650005000	1.718087000
H	3.819176000	1.369381000	0.047489000
H	6.966455000	2.443385000	2.001832000
H	7.857107000	2.550526000	0.436220000
H	5.302632000	3.554321000	0.569020000
H	5.771865000	2.578540000	-0.828857000
O	6.944212000	0.716617000	0.798039000
H	7.502808000	0.252430000	1.444608000

6_{OH6}

Sum of electronic and zero-point Energies = -2124.542460
Sum of electronic and thermal Free Energies = -2124.616838
Esolv = -2125.9512497

C	-4.129871000	1.039543000	-0.561829000
C	-3.374263000	1.927505000	0.187833000
H	-3.731340000	2.264539000	1.157745000
Au	-0.361745000	1.037574000	0.022250000
C	-3.664774000	0.658555000	-1.844952000
H	-4.243917000	-0.029692000	-2.455168000
C	-2.487253000	1.162883000	-2.357542000
H	-2.164119000	0.886040000	-3.355860000
C	-1.716779000	2.084306000	-1.608175000
H	-0.919685000	2.634353000	-2.099813000
C	-2.160164000	2.465015000	-0.315366000
H	-1.726818000	3.333318000	0.177101000
P	1.579174000	-0.266747000	0.218937000
C	2.055896000	-0.193030000	1.991002000
C	1.183868000	-2.079303000	-0.175422000
C	2.978621000	0.532438000	-0.795623000
C	3.247021000	-0.814831000	2.410387000
C	1.282954000	0.485057000	2.958299000
C	-0.092528000	-2.435502000	0.625522000
C	2.286724000	-3.089221000	0.215743000
C	0.879625000	-2.216701000	-1.683642000
C	4.207178000	-0.373360000	-1.035233000
C	2.415327000	0.970063000	-2.170425000
C	3.424100000	1.802613000	-0.031623000
H	3.865982000	-1.330885000	1.686832000
C	3.664502000	-0.785252000	3.735139000
C	1.712254000	0.500455000	4.290541000
N	0.052479000	1.179132000	2.619949000

H	0.104354000	-2.341743000	1.701531000
H	-0.898105000	-1.728718000	0.375207000
C	-0.545080000	-3.870422000	0.299619000
C	1.830876000	-4.522293000	-0.125715000
H	2.486525000	-3.029626000	1.290652000
H	3.222233000	-2.869637000	-0.308632000
C	0.420638000	-3.651439000	-2.001522000
H	1.774510000	-1.989784000	-2.273881000
H	0.103284000	-1.496770000	-1.977834000
H	4.642591000	-0.701537000	-0.085482000
H	3.917261000	-1.270541000	-1.593772000
C	5.277729000	0.396385000	-1.834827000
C	3.490421000	1.724972000	-2.973429000
H	2.069047000	0.102618000	-2.742873000
H	1.544444000	1.621784000	-2.017091000
C	4.494730000	2.558351000	-0.840175000
H	3.828301000	1.535833000	0.950848000
H	2.557088000	2.457890000	0.140761000
H	4.590414000	-1.273703000	4.021682000
C	2.888163000	-0.126396000	4.683797000
H	1.116767000	1.017421000	5.037240000
C	0.140681000	2.615478000	2.926723000
C	-1.109420000	0.568018000	3.278400000
H	-1.449301000	-4.090969000	0.881259000
C	0.567039000	-4.859193000	0.675684000
C	-0.847888000	-3.978774000	-1.201656000
H	2.638025000	-5.211325000	0.148520000
C	1.537938000	-4.635515000	-1.627524000
H	0.210896000	-3.718323000	-3.075852000
H	6.137678000	-0.267671000	-1.980670000
C	5.709677000	1.643332000	-1.051216000
C	4.702694000	0.810184000	-3.195373000
C	3.919990000	2.978108000	-2.199263000
H	3.060024000	2.012370000	-3.940518000
H	4.793192000	3.446277000	-0.270367000
H	3.196987000	-0.095415000	5.724095000
H	-0.772318000	3.111689000	2.584417000
H	0.995303000	3.050735000	2.404722000
H	0.249675000	2.817707000	4.001365000
H	-1.078245000	0.664436000	4.373309000
H	-1.158005000	-0.493008000	3.025017000
H	-2.020369000	1.057253000	2.920166000
H	0.245752000	-5.885431000	0.461349000
H	0.773099000	-4.809400000	1.752516000
H	-1.658257000	-3.289203000	-1.478217000
H	-1.194292000	-4.990665000	-1.443912000
H	2.442445000	-4.422307000	-2.211284000
H	1.237043000	-5.660219000	-1.875847000
H	6.492142000	2.179389000	-1.600942000
H	6.142947000	1.356416000	-0.084438000
H	4.411841000	-0.076822000	-3.773179000
H	5.465734000	1.333605000	-3.783215000
H	3.064728000	3.653398000	-2.059730000
H	4.671722000	3.535286000	-2.770473000
C	-6.565322000	1.213167000	-0.877903000
H	-6.535587000	0.936414000	-1.933294000
H	-6.606266000	2.299565000	-0.786389000
C	-5.474832000	0.551323000	-0.042301000
C	-5.650582000	-0.978137000	-0.041515000
H	-5.580743000	0.910779000	0.990849000
C	-8.129347000	-0.743706000	-0.420791000
C	-7.054648000	-1.381820000	0.423637000
H	-5.467062000	-1.379425000	-1.045451000
H	-4.900899000	-1.425513000	0.616547000
H	-9.148576000	-0.871178000	-0.053430000
H	-8.083494000	-1.012279000	-1.477831000
H	-7.190204000	-2.466884000	0.349465000
H	-7.206979000	-1.127320000	1.480245000
H	-8.194153000	1.156446000	0.398227000
O	-7.936972000	0.755283000	-0.451675000

6_{OH6Z}

Sum of electronic and zero-point Energies = -2163.503331
Sum of electronic and thermal Free Energies = -2163.574568
Esolv = -2165.1940651

C	1.461873000000	-1.084910000000	1.482624000000
C	1.492226000000	-0.371726000000	2.680270000000
H	1.450613000000	0.711914000000	2.685387000000
Au	1.241885000000	-0.202029000000	-0.337855000000
C	1.533665000000	-2.481850000000	1.513925000000
H	1.522697000000	-3.059970000000	0.594062000000
C	1.625705000000	-3.153757000000	2.733871000000
H	1.680181000000	-4.238842000000	2.742298000000
C	1.655273000000	-2.439698000000	3.930017000000
H	1.734457000000	-2.962736000000	4.878134000000
C	1.589468000000	-1.049540000000	3.898791000000
H	1.618672000000	-0.479859000000	4.823544000000
P	-1.205824000000	0.225607000000	-0.303785000000
C	-1.448317000000	0.817465000000	-2.029610000000
C	-2.330261000000	-1.301644000000	-0.108851000000
C	-1.627729000000	1.715520000000	0.811577000000
C	-2.718093000000	1.199829000000	-2.503698000000
C	-0.371226000000	0.874995000000	-2.926603000000
C	-1.978400000000	-2.245123000000	-1.285205000000
C	-3.847817000000	-1.015574000000	-0.133364000000
C	-1.991162000000	-2.028728000000	1.214512000000
C	-2.899132000000	2.499553000000	0.414839000000
C	-1.777125000000	1.253447000000	2.278125000000
C	-0.422969000000	2.682679000000	0.699298000000
H	-3.566091000000	1.176655000000	-1.833293000000
C	-2.922364000000	1.617224000000	-3.811738000000
C	-0.582822000000	1.292747000000	-4.246334000000
N	0.995856000000	0.473823000000	-2.543091000000
H	-2.204417000000	-1.760905000000	-2.242986000000
H	-0.901328000000	-2.467705000000	-1.273682000000
C	-2.775189000000	-3.557783000000	-1.172231000000
C	-4.635944000000	-2.336657000000	-0.021540000000
H	-4.137390000000	-0.522446000000	-1.065872000000
H	-4.125444000000	-0.353719000000	0.695248000000
C	-2.784660000000	-3.344183000000	1.319163000000
H	-2.232461000000	-1.397383000000	2.074333000000
H	-0.920326000000	-2.240619000000	1.267453000000
H	-2.811203000000	2.871595000000	-0.610918000000
H	-3.783826000000	1.853662000000	0.460974000000
C	-3.094958000000	3.705958000000	1.356008000000
C	-1.949376000000	2.472531000000	3.203514000000
H	-2.660894000000	0.613014000000	2.375761000000
H	-0.911006000000	0.659908000000	2.590133000000
C	-0.616920000000	3.894941000000	1.627980000000
H	-0.315559000000	3.024025000000	-0.339585000000
H	0.507019000000	2.163537000000	0.968605000000
H	-3.916290000000	1.904693000000	-4.139666000000
C	-1.844862000000	1.662534000000	-4.690531000000
H	0.242684000000	1.328281000000	-4.948956000000
C	1.939232000000	1.584141000000	-2.841704000000
C	1.366843000000	-0.734279000000	-3.334241000000
H	-2.509773000000	-4.198131000000	-2.022391000000
C	-4.278086000000	-3.245469000000	-1.205892000000
C	-2.422555000000	-4.262086000000	0.144458000000
H	-5.706357000000	-2.099109000000	-0.046833000000

C	-4.288039000000	-3.040847000000	1.295799000000
H	-2.516482000000	-3.828922000000	2.265523000000
H	-4.009944000000	4.229005000000	1.052680000000
C	-1.891838000000	4.649480000000	1.228523000000
C	-3.225200000000	3.227245000000	2.807589000000
C	-0.737297000000	3.406017000000	3.078285000000
H	-2.034830000000	2.111652000000	4.235517000000
H	0.254124000000	4.553959000000	1.527160000000
H	-1.981434000000	1.982594000000	-5.718727000000
H	2.952989000000	1.282726000000	-2.582663000000
H	1.656506000000	2.461746000000	-2.258418000000
H	1.928035000000	1.845363000000	-3.903735000000
H	1.363417000000	-0.518319000000	-4.407669000000
H	0.648423000000	-1.529902000000	-3.130773000000
H	2.365610000000	-1.067325000000	-3.049958000000
H	-4.855502000000	-4.176352000000	-1.152914000000
H	-4.545256000000	-2.760163000000	-2.153892000000
H	-1.352876000000	-4.508425000000	0.170132000000
H	-2.968610000000	-5.210178000000	0.221950000000
H	-4.562429000000	-2.408516000000	2.150257000000
H	-4.864838000000	-3.969044000000	1.389998000000
H	-2.028564000000	5.525251000000	1.874342000000
H	-1.807349000000	5.023342000000	0.199733000000
H	-4.101887000000	2.575828000000	2.918336000000
H	-3.381533000000	4.084691000000	3.473398000000
H	0.180097000000	2.884802000000	3.382558000000
H	-0.851014000000	4.261832000000	3.754633000000
C	3.369561000000	-0.298779000000	-0.520575000000
H	3.446911000000	-0.348588000000	-1.614827000000
C	4.068889000000	1.018842000000	-0.126326000000
C	4.139831000000	-1.503968000000	0.008909000000
H	4.140063000000	-1.525129000000	1.103163000000
H	3.677366000000	-2.441661000000	-0.317931000000
C	6.196586000000	-0.072846000000	-0.203173000000
H	7.163931000000	0.050609000000	-0.697674000000
H	6.363506000000	0.035608000000	0.879107000000
C	5.585421000000	-1.433038000000	-0.511807000000
H	5.594931000000	-1.589801000000	-1.598205000000
H	6.192742000000	-2.229144000000	-0.065270000000
O	5.381886000000	0.986649000000	-0.691645000000
C	4.094898000000	1.379110000000	1.356078000000
H	4.811385000000	2.191908000000	1.505398000000
H	3.112793000000	1.735974000000	1.675401000000
H	4.371722000000	0.548026000000	2.008130000000
H	3.593626000000	1.856313000000	-0.648747000000

6_{OH6E}

Sum of electronic and zero-point Energies = -2163.827292

Sum of electronic and thermal Free Energies = -2163.904513

Esolv = -2165.269491

C	2.823033000000	1.176674000000	-1.108030000000
C	2.586584000000	0.801441000000	-2.448244000000
H	2.762521000000	-0.210561000000	-2.793687000000
Au	0.880650000000	0.034099000000	0.243150000000
C	2.550837000000	2.518973000000	-0.761049000000
H	2.706550000000	2.871187000000	0.252485000000
C	2.127911000000	3.441740000000	-1.714514000000
H	1.960259000000	4.471909000000	-1.416980000000
C	1.956653000000	3.058030000000	-3.042412000000
H	1.669069000000	3.786870000000	-3.791663000000
C	2.160182000000	1.727122000000	-3.397085000000
H	2.015929000000	1.406183000000	-4.423906000000
P	-1.412637000000	-0.235835000000	0.285284000000
C	-1.817075000000	-0.928643000000	1.933656000000
C	-2.215766000000	1.486464000000	0.129997000000
C	-1.887565000000	-1.519399000000	-1.029462000000
C	-3.175512000000	-1.113409000000	2.254234000000
C	-0.848948000000	-1.273714000000	2.907456000000
C	-1.976375000000	2.221170000000	1.470275000000
C	-3.731711000000	1.449598000000	-0.170614000000
C	-1.512594000000	2.286658000000	-0.993775000000
C	-3.343604000000	-2.028034000000	-0.919929000000
C	-1.660321000000	-0.925834000000	-2.437658000000
C	-0.943376000000	-2.730385000000	-0.836930000000
H	-3.937894000000	-0.852126000000	1.530949000000
C	-3.580783000000	-1.625738000000	3.480089000000
C	-1.276506000000	-1.795492000000	4.133383000000
N	0.575192000000	-1.103931000000	2.699614000000
H	-2.455344000000	1.681405000000	2.294672000000
H	-0.898209000000	2.257680000000	1.687289000000
C	-2.541356000000	3.652553000000	1.394958000000
C	-4.286180000000	2.887817000000	-0.231522000000
H	-4.273162000000	0.896972000000	0.603920000000
H	-3.919059000000	0.950655000000	-1.128003000000
C	-2.079694000000	3.717758000000	-1.061561000000
H	-1.646140000000	1.796379000000	-1.963148000000
H	-0.432997000000	2.324867000000	-0.800646000000
H	-3.510517000000	-2.483572000000	0.061645000000
H	-4.055417000000	-1.203463000000	-1.030120000000
C	-3.608575000000	-3.085339000000	-2.011413000000
C	-1.923398000000	-1.999362000000	-3.510053000000
H	-2.338398000000	-0.082353000000	-2.607474000000
H	-0.634088000000	-0.542571000000	-2.525593000000
C	-1.212859000000	-3.795018000000	-1.915467000000
H	-1.087386000000	-3.163140000000	0.161682000000
H	0.103171000000	-2.395472000000	-0.900578000000
H	-4.637975000000	-1.751920000000	3.690518000000
C	-2.622700000000	-1.974310000000	4.426366000000
H	-0.536142000000	-2.065370000000	4.880877000000
C	1.283886000000	-2.385155000000	2.701980000000
C	1.161478000000	-0.160735000000	3.658435000000
H	-2.364368000000	4.142808000000	2.359545000000
C	-4.049234000000	3.588576000000	1.113016000000
C	-1.837701000000	4.430389000000	0.275238000000
H	-5.361517000000	2.827759000000	-0.435379000000
C	-3.585128000000	3.662414000000	-1.354296000000
H	-1.564202000000	4.251717000000	-1.869195000000
H	-4.649547000000	-3.415128000000	-1.917416000000
C	-2.665766000000	-4.277546000000	-1.805811000000
C	-3.378682000000	-2.475554000000	-3.400396000000
C	-0.972107000000	-3.185608000000	-3.303822000000
H	-1.752392000000	-1.551281000000	-4.496132000000
H	-0.529003000000	-4.637140000000	-1.751747000000
H	-2.919276000000	-2.380082000000	5.388586000000
H	2.336794000000	-2.210673000000	2.447943000000
H	0.847806000000	-3.050679000000	1.953524000000
H	1.261271000000	-2.896605000000	3.675868000000
H	1.137630000000	-0.521477000000	4.697218000000
H	0.626853000000	0.790472000000	3.612207000000
H	2.209878000000	0.015202000000	3.392454000000
H	-4.469384000000	4.600696000000	1.087547000000
H	-4.565301000000	3.052997000000	1.920037000000
H	-0.760869000000	4.502357000000	0.481296000000
H	-2.220792000000	5.456637000000	0.231638000000
H	-3.769270000000	3.181431000000	-2.323649000000
H	-3.993372000000	4.677454000000	-1.422832000000
H	-2.862360000000	-5.049213000000	-2.559120000000
H	-2.841082000000	-4.739432000000	-0.825884000000
H	-4.066461000000	-1.637384000000	-3.570094000000
H	-3.591479000000	-3.219504000000	-4.176844000000

H	0.072663000000	-2.857047000000	-3.402282000000
H	-1.137936000000	-3.941269000000	-4.080377000000
C	3.610547000000	0.277359000000	-0.112485000000
H	2.970060000000	-0.260036000000	0.629257000000
C	4.320368000000	-0.839462000000	-0.896885000000
H	4.782238000000	-0.442381000000	-1.803990000000
C	4.545013000000	1.175473000000	0.732614000000
H	4.999448000000	1.923381000000	0.072483000000
H	3.934948000000	1.725853000000	1.451595000000
C	3.514491000000	-2.096009000000	-1.151931000000
H	3.232033000000	-2.576995000000	-0.206728000000
H	2.577338000000	-1.852475000000	-1.654909000000
H	4.065081000000	-2.806034000000	-1.773940000000
H	5.295162000000	-2.066376000000	0.469095000000
C	6.459527000000	-0.371147000000	0.505672000000
H	7.252230000000	-0.984298000000	0.937044000000
H	6.856547000000	0.210220000000	-0.328856000000
C	5.647674000000	0.435679000000	1.478998000000
H	5.255841000000	-0.212289000000	2.274172000000
H	6.312816000000	1.153747000000	1.970708000000
O	5.546861000000	-1.360534000000	-0.153549000000

2_v

Sum of electronic and zero-point Energies = -1699.395813
Sum of electronic and thermal Free Energies = -1699.455856
Esolv = -1700,5591499

C	0.075583000	-0.566681000	-3.033020000
C	-0.935344000	-0.763114000	-3.891401000
H	-1.937813000	-1.064886000	-3.594800000
Au	-0.044948000	-1.482519000	-1.278567000
P	0.035100000	0.217595000	0.339116000
C	0.003411000	-0.867953000	1.812736000
C	1.716592000	1.072547000	0.189359000
C	-1.574541000	1.203389000	0.230879000
C	0.025013000	-0.297654000	3.099270000
C	-0.072879000	-2.265335000	1.694508000
C	2.781917000	-0.011761000	-0.104823000
C	2.081260000	1.761921000	1.535089000
C	1.702857000	2.125691000	-0.937653000
C	-1.539128000	2.336308000	1.297625000
C	-1.782607000	1.828786000	-1.165230000
C	-2.742660000	0.238357000	0.536976000
H	0.082329000	0.776515000	3.223793000
C	-0.022000000	-1.099047000	4.234463000
C	-0.113991000	-3.062170000	2.838043000
N	-0.115284000	-2.909602000	0.372831000
H	2.794183000	-0.760277000	0.698475000
H	2.551462000	-0.529100000	-1.047092000
C	4.170033000	0.650423000	-0.224553000
C	3.472387000	2.424221000	1.391354000
H	2.127390000	1.024837000	2.342418000
H	1.339172000	2.522083000	1.802172000
C	3.104595000	2.759535000	-1.058494000
H	0.976131000	2.913701000	-0.713439000
H	1.414097000	1.671737000	-1.893013000
H	-1.400159000	1.926308000	2.302835000
H	-0.721079000	3.035319000	1.092807000
C	-2.887254000	3.094899000	1.251596000
C	-3.119605000	2.597906000	-1.186381000
H	-0.966521000	2.510663000	-1.419178000
H	-1.799181000	1.042819000	-1.928474000
C	-4.072785000	1.020064000	0.504375000
H	-2.614940000	-0.229965000	1.519632000
H	-2.772913000	-0.561795000	-0.217913000
H	-0.003876000	-0.639438000	5.217356000
C	-0.088592000	-2.483328000	4.103419000
H	-0.168501000	-4.142885000	2.755399000
C	-1.378452000	-3.692352000	0.199613000
C	1.063791000	-3.810571000	0.179409000
H	4.901099000	-0.136833000	-0.440338000
C	4.521175000	1.345925000	1.097406000
C	4.145022000	1.674907000	-1.367422000
H	3.693226000	2.912629000	2.347630000
C	3.450450000	3.460189000	0.262024000
H	3.072215000	3.490803000	-1.873571000
H	-2.836681000	3.881100000	2.014064000
C	-4.034240000	2.127188000	1.567084000
C	-3.082994000	3.715540000	-0.135185000
C	-4.274919000	1.634539000	-0.886582000
H	-3.239811000	3.029740000	-2.186137000
H	-4.882338000	0.316155000	0.727506000
H	-0.122180000	-3.116993000	4.983905000
H	-1.414485000	-4.095004000	-0.816500000
H	-2.237227000	-3.042962000	0.372122000
H	-1.416180000	-4.531481000	0.900289000
H	1.039212000	-4.641127000	0.890899000
H	1.984538000	-3.243993000	0.322129000
H	1.037260000	-4.225392000	-0.832018000
H	5.515259000	1.800489000	1.026129000
H	4.566306000	0.617429000	1.916790000
H	3.916701000	1.181851000	-2.321613000
H	5.135065000	2.129240000	-1.481880000
H	2.723465000	4.253099000	0.478274000
H	4.429345000	3.945818000	0.184359000
H	-4.986166000	2.669169000	1.574293000
H	-3.911209000	1.695949000	2.568653000
H	-2.278080000	4.427553000	-0.356631000
H	-4.019521000	4.283386000	-0.160241000
H	-4.328529000	0.847573000	-1.650416000
H	-5.229807000	2.170020000	-0.923467000
H	-0.770768000	-0.644754000	-4.963755000
H	1.038296000	-0.161053000	-3.338128000

3_v^F

Sum of electronic and zero-point Energies = -1970.948462
Sum of electronic and thermal Free Energies = -1971.017281
Esolv = -1972.2911855

C	1.394907000	0.172640000	-1.944856000
C	1.459589000	-0.553890000	-3.054554000
H	1.382164000	-1.637755000	-3.079675000
Au	1.237549000	-0.568246000	-0.061014000
P	-1.038678000	0.125726000	0.229847000
C	-1.228027000	-0.309702000	2.001680000
C	-1.180470000	2.021243000	0.059137000
C	-2.279562000	-0.950875000	-0.739020000
C	-2.430668000	-0.036482000	2.680861000
C	-0.208595000	-0.970298000	2.701145000
C	0.133071000	2.631613000	0.606875000
C	-2.352020000	2.613636000	0.881317000
C	-1.370038000	2.414486000	-1.423699000
C	-3.736630000	-0.478627000	-0.517629000
C	-1.967653000	-0.939343000	-2.251823000
C	-2.128290000	-2.400961000	-0.222164000
H	-3.239979000	0.464280000	2.167355000
C	-2.611190000	-0.393000000	4.011140000
C	-0.395778000	-1.329354000	4.038857000
N	1.063445000	-1.346440000	2.047325000
H	0.257710000	2.363193000	1.665024000

H	1.000441000	2.234825000	0.062129000
C	0.105269000	4.165554000	0.455497000
C	-2.379052000	4.149078000	0.718800000
H	-2.225144000	2.382327000	1.943204000
H	-3.310482000	2.195621000	0.553692000
C	-1.372333000	3.949904000	-1.558819000
H	-2.324391000	2.024912000	-1.794245000
H	-0.582104000	1.985296000	-2.051378000
H	-3.995522000	-0.494892000	0.545502000
H	-3.869284000	0.545706000	-0.883123000
C	-4.700823000	-1.419905000	-1.271140000
C	-2.948682000	-1.867549000	-2.995183000
H	-2.040106000	0.072095000	-2.661817000
H	-0.943757000	-1.282568000	-2.421419000
C	-3.106044000	-3.328090000	-0.972781000
H	-2.330162000	-2.452544000	0.854072000
H	-1.097695000	-2.748677000	-0.390355000
H	-3.548816000	-0.166127000	4.508094000
C	-1.586578000	-1.040042000	4.694537000
H	0.382333000	-1.849242000	4.587507000
C	1.153233000	-2.838034000	2.009323000
C	2.207150000	-0.788267000	2.823805000
H	1.050687000	4.565934000	0.840722000
C	-1.070063000	4.738820000	1.257581000
C	-0.051081000	4.524165000	-1.029296000
H	-3.226257000	4.528943000	1.301102000
C	-2.549109000	4.522053000	-0.758559000
H	-1.486838000	4.196798000	-2.620400000
H	-5.721889000	-1.063735000	-1.092760000
C	-4.544724000	-2.848590000	-0.733811000
C	-4.387398000	-1.385064000	-2.771631000
C	-2.791792000	-3.301603000	-2.473998000
H	-2.704414000	-1.829509000	-4.063074000
H	-2.981428000	-4.343866000	-0.579938000
H	-1.711153000	-1.326993000	5.733743000
H	2.066609000	-3.145193000	1.499304000
H	0.290036000	-3.236577000	1.476170000
H	1.166995000	-3.251179000	3.021834000
H	2.210454000	-1.160178000	3.851427000
H	2.135528000	0.300531000	2.842318000
H	3.143308000	-1.093773000	2.356418000
H	-1.085133000	5.831047000	1.170453000
H	-0.954779000	4.508466000	2.324441000
H	0.796224000	4.134285000	-1.610544000
H	-0.039176000	5.612732000	-1.153997000
H	-3.499079000	4.134149000	-1.147313000
H	-2.586560000	5.611826000	-0.867666000
H	-5.249279000	-3.517950000	-1.240007000
H	-4.788803000	-2.885170000	0.335709000
H	-4.517681000	-0.369650000	-3.167137000
H	-5.088816000	-2.027031000	-3.316357000
H	-1.773090000	-3.667993000	-2.659109000
H	-3.469706000	-3.974805000	-3.010638000
C	3.324611000	-1.339228000	-0.346777000
H	3.280499000	-1.783522000	-1.339037000
H	3.389503000	-2.069131000	0.457513000
C	4.180795000	-0.228665000	-0.204826000
C	4.590725000	0.692698000	-1.297607000
H	4.391551000	0.121198000	0.806202000
C	6.835070000	-0.333371000	-1.220118000
C	6.098986000	0.996273000	-1.243039000
H	4.305784000	0.284714000	-2.270344000
H	4.045467000	1.637832000	-1.157782000
H	7.903238000	-0.214430000	-1.014853000
H	6.725404000	-0.868399000	-2.167002000
H	6.391193000	1.602556000	-2.105388000
H	6.337521000	1.577001000	-0.343687000
H	6.673190000	-1.022982000	0.623022000
O	6.217114000	-1.158286000	-0.218921000
H	1.585039000	-0.060744000	-4.017968000
H	1.472626000	1.257868000	-1.962827000

3_{vz}

Sum of electronic and zero-point Energies = -2010.225841
Sum of electronic and thermal Free Energies = -2010.297775
Esolv = -2011.6077338

C	1.144692000	-0.625238000	-2.006811000
C	0.973070000	-1.725193000	-2.729366000
H	0.752769000	-2.705029000	-2.315919000
Au	1.162061000	-0.610463000	0.025661000
P	-1.091217000	0.169756000	0.227160000
C	-1.235359000	0.122651000	2.052858000
C	-1.213936000	2.008780000	-0.282472000
C	-2.354361000	-1.048310000	-0.508561000
C	-2.418057000	0.561658000	2.678589000
C	-0.171815000	-0.295892000	2.862261000
C	-0.390555000	2.841556000	0.727767000
C	-2.685753000	2.489700000	-0.264593000
C	-0.623379000	2.236695000	-1.690646000
C	-3.752148000	-0.915230000	0.148454000
C	-2.493085000	-0.837670000	-2.032898000
C	-1.827527000	-2.473302000	-0.214778000
H	-3.254343000	0.898991000	2.081206000
C	-2.544881000	0.574734000	4.061364000
C	-0.308748000	-0.291765000	4.253486000
N	1.099813000	-0.779690000	2.283963000
H	-0.771326000	2.706750000	1.745684000
H	0.659123000	2.509614000	0.720709000
C	-0.463044000	4.335720000	0.348865000
C	-2.741531000	3.988068000	-0.631035000
H	-3.128069000	2.357160000	0.727259000
H	-3.287357000	1.919931000	-0.980369000
C	-0.712684000	3.730127000	-2.064573000
H	-1.136348000	1.627578000	-2.442291000
H	0.432720000	1.947107000	-1.688446000
H	-3.685316000	-1.095354000	1.225739000
H	-4.164802000	0.087679000	-0.005253000
C	-4.709402000	-1.962550000	-0.461879000
C	-3.445434000	-1.901557000	-2.615109000
H	-2.899804000	0.158231000	-2.241381000
H	-1.517589000	-0.906354000	-2.522479000
C	-2.787186000	-3.520011000	-0.816179000
H	-1.748737000	-2.626180000	0.870401000
H	-0.826372000	-2.606592000	-0.645681000
H	-3.468093000	0.919757000	4.515431000
C	-1.485285000	0.141645000	4.852591000
H	0.500754000	-0.629757000	4.891415000
C	1.230340000	-2.233566000	2.612157000
C	2.238471000	-0.014669000	2.870619000
H	0.124480000	4.900279000	1.082028000
C	-1.927078000	4.796620000	0.387152000
C	0.108362000	4.547716000	-1.058871000
H	-3.792070000	4.298765000	-0.600727000
C	-2.175773000	4.189245000	-2.041287000
H	-0.299499000	3.851414000	-3.072627000
H	-5.687502000	-1.831844000	0.015126000
C	-4.172598000	-3.369735000	-0.173727000
C	-4.830495000	-1.747992000	-1.975034000
C	-2.891081000	-3.305002000	-2.332839000
H	-3.512863000	-1.738902000	-3.696774000

H	-2.382817000	-4.516925000	-0.604893000
H	-1.569434000	0.139070000	5.934590000
H	2.160733000	-2.631556000	2.207103000
H	0.387130000	-2.776177000	2.182612000
H	1.234356000	-2.385712000	3.695071000
H	2.291893000	-0.147747000	3.953793000
H	2.110595000	1.046400000	2.651339000
H	3.179296000	-0.370428000	2.449769000
H	-1.988037000	5.864998000	0.151660000
H	-2.341908000	4.672776000	1.395619000
H	1.165038000	4.248920000	-1.093639000
H	0.075110000	5.611262000	-1.320258000
H	-2.767118000	3.627872000	-2.775732000
H	-2.241348000	5.245239000	-2.326148000
H	-4.856243000	-4.123558000	-0.579919000
H	-4.115681000	-3.545610000	0.908163000
H	-5.244023000	-0.754787000	-2.191426000
H	-5.527435000	-2.478209000	-2.401501000
H	-1.909370000	-3.431300000	-2.807961000
H	-3.549804000	-4.062984000	-2.771199000
C	3.773835000	-0.177857000	-0.326647000
H	3.799529000	0.511539000	0.517021000
C	3.413369000	-1.487871000	-0.057721000
C	4.212882000	0.405846000	-1.623456000
H	4.288321000	-0.358070000	-2.401441000
H	3.459189000	1.134008000	-1.953073000
H	7.068226000	-0.800931000	0.527530000
C	6.701416000	0.311865000	-1.044298000
H	7.607796000	0.926704000	-0.964376000
H	6.894779000	-0.475615000	-1.787972000
C	5.542613000	1.183285000	-1.485187000
H	5.417393000	2.001649000	-0.766470000
H	5.773119000	1.638374000	-2.453323000
O	6.345523000	-0.244012000	0.214704000
C	3.611543000	-2.658570000	-0.972306000
H	4.622521000	-3.050094000	-0.802070000
H	2.916302000	-3.471468000	-0.745283000
H	3.518505000	-2.406343000	-2.027625000
H	3.397326000	-1.744714000	0.999399000
H	1.058788000	-1.672674000	-3.814871000
H	1.343706000	0.339397000	-2.462844000

3_{vE}

Sum of electronic and zero-point Energies = -2010.221971
Sum of electronic and thermal Free Energies = -2010.294314
Esolv = -2011.6054364

C	1.214877000	-0.119755000	-2.045722000
C	1.034432000	-1.120411000	-2.897406000
H	0.824328000	-2.144350000	-2.604090000
Au	1.275249000	-0.155383000	-0.007047000
P	-1.105906000	0.066755000	0.205261000
C	-1.205764000	0.157681000	2.034374000
C	-1.743136000	1.733708000	-0.473407000
C	-1.983125000	-1.541766000	-0.347272000
C	-2.456412000	0.275674000	2.672352000
C	-0.055789000	0.141237000	2.833142000
C	-0.975877000	2.849796000	0.272841000
C	-3.261104000	1.898779000	-0.213927000
C	-1.476665000	1.876672000	-1.988156000
C	-3.291198000	-1.793253000	0.444567000
C	-2.324675000	-1.500371000	-1.852768000
C	-1.014445000	-2.710691000	-0.047551000
H	-3.365358000	0.283488000	2.087322000
C	-2.561782000	0.371242000	4.053609000
C	-0.167095000	0.238420000	4.223425000
N	1.298304000	0.068638000	2.244923000
H	-1.139783000	2.774430000	1.354525000
H	0.104665000	2.748939000	0.087076000
C	-1.451652000	4.230553000	-0.224480000
C	-3.719402000	3.286209000	-0.714283000
H	-3.482020000	1.833113000	0.854677000
H	-3.829179000	1.116198000	-0.729600000
C	-1.950741000	3.261039000	-2.475178000
H	-1.988745000	1.094032000	-2.553938000
H	-0.407886000	1.777591000	-2.191522000
H	-3.084537000	-1.856257000	1.516846000
H	-4.007855000	-0.979963000	0.280852000
C	-3.922635000	-3.128741000	-0.007807000
C	-2.934863000	-2.850185000	-2.282410000
H	-3.054031000	-0.706181000	-2.047120000
H	-1.435838000	-1.282592000	-2.451060000
C	-1.648441000	-4.044568000	-0.491154000
H	-0.790903000	-2.746326000	1.027785000
H	-0.064032000	-2.566034000	-0.579215000
H	-3.540177000	0.458722000	4.514706000
C	-1.410172000	0.350461000	4.833716000
H	0.716638000	0.236158000	4.852168000
C	2.035290000	-1.080783000	2.844402000
C	2.004157000	1.350621000	2.557207000
H	-0.896521000	4.999869000	0.324671000
C	-2.955615000	4.377953000	0.046708000
C	-1.180520000	4.357909000	-1.729038000
H	-4.793206000	3.372589000	-0.512749000
C	-3.455189000	3.409673000	-2.218748000
H	-1.746729000	3.326107000	-3.550066000
H	-4.846367000	-3.266060000	0.565871000
C	-2.949125000	-4.275756000	0.289131000
C	-4.236987000	-3.083703000	-1.507259000
C	-1.947108000	-3.989905000	-1.996112000
H	-3.142816000	-2.799210000	-3.357155000
H	-0.936493000	-4.850750000	-0.278231000
H	-1.473324000	0.423963000	5.914649000
H	3.049337000	-1.105194000	2.450010000
H	1.519316000	-2.010022000	2.598708000
H	2.101297000	-0.987630000	3.930887000
H	2.099559000	1.485561000	3.638058000
H	1.432236000	2.183604000	2.147147000
H	3.002197000	1.343643000	2.121557000
H	-3.296511000	5.367812000	-0.276583000
H	-3.160133000	4.310795000	1.122962000
H	-0.104221000	4.277964000	-1.933525000
H	-1.493733000	5.346286000	-2.083367000
H	-4.016323000	2.645638000	-2.771517000
H	-3.805318000	4.382005000	-2.582922000
H	-3.395265000	-5.232819000	-0.003298000
H	-2.745244000	-4.338515000	1.365756000
H	-4.960145000	-2.287900000	-1.725867000
H	-4.701487000	-4.025332000	-1.820913000
H	-1.020680000	-3.849699000	-2.569028000
H	-2.372484000	-4.944381000	-2.325672000
C	3.716134000	-0.026183000	-0.073115000
H	3.973298000	-0.024508000	0.985380000
C	3.552537000	-1.259345000	-0.664640000
H	3.451106000	-1.272218000	-1.746773000
C	4.067130000	1.231457000	-0.826773000
H	3.804066000	1.124243000	-1.884724000
H	3.499419000	2.083700000	-0.432525000
C	3.704049000	-2.589583000	-0.024620000
H	3.763830000	-2.561604000	1.062923000
H	2.931787000	-3.296871000	-0.342405000

H	4.658433000	-2.987427000	-0.396438000
H	7.147141000	-1.204495000	-1.098396000
C	6.506736000	0.612094000	-1.414654000
H	7.531399000	0.994911000	-1.322266000
H	6.265343000	0.577693000	-2.488897000
C	5.568778000	1.571597000	-0.701340000
H	5.852980000	1.629042000	0.356230000
H	5.716735000	2.572361000	-1.121854000
O	6.388690000	-0.676764000	-0.823236000
H	1.107979000	-0.932230000	-3.968232000
H	1.442883000	0.889032000	-2.387524000

3'$_{vE}$

Sum of electronic and zero-point Energies = -2010.225477

Sum of electronic and thermal Free Energies = -2010.296415

Esolv = -2011.6076949

C	1.162943000	-0.659133000	-1.985955000
C	1.013773000	-1.778564000	-2.683968000
H	0.834197000	-2.756945000	-2.248354000
Au	1.193628000	-0.578115000	0.053557000
P	-1.077736000	0.171328000	0.207623000
C	-1.251227000	0.161841000	2.030696000
C	-1.241342000	1.991866000	-0.351156000
C	-2.307220000	-1.097360000	-0.508434000
C	-2.456969000	0.573927000	2.630641000
C	-0.191656000	-0.216997000	2.863174000
C	-0.451802000	2.872191000	0.645210000
C	-2.725020000	2.435731000	-0.361415000
C	-0.639837000	2.197665000	-1.758175000
C	-3.711555000	-0.984515000	0.137980000
C	-2.445128000	-0.925756000	-2.037780000
C	-1.746921000	-2.501457000	-0.177695000
H	-3.290333000	0.884428000	2.015079000
C	-2.611645000	0.590429000	4.010403000
C	-0.355736000	-0.209082000	4.251716000
N	1.121328000	-0.621422000	2.316462000
H	-0.840245000	2.753510000	1.662380000
H	0.606234000	2.567878000	0.656632000
C	-0.557656000	4.353581000	0.226315000
C	-2.814604000	3.921487000	-0.769389000
H	-3.173904000	2.318789000	0.629544000
H	-3.304593000	1.831346000	-1.066793000
C	-0.762721000	3.677772000	-2.172850000
H	-1.128617000	1.555940000	-2.498724000
H	0.423488000	1.935311000	-1.736829000
H	-3.646927000	-1.139082000	1.219144000
H	-4.148416000	0.003944000	-0.040742000
C	-4.639910000	-2.069174000	-0.451555000
C	-3.366732000	-2.027702000	-2.598757000
H	-2.877846000	0.053654000	-2.270671000
H	-1.466913000	-0.979898000	-2.522611000
C	-2.677873000	-3.585722000	-0.757950000
H	-1.668203000	-2.626199000	0.911122000
H	-0.741929000	-2.621316000	-0.602053000
H	-3.552775000	0.912389000	4.444173000
C	-1.557123000	0.188985000	4.824951000
H	0.453979000	-0.510537000	4.907580000
C	1.398038000	-2.029781000	2.734098000
C	2.164641000	0.296439000	2.860839000
H	0.006401000	4.952401000	0.950758000
C	-2.033222000	4.777819000	0.235496000
C	0.024969000	4.542674000	-1.180114000
H	-3.872945000	4.205884000	-0.759328000
C	-2.237300000	4.099340000	-2.178227000
H	-0.340653000	3.783324000	-3.178946000
H	-5.623193000	-1.950849000	0.018028000
C	-4.070110000	-3.455107000	-0.126431000
C	-4.758725000	-1.894291000	-1.969802000
C	-2.778924000	-3.409603000	-2.279857000
H	-3.431893000	-1.892859000	-3.684397000
H	-2.249892000	-4.566898000	-0.521015000
H	-1.663071000	0.188516000	5.905036000
H	2.366773000	-2.348869000	2.352047000
H	0.616751000	-2.680725000	2.339012000
H	1.413740000	-2.118120000	3.823569000
H	2.208921000	0.240395000	3.951479000
H	1.929605000	1.320520000	2.568094000
H	3.141855000	0.018411000	2.468737000
H	-2.118063000	5.837595000	-0.029603000
H	-2.456817000	4.670606000	1.242226000
H	1.089281000	4.270121000	-1.195371000
H	-0.031836000	5.597697000	-1.470352000
H	-2.805219000	3.503261000	-2.903721000
H	-2.326537000	5.145219000	-2.492456000
H	-4.732503000	-4.235428000	-0.517565000
H	-4.014941000	-3.603064000	0.959721000
H	-5.196147000	-0.917412000	-2.212060000
H	-5.434633000	-2.652125000	-2.381740000
H	-1.791906000	-3.522655000	-2.747100000
H	-3.416269000	-4.194036000	-2.703079000
C	3.410072000	-1.431667000	0.067231000
H	3.658763000	-1.323008000	1.122204000
C	3.772393000	-0.364780000	-0.742137000
H	3.690101000	-0.508435000	-1.817322000
C	5.536806000	1.396788000	-1.076242000
C	6.673973000	0.406976000	-0.925786000
H	6.861988000	-1.516459000	-1.288664000
H	5.290360000	1.499628000	-2.139029000
H	5.848525000	2.382859000	-0.719101000
H	7.002153000	0.349460000	0.122807000
H	7.530657000	0.733639000	-1.529447000
C	4.275410000	0.965608000	-0.295013000
H	4.471149000	0.968361000	0.782622000
H	3.504004000	1.723708000	-0.492355000
O	6.175439000	-0.844906000	-1.379508000
C	3.367886000	-2.848950000	-0.440998000
H	3.121275000	-2.897399000	-1.502217000
H	4.368068000	-3.280979000	-0.316549000
H	2.673979000	-3.482372000	0.118933000
H	1.067550000	-1.741494000	-3.772030000
H	1.324353000	0.299840000	-2.469582000

TS1v$_{OH5}$

Sum of electronic and zero-point Energies = -1970.947680

Sum of electronic and thermal Free Energies = -1971.015149

Esolv = -1972.2897206

C	1.417283000	0.133226000	-1.947439000
C	1.502837000	-0.607941000	-3.046647000
H	1.461247000	-1.694081000	-3.052863000
Au	1.250675000	-0.579442000	-0.056028000
P	-1.030744000	0.127502000	0.228315000
C	-1.233495000	-0.299774000	2.001210000
C	-1.174741000	2.022372000	0.055475000
C	-2.280120000	-0.941714000	-0.737888000
C	-2.437672000	-0.023793000	2.676296000
C	-0.217454000	-0.959004000	2.706840000
C	0.140281000	2.630720000	0.602337000
C	-2.345266000	2.623775000	0.871258000

C	-1.357785000	2.412558000	-1.429181000
C	-3.737467000	-0.469398000	-0.524291000
C	-1.962172000	-0.933960000	-2.250026000
C	-2.133289000	-2.392010000	-0.219282000
H	-3.244484000	0.476216000	2.158096000
C	-2.623549000	-0.376228000	4.006914000
C	-0.409400000	-1.314506000	4.045016000
N	1.058226000	-1.336808000	2.060042000
H	0.263021000	2.364813000	1.661424000
H	1.006348000	2.227682000	0.059592000
C	0.119329000	4.164035000	0.446516000
C	-2.366057000	4.158347000	0.703963000
H	-2.223059000	2.394817000	1.934306000
H	-3.304456000	2.208162000	0.542510000
C	-1.353997000	3.947133000	-1.570501000
H	-2.312044000	2.025003000	-1.802000000
H	-0.569243000	1.977931000	-2.052772000
H	-4.000669000	-0.483497000	0.537886000
H	-3.867713000	0.554891000	-0.891034000
C	-4.699932000	-1.409950000	-1.280117000
C	-2.940780000	-1.862156000	-2.996255000
H	-2.033474000	0.076809000	-2.662005000
H	-0.937310000	-1.276746000	-2.415798000
C	-3.108332000	-3.319475000	-0.972380000
H	-2.339889000	-2.441773000	0.856207000
H	-1.102175000	-2.741058000	-0.381751000
H	-3.562722000	-0.147177000	4.499952000
C	-1.602158000	-1.022594000	4.695789000
H	0.366425000	-1.833558000	4.597767000
C	1.151221000	-2.827756000	2.029845000
C	2.196061000	-0.771841000	2.838418000
H	1.065243000	4.562615000	0.832751000
C	-1.055789000	4.744750000	1.243705000
C	-0.032080000	4.518676000	-1.039739000
H	-3.212833000	4.544989000	1.282414000
C	-2.530472000	4.527030000	-0.775235000
H	-1.464962000	4.190984000	-2.633224000
H	-5.721772000	-1.053271000	-1.107225000
C	-4.547655000	-2.838356000	-0.740579000
C	-4.380058000	-1.378135000	-2.779474000
C	-2.787767000	-3.295639000	-2.472338000
H	-2.692297000	-1.826345000	-4.063322000
H	-2.986712000	-4.335003000	-0.577746000
H	-1.730784000	-1.306837000	5.735250000
H	2.066733000	-3.134370000	1.523177000
H	0.290641000	-3.230113000	1.495225000
H	1.162073000	-3.236886000	3.044112000
H	2.198063000	-1.139863000	3.867590000
H	2.119861000	0.316709000	2.852462000
H	3.134319000	-1.074843000	2.373436000
H	-1.066153000	5.836821000	1.153123000
H	-0.943990000	4.517117000	2.311555000
H	0.815128000	4.123351000	-1.617597000
H	-0.015927000	5.606833000	-1.168111000
H	-3.480891000	4.141316000	-1.165098000
H	-2.563280000	5.616591000	-0.888516000
H	-5.250696000	-3.507737000	-1.248994000
H	-4.796261000	-2.873231000	0.327985000
H	-4.507431000	-0.363106000	-3.177023000
H	-5.079886000	-2.020206000	-3.326263000
H	-1.768491000	-3.663055000	-2.652367000
H	-3.464086000	-3.969031000	-3.010905000
C	3.303074000	-1.323255000	-0.314344000
H	3.291337000	-1.786671000	-1.300235000
H	3.403552000	-2.064000000	0.479028000
C	4.251413000	-0.251000000	-0.191481000
C	4.589661000	0.707383000	-1.290830000
H	4.419823000	0.138325000	0.814463000
C	6.771184000	-0.355358000	-1.207509000
C	6.095620000	1.002406000	-1.274537000
H	4.275033000	0.303635000	-2.256178000
H	4.029685000	1.635366000	-1.117764000
H	7.816284000	-0.316347000	-0.891804000
H	6.703569000	-0.899427000	-2.151604000
H	6.399225000	1.559730000	-2.164520000
H	6.363468000	1.608277000	-0.401131000
H	6.424358000	-1.082509000	0.624100000
O	6.004496000	-1.136463000	-0.247599000
H	1.601262000	-0.126834000	-4.019067000
H	1.458374000	1.219972000	-1.986535000

TS1v$_{OH5Z}$

Sum of electronic and zero-point Energies = -2010.218876
Sum of electronic and thermal Free Energies = -2010.288233
Esolv = -2011.5989191

C	1.375344000	-0.427356000	-1.885830000
C	1.348501000	-1.517375000	-2.645583000
H	1.238624000	-2.526728000	-2.259402000
Au	1.248765000	-0.398221000	0.135846000
P	-1.113008000	0.115793000	0.200949000
C	-1.365408000	0.097381000	2.016648000
C	-1.502842000	1.893203000	-0.377095000
C	-2.167067000	-1.287656000	-0.539105000
C	-2.628813000	0.375234000	2.572190000
C	-0.301094000	-0.153892000	2.891055000
C	-0.866910000	2.871706000	0.638952000
C	-3.023147000	2.167511000	-0.461960000
C	-0.863811000	2.157797000	-1.758716000
C	-3.600837000	-1.348537000	0.040376000
C	-2.252035000	-1.146741000	-2.075973000
C	-1.452556000	-2.612511000	-0.176517000
H	-3.468602000	0.583036000	1.922796000
C	-2.833292000	0.389247000	3.945551000
C	-0.511511000	-0.145733000	4.273644000
N	1.066935000	-0.425797000	2.396106000
H	-1.286374000	2.718463000	1.639110000
H	0.217172000	2.690251000	0.704605000
C	-1.121279000	4.326597000	0.195191000
C	-3.264523000	3.627610000	-0.896566000
H	-3.502386000	2.008602000	0.509128000
H	-3.497174000	1.492350000	-1.182054000
C	-1.132981000	3.609376000	-2.200536000
H	-1.242529000	1.458775000	-2.511754000
H	0.219694000	2.013731000	-1.686555000
H	-3.565804000	-1.484213000	1.125816000
H	-4.146918000	-0.421866000	-0.167767000
C	-4.361985000	-2.541655000	-0.575741000
C	-3.007086000	-2.354099000	-2.666674000
H	-2.786347000	-0.227829000	-2.342209000
H	-1.250672000	-1.085345000	-2.512780000
C	-2.216078000	-3.806732000	-0.782360000
H	-1.400735000	-2.722655000	0.915643000
H	-0.423513000	-2.607167000	-0.560656000
H	-3.819412000	0.606269000	4.343136000
C	-1.768657000	0.121899000	4.801039000
H	0.304317000	-0.345795000	4.960179000
C	1.473808000	-1.787250000	2.853914000
C	1.987667000	0.609499000	2.948148000
H	-0.664852000	4.994204000	0.935387000
C	-2.634382000	4.579054000	0.129241000

C	-0.497797000	4.568740000	-1.185461000
H	-4.347279000	3.791331000	-0.940682000
C	-2.644264000	3.858735000	-2.279746000
H	-0.679210000	3.754884000	-3.187987000
H	-5.373396000	-2.546492000	-0.153423000
C	-3.640236000	-3.845579000	-0.212954000
C	-4.431806000	-2.390064000	-2.100100000
C	-2.271223000	-3.653115000	-2.309051000
H	-3.041003000	-2.234063000	-3.755663000
H	-1.682337000	-4.727269000	-0.517296000
H	-1.910779000	0.124033000	5.876967000
H	2.478012000	-2.018031000	2.498141000
H	0.771187000	-2.521613000	2.457384000
H	1.475057000	-1.851375000	3.945350000
H	2.018261000	0.571741000	4.040397000
H	1.642159000	1.596775000	2.638301000
H	2.999802000	0.443612000	2.576976000
H	-2.827149000	5.619550000	-0.155746000
H	-3.090045000	4.433583000	1.117052000
H	0.590195000	4.419246000	-1.146411000
H	-0.660974000	5.607664000	-1.493701000
H	-3.104503000	3.194001000	-3.021836000
H	-2.838154000	4.884565000	-2.612997000
H	-4.183831000	-4.704033000	-0.623591000
H	-3.614403000	-3.979464000	0.876194000
H	-4.973733000	-1.474931000	-2.370853000
H	-4.990676000	-3.226748000	-2.534684000
H	-1.257766000	-3.646987000	-2.731640000
H	-2.789567000	-4.511491000	-2.751304000
C	4.170676000	0.212952000	-0.350116000
H	4.019891000	1.150070000	0.187107000
C	3.398672000	-0.914464000	0.189921000
C	4.508530000	0.408898000	-1.803620000
H	4.499133000	-0.542127000	-2.341935000
H	3.752894000	1.054990000	-2.259796000
H	6.004240000	-0.838895000	0.613251000
C	6.754845000	0.275106000	-0.919305000
H	7.622109000	0.815850000	-0.536923000
H	7.058459000	-0.688047000	-1.337690000
C	5.888964000	1.073704000	-1.872711000
H	5.835942000	2.118818000	-1.551084000
H	6.298801000	1.058170000	-2.885145000
O	5.855428000	0.034714000	0.219030000
C	3.687994000	-2.299653000	-0.371521000
H	4.736801000	-2.594620000	-0.227753000
H	3.086497000	-3.059160000	0.135558000
H	3.475180000	-2.371867000	-1.439074000
H	3.525107000	-0.907811000	1.276437000
H	1.422260000	-1.425252000	-3.728645000
H	1.458734000	0.561676000	-2.328660000

TS1v$_{OH5E}$

Sum of electronic and zero-point Energies = –2010.212698

Sum of electronic and thermal Free Energies = –2010.281290

Esolv = -2011.5941209

C	1.048421000	-0.723286000	-2.072545000
C	1.063008000	-1.820832000	-2.824384000
H	1.173308000	-2.828996000	-2.432637000
Au	1.175548000	-0.757743000	-0.043071000
P	-1.049590000	0.161403000	0.232502000
C	-1.215462000	0.044098000	2.051470000
C	-1.085538000	2.035652000	-0.161635000
C	-2.401729000	-0.914977000	-0.566522000
C	-2.369170000	0.514945000	2.706611000
C	-0.164441000	-0.450282000	2.833157000
C	-0.227984000	2.761881000	0.902289000
C	-2.522267000	2.609006000	-0.129709000
C	-0.461634000	2.317794000	-1.546635000
C	-3.793492000	-0.735004000	0.086049000
C	-2.508793000	-0.608753000	-2.077939000
C	-1.970026000	-2.388351000	-0.366440000
H	-3.194890000	0.908549000	2.129210000
C	-2.480013000	0.490510000	4.090492000
C	-0.283921000	-0.482724000	4.226278000
N	1.062527000	-1.004808000	2.220798000
H	-0.629537000	2.591242000	1.906390000
H	0.799382000	2.365023000	0.889664000
C	-0.203940000	4.276733000	0.614904000
C	-2.485968000	4.125845000	-0.405981000
H	-2.984377000	2.442580000	0.848078000
H	-3.149257000	2.119388000	-0.882003000
C	-0.456377000	3.832421000	-1.832599000
H	-1.002094000	1.789972000	-2.339993000
H	0.576510000	1.963981000	-1.552507000
H	-3.747565000	-0.984692000	1.150814000
H	-4.139178000	0.300490000	-0.004747000
C	-4.811880000	-1.676209000	-0.591980000
C	-3.524198000	-1.567565000	-2.730934000
H	-2.841772000	0.423592000	-2.234043000
H	-1.533936000	-0.722018000	-2.561814000
C	-2.991853000	-3.331691000	-1.031896000
H	-1.905091000	-2.615003000	0.706804000
H	-0.979707000	-2.557923000	-0.808690000
H	-3.381651000	0.860817000	4.567481000
C	-1.430932000	-0.012611000	4.853994000
H	0.512639000	-0.883859000	4.843436000
C	1.055083000	-2.477941000	2.471167000
C	2.264629000	-0.386279000	2.841621000
H	0.405735000	4.761303000	1.386615000
C	-1.637954000	4.823500000	0.665632000
C	0.397223000	4.538986000	-0.771619000
H	-3.515414000	4.499796000	-0.368145000
C	-1.889797000	4.377005000	-1.795882000
H	-0.023658000	3.989940000	-2.827691000
H	-5.785506000	-1.512788000	-0.115855000
C	-4.370623000	-3.131082000	-0.389683000
C	-4.902337000	-1.361642000	-2.090154000
C	-3.066736000	-3.019568000	-2.533189000
H	-3.571281000	-1.335039000	-3.801091000
H	-2.655249000	-4.364199000	-0.880484000
H	-1.502125000	-0.045045000	5.936470000
H	1.943415000	-2.930580000	2.037993000
H	0.164089000	-2.913463000	2.017186000
H	1.040146000	-2.686115000	3.545297000
H	2.304561000	-0.567493000	3.918709000
H	2.248697000	0.690595000	2.665745000
H	3.156140000	-0.825444000	2.397609000
H	-1.631460000	5.905839000	0.493853000
H	-2.072223000	4.665343000	1.660985000
H	1.435136000	4.179058000	-0.814152000
H	0.431502000	5.616402000	-0.968974000
H	-2.502821000	3.895924000	-2.568557000
H	-1.888634000	5.450086000	-2.018198000
H	-5.098349000	-3.812182000	-0.845274000
H	-4.335249000	-3.375902000	0.679735000
H	-5.246461000	-0.331224000	-2.246563000
H	-5.641794000	-2.015729000	-2.566098000
H	-2.089420000	-3.180121000	-3.007074000
H	-3.769738000	-3.703721000	-3.021823000

C	4.099565000	-0.254249000	-0.239375000
H	4.346382000	-0.012564000	0.795022000
C	3.267029000	-1.448003000	-0.428148000
C	3.974154000	0.958272000	-1.128642000
H	3.554480000	0.672069000	-2.098408000
H	3.292521000	1.679500000	-0.665961000
H	5.838299000	-1.538180000	-1.033771000
C	6.272747000	0.380445000	-1.579404000
H	7.324986000	0.539045000	-1.337864000
H	6.166596000	0.015407000	-2.604345000
C	5.373624000	1.563749000	-1.288342000
H	5.691302000	2.063539000	-0.367689000
H	5.413944000	2.297412000	-2.096665000
O	5.767743000	-0.647583000	-0.655572000
H	0.942484000	-1.737730000	-3.903906000
H	0.928333000	0.261212000	-2.512836000
C	3.721255000	-2.695645000	0.323610000
H	3.936274000	-2.522174000	1.381946000
H	2.987382000	-3.501586000	0.243914000
H	4.643408000	-3.107290000	-0.109332000
H	3.178661000	-1.666957000	-1.493524000

TS1’v$_{OH5E}$
Sum of electronic and zero-point Energies = -2010.221676
Sum of electronic and thermal Free Energies = -2010.290809
Esolv = -2011.6020462

C	1.289426000	-0.536681000	-1.966901000
C	1.168056000	-1.624039000	-2.721432000
H	0.996940000	-2.622821000	-2.330339000
Au	1.239518000	-0.489500000	0.065188000
P	-1.084378000	0.145861000	0.211407000
C	-1.271217000	0.137704000	2.034620000
C	-1.395003000	1.939726000	-0.359046000
C	-2.232001000	-1.203753000	-0.490383000
C	-2.501471000	0.468069000	2.634311000
C	-0.189026000	-0.163987000	2.870485000
C	-0.651439000	2.881121000	0.617870000
C	-2.899926000	2.300301000	-0.364445000
C	-0.816867000	2.163284000	-1.774295000
C	-3.643821000	-1.194860000	0.144629000
C	-2.370791000	-1.053858000	-2.022421000
C	-1.571068000	-2.563012000	-0.155098000
H	-3.353734000	0.714839000	2.015640000
C	-2.657870000	0.482586000	4.013990000
C	-0.351591000	-0.154409000	4.259408000
N	1.152235000	-0.474991000	2.327674000
H	-1.025968000	2.753228000	1.639388000
H	0.422413000	2.637735000	0.626355000
C	-0.844603000	4.347539000	0.181349000
C	-3.079114000	3.771288000	-0.792737000
H	-3.334719000	2.171358000	0.631588000
H	-3.448957000	1.651872000	-1.055246000
C	-1.024102000	3.627769000	-2.207279000
H	-1.276986000	1.487693000	-2.502574000
H	0.258281000	1.953833000	-1.762200000
H	-3.574195000	-1.337340000	1.227405000
H	-4.151022000	-0.241491000	-0.040503000
C	-4.486639000	-2.346873000	-0.443283000
C	-3.205745000	-2.221871000	-2.584302000
H	-2.871819000	-0.110021000	-2.265383000
H	-1.386124000	-1.038112000	-2.498986000
C	-2.416018000	-3.716277000	-0.732152000
H	-1.483149000	-2.677571000	0.934272000
H	-0.558597000	-2.608379000	-0.577891000
H	-3.619409000	0.739856000	4.446248000
C	-1.577505000	0.163142000	4.830924000
H	0.478410000	-0.390290000	4.916934000
C	1.562476000	-1.830969000	2.797057000
C	2.111167000	0.553761000	2.824577000
H	-0.312144000	4.989069000	0.893306000
C	-2.342151000	4.686416000	0.194169000
C	-0.282128000	4.550435000	-1.231438000
H	-4.151526000	3.996830000	-0.780285000
C	-2.520747000	3.964203000	-2.207621000
H	-0.615598000	3.745206000	-3.217861000
H	-5.479830000	-2.301707000	0.018103000
C	-3.817521000	-3.685838000	-0.108739000
C	-4.607691000	-2.188554000	-1.963587000
C	-2.521802000	-3.556367000	-2.255539000
H	-3.275005000	-2.097540000	-3.671088000
H	-1.918836000	-4.662701000	-0.488174000
H	-1.682533000	0.164577000	5.911106000
H	2.553686000	-2.070078000	2.413247000
H	0.842751000	-2.568031000	2.437778000
H	1.599338000	-1.876207000	3.888598000
H	2.168259000	0.544378000	3.916473000
H	1.781381000	1.540327000	2.496132000
H	3.106663000	0.349945000	2.429825000
H	-2.489666000	5.735767000	-0.085183000
H	-2.752431000	4.569193000	1.205339000
H	0.796077000	4.338373000	-1.249055000
H	-0.400784000	5.596506000	-1.535678000
H	-3.057152000	3.326384000	-2.921572000
H	-2.672421000	4.999068000	-2.534668000
H	-4.418376000	-4.515129000	-0.498910000
H	-3.757071000	-3.823634000	0.978531000
H	-5.113772000	-1.247223000	-2.213055000
H	-5.223608000	-2.995666000	-2.376352000
H	-1.526435000	-3.599837000	-2.716993000
H	-3.098648000	-4.386969000	-2.677862000
C	3.358654000	-1.172498000	0.033853000
H	3.673068000	-0.964658000	1.060547000
C	4.031256000	-0.295943000	-0.901525000
H	3.860144000	-0.521588000	-1.952147000
C	5.703727000	1.487040000	-1.252109000
C	6.643781000	0.362442000	-0.868558000
H	6.095770000	-1.545934000	-0.471856000
H	5.603104000	1.536520000	-2.341060000
H	6.079615000	2.453681000	-0.909024000
H	6.953119000	0.422594000	0.179468000
H	7.522843000	0.287607000	-1.512740000
C	4.357122000	1.138552000	-0.603235000
H	4.387959000	1.311728000	0.478087000
H	3.566689000	1.772862000	-1.021004000
O	5.835481000	-0.831491000	-1.071531000
C	3.386350000	-2.659699000	-0.298804000
H	3.142909000	-2.840922000	-1.347897000
H	4.378348000	-3.092089000	-0.115935000
H	2.687340000	-3.234502000	0.314411000
H	1.222540000	-1.538202000	-3.806204000
H	1.442128000	0.441669000	-2.417330000

TS1v$_{OH6}$
Sum of electronic and zero-point Energies = -1970.933363
Sum of electronic and thermal Free Energies = -1971.000405
Esolv = -1972.2813016

C	-1.431076000	0.088076000	-1.973362000
C	-1.520158000	1.199226000	-2.694302000

H	-1.501555000	2.202704000	-2.278990000
Au	-1.380750000	-0.087133000	0.051588000
P	1.017886000	0.001064000	0.205237000
C	1.174271000	-0.161215000	2.025495000
C	1.862979000	-1.518981000	-0.573962000
C	1.666065000	1.730962000	-0.279330000
C	2.443886000	-0.152593000	2.634506000
C	0.049352000	-0.316707000	2.845760000
C	1.382119000	-2.767409000	0.202153000
C	3.405522000	-1.422397000	-0.481532000
C	1.455475000	-1.680148000	-2.055583000
C	2.987942000	2.090234000	0.443550000
C	1.893925000	1.823435000	-1.804450000
C	0.591259000	2.754182000	0.160107000
H	3.333289000	-0.040410000	2.029687000
C	2.591243000	-0.277331000	4.009857000
C	0.201666000	-0.438473000	4.229905000
N	-1.313945000	-0.418107000	2.281240000
H	1.644330000	-2.688873000	1.263538000
H	0.286532000	-2.850677000	0.132132000
C	2.029724000	-4.033696000	-0.395373000
C	4.037283000	-2.699098000	-1.075903000
H	3.728688000	-1.328836000	0.559507000
H	3.770778000	-0.546666000	-1.028769000
C	2.109990000	-2.945337000	-2.645592000
H	1.742484000	-0.803583000	-2.643984000
H	0.368949000	-1.783296000	-2.128412000
H	2.848922000	2.065339000	1.528428000
H	3.780854000	1.379625000	0.184445000
C	3.426660000	3.516051000	0.043394000
C	2.316269000	3.259066000	-2.177998000
H	2.687187000	1.131232000	-2.107842000
H	0.988216000	1.545150000	-2.351148000
C	1.032593000	4.179461000	-0.229999000
H	0.441129000	2.697567000	1.247211000
H	-0.370578000	2.527984000	-0.320586000
H	3.583292000	-0.265055000	4.449444000
C	1.463058000	-0.413795000	4.812510000
H	-0.661554000	-0.565877000	4.874266000
C	-2.201463000	0.592714000	2.924334000
C	-1.821844000	-1.798154000	2.554338000
H	1.676282000	-4.899155000	0.177003000
C	3.557016000	-3.922978000	-0.284788000
C	1.625784000	-4.176321000	-1.868505000
H	5.125679000	-2.603183000	-0.990550000
C	3.636431000	-2.832941000	-2.549558000
H	1.806769000	-3.023080000	-3.696045000
H	4.367278000	3.730169000	0.563611000
C	2.350315000	4.517912000	0.479250000
C	3.635196000	3.597689000	-1.473125000
C	1.227628000	4.253685000	-1.751091000
H	2.451514000	3.299436000	-3.264791000
H	0.248928000	4.879618000	0.082858000
H	1.559477000	-0.509239000	5.889259000
H	-3.210535000	0.499112000	2.522147000
H	-1.812664000	1.592983000	2.727315000
H	-2.260418000	0.442287000	4.005052000
H	-1.884563000	-1.980656000	3.630732000
H	-1.139812000	-2.525093000	2.112331000
H	-2.815264000	-1.925752000	2.124667000
H	4.025633000	-4.831460000	-0.679359000
H	3.860891000	-3.843599000	0.766886000
H	0.536316000	-4.283201000	-1.959484000
H	2.066150000	-5.086368000	-2.290989000
H	3.995301000	-1.970007000	-3.124763000
H	4.106578000	-3.720389000	-2.987809000
H	2.660198000	5.537689000	0.224896000
H	2.218246000	4.490555000	1.568508000
H	4.426868000	2.907387000	-1.790777000
H	3.965867000	4.604619000	-1.751861000
H	0.285196000	4.038550000	-2.272113000
H	1.516156000	5.270626000	-2.039691000
C	-3.848565000	0.990724000	-0.218393000
H	-3.759084000	1.427039000	-1.206923000
H	-3.855312000	1.689530000	0.614110000
C	-3.686800000	-0.379251000	-0.034701000
C	-4.186118000	-1.319442000	-1.115899000
H	-3.874251000	-0.735179000	0.978570000
C	-6.298953000	0.081927000	-1.551874000
C	-5.725739000	-1.279484000	-1.162669000
H	-3.794858000	-1.023560000	-2.094526000
H	-3.850250000	-2.342785000	-0.922215000
H	-7.386877000	0.029787000	-1.663225000
H	-5.888908000	0.418976000	-2.508544000
H	-6.072260000	-2.009034000	-1.903305000
H	-6.145633000	-1.602496000	-0.200881000
H	-6.517944000	1.019649000	0.177886000
O	-5.978584000	1.135236000	-0.617036000
H	-1.611899000	1.130543000	-3.777862000
H	-1.450121000	-0.895810000	-2.438490000

TS1v$_{OH6Z}$

Sum of electronic and zero-point Energies = -2010.212586
Sum of electronic and thermal Free Energies = -2010.280815
Esolv = -2011.5966095

C	-1.318551000	0.058046000	-2.007232000
C	-1.250549000	1.146164000	-2.763574000
H	-1.111430000	2.151368000	-2.377932000
Au	-1.295090000	-0.088545000	0.011479000
P	1.107764000	-0.015030000	0.220924000
C	1.237189000	-0.198314000	2.042078000
C	1.958939000	-1.532224000	-0.556739000
C	1.800235000	1.708732000	-0.224802000
C	2.495066000	-0.211045000	2.674860000
C	0.096438000	-0.346511000	2.841567000
C	1.428770000	-2.784774000	0.180392000
C	3.499132000	-1.473826000	-0.414969000
C	1.596184000	-1.658072000	-2.053869000
C	3.095791000	2.055936000	0.548750000
C	2.091344000	1.806828000	-1.739161000
C	0.718890000	2.741597000	0.175210000
H	3.395976000	-0.103729000	2.086615000
C	2.617242000	-0.349718000	4.051245000
C	0.222954000	-0.482171000	4.227412000
N	-1.258250000	-0.428308000	2.253347000
H	1.660323000	-2.730049000	1.250576000
H	0.334239000	-2.842990000	0.076755000
C	2.066695000	-4.054245000	-0.419606000
C	4.121931000	-2.751985000	-1.014978000
H	3.790903000	-1.409352000	0.637462000
H	3.901032000	-0.594949000	-0.930765000
C	2.237979000	-2.928267000	-2.646050000
H	1.928175000	-0.780301000	-2.615827000
H	0.510090000	-1.724224000	-2.167293000
H	2.916085000	2.026119000	1.627545000
H	3.891929000	1.339291000	0.316597000
C	3.562496000	3.479098000	0.173269000
C	2.538349000	3.239989000	-2.091116000
H	2.892242000	1.110547000	-2.011504000
H	1.209071000	1.533333000	-2.324920000

C	1.186509000	4.164178000	-0.191540000
H	0.526539000	2.682334000	1.255609000
H	-0.226718000	2.523463000	-0.340558000
H	3.601543000	-0.353913000	4.508095000
C	1.473552000	-0.478687000	4.832854000
H	-0.653019000	-0.604784000	4.855351000
C	-2.145481000	0.583367000	2.891131000
C	-1.782427000	-1.804504000	2.507086000
H	1.677929000	-4.922109000	0.125816000
C	3.592038000	-3.979428000	-0.261746000
C	1.704256000	-4.161805000	-1.906477000
H	5.209185000	-2.682290000	-0.895774000
C	3.763045000	-2.852019000	-2.502168000
H	1.965631000	-2.981514000	-3.706519000
H	4.483617000	3.684151000	0.730711000
C	2.478305000	4.488814000	0.570132000
C	3.831183000	3.565837000	-1.333544000
C	1.442344000	4.242568000	-1.703377000
H	2.716789000	3.283709000	-3.171587000
H	0.397768000	4.870954000	0.093005000
H	1.549566000	-0.584776000	5.910249000
H	-3.150641000	0.490802000	2.479232000
H	-1.754076000	1.583135000	2.696835000
H	-2.214818000	0.433584000	3.971505000
H	-1.862728000	-1.997669000	3.580596000
H	-1.101931000	-2.534094000	2.066974000
H	-2.771078000	-1.918371000	2.061610000
H	4.052725000	-4.890849000	-0.659103000
H	3.865489000	-3.925561000	0.799889000
H	0.615799000	-4.242364000	-2.031279000
H	2.137083000	-5.073953000	-2.332457000
H	4.157540000	-1.987336000	-3.050869000
H	4.227277000	-3.742016000	-2.941807000
H	2.806611000	5.506909000	0.332348000
H	2.302940000	4.458343000	1.653210000
H	4.629009000	2.870243000	-1.622934000
H	4.180412000	4.571247000	-1.594850000
H	0.519233000	4.038040000	-2.262480000
H	1.750664000	5.258071000	-1.976529000
C	-3.531849000	-0.304664000	-0.131639000
H	-3.694592000	-0.737762000	0.859634000
C	-4.066786000	1.025060000	-0.170820000
C	-4.015255000	-1.272448000	-1.211630000
H	-3.857601000	-0.855228000	-2.210828000
H	-3.439042000	-2.202326000	-1.167484000
H	-6.316182000	0.294339000	0.790628000
C	-6.415320000	-0.383552000	-1.074240000
H	-7.465103000	-0.653723000	-0.924258000
H	-6.340737000	0.129911000	-2.035605000
C	-5.503588000	-1.601463000	-1.013128000
H	-5.648715000	-2.124527000	-0.057775000
H	-5.830366000	-2.300711000	-1.791019000
O	-6.070336000	0.624570000	-0.086400000
C	-4.155291000	1.914971000	-1.354765000
H	-4.998429000	2.600957000	-1.247121000
H	-3.248525000	2.533415000	-1.369013000
H	-4.212315000	1.387432000	-2.305343000
H	-4.088957000	1.565217000	0.776327000
H	-1.330904000	1.056799000	-3.846455000
H	-1.450026000	-0.928889000	-2.449134000

TS1v$_{OH6E}$

Sum of electronic and zero-point Energies = -2010.219531
Sum of electronic and thermal Free Energies = -2010.288795
Esolv = -2011.601977

C	1.338523000	-0.159045000	-1.981608000
C	1.224203000	-1.222760000	-2.767585000
H	1.035290000	-2.230449000	-2.409739000
Au	1.298612000	-0.092574000	0.054217000
P	-1.098885000	0.054372000	0.204216000
C	-1.255546000	0.157926000	2.027945000
C	-1.755063000	1.698858000	-0.505036000
C	-1.948251000	-1.560763000	-0.357104000
C	-2.522328000	0.281095000	2.631089000
C	-0.127961000	0.143441000	2.857893000
C	-1.129324000	2.842750000	0.327185000
C	-3.298156000	1.787039000	-0.413381000
C	-1.327886000	1.876427000	-1.978884000
C	-3.299280000	-1.798586000	0.362466000
C	-2.196098000	-1.550846000	-1.882130000
C	-0.999619000	-2.723658000	0.020541000
H	-3.413641000	0.298258000	2.019112000
C	-2.665654000	0.374393000	4.009083000
C	-0.276930000	0.230132000	4.245459000
N	1.242727000	0.066890000	2.307546000
H	-1.404950000	2.748380000	1.383738000
H	-0.031175000	2.794330000	0.262651000
C	-1.615034000	4.204346000	-0.210932000
C	-3.767539000	3.156854000	-0.948444000
H	-3.633432000	1.688510000	0.623114000
H	-3.766416000	0.987646000	-0.997872000
C	-1.819200000	3.238160000	-2.508822000
H	-1.721371000	1.070258000	-2.605071000
H	-0.237338000	1.844247000	-2.051779000
H	-3.152594000	-1.841897000	1.445752000
H	-4.004725000	-0.988357000	0.146035000
C	-3.904899000	-3.141843000	-0.100530000
C	-2.785871000	-2.906709000	-2.319897000
H	-2.904284000	-0.755999000	-2.141152000
H	-1.268609000	-1.352532000	-2.426446000
C	-1.606916000	-4.066094000	-0.433889000
H	-0.840901000	-2.739264000	1.107807000
H	-0.018914000	-2.586203000	-0.455407000
H	-3.655667000	0.468001000	4.443440000
C	-1.536304000	0.342582000	4.821271000
H	0.589405000	0.217666000	4.898194000
C	1.923484000	-1.136590000	2.867526000
C	1.974308000	1.308726000	2.702621000
H	-1.161182000	4.993468000	0.399761000
C	-3.144892000	4.275928000	-0.103173000
C	-1.191161000	4.363735000	-1.676733000
H	-4.859850000	3.190384000	-0.865385000
C	-3.348180000	3.308700000	-2.415143000
H	-1.504814000	3.326420000	-3.555183000
H	-4.860907000	-3.269948000	0.419949000
C	-2.951221000	-4.283232000	0.273245000
C	-4.131064000	-3.123870000	-1.616690000
C	-1.818439000	-4.041328000	-1.954417000
H	-2.931565000	-2.877569000	-3.405699000
H	-0.909502000	-4.868304000	-0.164697000
H	-1.629831000	0.409452000	5.900430000
H	2.940240000	-1.195462000	2.480826000
H	1.369365000	-2.030777000	2.578662000
H	1.977513000	-1.089017000	3.958178000
H	2.040098000	1.393665000	3.790487000
H	1.441224000	2.176804000	2.312789000

H	2.986138000	1.290335000	2.299801000
H	-3.497697000	5.251730000	-0.455358000
H	-3.460309000	4.186574000	0.944288000
H	-0.096397000	4.340347000	-1.764615000
H	-1.515125000	5.339318000	-2.056183000
H	-3.807273000	2.523269000	-3.028856000
H	-3.705449000	4.266044000	-2.810881000
H	-3.380155000	-5.245757000	-0.027145000
H	-2.810472000	-4.325878000	1.360923000
H	-4.838149000	-2.330875000	-1.891112000
H	-4.579235000	-4.070257000	-1.939867000
H	-0.860401000	-3.912061000	-2.475635000
H	-2.225474000	-5.002196000	-2.288792000
C	3.599556000	0.040396000	0.016403000
H	3.823475000	-0.054493000	1.080281000
C	3.868801000	-1.124433000	-0.715299000
H	3.831959000	-1.051228000	-1.798546000
C	3.989914000	1.387075000	-0.582822000
H	3.611101000	1.466971000	-1.608451000
H	3.528110000	2.199943000	-0.012341000
C	3.984653000	-2.498686000	-0.177531000
H	4.060402000	-2.542808000	0.910068000
H	3.127917000	-3.100333000	-0.507943000
H	4.862188000	-2.976002000	-0.626009000
H	6.662309000	-0.910140000	-0.359869000
C	6.257658000	0.629534000	-1.518526000
H	7.324067000	0.875995000	-1.562852000
H	5.868958000	0.687067000	-2.540101000
C	5.514493000	1.595056000	-0.602118000
H	5.920021000	1.531116000	0.416577000
H	5.721068000	2.614748000	-0.946998000
O	6.097578000	-0.744022000	-1.126756000
H	1.322770000	-1.107994000	-3.846712000
H	1.540713000	0.828300000	-2.393511000

4v$_{OH5}$

Sum of electronic and zero-point Energies = -1970.947016
Sum of electronic and thermal Free Energies = -1971.014988
Esolv = -1972.2906146

C	1.432757000	0.058108000	-1.964124000
C	1.536209000	-0.701646000	-3.050044000
H	1.529212000	-1.788443000	-3.032817000
Au	1.256833000	-0.616818000	-0.063448000
P	-1.020229000	0.135256000	0.228219000
C	-1.232765000	-0.282796000	2.002931000
C	-1.143466000	2.031697000	0.058219000
C	-2.297740000	-0.907240000	-0.729753000
C	-2.430768000	0.010926000	2.681284000
C	-0.224382000	-0.953477000	2.708879000
C	0.184571000	2.618960000	0.597178000
C	-2.299661000	2.656977000	0.875092000
C	-1.323073000	2.424227000	-1.426366000
C	-3.748761000	-0.420462000	-0.512256000
C	-1.984023000	-0.900963000	-2.243470000
C	-2.167494000	-2.361293000	-0.215967000
H	-3.231702000	0.520497000	2.163197000
C	-2.618655000	-0.335244000	4.013261000
C	-0.417644000	-1.303164000	4.048615000
N	1.046283000	-1.349483000	2.061846000
H	0.308408000	2.351765000	1.655898000
H	1.040031000	2.198493000	0.050210000
C	0.190904000	4.151906000	0.439942000
C	-2.293778000	4.190995000	0.705910000
H	-2.179500000	2.426806000	1.938227000
H	-3.267107000	2.257791000	0.549876000
C	-1.292627000	3.957841000	-1.571514000
H	-2.284668000	2.052784000	-1.796641000
H	-0.543767000	1.974247000	-2.051211000
H	-4.008976000	-0.434022000	0.550738000
H	-3.868122000	0.606721000	-0.874967000
C	-4.724654000	-1.346871000	-1.267461000
C	-2.974891000	-1.816096000	-2.989302000
H	-2.047249000	0.111317000	-2.653162000
H	-0.962698000	-1.252771000	-2.413930000
C	-3.154681000	-3.275812000	-0.968269000
H	-2.372190000	-2.410843000	0.860010000
H	-1.140727000	-2.722034000	-0.380854000
H	-3.553493000	-0.092261000	4.507899000
C	-1.604678000	-0.993783000	4.701582000
H	0.352575000	-1.831192000	4.600815000
C	1.122049000	-2.840683000	2.037761000
C	2.190330000	-0.794087000	2.835892000
H	1.144906000	4.534909000	0.822470000
C	-0.971138000	4.754694000	1.240114000
C	0.040691000	4.507436000	-1.046212000
H	-3.131154000	4.595253000	1.286021000
C	-2.455888000	4.560386000	-0.773491000
H	-1.402850000	4.201994000	-2.634322000
H	-5.742278000	-0.979531000	-1.091947000
C	-4.587849000	-2.778622000	-0.732164000
C	-4.408029000	-1.316052000	-2.767702000
C	-2.837555000	-3.252485000	-2.468998000
H	-2.729184000	-1.781272000	-4.057128000
H	-3.044379000	-4.293839000	-0.576478000
H	-1.734768000	-1.273791000	5.742018000
H	2.032295000	-3.158340000	1.528095000
H	0.255429000	-3.234879000	1.506703000
H	1.130929000	-3.246951000	3.053349000
H	2.192177000	-1.158912000	3.866396000
H	2.124925000	0.295194000	2.846556000
H	3.123639000	-1.106945000	2.367168000
H	-0.962163000	5.846759000	1.148285000
H	-0.859790000	4.526092000	2.307844000
H	0.878794000	4.096040000	-1.626299000
H	0.075310000	5.595033000	-1.176424000
H	-3.414218000	4.190875000	-1.159691000
H	-2.469475000	5.650219000	-0.888832000
H	-5.299911000	-3.438553000	-1.240554000
H	-4.834093000	-2.813162000	0.336994000
H	-4.524283000	-0.298661000	-3.162736000
H	-5.116765000	-1.948710000	-3.314171000
H	-1.822858000	-3.631001000	-2.652056000
H	-3.522982000	-3.916827000	-3.007485000
C	3.264727000	-1.358265000	-0.308137000
H	3.283127000	-1.833853000	-1.290263000
H	3.393545000	-2.121694000	0.464471000
C	4.330893000	-0.335735000	-0.198727000
C	4.560187000	0.703419000	-1.273434000
H	4.448558000	0.083909000	0.805007000
C	6.732909000	-0.315714000	-1.151681000
C	6.055218000	1.036919000	-1.244291000
H	4.254306000	0.312198000	-2.247217000
H	3.951896000	1.587083000	-1.061803000
H	7.692819000	-0.326730000	-0.633906000
H	6.813662000	-0.831632000	-2.108803000
H	6.374546000	1.574823000	-2.139925000
H	6.304215000	1.656204000	-0.376361000
H	6.142444000	-1.409584000	0.500253000
O	5.773427000	-1.139333000	-0.356021000
H	1.605605000	-0.238162000	-4.033262000

H	1.436728000	1.144373000	-2.030733000

4v$_{OH5Z}$

Sum of electronic and zero-point Energies = -2010.218379
Sum of electronic and thermal Free Energies = -2010.288496
Esolv = -2011.599338

C	1.389737000	-0.422154000	-1.878772000
C	1.372215000	-1.512989000	-2.638026000
H	1.271309000	-2.522874000	-2.250691000
Au	1.254408000	-0.389378000	0.141359000
P	-1.112728000	0.113282000	0.199990000
C	-1.371858000	0.094594000	2.014840000
C	-1.514894000	1.886831000	-0.380573000
C	-2.158632000	-1.296054000	-0.540535000
C	-2.638310000	0.364667000	2.567152000
C	-0.308682000	-0.151044000	2.892255000
C	-0.890078000	2.870669000	0.637322000
C	-3.036105000	2.152400000	-0.473642000
C	-0.870319000	2.154137000	-1.759252000
C	-3.594883000	-1.363706000	0.031387000
C	-2.236274000	-1.158642000	-2.078169000
C	-1.439187000	-2.616842000	-0.172648000
H	-3.477165000	0.568626000	1.915288000
C	-2.847063000	0.375850000	3.939898000
C	-0.523168000	-0.145761000	4.274281000
N	1.063378000	-0.411021000	2.401445000
H	-1.313585000	2.715910000	1.635553000
H	0.194706000	2.695617000	0.708756000
C	-1.150719000	4.323480000	0.190843000
C	-3.284159000	3.610405000	-0.911019000
H	-3.519463000	1.991148000	0.495078000
H	-3.502419000	1.473701000	-1.195466000
C	-1.145737000	3.603523000	-2.204126000
H	-1.241212000	1.452127000	-2.513448000
H	0.213689000	2.016015000	-1.681515000
H	-3.564672000	-1.497423000	1.117245000
H	-4.144633000	-0.440132000	-0.180722000
C	-4.346983000	-2.561517000	-0.586483000
C	-2.982201000	-2.370627000	-2.670798000
H	-2.773546000	-0.242793000	-2.348976000
H	-1.232839000	-1.093087000	-2.509713000
C	-2.193631000	-3.816026000	-0.779962000
H	-1.391584000	-2.724924000	0.919918000
H	-0.408429000	-2.606879000	-0.552184000
H	-3.835564000	0.586791000	4.334845000
C	-1.783484000	0.113478000	4.798255000
H	0.291947000	-0.341071000	4.963050000
C	1.484178000	-1.766067000	2.864559000
C	1.971155000	0.635921000	2.952555000
H	-0.702130000	4.994748000	0.932551000
C	-2.664949000	4.566880000	0.116794000
C	-0.521631000	4.567860000	-1.186882000
H	-4.367581000	3.767928000	-0.961026000
C	-2.658099000	3.843796000	-2.291271000
H	-0.687878000	3.750904000	-3.189441000
H	-5.360605000	-2.571002000	-0.169571000
C	-3.620503000	-3.861192000	-0.217858000
C	-4.409629000	-2.412847000	-2.111495000
C	-2.241816000	-3.665413000	-2.307196000
H	-3.011200000	-2.252702000	-3.760185000
H	-1.656658000	-4.733469000	-0.510627000
H	-1.928824000	0.113414000	5.873758000
H	2.491133000	-1.986768000	2.510041000
H	0.789879000	-2.509335000	2.469986000
H	1.485604000	-1.826712000	3.956200000
H	1.998060000	0.602762000	4.045105000
H	1.616430000	1.618292000	2.637572000
H	2.986145000	0.479163000	2.585160000
H	-2.862462000	5.605897000	-0.170403000
H	-3.124731000	4.419776000	1.102455000
H	0.567037000	4.424841000	-1.142013000
H	-0.689413000	5.605460000	-1.497184000
H	-3.110507000	3.175443000	-3.034937000
H	-2.856413000	4.868091000	-2.626700000
H	-4.157609000	-4.723147000	-0.629761000
H	-3.599537000	-3.993013000	0.871654000
H	-4.954674000	-1.500834000	-2.386524000
H	-4.962078000	-3.253021000	-2.547610000
H	-1.226207000	-3.654878000	-2.724542000
H	-2.753661000	-4.527107000	-2.750622000
C	4.198424000	0.234027000	-0.351972000
H	4.017893000	1.183474000	0.154693000
C	3.394858000	-0.883974000	0.200294000
C	4.517848000	0.409598000	-1.817326000
H	4.504084000	-0.549904000	-2.340482000
H	3.763014000	1.050331000	-2.279496000
H	5.921369000	-0.834467000	0.606945000
C	6.744984000	0.281779000	-0.904129000
H	7.590985000	0.828033000	-0.485663000
H	7.064201000	-0.687707000	-1.294037000
C	5.902968000	1.063203000	-1.891470000
H	5.856425000	2.117547000	-1.601175000
H	6.328434000	1.012940000	-2.896278000
O	5.798866000	0.043540000	0.210913000
C	3.697847000	-2.276287000	-0.340877000
H	4.750452000	-2.564203000	-0.204104000
H	3.108744000	-3.033971000	0.183203000
H	3.476467000	-2.367862000	-1.405372000
H	3.534772000	-0.866422000	1.286168000
H	1.442155000	-1.421318000	-3.721327000
H	1.463118000	0.566783000	-2.323806000

4v$_{OH5E}$

Sum of electronic and zero-point Energies = -2010.212331
Sum of electronic and thermal Free Energies = -2010.281892
Esolv = -2011.594381

C	1.053841000	-0.722803000	-2.072114000
C	1.077556000	-1.819756000	-2.824843000
H	1.198408000	-2.826925000	-2.433634000
Au	1.179448000	-0.755981000	-0.043277000
P	-1.048741000	0.160378000	0.232354000
C	-1.215263000	0.044481000	2.051425000
C	-1.090915000	2.034307000	-0.162597000
C	-2.399787000	-0.918451000	-0.565141000
C	-2.369413000	0.514100000	2.706614000
C	-0.163392000	-0.447781000	2.833341000
C	-0.235925000	2.763663000	0.901339000
C	-2.528587000	2.604973000	-0.132829000
C	-0.466036000	2.316691000	-1.547226000
C	-3.792552000	-0.740080000	0.085396000
C	-2.505512000	-0.614909000	-2.077225000
C	-1.965932000	-2.390936000	-0.362894000
H	-3.195750000	0.906102000	2.128977000
C	-2.479942000	0.490704000	4.090540000
C	-0.282383000	-0.479115000	4.226562000
N	1.064111000	-1.001801000	2.221280000
H	-0.638092000	2.593182000	1.905221000
H	0.792243000	2.368801000	0.890279000

C	-0.214682000	4.278236000	0.612575000
C	-2.495355000	4.121545000	-0.410521000
H	-2.991514000	2.438349000	0.844562000
H	-3.153778000	2.113283000	-0.885279000
C	-0.463591000	3.830992000	-1.834750000
H	-1.004709000	1.787086000	-2.340641000
H	0.572796000	1.964769000	-1.551760000
H	-3.747324000	-0.988135000	1.150598000
H	-4.139873000	0.294718000	-0.007053000
C	-4.808691000	-1.683801000	-0.592356000
C	-3.518692000	-1.576088000	-2.730093000
H	-2.839614000	0.416756000	-2.235474000
H	-1.529809000	-0.727622000	-2.559683000
C	-2.985464000	-3.336935000	-1.028017000
H	-1.901614000	-2.616114000	0.710708000
H	-0.974863000	-2.559419000	-0.803948000
H	-3.381995000	0.860066000	4.567481000
C	-1.429874000	-0.010103000	4.854210000
H	0.514905000	-0.878595000	4.843864000
C	1.057128000	-2.474694000	2.472701000
C	2.265569000	-0.382075000	2.841809000
H	0.393148000	4.764925000	1.384432000
C	-1.649874000	4.822103000	0.661223000
C	0.387378000	4.540448000	-0.773575000
H	-3.525547000	4.493584000	-0.374195000
C	-1.898176000	4.372653000	-1.800036000
H	-0.030185000	3.988557000	-2.829557000
H	-5.783226000	-1.521449000	-0.117724000
C	-4.365292000	-3.137705000	-0.387642000
C	-4.897882000	-1.371457000	-2.091105000
C	-3.059244000	-3.027115000	-2.529841000
H	-3.565025000	-1.345140000	-3.800638000
H	-2.647368000	-4.368705000	-0.874810000
H	-1.500684000	-0.041638000	5.936739000
H	1.945501000	-2.927564000	2.039915000
H	0.166351000	-2.910685000	2.018766000
H	1.041955000	-2.682180000	3.546973000
H	2.306455000	-0.563586000	3.918844000
H	2.248031000	0.694857000	2.666359000
H	3.157451000	-0.819734000	2.396999000
H	-1.645467000	5.904297000	0.488382000
H	-2.084820000	4.663997000	1.656292000
H	1.426089000	4.182604000	-0.814649000
H	0.419581000	5.617754000	-0.971963000
H	-2.509357000	3.889502000	-2.572885000
H	-1.899031000	5.445529000	-2.023413000
H	-5.091406000	-3.820640000	-0.843089000
H	-4.330668000	-3.380972000	0.682160000
H	-5.243399000	-0.341770000	-2.249285000
H	-5.635773000	-2.027328000	-2.567052000
H	-2.081128000	-3.186781000	-3.002394000
H	-3.760671000	-3.713046000	-3.018273000
C	4.118716000	-0.248371000	-0.237984000
H	4.353336000	-0.004107000	0.799030000
C	3.264430000	-1.437696000	-0.422546000
C	3.976035000	0.971285000	-1.119178000
H	3.546744000	0.688396000	-2.085759000
H	3.298817000	1.689216000	-0.646318000
H	5.785575000	-1.540556000	-1.040896000
C	6.263721000	0.379083000	-1.580219000
H	7.315607000	0.515925000	-1.326002000
H	6.159248000	0.013933000	-2.604965000
C	5.376239000	1.571145000	-1.290451000
H	5.702923000	2.074565000	-0.375085000
H	5.417566000	2.298516000	-2.104360000
O	5.733069000	-0.648857000	-0.660790000
H	0.952725000	-1.737542000	-3.903908000
H	0.922949000	0.260414000	-2.512285000
C	3.722072000	-2.687999000	0.325269000
H	3.932722000	-2.518722000	1.385232000
H	2.989978000	-3.495047000	0.240270000
H	4.646619000	-3.099182000	-0.104105000
H	3.184953000	-1.657413000	-1.489007000

$4v_{OH5E'}$

Sum of electronic and zero-point Energies = -2010.221096
Sum of electronic and thermal Free Energies = -2010.290589
Esolv = -2011.6032845

C	1.321593000	-0.511885000	-1.967143000
C	1.199754000	-1.587168000	-2.739837000
H	1.024773000	-2.590964000	-2.363212000
Au	1.253710000	-0.480821000	0.060266000
P	-1.080889000	0.141941000	0.213753000
C	-1.268369000	0.126950000	2.037441000
C	-1.419667000	1.931825000	-0.350328000
C	-2.222387000	-1.213725000	-0.485803000
C	-2.499289000	0.444603000	2.642226000
C	-0.182239000	-0.170875000	2.869919000
C	-0.681464000	2.879232000	0.625265000
C	-2.926211000	2.283123000	-0.353670000
C	-0.845277000	2.161477000	-1.766612000
C	-3.634883000	-1.220427000	0.146030000
C	-2.359519000	-1.064046000	-2.018161000
C	-1.548904000	-2.567939000	-0.154165000
H	-3.354347000	0.688085000	2.026024000
C	-2.653167000	0.450839000	4.022268000
C	-0.341521000	-0.169124000	4.259379000
N	1.161897000	-0.465799000	2.323493000
H	-1.053345000	2.747713000	1.647359000
H	0.394115000	2.643249000	0.632200000
C	-0.885368000	4.344368000	0.190478000
C	-3.117021000	3.752574000	-0.781092000
H	-3.358891000	2.150178000	0.642894000
H	-3.471822000	1.630773000	-1.043566000
C	-1.062378000	3.624628000	-2.198351000
H	-1.303061000	1.484202000	-2.494752000
H	0.230887000	1.956526000	-1.757552000
H	-3.565400000	-1.363433000	1.228850000
H	-4.150753000	-0.271391000	-0.037697000
C	-4.466098000	-2.378981000	-0.444629000
C	-3.182975000	-2.238098000	-2.583873000
H	-2.867924000	-0.124207000	-2.261347000
H	-1.373538000	-1.038851000	-2.492384000
C	-2.381946000	-3.728336000	-0.733583000
H	-1.459208000	-2.684229000	0.934914000
H	-0.535812000	-2.602039000	-0.577181000
H	-3.615699000	0.698401000	4.458000000
C	-1.568571000	0.135764000	4.835284000
H	0.491987000	-0.400779000	4.914022000
C	1.594459000	-1.813204000	2.794735000
C	2.107507000	0.577134000	2.814825000
H	-0.356747000	4.989654000	0.902018000
C	-2.385253000	4.672826000	0.205058000
C	-0.325978000	4.552119000	-1.222859000
H	-4.190806000	3.971447000	-0.767624000
C	-2.561415000	3.950696000	-2.196493000
H	-0.656178000	3.746151000	-3.209471000
H	-5.460691000	-2.345090000	0.014706000
C	-3.784516000	-3.712287000	-0.111965000
C	-4.586120000	-2.219051000	-1.964980000

C	-2.487648000	-3.567094000	-2.256815000
H	-3.252209000	-2.112533000	-3.670581000
H	-1.876431000	-4.670713000	-0.490726000
H	-1.671129000	0.131116000	5.915700000
H	2.588847000	-2.035555000	2.408547000
H	0.886369000	-2.562737000	2.438215000
H	1.635230000	-1.856729000	3.886237000
H	2.167597000	0.571897000	3.906736000
H	1.763345000	1.558140000	2.484448000
H	3.104462000	0.385225000	2.417060000
H	-2.540504000	5.721288000	-0.073686000
H	-2.793320000	4.552181000	1.216736000
H	0.753706000	4.347345000	-1.241529000
H	-0.452222000	5.597552000	-1.526493000
H	-3.093976000	3.309440000	-2.910273000
H	-2.720862000	4.984740000	-2.522662000
H	-4.376988000	-4.546688000	-0.504232000
H	-3.723893000	-3.851247000	0.975171000
H	-5.100326000	-1.281808000	-2.213318000
H	-5.193966000	-3.030947000	-2.380469000
H	-1.491277000	-3.600592000	-2.717098000
H	-3.056554000	-4.402148000	-2.681283000
C	3.341706000	-1.131702000	0.009546000
H	3.687944000	-0.918305000	1.027921000
C	4.106610000	-0.293019000	-0.947264000
H	3.866658000	-0.507274000	-1.988393000
C	5.719069000	1.514671000	-1.267559000
C	6.600827000	0.357802000	-0.847244000
H	5.867320000	-1.510761000	-0.354965000
H	5.669254000	1.577840000	-2.358779000
H	6.112140000	2.463104000	-0.894700000
H	6.913292000	0.416607000	0.198303000
H	7.455483000	0.165034000	-1.496437000
C	4.346566000	1.175842000	-0.674034000
H	4.329117000	1.373384000	0.403580000
H	3.556499000	1.772788000	-1.137782000
O	5.688673000	-0.802044000	-0.994177000
C	3.400318000	-2.629342000	-0.293704000
H	3.129755000	-2.837667000	-1.331625000
H	4.399419000	-3.058526000	-0.130635000
H	2.727156000	-3.203237000	0.348347000
H	1.252983000	-1.485767000	-3.823153000
H	1.475717000	0.471842000	-2.406458000

4v$_{OH6}$

Sum of electronic and zero-point Energies = -1970.939664
Sum of electronic and thermal Free Energies = -1971.006177
Esolv = -1972.2898644

C	1.474214000	-0.164372000	-1.999336000
C	1.487831000	-1.280406000	-2.722959000
H	1.426526000	-2.279425000	-2.299630000
Au	1.414826000	-0.008753000	0.019224000
P	-0.998835000	0.023158000	0.211856000
C	-1.139990000	0.168036000	2.035486000
C	-1.776700000	1.600898000	-0.522418000
C	-1.787291000	-1.638962000	-0.284421000
C	-2.397238000	0.248453000	2.663740000
C	0.003195000	0.230269000	2.842273000
C	-1.239456000	2.802005000	0.292358000
C	-3.322128000	1.594803000	-0.458399000
C	-1.335522000	1.781704000	-1.992928000
C	-3.136291000	-1.916211000	0.420186000
C	-2.002169000	-1.697429000	-1.814001000
C	-0.792628000	-2.745569000	0.143531000
H	-3.299606000	0.211053000	2.068478000
C	-2.517318000	0.371543000	4.041806000
C	-0.120082000	0.347303000	4.230240000
N	1.366411000	0.200380000	2.265256000
H	-1.523886000	2.711913000	1.346794000
H	-0.139393000	2.822332000	0.245849000
C	-1.801798000	4.118492000	-0.279916000
C	-3.871382000	2.919712000	-1.025811000
H	-3.668693000	1.487817000	0.574161000
H	-3.727857000	0.756786000	-1.035394000
C	-1.906637000	3.096005000	-2.559828000
H	-1.660019000	0.938037000	-2.609867000
H	-0.242879000	1.823072000	-2.044148000
H	-3.004241000	-1.915694000	1.506604000
H	-3.873593000	-1.145671000	0.168973000
C	-3.674177000	-3.299090000	-0.004276000
C	-2.525944000	-3.091021000	-2.213625000
H	-2.735004000	-0.942319000	-2.119721000
H	-1.069122000	-1.481675000	-2.343936000
C	-1.331086000	-4.129696000	-0.269319000
H	-0.645725000	-2.715249000	1.232221000
H	0.186349000	-2.579765000	-0.327977000
H	-3.501375000	0.430905000	4.495383000
C	-1.371467000	0.415049000	4.830169000
H	0.759408000	0.393584000	4.863808000
C	2.106707000	-0.962105000	2.833258000
C	2.055133000	1.477907000	2.614287000
H	-1.409674000	4.947870000	0.320365000
C	-3.334771000	4.093872000	-0.196040000
C	-1.366766000	4.276910000	-1.742556000
H	-4.964954000	2.887016000	-0.960900000
C	-3.438646000	3.069425000	-2.489270000
H	-1.583178000	3.184980000	-3.603584000
H	-4.633108000	-3.455774000	0.502809000
C	-2.676605000	-4.382990000	0.423445000
C	-3.872658000	-3.342967000	-1.524302000
C	-1.517042000	-4.168842000	-1.792899000
H	-2.654615000	-3.107209000	-3.302019000
H	-0.603516000	-4.890127000	0.039288000
H	-1.446286000	0.506647000	5.908978000
H	3.111194000	-0.999405000	2.409925000
H	1.572242000	-1.881615000	2.590144000
H	2.198539000	-0.882002000	3.919822000
H	2.155148000	1.587722000	3.697590000
H	1.472255000	2.314440000	2.226906000
H	3.052208000	1.495114000	2.173707000
H	-3.744072000	5.037895000	-0.573299000
H	-3.659610000	4.004817000	0.848600000
H	-0.271363000	4.322905000	-1.814012000
H	-1.747661000	5.221607000	-2.146935000
H	-3.836105000	2.243335000	-3.092672000
H	-3.850129000	3.993550000	-2.910853000
H	-3.056486000	-5.374125000	0.150623000
H	-2.552653000	-4.381158000	1.514084000
H	-4.608143000	-2.591164000	-1.837573000
H	-4.273687000	-4.318544000	-1.822177000
H	-0.556343000	-4.013473000	-2.302142000
H	-1.875734000	-5.158088000	-2.099270000
C	4.122462000	-1.212259000	-0.629971000
H	3.843972000	-1.412903000	-1.664303000
H	3.965248000	-2.098633000	-0.013573000
C	3.586275000	0.069673000	-0.087481000
C	4.112195000	1.274688000	-0.877622000
H	3.893898000	0.163707000	0.961594000
C	6.191548000	0.006291000	-1.519084000
C	5.648015000	1.288859000	-0.934245000

H	3.722734000	1.253478000	-1.902233000
H	3.761594000	2.208777000	-0.428452000
H	7.277447000	-0.094668000	-1.480824000
H	5.843858000	-0.193900000	-2.534292000
H	6.007637000	2.111664000	-1.563133000
H	6.077051000	1.456647000	0.062325000
H	6.081169000	-1.191303000	0.137714000
O	5.675081000	-1.172595000	-0.747203000
H	1.538622000	-1.222401000	-3.809510000
H	1.526652000	0.810955000	-2.480452000

4v$_{OH6Z}$

Sum of electronic and zero-point Energies = -2010.212654

Sum of electronic and thermal Free Energies = -2010.280331

Esolv = -2011.6001416

C	-1.338098000	-0.019821000	-2.024787000
C	-1.288550000	1.063609000	-2.789969000
H	-1.200970000	2.076446000	-2.409813000
Au	-1.317347000	-0.169087000	-0.007037000
P	1.092667000	0.004885000	0.221820000
C	1.230540000	-0.198726000	2.041047000
C	2.033948000	-1.455309000	-0.563417000
C	1.722099000	1.760067000	-0.190563000
C	2.483414000	-0.167534000	2.682597000
C	0.094274000	-0.414908000	2.831159000
C	1.563708000	-2.741473000	0.157406000
C	3.568935000	-1.329210000	-0.421199000
C	1.677829000	-1.582968000	-2.062333000
C	2.995649000	2.159918000	0.592237000
C	2.013886000	1.891358000	-1.702712000
C	0.592690000	2.739919000	0.211188000
H	3.380899000	-0.006817000	2.101332000
C	2.605465000	-0.330790000	4.056241000
C	0.219404000	-0.577182000	4.214622000
N	-1.255226000	-0.537082000	2.235752000
H	1.792517000	-2.688842000	1.228276000
H	0.472766000	-2.849130000	0.053880000
C	2.259280000	-3.972953000	-0.456485000
C	4.251323000	-2.569162000	-1.035304000
H	3.857512000	-1.262253000	0.632093000
H	3.930469000	-0.426692000	-0.926183000
C	2.377296000	-2.814529000	-2.669609000
H	1.968881000	-0.684450000	-2.614211000
H	0.595542000	-1.695955000	-2.176481000
H	2.812599000	2.108290000	1.669872000
H	3.825149000	1.483146000	0.357033000
C	3.399140000	3.606349000	0.236114000
C	2.398079000	3.346286000	-2.036993000
H	2.844849000	1.233861000	-1.980940000
H	1.145812000	1.585903000	-2.294326000
C	0.996176000	4.186548000	-0.135334000
H	0.393301000	2.658865000	1.289035000
H	-0.337240000	2.484021000	-0.316275000
H	3.586520000	-0.299665000	4.519036000
C	1.465188000	-0.531071000	4.827853000
H	-0.653318000	-0.753747000	4.834327000
C	-2.177908000	0.427857000	2.891273000
C	-1.729634000	-1.935126000	2.459346000
H	1.911691000	-4.864672000	0.078390000
C	3.779535000	-3.829354000	-0.297459000
C	1.902189000	-4.080310000	-1.944722000
H	5.334415000	-2.451504000	-0.915638000
C	3.897234000	-2.669150000	-2.523923000
H	2.108081000	-2.868653000	-3.730953000
H	4.307136000	3.848855000	0.800050000
C	2.267818000	4.561324000	0.637318000
C	3.670829000	3.722414000	-1.268461000
C	1.256129000	4.294289000	-1.644633000
H	2.580243000	3.410823000	-3.115894000
H	0.174720000	4.853955000	0.152437000
H	1.540555000	-0.658990000	5.902927000
H	-3.177693000	0.305182000	2.474084000
H	-1.823438000	1.444948000	2.716367000
H	-2.243822000	0.256040000	3.968759000
H	-1.808395000	-2.154565000	3.528074000
H	-1.020562000	-2.629633000	2.007705000
H	-2.710826000	-2.074695000	2.003946000
H	4.282300000	-4.713552000	-0.705438000
H	4.050022000	-3.775262000	0.764962000
H	0.818467000	-4.209656000	-2.070517000
H	2.376888000	-4.966411000	-2.381227000
H	4.250622000	-1.780459000	-3.062273000
H	4.402665000	-3.531339000	-2.973688000
H	2.550375000	5.596164000	0.412956000
H	2.088066000	4.510077000	1.718954000
H	4.500828000	3.066842000	-1.560901000
H	3.975332000	4.745525000	-1.517123000
H	0.345951000	4.053767000	-2.210811000
H	1.519564000	5.325820000	-1.904819000
C	-3.488212000	-0.401216000	-0.139992000
H	-3.692332000	-0.905161000	0.815052000
C	-4.227331000	0.905383000	-0.108098000
C	-3.975058000	-1.324188000	-1.264513000
H	-3.781431000	-0.877375000	-2.244853000
H	-3.419488000	-2.266775000	-1.239748000
H	-6.020595000	0.168097000	0.830658000
C	-6.304688000	-0.368622000	-1.105922000
H	-7.360776000	-0.524184000	-0.876816000
H	-6.224438000	0.224190000	-2.017461000
C	-5.474050000	-1.631199000	-1.130897000
H	-5.669142000	-2.223233000	-0.226721000
H	-5.822896000	-2.238925000	-1.973691000
O	-5.816015000	0.553397000	-0.038844000
C	-4.146052000	1.894937000	-1.238452000
H	-4.935438000	2.644933000	-1.148047000
H	-3.188551000	2.414665000	-1.155547000
H	-4.179880000	1.434700000	-2.226090000
H	-4.143825000	1.421853000	0.851194000
H	-1.325455000	0.961405000	-3.873867000
H	-1.423950000	-1.011529000	-2.467701000

4v$_{OH6E}$

Sum of electronic and zero-point Energies = -2010.223016

Sum of electronic and thermal Free Energies = -2010.290749

Esolv = -2011.6084326

C	1.402352000	-0.101381000	-1.994198000
C	1.382499000	-1.200231000	-2.743178000
H	1.292044000	-2.206522000	-2.343676000
Au	1.334003000	0.017689000	0.026976000
P	-1.082797000	0.027721000	0.212589000
C	-1.234513000	0.167972000	2.035599000
C	-1.865448000	1.605692000	-0.517975000
C	-1.866352000	-1.634009000	-0.293262000
C	-2.494994000	0.244855000	2.658044000
C	-0.095369000	0.234585000	2.847664000
C	-1.327171000	2.805283000	0.298566000
C	-3.410742000	1.599107000	-0.450028000
C	-1.429449000	1.790417000	-1.989551000

C	-3.212707000	-1.922039000	0.412145000
C	-2.084880000	-1.683604000	-1.822740000
C	-0.866710000	-2.739755000	0.124714000
H	-3.394364000	0.201669000	2.058825000
C	-2.622333000	0.371872000	4.034988000
C	-0.226161000	0.355052000	4.234831000
N	1.271271000	0.192272000	2.279653000
H	-1.609408000	2.712235000	1.353329000
H	-0.227141000	2.826285000	0.250487000
C	-1.891483000	4.122938000	-0.268857000
C	-3.962314000	2.925099000	-1.012749000
H	-3.754693000	1.490334000	0.583068000
H	-3.817822000	0.762081000	-1.027593000
C	-2.001831000	3.106418000	-2.551140000
H	-1.757476000	0.949006000	-2.607506000
H	-0.337164000	1.829617000	-2.046033000
H	-3.078746000	-1.927329000	1.498337000
H	-3.953597000	-1.152950000	0.166926000
C	-3.745613000	-3.304324000	-0.020146000
C	-2.603257000	-3.076734000	-2.230707000
H	-2.822182000	-0.930016000	-2.121222000
H	-1.154595000	-1.458702000	-2.353854000
C	-1.399731000	-4.123395000	-0.296111000
H	-0.717040000	-2.716099000	1.213194000
H	0.110417000	-2.565289000	-0.347943000
H	-3.608792000	0.428849000	4.483629000
C	-1.480380000	0.422283000	4.828575000
H	0.649916000	0.402046000	4.873020000
C	1.985726000	-0.989845000	2.840004000
C	1.982641000	1.449629000	2.652109000
H	-1.498386000	4.951053000	0.332583000
C	-3.424252000	4.097373000	-0.181252000
C	-1.460229000	4.285317000	-1.732158000
H	-5.055699000	2.891591000	-0.944943000
C	-3.533617000	3.079384000	-2.476858000
H	-1.681085000	3.198300000	-3.595500000
H	-4.702732000	-3.468648000	0.487946000
C	-2.742576000	-4.386840000	0.398141000
C	-3.947288000	-3.339203000	-1.540022000
C	-1.588945000	-4.153152000	-1.819492000
H	-2.734383000	-3.086319000	-3.318886000
H	-0.668553000	-4.883092000	0.005954000
H	-1.560504000	0.516986000	5.906740000
H	2.995115000	-1.034464000	2.431239000
H	1.443329000	-1.897558000	2.572047000
H	2.058464000	-0.928322000	3.929292000
H	2.070223000	1.547620000	3.737533000
H	1.425800000	2.303856000	2.264630000
H	2.986557000	1.443867000	2.227479000
H	-3.835033000	5.042175000	-0.554978000
H	-3.746320000	4.005348000	0.863991000
H	-0.364965000	4.331634000	-1.806178000
H	-1.842256000	5.231038000	-2.133122000
H	-3.932302000	2.254913000	-3.081660000
H	-3.946552000	4.004627000	-2.894610000
H	-3.118900000	-5.377777000	0.119726000
H	-2.615957000	-4.391619000	1.488460000
H	-4.686738000	-2.588601000	-1.846736000
H	-4.344705000	-4.314557000	-1.843429000
H	-0.630000000	-3.990433000	-2.329971000
H	-1.944045000	-5.141848000	-2.131770000
C	3.503972000	0.161087000	-0.077308000
H	3.821804000	0.084879000	0.971622000
C	4.130598000	-0.973056000	-0.833870000
H	3.893773000	-0.948401000	-1.898381000
C	3.919976000	1.526780000	-0.642940000
H	3.529955000	1.644751000	-1.661349000
H	3.480699000	2.334752000	-0.049916000
C	4.030279000	-2.366283000	-0.269034000
H	4.335548000	-2.410891000	0.782066000
H	2.987405000	-2.689846000	-0.322844000
H	4.627436000	-3.074965000	-0.847519000
H	6.104532000	-0.871019000	-0.054055000
C	6.104598000	0.603565000	-1.468681000
H	7.195536000	0.600756000	-1.432643000
H	5.779288000	0.562136000	-2.509935000
C	5.444590000	1.705951000	-0.676420000
H	5.858860000	1.736307000	0.339887000
H	5.713790000	2.659157000	-1.145674000
O	5.699264000	-0.726767000	-0.928018000
H	1.439889000	-1.119667000	-3.827986000
H	1.491125000	0.881172000	-2.455657000

5v$_{O5}$

Sum of electronic and zero-point Energies = -1970.684649
Sum of electronic and thermal Free Energies = -1970.753744
Esolv = -1971.8753645

C	1.397992000	-0.105908000	-2.159926000
C	1.417012000	-0.908730000	-3.220051000
H	1.391984000	-1.992922000	-3.151496000
Au	1.258138000	-0.667198000	-0.229156000
P	-1.003813000	0.137459000	0.242379000
C	-1.089545000	-0.230051000	2.040692000
C	-1.126021000	2.028085000	0.061330000
C	-2.391434000	-0.872922000	-0.577405000
C	-2.230534000	0.081839000	2.803322000
C	-0.018518000	-0.867762000	2.686792000
C	0.232415000	2.594683000	0.545825000
C	-2.241553000	2.701732000	0.891722000
C	-1.326327000	2.394958000	-1.426976000
C	-3.818795000	-0.344027000	-0.319807000
C	-2.146884000	-0.932839000	-2.104266000
C	-2.279029000	-2.312564000	-0.018464000
H	-3.074149000	0.564331000	2.327430000
C	-2.309370000	-0.210587000	4.158434000
C	-0.102866000	-1.159410000	4.052838000
N	1.199888000	-1.284859000	1.964054000
H	0.383550000	2.344510000	1.604790000
H	1.057997000	2.139071000	-0.017129000
C	0.273373000	4.122298000	0.358858000
C	-2.202815000	4.230330000	0.689429000
H	-2.098637000	2.489098000	1.956101000
H	-3.227498000	2.318841000	0.604303000
C	-1.265464000	3.922861000	-1.608594000
H	-2.302422000	2.036417000	-1.772709000
H	-0.565871000	1.912419000	-2.051999000
H	-4.031441000	-0.305036000	0.753819000
H	-3.927397000	0.671864000	-0.716849000
C	-4.851034000	-1.269016000	-0.995386000
C	-3.189345000	-1.847226000	-2.773558000
H	-2.206761000	0.066371000	-2.546364000
H	-1.138879000	-1.307236000	-2.307672000
C	-3.317757000	-3.227367000	-0.693205000
H	-2.438974000	-2.315798000	1.066130000
H	-1.268621000	-2.704812000	-0.205525000
H	-3.204091000	0.045171000	4.717083000
C	-1.234992000	-0.831952000	4.787656000
H	0.718329000	-1.655633000	4.558897000
C	1.288427000	-2.772475000	1.999995000
C	2.400956000	-0.689350000	2.615772000

H	1.246780000	4.488654000	0.707110000
C	-0.851850000	4.772002000	1.174973000
C	0.092718000	4.453570000	-1.129374000
H	-3.013947000	4.672874000	1.280027000
C	-2.393179000	4.570616000	-0.794279000
H	-1.397720000	4.148457000	-2.673527000
H	-5.852720000	-0.871284000	-0.792704000
C	-4.727181000	-2.685280000	-0.416049000
C	-4.599519000	-1.303397000	-2.508584000
C	-3.066338000	-3.267235000	-2.206170000
H	-2.991776000	-1.859429000	-3.852183000
H	-3.217316000	-4.234434000	-0.270436000
H	-1.275994000	-1.068640000	5.846218000
H	2.166666000	-3.099407000	1.443247000
H	0.392399000	-3.195997000	1.544458000
H	1.371583000	-3.136138000	3.029146000
H	2.494323000	-1.020188000	3.654345000
H	2.315417000	0.398205000	2.595907000
H	3.298637000	-0.993191000	2.078272000
H	-0.818633000	5.862086000	1.059644000
H	-0.718293000	4.561319000	2.244067000
H	0.905613000	4.010380000	-1.720240000
H	0.147308000	5.538076000	-1.282397000
H	-3.369708000	4.213705000	-1.146703000
H	-2.384483000	5.658297000	-0.934256000
H	-5.477807000	-3.343487000	-0.869824000
H	-4.926079000	-2.674630000	0.663688000
H	-4.706842000	-0.297674000	-2.935585000
H	-5.347742000	-1.937240000	-2.999729000
H	-2.069361000	-3.676550000	-2.416412000
H	-3.790975000	-3.932988000	-2.690408000
C	3.223563000	-1.348182000	-0.505814000
H	3.299902000	-1.866642000	-1.463821000
H	3.380982000	-2.086692000	0.286358000
C	4.301142000	-0.274266000	-0.374908000
C	4.739914000	0.431594000	-1.659693000
H	3.995986000	0.487713000	0.364994000
C	6.644313000	-0.427809000	-0.535392000
C	6.192140000	0.789590000	-1.339955000
H	4.688880000	-0.263664000	-2.505268000
H	4.123283000	1.301165000	-1.901781000
H	7.399048000	-0.189176000	0.221407000
H	7.050836000	-1.212723000	-1.187411000
H	6.803126000	0.958856000	-2.230279000
H	6.236930000	1.695708000	-0.725468000
O	5.473824000	-0.927820000	0.122806000
H	1.454344000	-0.489195000	-4.223991000
H	1.453057000	0.975714000	-2.277141000

5v$_{O5Z}$

Sum of electronic and zero-point Energies = -2009.954436

Sum of electronic and thermal Free Energies = -2010.024851

Esolv = -2011.1833386

C	1.466105000	-0.438200000	-1.968806000
C	1.351140000	-1.487867000	-2.777711000
H	1.187313000	-2.502692000	-2.425557000
Au	1.283933000	-0.449101000	0.038214000
P	-1.098720000	0.127914000	0.215284000
C	-1.295204000	0.101369000	2.040930000
C	-1.515268000	1.902346000	-0.336194000
C	-2.228460000	-1.237662000	-0.472554000
C	-2.526488000	0.398225000	2.654768000
C	-0.199201000	-0.187730000	2.867870000
C	-0.798111000	2.865140000	0.641330000
C	-3.022838000	2.237815000	-0.355590000
C	-0.931660000	2.140891000	-1.749119000
C	-3.657583000	-1.270103000	0.112910000
C	-2.316479000	-1.110620000	-2.010700000
C	-1.538384000	-2.578515000	-0.117226000
H	-3.384901000	0.635100000	2.039762000
C	-2.677127000	0.397040000	4.034904000
C	-0.357401000	-0.193276000	4.258647000
N	1.142813000	-0.476866000	2.324006000
H	-1.177872000	2.729624000	1.660248000
H	0.278876000	2.642036000	0.660674000
C	-1.015848000	4.325853000	0.204012000
C	-3.230993000	3.702967000	-0.786938000
H	-3.465378000	2.096792000	0.636275000
H	-3.552916000	1.577079000	-1.050400000
C	-1.159323000	3.600009000	-2.184304000
H	-1.382719000	1.461659000	-2.479666000
H	0.143328000	1.933900000	-1.737934000
H	-3.617172000	-1.398712000	1.199559000
H	-4.185429000	-0.331981000	-0.092555000
C	-4.448985000	-2.447995000	-0.490772000
C	-3.101089000	-2.301471000	-2.592661000
H	-2.830162000	-0.181158000	-2.282529000
H	-1.312439000	-1.072995000	-2.447675000
C	-2.330984000	-3.759889000	-0.706306000
H	-1.470607000	-2.684658000	0.974302000
H	-0.514686000	-2.592061000	-0.514164000
H	-3.641168000	0.630622000	4.475489000
C	-1.584471000	0.093798000	4.841844000
H	0.484183000	-0.418805000	4.904971000
C	1.559025000	-1.838777000	2.761826000
C	2.098630000	0.548660000	2.832567000
H	-0.502207000	4.980195000	0.918789000
C	-2.519708000	4.635914000	0.202954000
C	-0.444671000	4.539219000	-1.203882000
H	-4.307643000	3.911419000	-0.785628000
C	-2.662337000	3.908525000	-2.196880000
H	-0.744786000	3.727276000	-3.191661000
H	-5.459829000	-2.433485000	-0.065649000
C	-3.752190000	-3.766104000	-0.128009000
C	-4.523111000	-2.303525000	-2.016458000
C	-2.394509000	-3.616121000	-2.233567000
H	-3.140665000	-2.187665000	-3.682642000
H	-1.815666000	-4.691014000	-0.440411000
H	-1.681557000	0.084188000	5.922933000
H	2.539098000	-2.074063000	2.347310000
H	0.829962000	-2.566449000	2.401884000
H	1.621033000	-1.904135000	3.852430000
H	2.181565000	0.505924000	3.923010000
H	1.743625000	1.538631000	2.542401000
H	3.087253000	0.380428000	2.404804000
H	-2.686686000	5.682124000	-0.080289000
H	-2.935460000	4.510402000	1.211327000
H	0.636429000	4.346033000	-1.210471000
H	-0.581659000	5.583068000	-1.511057000
H	-3.179502000	3.258605000	-2.914761000
H	-2.832481000	4.941066000	-2.524930000
H	-4.317737000	-4.615321000	-0.530229000
H	-3.720500000	-3.893079000	0.962067000
H	-5.044848000	-1.376311000	-2.287519000
H	-5.103827000	-3.129908000	-2.443986000
H	-1.383654000	-3.633500000	-2.660998000
H	-2.936516000	-4.464996000	-2.667699000
C	4.263774000	0.201222000	-0.428455000
H	3.792426000	1.165310000	-0.164052000
C	3.357901000	-0.942381000	0.048977000

C	4.713266000	0.255466000	-1.890550000
H	4.848931000	-0.753491000	-2.292389000
H	4.006093000	0.780953000	-2.535543000
C	6.613584000	0.392473000	-0.475053000
H	7.249881000	1.095074000	0.073413000
H	7.192460000	-0.523917000	-0.654275000
C	6.061722000	0.969713000	-1.776250000
H	5.914503000	2.051657000	-1.684664000
H	6.719140000	0.792873000	-2.631353000
O	5.474030000	0.074639000	0.332269000
C	3.698426000	-2.319384000	-0.493889000
H	4.765667000	-2.514433000	-0.331312000
H	3.142369000	-3.106262000	0.027503000
H	3.494646000	-2.418721000	-1.561632000
H	3.525040000	-0.968264000	1.130768000
H	1.429028000	-1.359836000	-3.856290000
H	1.649088000	0.554978000	-2.374372000

5v$_{O5E}$

Sum of electronic and zero-point Energies = -2009.947843
Sum of electronic and thermal Free Energies = -2010.017409
Esolv = -2011.1786404

C	1.127534000	-0.742443000	-2.143297000
C	1.103860000	-1.826498000	-2.915600000
H	1.224502000	-2.838138000	-2.537377000
Au	1.229329000	-0.782525000	-0.126471000
P	-1.025722000	0.163437000	0.243637000
C	-1.168818000	0.002182000	2.064045000
C	-1.098698000	2.041193000	-0.103504000
C	-2.427697000	-0.865230000	-0.524791000
C	-2.296369000	0.473893000	2.761763000
C	-0.115017000	-0.551467000	2.805441000
C	-0.224029000	2.746996000	0.961448000
C	-2.521011000	2.642275000	-0.061457000
C	-0.473231000	2.329956000	-1.488693000
C	-3.820616000	-0.668577000	0.114047000
C	-2.517152000	-0.563490000	-2.038587000
C	-2.022610000	-2.348120000	-0.332447000
H	-3.116824000	0.916672000	2.212069000
C	-2.388280000	0.393348000	4.144556000
C	-0.218240000	-0.641735000	4.198750000
N	1.095477000	-1.097266000	2.160369000
H	-0.625772000	2.572700000	1.965288000
H	0.794697000	2.332518000	0.942494000
C	-0.174483000	4.262629000	0.689908000
C	-2.462939000	4.159814000	-0.323405000
H	-2.985401000	2.470281000	0.915292000
H	-3.159058000	2.170466000	-0.816555000
C	-0.441294000	3.845451000	-1.760350000
H	-1.029018000	1.821446000	-2.284178000
H	0.554071000	1.948738000	-1.507145000
H	-3.784406000	-0.917235000	1.179990000
H	-4.149269000	0.372688000	0.025513000
C	-4.849911000	-1.590869000	-0.570463000
C	-3.543907000	-1.500335000	-2.702054000
H	-2.826647000	0.475638000	-2.200223000
H	-1.536943000	-0.695813000	-2.509374000
C	-3.053111000	-3.274942000	-1.003583000
H	-1.963685000	-2.579204000	0.740140000
H	-1.031503000	-2.527605000	-0.768261000
H	-3.270900000	0.768581000	4.652695000
C	-1.341644000	-0.172368000	4.866676000
H	0.580746000	-1.087154000	4.781285000
C	1.065016000	-2.578286000	2.333080000
C	2.311683000	-0.530586000	2.798736000
H	0.446699000	4.730750000	1.463159000
C	-1.598193000	4.834606000	0.750213000
C	0.427132000	4.528743000	-0.695980000
H	-3.485011000	4.554867000	-0.279643000
C	-1.864951000	4.415123000	-1.712718000
H	-0.008325000	4.007616000	-2.754907000
H	-5.827189000	-1.416259000	-0.104431000
C	-4.433414000	-3.054078000	-0.371058000
C	-4.923269000	-1.273203000	-2.069727000
C	-3.114861000	-2.961076000	-2.505116000
H	-3.581677000	-1.266192000	-3.772763000
H	-2.735102000	-4.313861000	-0.852812000
H	-1.395694000	-0.251111000	5.947969000
H	1.933071000	-3.020507000	1.849848000
H	0.156173000	-2.971532000	1.875331000
H	1.070390000	-2.846002000	3.395382000
H	2.369157000	-0.781593000	3.862169000
H	2.302157000	0.554918000	2.688823000
H	3.191373000	-0.936201000	2.304647000
H	-1.574321000	5.918814000	0.587199000
H	-2.031976000	4.673309000	1.745770000
H	1.457017000	4.150723000	-0.745041000
H	0.477206000	5.608124000	-0.883454000
H	-2.488942000	3.950344000	-2.487236000
H	-1.846906000	5.491012000	-1.924287000
H	-5.170077000	-3.722487000	-0.832963000
H	-4.407413000	-3.300545000	0.698535000
H	-5.247069000	-0.235657000	-2.225163000
H	-5.671082000	-1.913685000	-2.552879000
H	-2.136671000	-3.135773000	-2.971237000
H	-3.827661000	-3.632197000	-2.999408000
C	4.221001000	-0.203541000	-0.233153000
H	4.231438000	0.055725000	0.836823000
C	3.273708000	-1.378970000	-0.497407000
C	3.967323000	1.060122000	-1.065566000
H	3.520513000	0.788773000	-2.029321000
H	3.296009000	1.765949000	-0.566344000
C	6.171584000	0.317582000	-1.441224000
H	7.222947000	0.405969000	-1.150978000
H	6.131889000	-0.042066000	-2.480012000
C	5.379004000	1.608833000	-1.276328000
H	5.718062000	2.155167000	-0.389295000
H	5.462369000	2.275433000	-2.138735000
O	5.548847000	-0.629636000	-0.571659000
H	0.964338000	-1.727099000	-3.991125000
H	1.031082000	0.251211000	-2.572564000
C	3.724785000	-2.666183000	0.181252000
H	3.845734000	-2.583029000	1.266318000
H	3.058081000	-3.507349000	-0.035990000
H	4.714521000	-2.920846000	-0.212853000
H	3.296738000	-1.557894000	-1.573297000

5v$_{O5E'}$

Sum of electronic and zero-point Energies = -2009.957138
Sum of electronic and thermal Free Energies = -2010.026688
Esolv = -2011.1883365

C	1.389463000	-0.515966000	-2.061421000
C	1.147311000	-1.538083000	-2.877155000
H	0.898536000	-2.537434000	-2.531108000
Au	1.286671000	-0.546800000	-0.045473000
P	-1.053792000	0.155080000	0.231440000
C	-1.191433000	0.088446000	2.061408000
C	-1.391235000	1.960170000	-0.268035000

C	-2.282515000	-1.130362000	-0.443326000
C	-2.383792000	0.432829000	2.725197000
C	-0.090870000	-0.297215000	2.841374000
C	-0.587533000	2.859522000	0.702300000
C	-2.877416000	2.379177000	-0.226760000
C	-0.844964000	2.196622000	-1.696449000
C	-3.689430000	-1.096515000	0.194000000
C	-2.419474000	-0.961656000	-1.973932000
C	-1.658675000	-2.516249000	-0.142671000
H	-3.243742000	0.745705000	2.147433000
C	-2.494560000	0.381493000	4.108167000
C	-0.209743000	-0.355345000	4.234881000
N	1.221967000	-0.617376000	2.245773000
H	-0.938379000	2.721913000	1.731377000
H	0.475113000	2.576407000	0.677296000
C	-0.737659000	4.339442000	0.303250000
C	-3.017189000	3.862924000	-0.620436000
H	-3.293129000	2.240423000	0.777032000
H	-3.467445000	1.764849000	-0.915900000
C	-1.003890000	3.675700000	-2.092926000
H	-1.361116000	1.561298000	-2.423332000
H	0.215728000	1.926611000	-1.730195000
H	-3.618153000	-1.254511000	1.275118000
H	-4.169729000	-0.125402000	0.029729000
C	-4.568608000	-2.212488000	-0.405835000
C	-3.290755000	-2.092573000	-2.552269000
H	-2.890021000	0.001066000	-2.205156000
H	-1.431790000	-0.968394000	-2.447878000
C	-2.538714000	-3.636519000	-0.727689000
H	-1.558377000	-2.652190000	0.943026000
H	-0.652133000	-2.579088000	-0.576705000
H	-3.429295000	0.653784000	4.587929000
C	-1.400619000	-0.023452000	4.867282000
H	0.635342000	-0.655435000	4.845358000
C	1.623008000	-1.997106000	2.636074000
C	2.220009000	0.366988000	2.748648000
H	-0.163609000	4.948357000	1.012235000
C	-2.220836000	4.732569000	0.362277000
C	-0.204315000	4.551726000	-1.119643000
H	-4.079640000	4.131018000	-0.576623000
C	-2.486707000	4.067947000	-2.045306000
H	-0.617991000	3.802437000	-3.111647000
H	-5.561003000	-2.150458000	0.056853000
C	-3.936535000	-3.576135000	-0.097632000
C	-4.689078000	-2.027901000	-1.924136000
C	-2.648661000	-3.453289000	-2.248045000
H	-3.362468000	-1.950807000	-3.637311000
H	-2.068522000	-4.601340000	-0.501321000
H	-1.467719000	-0.075654000	5.949427000
H	2.586905000	-2.234003000	2.186676000
H	0.872248000	-2.702730000	2.276880000
H	1.714211000	-2.094658000	3.722216000
H	2.322263000	0.306575000	3.836802000
H	1.897758000	1.373297000	2.477169000
H	3.190754000	0.164101000	2.296925000
H	-2.338261000	5.792494000	0.106541000
H	-2.607898000	4.607808000	1.382093000
H	0.863340000	4.298952000	-1.168544000
H	-0.292660000	5.608112000	-1.400691000
H	-3.064260000	3.464219000	-2.757410000
H	-2.609323000	5.115475000	-2.345856000
H	-4.563751000	-4.382204000	-0.497160000
H	-3.873543000	-3.730630000	0.987531000
H	-5.166140000	-1.066486000	-2.155474000
H	-5.331259000	-2.809746000	-2.347506000
H	-1.656713000	-3.518524000	-2.713339000
H	-3.254105000	-4.259444000	-2.679735000
C	3.312213000	-1.203287000	-0.152808000
H	3.647294000	-1.073034000	0.884839000
C	4.233315000	-0.334728000	-1.004342000
H	4.004258000	-0.498292000	-2.068777000
C	5.663247000	1.557774000	-1.137747000
C	6.471723000	0.333112000	-0.709665000
H	5.701190000	1.683970000	-2.225110000
H	6.021316000	2.483697000	-0.680298000
H	6.858387000	0.443821000	0.312945000
H	7.320312000	0.127738000	-1.371036000
C	4.250941000	1.165041000	-0.702213000
H	4.123276000	1.336607000	0.374536000
H	3.466904000	1.717356000	-1.228369000
O	5.572073000	-0.778453000	-0.750798000
C	3.408795000	-2.675042000	-0.531225000
H	3.112243000	-2.840858000	-1.570169000
H	4.450595000	-2.999395000	-0.431344000
H	2.793813000	-3.320246000	0.105388000
H	1.202987000	-1.402634000	-3.956204000
H	1.657975000	0.460328000	-2.461577000

5v$_{O6}$
Sum of electronic and zero-point Energies = -1970.683071
Sum of electronic and thermal Free Energies = -1970.749742
Esolv = -1971.8789714

C	1.515455000	-0.033303000	-2.106431000
C	1.571458000	-1.152300000	-2.822913000
H	1.603075000	-2.145810000	-2.384674000
Au	1.443703000	0.116869000	-0.095750000
P	-0.987831000	0.000109000	0.226080000
C	-1.068578000	0.135338000	2.056125000
C	-1.927906000	1.502655000	-0.466501000
C	-1.707460000	-1.703270000	-0.219663000
C	-2.296848000	0.138287000	2.743335000
C	0.108217000	0.259111000	2.810180000
C	-1.455368000	2.741644000	0.332620000
C	-3.466456000	1.402913000	-0.374610000
C	-1.527906000	1.711082000	-1.946848000
C	-3.024611000	-2.069835000	0.499517000
C	-1.929150000	-1.789928000	-1.747178000
C	-0.634655000	-2.741266000	0.194676000
H	-3.220190000	0.055761000	2.184891000
C	-2.363145000	0.241958000	4.126304000
C	0.036940000	0.355856000	4.204595000
N	1.442417000	0.324047000	2.180370000
H	-1.713558000	2.635293000	1.392453000
H	-0.360706000	2.830077000	0.268130000
C	-2.109455000	4.017821000	-0.229101000
C	-4.111661000	2.686549000	-0.933317000
H	-3.785637000	1.272415000	0.664897000
H	-3.828719000	0.538582000	-0.942216000
C	-2.188912000	2.984773000	-2.504735000
H	-1.816680000	0.848411000	-2.555498000
H	-0.439796000	1.805914000	-2.022558000
H	-2.880110000	-2.051619000	1.584663000
H	-3.815111000	-1.350930000	0.255926000
C	-3.473896000	-3.486923000	0.089448000
C	-2.365227000	-3.215309000	-2.135628000
H	-2.711027000	-1.085409000	-2.053460000
H	-1.013197000	-1.516846000	-2.282490000
C	-1.083006000	-4.160314000	-0.201200000
H	-0.469916000	-2.694088000	1.279982000
H	0.321950000	-2.510941000	-0.293174000

H	-3.327413000	0.240747000	4.624512000
C	-1.186230000	0.344206000	4.861992000
H	0.942378000	0.448794000	4.794892000
C	2.295397000	-0.775731000	2.711700000
C	2.049785000	1.648294000	2.495209000
H	-1.760562000	4.873426000	0.361777000
C	-3.636083000	3.896861000	-0.117769000
C	-1.712160000	4.200791000	-1.699715000
H	-5.200621000	2.587432000	-0.850118000
C	-3.715153000	2.862098000	-2.404953000
H	-1.892319000	3.093942000	-3.554887000
H	-4.415369000	-3.709021000	0.606167000
C	-2.400387000	-4.500170000	0.508371000
C	-3.684576000	-3.552382000	-1.428850000
C	-1.283956000	-4.223370000	-1.721958000
H	-2.505735000	-3.247544000	-3.222702000
H	-0.302174000	-4.868571000	0.101763000
H	-1.215922000	0.421228000	5.944316000
H	3.273450000	-0.744095000	2.231989000
H	1.817514000	-1.732843000	2.497414000
H	2.438833000	-0.682555000	3.792485000
H	2.191676000	1.771714000	3.573439000
H	1.390984000	2.437938000	2.130948000
H	3.019937000	1.732667000	2.005592000
H	-4.112692000	4.811657000	-0.490023000
H	-3.934299000	3.789377000	0.933397000
H	-0.623545000	4.314398000	-1.789761000
H	-2.160246000	5.118650000	-2.099016000
H	-4.069300000	2.009787000	-2.999351000
H	-4.193327000	3.757738000	-2.819483000
H	-2.717257000	-5.516567000	0.245259000
H	-2.263291000	-4.482073000	1.597513000
H	-4.471097000	-2.850755000	-1.736197000
H	-4.023945000	-4.554452000	-1.717895000
H	-0.341670000	-4.006034000	-2.241394000
H	-1.581345000	-5.236376000	-2.018771000
C	4.221750000	-1.104928000	-0.547312000
H	4.001432000	-1.408160000	-1.582256000
H	3.876969000	-1.902181000	0.120150000
C	3.561385000	0.235741000	-0.235106000
C	4.128253000	1.320661000	-1.149042000
H	3.839350000	0.491703000	0.795670000
C	6.212782000	-0.079289000	-1.247925000
C	5.662351000	1.326622000	-1.035629000
H	3.850976000	1.115755000	-2.190307000
H	3.726104000	2.309968000	-0.898356000
H	7.289244000	-0.119352000	-1.061513000
H	6.036288000	-0.402161000	-2.288298000
H	6.097356000	2.015787000	-1.768982000
H	5.962564000	1.679970000	-0.041178000
O	5.621738000	-1.009888000	-0.351835000
H	1.597420000	-1.104716000	-3.910489000
H	1.517479000	0.940774000	-2.594145000

5v$_{O6Z}$

Sum of electronic and zero-point Energies = -2009.951628

Sum of electronic and thermal Free Energies = -2010.018848

Esolv = -2011.18628

C	-1.394777000	-0.160481000	-2.119101000
C	-1.341943000	0.907042000	-2.907947000
H	-1.297163000	1.927602000	-2.541682000
Au	-1.348120000	-0.260420000	-0.104883000
P	1.075493000	0.019290000	0.235502000
C	1.170764000	-0.190894000	2.058278000
C	2.147292000	-1.361464000	-0.519331000
C	1.649226000	1.799469000	-0.126265000
C	2.396755000	-0.115369000	2.745895000
C	0.012753000	-0.456055000	2.805116000
C	1.753918000	-2.677814000	0.194106000
C	3.670964000	-1.150640000	-0.377511000
C	1.801768000	-1.511478000	-2.020349000
C	2.900557000	2.258505000	0.655096000
C	1.920117000	1.967353000	-1.638789000
C	0.470091000	2.717212000	0.282792000
H	3.306038000	0.081281000	2.193570000
C	2.478961000	-0.281133000	4.121775000
C	0.100687000	-0.620536000	4.192557000
N	-1.309963000	-0.625494000	2.171366000
H	1.975943000	-2.613952000	1.265700000
H	0.671914000	-2.846539000	0.090677000
C	2.519406000	-3.865131000	-0.419234000
C	4.427203000	-2.345529000	-0.991587000
H	3.953934000	-1.061988000	0.676479000
H	3.979061000	-0.227762000	-0.881587000
C	2.570753000	-2.697911000	-2.629702000
H	2.043887000	-0.596858000	-2.569897000
H	0.725879000	-1.675370000	-2.137618000
H	2.722771000	2.182798000	1.732660000
H	3.763323000	1.626692000	0.413712000
C	3.228511000	3.726581000	0.315395000
C	2.229940000	3.441868000	-1.958940000
H	2.778799000	1.354240000	-1.935303000
H	1.059421000	1.625404000	-2.223195000
C	0.796595000	4.186257000	-0.042560000
H	0.269363000	2.610147000	1.357814000
H	-0.442916000	2.419892000	-0.250761000
H	3.441276000	-0.214957000	4.619366000
C	1.320039000	-0.530143000	4.850541000
H	-0.787917000	-0.832595000	4.777264000
C	-2.297758000	0.285943000	2.808281000
C	-1.733683000	-2.042506000	2.359982000
H	2.224690000	-4.777974000	0.112606000
C	4.028890000	-3.634286000	-0.259019000
C	2.171587000	-3.991654000	-1.908275000
H	5.502874000	-2.168272000	-0.872604000
C	4.080099000	-2.465348000	-2.481160000
H	2.307861000	-2.767851000	-3.692106000
H	4.126006000	4.013810000	0.876391000
C	2.050370000	4.618140000	0.729483000
C	3.485613000	3.873097000	-1.189927000
C	1.044484000	4.327813000	-1.551026000
H	2.404893000	3.530110000	-3.037900000
H	-0.057486000	4.806699000	0.255239000
H	1.361183000	-0.661381000	5.927272000
H	-3.274373000	0.134252000	2.350841000
H	-1.980499000	1.319541000	2.661355000
H	-2.393011000	0.091105000	3.880408000
H	-1.835246000	-2.283324000	3.423194000
H	-0.986034000	-2.701853000	1.917052000
H	-2.695193000	-2.206653000	1.872699000
H	4.583875000	-4.486627000	-0.669031000
H	4.293376000	-3.565863000	0.804388000
H	1.097411000	-4.182352000	-2.034315000
H	2.698609000	-4.847879000	-2.346315000
H	4.380464000	-1.555430000	-3.016891000
H	4.636426000	-3.296231000	-2.931585000
H	2.279149000	5.669338000	0.516551000
H	1.877328000	4.543393000	1.811041000
H	4.345679000	3.261405000	-1.492277000
H	3.737691000	4.913469000	-1.428540000

H	0.145029000	4.046120000	-2.114158000
H	1.255387000	5.374900000	-1.799723000
C	-3.466466000	-0.489980000	-0.230864000
H	-3.648862000	-1.024943000	0.711286000
C	-4.276672000	0.816210000	-0.112073000
C	-4.017525000	-1.380978000	-1.342234000
H	-3.895552000	-0.903448000	-2.320595000
H	-3.476028000	-2.333903000	-1.386575000
C	-6.243909000	-0.323853000	-0.864316000
H	-7.271088000	-0.492170000	-0.529540000
H	-6.293264000	0.245798000	-1.804640000
C	-5.509893000	-1.640840000	-1.081664000
H	-5.625166000	-2.266188000	-0.187130000
H	-5.957562000	-2.188533000	-1.919283000
O	-5.632607000	0.451238000	0.159836000
C	-4.174584000	1.813875000	-1.261504000
H	-4.944210000	2.579436000	-1.127951000
H	-3.199742000	2.307535000	-1.249492000
H	-4.301690000	1.362611000	-2.247736000
H	-3.975186000	1.348230000	0.797198000
H	-1.357748000	0.785489000	-3.989993000
H	-1.474693000	-1.160021000	-2.546883000

5v$_{O6E}$

Sum of electronic and zero-point Energies = -2009.961796
Sum of electronic and thermal Free Energies = -2010.029716
Esolv = -2011.195282

C	-1.461607000	-0.020344000	-2.076796000
C	-1.445482000	1.062587000	-2.848106000
H	-1.411245000	2.076912000	-2.460939000
Au	-1.373583000	-0.087948000	-0.061066000
P	1.068709000	-0.015358000	0.223364000
C	1.177563000	-0.145085000	2.052633000
C	1.964548000	-1.545966000	-0.471176000
C	1.827560000	1.665367000	-0.243477000
C	2.417028000	-0.186144000	2.718421000
C	0.011322000	-0.234110000	2.827193000
C	1.465959000	-2.768057000	0.338184000
C	3.506094000	-1.486078000	-0.392226000
C	1.549672000	-1.751947000	-1.947590000
C	3.160987000	2.002506000	0.459787000
C	2.035797000	1.735802000	-1.773672000
C	0.786544000	2.733777000	0.173619000
H	3.332376000	-0.126616000	2.144384000
C	2.504974000	-0.299635000	4.099289000
C	0.104647000	-0.340434000	4.219800000
N	-1.336517000	-0.230258000	2.224179000
H	1.734862000	-2.663084000	1.395436000
H	0.368897000	-2.828447000	0.282813000
C	2.082571000	-4.063739000	-0.221182000
C	4.113636000	-2.789279000	-0.947995000
H	3.837893000	-1.358457000	0.643428000
H	3.885836000	-0.634804000	-0.968079000
C	2.172544000	-3.045753000	-2.503031000
H	1.857369000	-0.900815000	-2.562984000
H	0.459479000	-1.817439000	-2.016675000
H	3.027268000	1.994675000	1.546481000
H	3.930031000	1.261763000	0.213022000
C	3.642623000	3.404622000	0.035255000
C	2.504136000	3.146817000	-2.176732000
H	2.796225000	1.009416000	-2.082833000
H	1.108127000	1.481215000	-2.297996000
C	1.267240000	4.138081000	-0.235962000
H	0.630923000	2.697687000	1.260712000
H	-0.180510000	2.524935000	-0.303248000
H	3.477631000	-0.329229000	4.580015000
C	1.338793000	-0.372217000	4.855322000
H	-0.792632000	-0.403404000	4.826277000
C	-2.098483000	0.937037000	2.747960000
C	-2.029337000	-1.499540000	2.582656000
H	1.716083000	-4.906820000	0.377023000
C	3.612660000	-3.981961000	-0.121739000
C	1.669911000	-4.244385000	-1.687810000
H	5.205379000	-2.717919000	-0.872948000
C	3.702099000	-2.963147000	-2.415563000
H	1.865003000	-3.152567000	-3.550297000
H	4.594909000	3.605753000	0.540622000
C	2.600189000	4.448224000	0.457939000
C	3.839007000	3.454307000	-1.485650000
C	1.453873000	4.185568000	-1.759120000
H	2.634323000	3.168179000	-3.265359000
H	0.508215000	4.868426000	0.069999000
H	1.385194000	-0.456778000	5.936499000
H	-3.090445000	0.954971000	2.298281000
H	-1.569882000	1.854985000	2.487357000
H	-2.209072000	0.883319000	3.835485000
H	-2.160142000	-1.589561000	3.665471000
H	-1.436477000	-2.342797000	2.225299000
H	-3.012414000	-1.522314000	2.112964000
H	4.062531000	-4.911027000	-0.491974000
H	3.921334000	-3.876155000	0.926580000
H	0.578046000	-4.329728000	-1.769379000
H	2.090772000	-5.175925000	-2.085121000
H	4.074107000	-2.123968000	-3.017701000
H	4.153426000	-3.873558000	-2.828067000
H	2.940600000	5.454279000	0.184674000
H	2.473808000	4.440887000	1.548510000
H	4.603851000	2.730434000	-1.796066000
H	4.201184000	4.445235000	-1.785243000
H	0.500625000	3.989601000	-2.267177000
H	1.774307000	5.188402000	-2.066308000
C	-3.496326000	-0.245757000	-0.180492000
H	-3.788636000	-0.202097000	0.877807000
C	-4.254878000	0.895466000	-0.870263000
H	-4.046398000	0.864759000	-1.950617000
C	-3.936081000	-1.596089000	-0.750722000
H	-3.630795000	-1.670248000	-1.802552000
H	-3.457712000	-2.431899000	-0.224595000
C	-3.953120000	2.282763000	-0.334022000
H	-4.210586000	2.350466000	0.727835000
H	-2.895340000	2.535381000	-0.457544000
H	-4.551797000	3.026216000	-0.866020000
C	-6.124187000	-0.499533000	-1.277607000
H	-7.207172000	-0.509392000	-1.127196000
H	-5.933414000	-0.463305000	-2.363880000
C	-5.464864000	-1.730181000	-0.670099000
H	-5.777954000	-1.821051000	0.377553000
H	-5.797471000	-2.636850000	-1.188996000
O	-5.650148000	0.688533000	-0.665202000
H	-1.483583000	0.962411000	-3.931774000
H	-1.533399000	-1.013472000	-2.519018000

3$_v^B$

Sum of electronic and zero-point Energies = -1970.947151
Sum of electronic and thermal Free Energies = -1971.016152
Esolv = -1972.2901652

Au	1.244682000	-0.527150000	-0.055453000
C	1.586941000	1.079741000	-2.600313000

H	1.687628000	2.012582000	-2.053775000
P	-1.060989000	0.072065000	0.213762000
C	-1.237058000	-0.321699000	1.997038000
C	-1.320037000	1.952798000	0.015200000
C	-2.217322000	-1.095772000	-0.748126000
C	-2.467883000	-0.127631000	2.652763000
C	-0.164060000	-0.828552000	2.741963000
C	-0.039052000	2.640885000	0.545202000
C	-2.517827000	2.477299000	0.845744000
C	-1.549011000	2.322994000	-1.467403000
C	-3.705014000	-0.706517000	-0.566699000
C	-1.875814000	-1.093405000	-2.254953000
C	-1.992894000	-2.523714000	-0.197317000
H	-3.318539000	0.254041000	2.104870000
C	-2.623015000	-0.406670000	4.004460000
C	-0.322523000	-1.101487000	4.103642000
N	1.131133000	-1.154294000	2.108470000
H	0.109868000	2.389816000	1.604555000
H	0.842117000	2.283914000	-0.005556000
C	-0.159203000	4.169636000	0.385208000
C	-2.636066000	4.007920000	0.677855000
H	-2.371299000	2.258290000	1.907469000
H	-3.452314000	2.002740000	0.525398000
C	-1.647317000	3.855348000	-1.609431000
H	-2.482444000	1.874254000	-1.824734000
H	-0.740388000	1.938829000	-2.095870000
H	-3.985677000	-0.721372000	0.490746000
H	-3.890549000	0.302150000	-0.952017000
C	-4.595192000	-1.716763000	-1.321734000
C	-2.783322000	-2.092197000	-3.000116000
H	-1.993348000	-0.094758000	-2.685407000
H	-0.832423000	-1.388915000	-2.397402000
C	-2.897227000	-3.520764000	-0.950598000
H	-2.217312000	-2.564177000	0.875010000
H	-0.939655000	-2.814538000	-0.331871000
H	-3.584113000	-0.245292000	4.481751000
C	-1.541306000	-0.886930000	4.735900000
H	0.499765000	-1.498949000	4.688921000
C	1.307188000	-2.638334000	2.171531000
C	2.241609000	-0.476880000	2.835332000
H	0.764928000	4.628482000	0.756605000
C	-1.357440000	4.675150000	1.199475000
C	-0.358055000	4.513417000	-1.097951000
H	-3.496990000	4.339688000	1.269449000
C	-2.846157000	4.362220000	-0.798738000
H	-1.787964000	4.088395000	-2.671016000
H	-5.639009000	-1.418339000	-1.171417000
C	-4.366903000	-3.123233000	-0.752467000
C	-4.252241000	-1.691285000	-2.815820000
C	-2.552141000	-3.503610000	-2.445415000
H	-2.518237000	-2.060238000	-4.063187000
H	-2.721911000	-4.520047000	-0.535609000
H	-1.643498000	-1.105148000	5.794081000
H	2.246752000	-2.928840000	1.701868000
H	0.478575000	-3.120321000	1.652017000
H	1.321696000	-2.980549000	3.209995000
H	2.284158000	-0.788282000	3.882083000
H	2.096959000	0.603826000	2.792242000
H	3.190210000	-0.744438000	2.369475000
H	-1.437730000	5.764283000	1.109472000
H	-1.216904000	4.455816000	2.265684000
H	0.504477000	4.180312000	-1.690814000
H	-0.418065000	5.600443000	-1.222908000
H	-3.776379000	3.916658000	-1.173187000
H	-2.949219000	5.447293000	-0.912706000
H	-5.018834000	-3.842920000	-1.260096000
H	-4.632722000	-3.154258000	0.312007000
H	-4.433511000	-0.693100000	-3.234314000
H	-4.902292000	-2.384060000	-3.361920000
H	-1.509579000	-3.811553000	-2.602347000
H	-3.176560000	-4.226647000	-2.982039000
C	3.364658000	-1.282005000	-0.326309000
H	3.328796000	-1.814853000	-1.274717000
H	3.414753000	-1.937711000	0.539650000
C	4.217997000	-0.167617000	-0.271182000
C	4.682665000	0.619794000	-1.437257000
H	4.420230000	0.271793000	0.705773000
C	6.935309000	-0.375653000	-1.183985000
C	6.186470000	0.937738000	-1.350788000
H	4.442432000	0.104994000	-2.370780000
H	4.127143000	1.570432000	-1.434925000
H	7.998029000	-0.217906000	-0.974802000
H	6.853555000	-0.995571000	-2.080938000
H	6.506993000	1.468412000	-2.252218000
H	6.386267000	1.598927000	-0.498661000
H	6.753124000	-0.895502000	0.707558000
O	6.312914000	-1.114833000	-0.124464000
C	1.343779000	-0.095289000	-2.033120000
H	1.246699000	-1.003182000	-2.627257000
H	1.690767000	1.144121000	-3.682979000

TS1v$^{B}_{OH5}$

Sum of electronic and zero-point Energies = -1970.945334
Sum of electronic and thermal Free Energies = -1971.012755
Esolv = -1972.2877283

Au	1.264134000	-0.555167000	-0.069447000
C	1.589036000	1.043690000	-2.625453000
H	1.689210000	1.979625000	-2.084145000
P	-1.043474000	0.076011000	0.216070000
C	-1.229812000	-0.328469000	1.996878000
C	-1.292950000	1.959545000	0.039002000
C	-2.233457000	-1.058275000	-0.745224000
C	-2.455500000	-0.127055000	2.659464000
C	-0.162419000	-0.858406000	2.733740000
C	-0.000556000	2.629833000	0.564984000
C	-2.479217000	2.498141000	0.874800000
C	-1.520045000	2.342851000	-1.441135000
C	-3.715144000	-0.650016000	-0.566406000
C	-1.888591000	-1.054459000	-2.251960000
C	-2.033557000	-2.494169000	-0.204411000
H	-3.301996000	0.272206000	2.117671000
C	-2.611913000	-0.421389000	4.007751000
C	-0.321250000	-1.148407000	4.092140000
N	1.130078000	-1.188935000	2.095456000
H	0.151741000	2.369915000	1.621863000
H	0.872496000	2.263867000	0.006636000
C	-0.100352000	4.160655000	0.415178000
C	-2.578404000	4.030360000	0.716227000
H	-2.332955000	2.270304000	1.934927000
H	-3.421052000	2.037382000	0.555624000
C	-1.598929000	3.876511000	-1.575729000
H	-2.459644000	1.907982000	-1.799347000
H	-0.718037000	1.950452000	-2.073363000
H	-3.998331000	-0.665530000	0.490534000
H	-3.884706000	0.363890000	-0.945380000
C	-4.619519000	-1.640876000	-1.328928000
C	-2.808946000	-2.034439000	-3.005526000
H	-1.990653000	-0.051884000	-2.677300000
H	-0.848275000	-1.362407000	-2.393683000
C	-2.950446000	-3.473211000	-0.965105000

H	-2.260772000	-2.537876000	0.867225000
H	-0.984398000	-2.800041000	-0.337556000
H	-3.569670000	-0.253463000	4.489514000
C	-1.535558000	-0.926660000	4.730242000
H	0.497008000	-1.564749000	4.670038000
C	1.295732000	-2.673571000	2.141644000
C	2.243723000	-0.527482000	2.828683000
H	0.830938000	4.605973000	0.785752000
C	-1.288915000	4.678017000	1.236289000
C	-0.299751000	4.515601000	-1.065284000
H	-3.432449000	4.372583000	1.311818000
C	-2.788102000	4.395402000	-0.758118000
H	-1.740257000	4.117704000	-2.635506000
H	-5.659573000	-1.328634000	-1.180491000
C	-4.414499000	-3.054655000	-0.768587000
C	-4.272194000	-1.612305000	-2.822203000
C	-2.601573000	-3.452720000	-2.459016000
H	-2.541477000	-2.000797000	-4.068072000
H	-2.791863000	-4.477804000	-0.555934000
H	-1.638600000	-1.158358000	5.785476000
H	2.228922000	-2.963624000	1.658809000
H	0.459657000	-3.143202000	1.622734000
H	1.315521000	-3.028736000	3.175862000
H	2.285259000	-0.848649000	3.872741000
H	2.106975000	0.554646000	2.795664000
H	3.189113000	-0.797647000	2.357496000
H	-1.354766000	5.768728000	1.152520000
H	-1.147432000	4.450624000	2.300744000
H	0.556400000	4.173807000	-1.662728000
H	-0.346075000	5.604066000	-1.184414000
H	-3.725239000	3.963751000	-1.131698000
H	-2.877231000	5.482380000	-0.866509000
H	-5.076124000	-3.761090000	-1.282472000
H	-4.683287000	-3.088194000	0.295109000
H	-4.436613000	-0.608772000	-3.234962000
H	-4.931539000	-2.291606000	-3.374274000
H	-1.563293000	-3.775562000	-2.614918000
H	-3.235814000	-4.162783000	-3.001647000
C	3.324691000	-1.268394000	-0.325618000
H	3.338339000	-1.792950000	-1.281598000
H	3.430725000	-1.976740000	0.496987000
C	4.318926000	-0.216564000	-0.252256000
C	4.683034000	0.665083000	-1.409031000
H	4.443894000	0.259144000	0.723016000
C	6.831869000	-0.407608000	-1.153689000
C	6.188275000	0.951038000	-1.359198000
H	4.401198000	0.189837000	-2.351784000
H	4.111842000	1.597031000	-1.326982000
H	7.845281000	-0.373116000	-0.748909000
H	6.822951000	-1.018160000	-2.058130000
H	6.537817000	1.424402000	-2.280111000
H	6.432212000	1.624057000	-0.529417000
H	6.340426000	-1.055137000	0.690054000
O	5.963859000	-1.107125000	-0.201960000
C	1.372524000	-0.131260000	-2.046980000
H	1.285397000	-1.042825000	-2.637316000
H	1.665599000	1.105528000	-3.710385000

TS1v$^{B}_{OH6}$

Sum of electronic and zero-point Energies = -1970.932722

Sum of electronic and thermal Free Energies = -1970.999744

Esolv = -1972.2803178

C	-1.388037000	0.538906000	-1.916196000
C	-1.591280000	-0.251543000	-2.963006000
H	-1.716152000	-1.328650000	-2.904048000
Au	-1.376168000	0.012298000	0.051111000
P	1.021211000	0.020948000	0.211500000
C	1.171149000	-0.151698000	2.029830000
C	1.820857000	-1.535032000	-0.548685000
C	1.717162000	1.724930000	-0.291059000
C	2.439340000	-0.157172000	2.641652000
C	0.044526000	-0.343462000	2.839664000
C	1.312236000	-2.755867000	0.253540000
C	3.365482000	-1.476988000	-0.469186000
C	1.394731000	-1.712012000	-2.021666000
C	3.076398000	2.039727000	0.382159000
C	1.894732000	1.798636000	-1.824380000
C	0.701632000	2.793398000	0.183024000
H	3.329836000	-0.024109000	2.042581000
C	2.583330000	-0.330366000	4.012172000
C	0.194051000	-0.517680000	4.218499000
N	-1.320919000	-0.398043000	2.272548000
H	1.583552000	-2.667013000	1.311501000
H	0.214450000	-2.809956000	0.190744000
C	1.920578000	-4.049834000	-0.324893000
C	3.957146000	-2.781660000	-1.044561000
H	3.700694000	-1.369445000	0.567037000
H	3.748566000	-0.623364000	-1.038784000
C	2.009449000	-3.004469000	-2.593919000
H	1.701590000	-0.855108000	-2.628723000
H	0.304840000	-1.775548000	-2.079729000
H	2.970952000	2.032771000	1.471150000
H	3.832078000	1.295884000	0.107047000
C	3.556101000	3.443690000	-0.048350000
C	2.353117000	3.214900000	-2.225538000
H	2.651192000	1.075402000	-2.148531000
H	0.964317000	1.545486000	-2.342366000
C	1.181752000	4.197218000	-0.237281000
H	0.594475000	2.748518000	1.275555000
H	-0.289226000	2.610131000	-0.253127000
H	3.573872000	-0.327249000	4.455323000
C	1.453880000	-0.507664000	4.805130000
H	-0.670555000	-0.670530000	4.855554000
C	-2.156578000	0.650773000	2.928105000
C	-1.888679000	-1.755393000	2.533556000
H	1.548989000	-4.894533000	0.266773000
C	3.451059000	-3.977677000	-0.227514000
C	1.500458000	-4.208802000	-1.791550000
H	5.048483000	-2.713921000	-0.970338000
C	3.539421000	-2.931507000	-2.512237000
H	1.695980000	-3.092959000	-3.640570000
H	4.523598000	3.624147000	0.433870000
C	2.537655000	4.491403000	0.416949000
C	3.709750000	3.506713000	-1.572468000
C	1.316838000	4.249900000	-1.765853000
H	2.449301000	3.243482000	-3.316826000
H	0.437437000	4.928644000	0.098927000
H	1.548059000	-0.644082000	5.877660000
H	-3.169541000	0.623013000	2.526128000
H	-1.712720000	1.630671000	2.745374000
H	-2.221301000	0.488073000	4.006897000
H	-1.972789000	-1.942762000	3.607518000
H	-1.233366000	-2.507516000	2.093626000
H	-2.881416000	-1.838081000	2.091474000
H	3.892633000	-4.905245000	-0.608662000
H	3.764935000	-3.887113000	0.820311000
H	0.407773000	-4.288812000	-1.871100000
H	1.913382000	-5.137469000	-2.201127000
H	3.916100000	-2.089018000	-3.106091000
H	3.982318000	-3.838948000	-2.937809000

H	2.876942000	5.495568000	0.139101000
H	2.446488000	4.480294000	1.510621000
H	4.461566000	2.784353000	-1.914705000
H	4.068020000	4.498246000	-1.871388000
H	0.346207000	4.060273000	-2.244624000
H	1.626276000	5.253489000	-2.078252000
C	-3.837362000	1.132168000	-0.304268000
H	-3.763609000	1.482375000	-1.327995000
H	-3.807814000	1.899304000	0.465794000
C	-3.687884000	-0.220367000	-0.004593000
C	-4.219977000	-1.241894000	-0.989118000
H	-3.871453000	-0.479247000	1.037859000
C	-6.307858000	0.168213000	-1.517853000
C	-5.759568000	-1.170495000	-1.028448000
H	-3.829270000	-1.045194000	-1.991988000
H	-3.908126000	-2.251612000	-0.704172000
H	-7.397625000	0.131945000	-1.616150000
H	-5.899741000	0.423135000	-2.500389000
H	-6.128735000	-1.947885000	-1.706834000
H	-6.177889000	-1.407328000	-0.041207000
H	-6.482851000	1.234537000	0.142651000
O	-5.957201000	1.283213000	-0.668381000
H	-1.627562000	0.180486000	-3.962756000
H	-1.253631000	1.613297000	-2.033769000

$4v^{B}_{OH5}$

Sum of electronic and zero-point Energies = -1970.944626
Sum of electronic and thermal Free Energies = -1971.012496
Esolv = -1972.2886207

Au	1.264869000	-0.590568000	-0.090426000
C	1.584236000	1.003841000	-2.652321000
H	1.696643000	1.940156000	-2.114237000
P	-1.033783000	0.082405000	0.219626000
C	-1.218255000	-0.332733000	1.998797000
C	-1.255160000	1.971207000	0.062855000
C	-2.259555000	-1.015511000	-0.739191000
C	-2.434313000	-0.115066000	2.673670000
C	-0.155813000	-0.890469000	2.722421000
C	0.054086000	2.614054000	0.581968000
C	-2.423851000	2.527622000	0.910742000
C	-1.485067000	2.370331000	-1.412838000
C	-3.731545000	-0.580947000	-0.547469000
C	-1.924809000	-1.006250000	-2.248553000
C	-2.084999000	-2.460356000	-0.213197000
H	-3.276926000	0.305827000	2.142299000
C	-2.586513000	-0.420547000	4.019967000
C	-0.310111000	-1.192723000	4.078793000
N	1.126469000	-1.237884000	2.071831000
H	0.211065000	2.343172000	1.635456000
H	0.915032000	2.235584000	0.012869000
C	-0.017323000	4.147445000	0.444566000
C	-2.495213000	4.062236000	0.763788000
H	-2.274414000	2.289130000	1.968180000
H	-3.376766000	2.087101000	0.595890000
C	-1.535600000	3.905888000	-1.536706000
H	-2.435174000	1.956062000	-1.767667000
H	-0.695286000	1.966588000	-2.053470000
H	-4.007831000	-0.599419000	0.511307000
H	-3.883631000	0.439395000	-0.916417000
C	-4.660711000	-1.546638000	-1.311966000
C	-2.869177000	-1.961201000	-3.004298000
H	-2.010516000	0.001621000	-2.664901000
H	-0.891128000	-1.332001000	-2.400274000
C	-3.025853000	-3.414708000	-0.975616000
H	-2.305381000	-2.509323000	0.859669000
H	-1.042980000	-2.785408000	-0.356390000
H	-3.537168000	-0.239280000	4.510900000
C	-1.515019000	-0.954794000	4.728687000
H	0.504146000	-1.631340000	4.645827000
C	1.264791000	-2.725196000	2.098332000
C	2.256415000	-0.607313000	2.805553000
H	0.924998000	4.572577000	0.811239000
C	-1.189598000	4.681574000	1.278314000
C	-0.220901000	4.516941000	-1.031741000
H	-3.337921000	4.417426000	1.367883000
C	-2.708505000	4.441753000	-0.706513000
H	-1.680254000	4.157474000	-2.593662000
H	-5.693632000	-1.215768000	-1.154253000
C	-4.480070000	-2.969278000	-0.765688000
C	-4.322643000	-1.512048000	-2.807328000
C	-2.686620000	-3.388139000	-2.471652000
H	-2.608486000	-1.923749000	-4.068454000
H	-2.884562000	-4.425878000	-0.576281000
H	-1.614747000	-1.196348000	5.782032000
H	2.188304000	-3.024817000	1.602650000
H	0.416057000	-3.172561000	1.580306000
H	1.286382000	-3.094417000	3.127708000
H	2.297706000	-0.939607000	3.846226000
H	2.142173000	0.477701000	2.783980000
H	3.192780000	-0.892311000	2.325044000
H	-1.235083000	5.773981000	1.202835000
H	-1.044432000	4.443604000	2.339982000
H	0.624178000	4.162897000	-1.637917000
H	-0.247470000	5.606946000	-1.143078000
H	-3.656390000	4.030535000	-1.076036000
H	-2.777649000	5.531006000	-0.806868000
H	-5.158938000	-3.657958000	-1.281283000
H	-4.742345000	-3.006899000	0.299520000
H	-4.469704000	-0.501786000	-3.210171000
H	-4.998972000	-2.173255000	-3.360927000
H	-1.655913000	-3.729955000	-2.637232000
H	-3.338454000	-4.080694000	-3.016167000
C	3.293831000	-1.308361000	-0.361904000
H	3.321695000	-1.801230000	-1.335590000
H	3.413066000	-2.064470000	0.418591000
C	4.363773000	-0.295180000	-0.254967000
C	4.663037000	0.683586000	-1.364475000
H	4.461120000	0.161281000	0.734340000
C	6.806742000	-0.375255000	-1.128347000
C	6.163002000	0.986773000	-1.295236000
H	4.385770000	0.255882000	-2.331331000
H	4.066154000	1.588228000	-1.220714000
H	7.758041000	-0.382762000	-0.594752000
H	6.894128000	-0.932941000	-2.061261000
H	6.523349000	1.484086000	-2.198763000
H	6.395943000	1.633908000	-0.443110000
H	6.159317000	-1.345952000	0.568532000
O	5.818521000	-1.141560000	-0.316984000
C	1.369884000	-0.168635000	-2.067600000
H	1.273550000	-1.080358000	-2.656437000
H	1.643733000	1.063347000	-3.738380000

$5v^{B}_{O5}$

Sum of electronic and zero-point Energies = -1970.944626
Sum of electronic and thermal Free Energies = -1971.012496
Esolv = -1971.85985871

Au	1.264869000	-0.590568000	-0.090426000
C	1.584236000	1.003841000	-2.652321000

H	1.696643000	1.940156000	-2.114237000
P	-1.033783000	0.082405000	0.219626000
C	-1.218255000	-0.332733000	1.998797000
C	-1.255160000	1.971207000	0.062855000
C	-2.259555000	-1.015511000	-0.739191000
C	-2.434313000	-0.115066000	2.673670000
C	-0.155813000	-0.890469000	2.722421000
C	0.054086000	2.614054000	0.581968000
C	-2.423851000	2.527622000	0.910742000
C	-1.485067000	2.370331000	-1.412838000
C	-3.731545000	-0.580947000	-0.547469000
C	-1.924809000	-1.006250000	-2.248553000
C	-2.084999000	-2.460356000	-0.213197000
H	-3.276926000	0.305827000	2.142299000
C	-2.586513000	-0.420547000	4.019967000
C	-0.310111000	-1.192723000	4.078793000
N	1.126469000	-1.237884000	2.071831000
H	0.211065000	2.343172000	1.635456000
H	0.915032000	2.235584000	0.012869000
C	-0.017323000	4.147445000	0.444566000
C	-2.495213000	4.062236000	0.763788000
H	-2.274414000	2.289130000	1.968180000
H	-3.376766000	2.087101000	0.595890000
C	-1.535600000	3.905888000	-1.536706000
H	-2.435174000	1.956062000	-1.767667000
H	-0.695286000	1.966588000	-2.053470000
H	-4.007831000	-0.599419000	0.511307000
H	-3.883631000	0.439395000	-0.916417000
C	-4.660711000	-1.546638000	-1.311966000
C	-2.869177000	-1.961201000	-3.004298000
H	-2.010516000	0.001621000	-2.664901000
H	-0.891128000	-1.332001000	-2.400274000
C	-3.025853000	-3.414708000	-0.975616000
H	-2.305381000	-2.509323000	0.859669000
H	-1.042980000	-2.785408000	-0.356390000
H	-3.537168000	-0.239280000	4.510900000
C	-1.515019000	-0.954794000	4.728687000
H	0.504146000	-1.631340000	4.645827000
C	1.264791000	-2.725196000	2.098332000
C	2.256415000	-0.607313000	2.805553000
H	0.924998000	4.572577000	0.811239000
C	-1.189598000	4.681574000	1.278314000
C	-0.220901000	4.516941000	-1.031741000
H	-3.337921000	4.417426000	1.367883000
C	-2.708505000	4.441753000	-0.706513000
H	-1.680254000	4.157474000	-2.593662000
H	-5.693632000	-1.215768000	-1.154253000
C	-4.480070000	-2.969278000	-0.765688000
C	-4.322643000	-1.512048000	-2.807328000
C	-2.686620000	-3.388139000	-2.471652000
H	-2.608486000	-1.923749000	-4.068454000
H	-2.884562000	-4.425878000	-0.576281000
H	-1.614747000	-1.196348000	5.782032000
H	2.188304000	-3.024817000	1.602650000
H	0.416057000	-3.172561000	1.580306000
H	1.286382000	-3.094417000	3.127708000
H	2.297706000	-0.939607000	3.846226000
H	2.142173000	0.477701000	2.783980000
H	3.192780000	-0.892311000	2.325044000
H	-1.235083000	5.773981000	1.202835000
H	-1.044432000	4.443604000	2.339982000
H	0.624178000	4.162897000	-1.637917000
H	-0.247470000	5.606946000	-1.143078000
H	-3.656390000	4.030535000	-1.076036000
H	-2.777649000	5.531006000	-0.806868000
H	-5.158938000	-3.657958000	-1.281283000
H	-4.742345000	-3.006899000	0.299520000
H	-4.469704000	-0.501786000	-3.210171000
H	-4.998972000	-2.173255000	-3.360927000
H	-1.655913000	-3.729955000	-2.637232000
H	-3.338454000	-4.080694000	-3.016167000
C	3.293831000	-1.308361000	-0.361904000
H	3.321695000	-1.801230000	-1.335590000
H	3.413066000	-2.064470000	0.418591000
C	4.363773000	-0.295180000	-0.254967000
C	4.663037000	0.683586000	-1.364475000
H	4.461120000	0.161281000	0.734340000
C	6.806742000	-0.375255000	-1.128347000
C	6.163002000	0.986773000	-1.295236000
H	4.385770000	0.255882000	-2.331331000
H	4.066154000	1.588228000	-1.220714000
H	7.758041000	-0.382762000	-0.594752000
H	6.894128000	-0.932941000	-2.061261000
H	6.523349000	1.484086000	-2.198763000
H	6.395943000	1.633908000	-0.443110000
H	6.159317000	-1.345952000	0.568532000
O	5.818521000	-1.141560000	-0.316984000
C	1.369884000	-0.168635000	-2.067600000
H	1.273550000	-1.080358000	-2.656437000
H	1.643733000	1.063347000	-3.738380000

$4v^{B}_{OH6}$

Sum of electronic and zero-point Energies = -1970.939048
Sum of electronic and thermal Free Energies = -1971.005178
Esolv = -1972.2898644

C	1.428020000	-0.579934000	-1.939803000
C	1.648678000	0.209138000	-2.986864000
H	1.819356000	1.279766000	-2.918736000
Au	1.405153000	-0.108002000	0.031360000
P	-1.004419000	0.002620000	0.217842000
C	-1.150606000	0.159878000	2.038719000
C	-1.741514000	1.606998000	-0.505110000
C	-1.821006000	-1.637606000	-0.298933000
C	-2.409676000	0.251926000	2.661509000
C	-0.008438000	0.263580000	2.842701000
C	-1.186922000	2.785637000	0.330855000
C	-3.286483000	1.633010000	-0.456596000
C	-1.278159000	1.795828000	-1.966319000
C	-3.201375000	-1.880751000	0.356430000
C	-1.983417000	-1.684961000	-1.835543000
C	-0.875917000	-2.774757000	0.162140000
H	-3.310774000	0.185996000	2.066656000
C	-2.532259000	0.429523000	4.033494000
C	-0.134639000	0.441016000	4.223898000
N	1.355951000	0.183152000	2.272611000
H	-1.485176000	2.690318000	1.380727000
H	-0.086302000	2.781625000	0.296178000
C	-1.713142000	4.121354000	-0.230864000
C	-3.799552000	2.977139000	-1.013537000
H	-3.647312000	1.518958000	0.570557000
H	-3.703484000	0.812619000	-1.050842000
C	-1.813083000	3.129006000	-2.523243000
H	-1.616358000	0.968430000	-2.597688000
H	-0.184902000	1.801865000	-2.002887000
H	-3.104956000	-1.891130000	1.446616000
H	-3.907773000	-1.088254000	0.086687000
C	-3.762881000	-3.245687000	-0.095491000
C	-2.527938000	-3.062321000	-2.261091000
H	-2.684134000	-0.908761000	-2.162591000
H	-1.029053000	-1.488860000	-2.336667000

C	-1.436667000	-4.141161000	-0.278361000
H	-0.770346000	-2.751561000	1.255624000
H	0.126296000	-2.642028000	-0.268254000
H	-3.517240000	0.495797000	4.484110000
C	-1.387541000	0.523209000	4.819149000
H	0.743414000	0.518843000	4.856346000
C	2.039873000	-1.000551000	2.870216000
C	2.099736000	1.433202000	2.600414000
H	-1.309716000	4.933934000	0.384783000
C	-3.247067000	4.129285000	-0.163691000
C	-1.257784000	4.288484000	-1.686112000
H	-4.894289000	2.968031000	-0.961761000
C	-3.346230000	3.135316000	-2.470109000
H	-1.476376000	3.223514000	-3.562360000
H	-4.744158000	-3.376634000	0.374725000
C	-2.813785000	-4.360457000	0.361560000
C	-3.905838000	-3.278117000	-1.622068000
C	-1.563186000	-4.167046000	-1.808305000
H	-2.617494000	-3.071280000	-3.353458000
H	-0.742172000	-4.922356000	0.053366000
H	-1.464402000	0.661974000	5.892772000
H	3.047954000	-1.090411000	2.464676000
H	1.469891000	-1.900652000	2.635163000
H	2.116185000	-0.903611000	3.956679000
H	2.207507000	1.559392000	3.681132000
H	1.556432000	2.288410000	2.196753000
H	3.096234000	1.393256000	2.159763000
H	-3.631833000	5.086368000	-0.533806000
H	-3.585091000	4.034762000	0.876278000
H	-0.160838000	4.311520000	-1.743924000
H	-1.613444000	5.246136000	-2.083032000
H	-3.754427000	2.325404000	-3.088140000
H	-3.733259000	4.073140000	-2.884622000
H	-3.211268000	-5.338548000	0.067604000
H	-2.730509000	-4.368960000	1.455961000
H	-4.607559000	-2.504797000	-1.959432000
H	-4.322746000	-4.240884000	-1.939021000
H	-0.578671000	-4.031522000	-2.277415000
H	-1.932685000	-5.145286000	-2.136125000
C	4.094822000	-1.308206000	-0.742956000
H	3.829924000	-1.381197000	-1.797378000
H	3.913739000	-2.256409000	-0.234815000
C	3.576900000	-0.085570000	-0.061184000
C	4.123093000	1.187656000	-0.715490000
H	3.882372000	-0.116460000	0.992483000
C	6.188267000	-0.039945000	-1.483201000
C	5.659855000	1.183741000	-0.772037000
H	3.731180000	1.278623000	-1.734743000
H	3.790225000	2.075218000	-0.169009000
H	7.272352000	-0.160180000	-1.449757000
H	5.844951000	-0.129327000	-2.515536000
H	6.032061000	2.060914000	-1.314170000
H	6.089853000	1.243345000	0.236385000
H	6.045582000	-1.400474000	0.041774000
O	5.649209000	-1.284015000	-0.840206000
H	1.631293000	-0.207709000	-3.993046000
H	1.240973000	-1.645115000	-2.081358000

5v $^{B}_{O6}$

Sum of electronic and zero-point Energies = -1970.682261
Sum of electronic and thermal Free Energies = -1970.748861
Esolv = -1971.8789714

C	-1.516231000	0.378820000	-2.056496000
C	-1.627110000	-0.478001000	-3.066564000
H	-1.726458000	-1.552046000	-2.937793000
Au	-1.443776000	-0.009445000	-0.076571000
P	0.993119000	0.020374000	0.225957000
C	1.085170000	-0.091584000	2.056626000
C	1.892035000	-1.525193000	-0.431058000
C	1.744524000	1.694795000	-0.267821000
C	2.318911000	-0.107134000	2.733623000
C	-0.086204000	-0.220679000	2.817986000
C	1.383740000	-2.728328000	0.400545000
C	3.432437000	-1.467230000	-0.343391000
C	1.483887000	-1.758879000	-1.904128000
C	3.099070000	2.036883000	0.392114000
C	1.908575000	1.747898000	-1.803993000
C	0.720443000	2.771840000	0.170480000
H	3.237856000	-0.018838000	2.168914000
C	2.395962000	-0.236296000	4.113954000
C	-0.003732000	-0.348147000	4.209255000
N	-1.428365000	-0.221415000	2.202039000
H	1.646810000	-2.603615000	1.456985000
H	0.286826000	-2.785597000	0.339070000
C	1.998584000	-4.037345000	-0.128331000
C	4.038184000	-2.784354000	-0.868327000
H	3.759538000	-1.316439000	0.690899000
H	3.817108000	-0.630774000	-0.937681000
C	2.104434000	-3.066194000	-2.429236000
H	1.802579000	-0.923019000	-2.534751000
H	0.393955000	-1.811409000	-1.979976000
H	2.995191000	2.044052000	1.482154000
H	3.858976000	1.291029000	0.134032000
C	3.571257000	3.432534000	-0.064128000
C	2.364808000	3.153122000	-2.237946000
H	2.658362000	1.016343000	-2.126355000
H	0.967518000	1.486052000	-2.301614000
C	1.191030000	4.169620000	-0.271281000
H	0.600579000	2.747966000	1.262312000
H	-0.263391000	2.564496000	-0.270966000
H	3.364083000	-0.244480000	4.604575000
C	1.224983000	-0.355929000	4.856448000
H	-0.904931000	-0.444014000	4.805583000
C	-2.191798000	0.944757000	2.731807000
C	-2.125492000	-1.493827000	2.537423000
H	1.625655000	-4.866422000	0.485312000
C	3.528247000	-3.958127000	-0.020799000
C	1.593388000	-4.246631000	-1.593114000
H	5.129766000	-2.715514000	-0.788736000
C	3.633923000	-2.987527000	-2.334445000
H	1.802705000	-3.193188000	-3.475923000
H	4.539223000	3.636476000	0.409225000
C	2.545551000	4.484954000	0.376819000
C	3.721001000	3.464389000	-1.590850000
C	1.328366000	4.197304000	-1.800203000
H	2.462210000	3.161846000	-3.330134000
H	0.443140000	4.905029000	0.049320000
H	1.263346000	-0.457087000	5.936511000
H	-3.177543000	0.983601000	2.268736000
H	-1.647877000	1.861530000	2.499378000
H	-2.320700000	0.872138000	3.816158000
H	-2.276984000	-1.594013000	3.616676000
H	-1.525194000	-2.332913000	2.182873000
H	-3.099659000	-1.514006000	2.048715000
H	3.977671000	-4.895896000	-0.368948000
H	3.830486000	-3.831691000	1.027127000
H	0.501689000	-4.329939000	-1.678547000
H	2.013188000	-5.187507000	-1.969025000
H	4.012514000	-2.162448000	-2.951794000
H	4.084654000	-3.907897000	-2.724971000

H	2.880252000	5.486151000	0.079800000
H	2.452705000	4.492514000	1.470714000
H	4.474095000	2.735081000	-1.916792000
H	4.076132000	4.450890000	-1.912454000
H	0.358641000	3.994583000	-2.274147000
H	1.638042000	5.195862000	-2.131102000
C	-4.206964000	1.192896000	-0.756096000
H	-3.981287000	1.292879000	-1.829689000
H	-3.851953000	2.095660000	-0.247558000
C	-3.564718000	-0.070953000	-0.190101000
C	-4.141139000	-1.306917000	-0.875556000
H	-3.845466000	-0.111040000	0.870200000
C	-6.205779000	0.076056000	-1.262709000
C	-5.676923000	-1.269798000	-0.779486000
H	-3.849321000	-1.315734000	-1.931657000
H	-3.759174000	-2.233498000	-0.428538000
H	-7.282900000	0.163952000	-1.097738000
H	-6.017691000	0.191584000	-2.343930000
H	-6.115999000	-2.081327000	-1.371672000
H	-5.990946000	-1.421318000	0.260891000
O	-5.607931000	1.153348000	-0.555594000
H	-1.625707000	-0.115529000	-4.093290000
H	-1.443289000	1.450371000	-2.246284000

TS1'v$_{OH6E}$
Sum of electronic and zero-point Energies = -2010.210896
Sum of electronic and thermal Free Energies = -2010.280312
Esolv = -2011.574858

C	1.065623000	0.407176000	-2.147581000
Au	1.273369000	-0.153182000	-0.202439000
P	-1.089982000	0.064311000	0.245405000
C	-1.062981000	-0.166217000	2.062729000
C	-1.647671000	1.854824000	-0.115588000
C	-2.158649000	-1.353547000	-0.460428000
C	-2.247528000	-0.058208000	2.816619000
C	0.117564000	-0.513483000	2.731291000
C	-0.464982000	2.785067000	0.251310000
C	-2.866222000	2.294742000	0.734320000
C	-2.007396000	2.013093000	-1.610483000
C	-3.663634000	-1.131704000	-0.176804000
C	-1.947808000	-1.502370000	-1.982529000
C	-1.698476000	-2.662711000	0.222185000
H	-3.176795000	0.201908000	2.328800000
C	-2.259101000	-0.274852000	4.188230000
C	0.099995000	-0.735318000	4.111953000
N	1.396530000	-0.697692000	2.009652000
H	-0.226488000	2.682488000	1.318951000
H	0.435707000	2.511594000	-0.313821000
C	-0.827161000	4.249660000	-0.065665000
C	-3.230058000	3.759595000	0.406824000
H	-2.627563000	2.231106000	1.800195000
H	-3.732467000	1.652812000	0.539859000
C	-2.343151000	3.486864000	-1.912218000
H	-2.878964000	1.394654000	-1.851868000
H	-1.187319000	1.676814000	-2.252534000
H	-3.850421000	-1.042568000	0.897568000
H	-4.014551000	-0.212698000	-0.658532000
C	-4.471437000	-2.331617000	-0.715943000
C	-2.775512000	-2.688543000	-2.515117000
H	-2.230288000	-0.587804000	-2.512111000
H	-0.889524000	-1.680279000	-2.189598000
C	-2.520843000	-3.850990000	-0.316902000
H	-1.820571000	-2.598388000	1.309444000
H	-0.631526000	-2.833184000	0.012287000
H	-3.187476000	-0.180104000	4.742057000
C	-1.077382000	-0.613495000	4.839649000
H	1.004472000	-1.014658000	4.641542000
C	1.791275000	-2.132389000	2.124943000
C	2.436683000	0.164895000	2.635890000
H	0.033223000	4.878961000	0.191234000
C	-2.047326000	4.667436000	0.764631000
C	-1.144416000	4.379614000	-1.562350000
H	-4.102405000	4.027442000	1.013889000
C	-3.564276000	3.902614000	-1.082654000
H	-2.570709000	3.571206000	-2.980961000
H	-5.529265000	-2.147848000	-0.495803000
C	-4.007976000	-3.616171000	-0.016395000
C	-4.263976000	-2.452544000	-2.230153000
C	-2.311752000	-3.980808000	-1.831011000
H	-2.609501000	-2.757839000	-3.596453000
H	-2.177841000	-4.760705000	0.189733000
H	-1.067506000	-0.788683000	5.910643000
H	2.739446000	-2.295060000	1.614483000
H	1.020482000	-2.757302000	1.673058000
H	1.909915000	-2.422785000	3.172904000
H	2.568568000	-0.070127000	3.694974000
H	2.145613000	1.211988000	2.539700000
H	3.387362000	0.001390000	2.132520000
H	-2.299705000	5.713781000	0.558940000
H	-1.822433000	4.601470000	1.836862000
H	-0.269615000	4.101482000	-2.166324000
H	-1.371427000	5.423472000	-1.806387000
H	-4.431849000	3.283710000	-1.344341000
H	-3.838992000	4.939616000	-1.306545000
H	-4.598590000	-4.469034000	-0.369325000
H	-4.173789000	-3.544813000	1.066258000
H	-4.612791000	-1.544629000	-2.738472000
H	-4.859496000	-3.282985000	-2.625920000
H	-1.254515000	-4.175945000	-2.056924000
H	-2.877801000	-4.836696000	-2.215564000
C	3.477742000	0.020497000	-0.797229000
C	4.048399000	1.356008000	-0.318305000
C	4.115035000	-1.170850000	-0.353974000
H	3.347119000	0.011277000	-1.879042000
C	5.473345000	1.574673000	-0.850001000
H	4.066440000	1.423613000	0.774563000
H	3.410331000	2.172920000	-0.669793000
H	4.429049000	-1.230066000	0.688033000
C	6.461572000	0.525953000	-0.363957000
H	5.837392000	2.555346000	-0.523605000
H	5.467297000	1.601797000	-1.947632000
H	7.474405000	0.713043000	-0.733712000
H	6.508241000	0.489747000	0.728123000
H	6.202871000	-0.901837000	-1.720038000
O	6.079921000	-0.816123000	-0.762084000
C	3.992678000	-2.480479000	-1.055848000
H	3.174145000	-3.062277000	-0.614550000
H	3.786437000	-2.362135000	-2.121857000
H	4.898463000	-3.075741000	-0.913486000
C	1.071777000	-0.386142000	-3.211804000
H	1.135004000	-1.469572000	-3.165945000
H	1.003348000	0.049018000	-4.208144000
H	1.023509000	1.490954000	-2.242460000

4v$_{OH6E}$'

Sum of electronic and zero-point Energies = -2010.211945
Sum of electronic and thermal Free Energies = -2010.280264
Esolv = -2011.581089

C	1.095724000	0.500924000	-2.142110000
Au	1.307828000	-0.054589000	-0.201124000
P	-1.077806000	0.033932000	0.247085000
C	-1.056825000	-0.155435000	2.069432000
C	-1.768413000	1.771947000	-0.139241000
C	-2.063345000	-1.452448000	-0.433503000
C	-2.248687000	-0.114366000	2.817647000
C	0.143101000	-0.390839000	2.751919000
C	-0.660854000	2.789802000	0.230298000
C	-3.029028000	2.140075000	0.680278000
C	-2.107917000	1.884757000	-1.643335000
C	-3.587631000	-1.307910000	-0.217979000
C	-1.779949000	-1.637003000	-1.940741000
C	-1.565304000	-2.717240000	0.305547000
H	-3.193013000	0.049662000	2.316643000
C	-2.249929000	-0.277956000	4.196577000
C	0.138125000	-0.555746000	4.141043000
N	1.433350000	-0.528588000	2.036915000
H	-0.434537000	2.721050000	1.303309000
H	0.266996000	2.569965000	-0.314151000
C	-1.118128000	4.220432000	-0.116236000
C	-3.489671000	3.569769000	0.323461000
H	-2.805853000	2.109568000	1.751126000
H	-3.843940000	1.434888000	0.483768000
C	-2.542801000	3.325269000	-1.975467000
H	-2.925959000	1.200464000	-1.894335000
H	-1.251258000	1.600886000	-2.262915000
H	-3.822688000	-1.195783000	0.845291000
H	-3.967388000	-0.423038000	-0.739885000
C	-4.308454000	-2.562904000	-0.752137000
C	-2.520861000	-2.879749000	-2.470434000
H	-2.084635000	-0.753925000	-2.510592000
H	-0.704394000	-1.763466000	-2.096371000
C	-2.299744000	-3.963359000	-0.228430000
H	-1.734623000	-2.628404000	1.384607000
H	-0.482683000	-2.836881000	0.145436000
H	-3.185316000	-0.237471000	4.745241000
C	-1.047525000	-0.493503000	4.862650000
H	1.058784000	-0.745808000	4.682329000
C	1.902059000	-1.932733000	2.211141000
C	2.423857000	0.411940000	2.626835000
H	-0.309187000	4.913253000	0.144977000
C	-2.379825000	4.564223000	0.685692000
C	-1.417093000	4.306411000	-1.619814000
H	-4.389691000	3.786692000	0.910132000
C	-3.805320000	3.666170000	-1.173948000
H	-2.756463000	3.377762000	-3.049241000
H	-5.383358000	-2.432151000	-0.582651000
C	-3.808618000	-3.801224000	0.003650000
C	-4.030432000	-2.715433000	-2.252697000
C	-2.020782000	-4.125659000	-1.728137000
H	-2.307195000	-2.972388000	-3.541765000
H	-1.931199000	-4.838772000	0.319331000
H	-1.028123000	-0.622553000	5.940059000
H	2.864937000	-2.063086000	1.719881000
H	1.172545000	-2.613863000	1.771925000
H	2.019564000	-2.179635000	3.270478000
H	2.575246000	0.223675000	3.692887000
H	2.073719000	1.436867000	2.495043000
H	3.379551000	0.286033000	2.120968000
H	-2.700853000	5.587188000	0.458524000
H	-2.170238000	4.530272000	1.762559000
H	-0.514172000	4.081805000	-2.204578000
H	-1.712968000	5.328002000	-1.884057000
H	-4.621693000	2.982846000	-1.440185000
H	-4.148599000	4.677482000	-1.420199000
H	-4.338034000	-4.694385000	-0.347059000
H	-4.023331000	-3.708320000	1.076042000
H	-4.403827000	-1.841617000	-2.801798000
H	-4.564845000	-3.587048000	-2.647454000
H	-0.945994000	-4.270602000	-1.904505000
H	-2.524976000	-5.021126000	-2.108988000
C	3.434787000	0.080226000	-0.749211000
C	3.996768000	1.469304000	-0.410886000
C	4.304358000	-1.044474000	-0.286102000
H	3.367466000	-0.004666000	-1.838344000
C	5.420836000	1.659319000	-0.952961000
H	4.006143000	1.651260000	0.669862000
H	3.351352000	2.239001000	-0.844461000
H	4.511422000	-1.056372000	0.787386000
C	6.348833000	0.582821000	-0.445065000
H	5.832077000	2.626567000	-0.642109000
H	5.419472000	1.663995000	-2.050539000
H	7.349328000	0.595076000	-0.881664000
H	6.435005000	0.557714000	0.643174000
H	5.841460000	-0.905080000	-1.744186000
O	5.803415000	-0.765188000	-0.780205000
C	4.031811000	-2.422938000	-0.837441000
H	3.071613000	-2.782201000	-0.459495000
H	3.955256000	-2.406874000	-1.929944000
H	4.801408000	-3.137663000	-0.536552000
C	1.155739000	-0.272913000	-3.219556000
H	1.294454000	-1.349921000	-3.188248000
H	1.044554000	0.168974000	-4.208700000
H	0.973413000	1.579994000	-2.224700000

4v$_{O6E}$'

Sum of electronic and zero-point Energies = -2009.952197
Sum of electronic and thermal Free Energies = -2010.020293
Esolv = -2011.16460315

C	1.172794000	0.457019000	-2.219393000
Au	1.357294000	-0.046766000	-0.270930000
P	-1.056482000	0.027156000	0.253756000
C	-1.009741000	-0.129990000	2.080316000
C	-1.773228000	1.753692000	-0.122746000
C	-2.091834000	-1.443877000	-0.376101000
C	-2.183275000	-0.084921000	2.856351000
C	0.211620000	-0.333637000	2.739203000
C	-0.663662000	2.773968000	0.236607000
C	-3.035842000	2.136702000	0.682489000
C	-2.095947000	1.860380000	-1.630953000
C	-3.614699000	-1.297461000	-0.166442000
C	-1.809300000	-1.653720000	-1.881953000
C	-1.599935000	-2.703472000	0.377103000
H	-3.139592000	0.052366000	2.369223000
C	-2.153773000	-0.211885000	4.238336000
C	0.235108000	-0.461071000	4.133471000
N	1.485953000	-0.485875000	2.006810000
H	-0.435090000	2.712131000	1.309367000
H	0.261918000	2.542031000	-0.305411000
C	-1.113217000	4.202926000	-0.120116000
C	-3.490210000	3.563572000	0.312233000
H	-2.819479000	2.111566000	1.755236000
H	-3.852048000	1.431826000	0.489467000
C	-2.527602000	3.296785000	-1.979878000

H	-2.910262000	1.173684000	-1.888944000
H	-1.228540000	1.571155000	-2.234305000
H	-3.850692000	-1.165491000	0.894984000
H	-3.991665000	-0.418511000	-0.700939000
C	-4.340202000	-2.555777000	-0.683172000
C	-2.549680000	-2.902055000	-2.395431000
H	-2.117712000	-0.779321000	-2.463458000
H	-0.733063000	-1.772481000	-2.039990000
C	-2.335795000	-3.954561000	-0.138309000
H	-1.768641000	-2.598081000	1.454909000
H	-0.518404000	-2.827352000	0.221695000
H	-3.077220000	-0.169759000	4.807096000
C	-0.933100000	-0.394040000	4.881053000
H	1.171412000	-0.627670000	4.654784000
C	1.945986000	-1.891534000	2.186501000
C	2.498493000	0.448759000	2.563821000
H	-0.303615000	4.895866000	0.139603000
C	-2.378713000	4.558224000	0.670962000
C	-1.404309000	4.281123000	-1.625587000
H	-4.394224000	3.792897000	0.889159000
C	-3.794594000	3.647751000	-1.188894000
H	-2.735270000	3.342114000	-3.055679000
H	-5.416307000	-2.422807000	-0.518851000
C	-3.844802000	-3.786578000	0.088410000
C	-4.059580000	-2.732208000	-2.181350000
C	-2.055220000	-4.139507000	-1.635250000
H	-2.335751000	-3.012406000	-3.465364000
H	-1.970505000	-4.824400000	0.421112000
H	-0.886494000	-0.494831000	5.960854000
H	2.891054000	-2.034286000	1.667291000
H	1.198245000	-2.569603000	1.773908000
H	2.086050000	-2.124875000	3.247387000
H	2.677141000	0.267591000	3.627911000
H	2.154099000	1.475445000	2.430184000
H	3.436566000	0.308226000	2.031433000
H	-2.695715000	5.581021000	0.434033000
H	-2.174949000	4.530261000	1.749347000
H	-0.498507000	4.050139000	-2.202221000
H	-1.699096000	5.301558000	-1.898411000
H	-4.609824000	2.961679000	-1.453369000
H	-4.134847000	4.657914000	-1.446944000
H	-4.377019000	-4.683067000	-0.251645000
H	-4.059858000	-3.677352000	1.159528000
H	-4.429296000	-1.864218000	-2.742755000
H	-4.596401000	-3.608665000	-2.563985000
H	-0.980678000	-4.287923000	-1.806481000
H	-2.561899000	-5.039176000	-2.004902000
C	3.444527000	0.078592000	-0.807180000
C	3.993423000	1.472733000	-0.488282000
C	4.356419000	-1.047218000	-0.301476000
H	3.441244000	-0.030960000	-1.893870000
C	5.461836000	1.572976000	-0.930975000
H	3.939655000	1.696988000	0.584070000
H	3.398391000	2.241566000	-0.995311000
H	4.415266000	-1.043889000	0.803049000
C	6.245240000	0.392250000	-0.379689000
H	5.901835000	2.517848000	-0.591078000
H	5.522252000	1.556559000	-2.025417000
H	7.275431000	0.384289000	-0.745670000
H	6.281086000	0.435353000	0.723831000
O	5.672141000	-0.835335000	-0.797888000
C	3.952855000	-2.424005000	-0.805279000
H	2.937464000	-2.691534000	-0.496987000
H	3.991232000	-2.435335000	-1.898366000
H	4.646393000	-3.183912000	-0.436213000
C	1.182964000	-0.350521000	-3.274572000
H	1.321585000	-1.425741000	-3.208100000
H	1.056768000	0.059470000	-4.275294000
H	1.081754000	1.536577000	-2.339909000
